# Developing an efficient protocol for monitoring eagle fatalities at wind energy facilities

**DOI:** 10.1371/journal.pone.0208700

**Published:** 2018-12-12

**Authors:** Eric C. Hallingstad, Paul A. Rabie, Andrew C. Telander, Jerry A. Roppe, Laura R. Nagy

**Affiliations:** 1 Western EcoSystems Technology, Inc., Cheyenne, Wyoming, United States of America; 2 Avangrid Renewables, LLC, Portland, Oregon, United States of America; Pacific Northwest National Laboratory, UNITED STATES

## Abstract

Researchers typically conduct fatality monitoring to determine a wind energy facility’s direct impacts on wildlife. In the United States, wind energy impacts on eagles have received increased attention in recent years because eagle incidental take permits became available. Permit holders are required to conduct fatality monitoring to evaluate compliance with permitted eagle take. Our objective was to develop an efficient eagle fatality monitoring protocol with a quantifiable detection probability based on a stationary scanning search method. We conducted scanning searches for eagle carcasses at four wind energy facilities. We estimated searcher efficiency of the scanning search method using feathered turkey decoys as eagle carcass surrogates, used publicly available data on large raptor carcass distances from turbines to evaluate the proportion of carcasses expected to occur in searched areas, and estimated carcass persistence rates for game birds and raptors. These three bias adjustments were combined to estimate the overall probability of detection for the scanning search method. We found generally high searcher efficiency for the scanning search method, with 76% of decoys detected; however, detection decreased with distance and difficulty of visibility class. Mean carcass persistence time varied between 28 and 76 days for raptors and between three and nine days for game birds, showing that game birds do not persist as long as raptors. We estimated that 95% of large avian carcasses fall within 100 m of turbine bases, and 99% fall within 150 m. Using these estimates and assuming a 30-day search interval for all facility turbines, we estimated that the probability of detecting a large raptor carcass using the scanning search method at a wind facility ranged from 0.50 to 0.69. Our research suggests a monitoring program that uses scanning searches can be a cost-effective approach for gathering data necessary to meet incidental eagle take permit requirements.

## Introduction

Growing concerns over climate change and carbon emissions have prompted state and national governments to prioritize the development of wind energy resources. As a result, global wind energy production has been continuously increasing during the past 25 years, with an average of 25% growth each year and a world-wide total of 487 gigawatts (GW) of operating wind capacity in 2016 [1]. In the USA, the Environmental Protection Agency announced the Clean Power Plan [2] on August 3, 2015, which emphasized developing renewable energy sources, such as wind and solar, to reduce reliance on fossil fuels. An increase in wind capacity of 69 GW is projected world-wide for 2018 and 2019 [3].

Wind energy is considered a “green” energy source, but concerns exist over risks to wildlife from wind facilities [4–7]. In particular, the impacts of wind facilities on bald (*Haliaeetus leucocephalus*) and golden (*Aquila chrysaetos*) eagles have received attention in the USA; both species are susceptible to collisions with wind turbines [8, 9]. While data from the US Fish and Wildlife Service (USFWS) suggest that shooting, poisoning, starvation, and electrocutions are the primary sources of mortality for golden eagles [10], vulnerability to sources of mortality is known to vary geographically [8, 11].

In the USA, the Bald and Golden Eagle Protection Act of 1940 [12] prohibits eagle take without a permit. In September 2009, the USFWS set in place rules establishing eagle take permits for incidental take (50 Code of Federal Regulations 22.26 and 22.27) [13]; the rules were revised in 2016 [14]. The USFWS requires permit holders to conduct fatality monitoring to ensure compliance with permit conditions, and monitoring “should be rigorous and sufficient to yield a reasonable estimate of the mean annual eagle fatality rate for the project” [15].

Regardless of location, fatality monitoring involves searching for carcasses under turbines and adjusting the number of carcasses found to account for the bias associated with these searches. First, the ability of a searcher to detect a carcass, or *searcher efficiency*, needs to be determined because not all carcasses present are found by the searchers [16, 17]. Second, a *carcass density distribution* needs to be estimated because carcasses fall at different distances from turbines and some carcasses may fall beyond the bounds of the searched area [18]. Third, the average probability that a carcass will be available for detection during the next search, or *carcass persistence*, must be estimated because carcasses can be removed by scavengers or other means and become unavailable for detection [16, 17]. These three bias adjustments are used to calculate an overall probability of detection, or the likelihood a carcass will be present and found during a search. The numbers of carcasses detected during searches are then adjusted using the overall probability of detection to estimate the total fatality rates at a facility by using one of a number of fatality estimators [19–24].

The exact form and intensity of eagle fatality monitoring required under eagle take permits issued in the USA has not been explicitly defined. “Standard” fatality monitoring studies at wind farms are designed to estimate the overall fatality rates for birds and bats at wind facilities [16, 17], rather than species-specific fatality rates. In the USA, these studies are typically conducted at approximately 30% of turbines searched every seven to 28 days via walking transects spaced 3–10 m apart [16, 17]. Two of the challenges of standard fatality monitoring are the high level of effort and the associated costs. These challenges may be minimized when searching for a particular species of concern because search methods can be adjusted to account for differences in carcass detectability, carcass distribution, and carcass persistence. For example, when monitoring is focused on eagles or other larger birds, searcher efficiency is higher than for smaller species because large carcasses are easier to detect [25, 26]. Higher detectability of large carcasses means that searchers may be able to efficiently and effectively search large plots by scanning the search area from fixed locations (turbine pads), as opposed to using the relatively time-consuming process of walking transects. Furthermore, research indicates that large search plots are necessary to capture a reasonable proportion of eagle carcasses, as the distribution of large carcasses extends farther from the turbine than that of smaller carcasses [18]. Finally, carcasses of eagles and other large birds persist for more time than do those of smaller birds [18, 27], allowing for longer search intervals (i.e., more days between searches). Modified monitoring methods that leverage these differences may reduce effort with minimal reduction of detection probability, providing an efficient eagle fatality monitoring technique for eagle take permit compliance.

Our goal was to develop a cost-effective eagle fatality monitoring protocol by replacing standard transect-based carcass searches with a scan-from-the-turbine-pad carcass search (hereafter scanning search), and to develop the methods necessary to quantify the resulting detection probability. As such, we were not interested in strict comparisons between our methods and existing methods, but rather in the overarching question of whether our methods appear viable in the context of eagle take permit compliance for incidental take. Our objectives were to: 1) estimate searcher efficiency using the scanning search method, 2) estimate the proportion of eagle carcasses occurring within our search areas using existing data on large raptor fatalities, 3) estimate carcass persistence probability of raptors and compare it to game bird persistence, 4) use searcher efficiency, raptor carcass density distribution, and raptor carcass persistence probability to estimate an overall probability of detection, and 5) demonstrate how the overall probability of detection from the scanning search method can be used to develop take estimates for compliance assessment.

## Study areas

**Fig 1:**
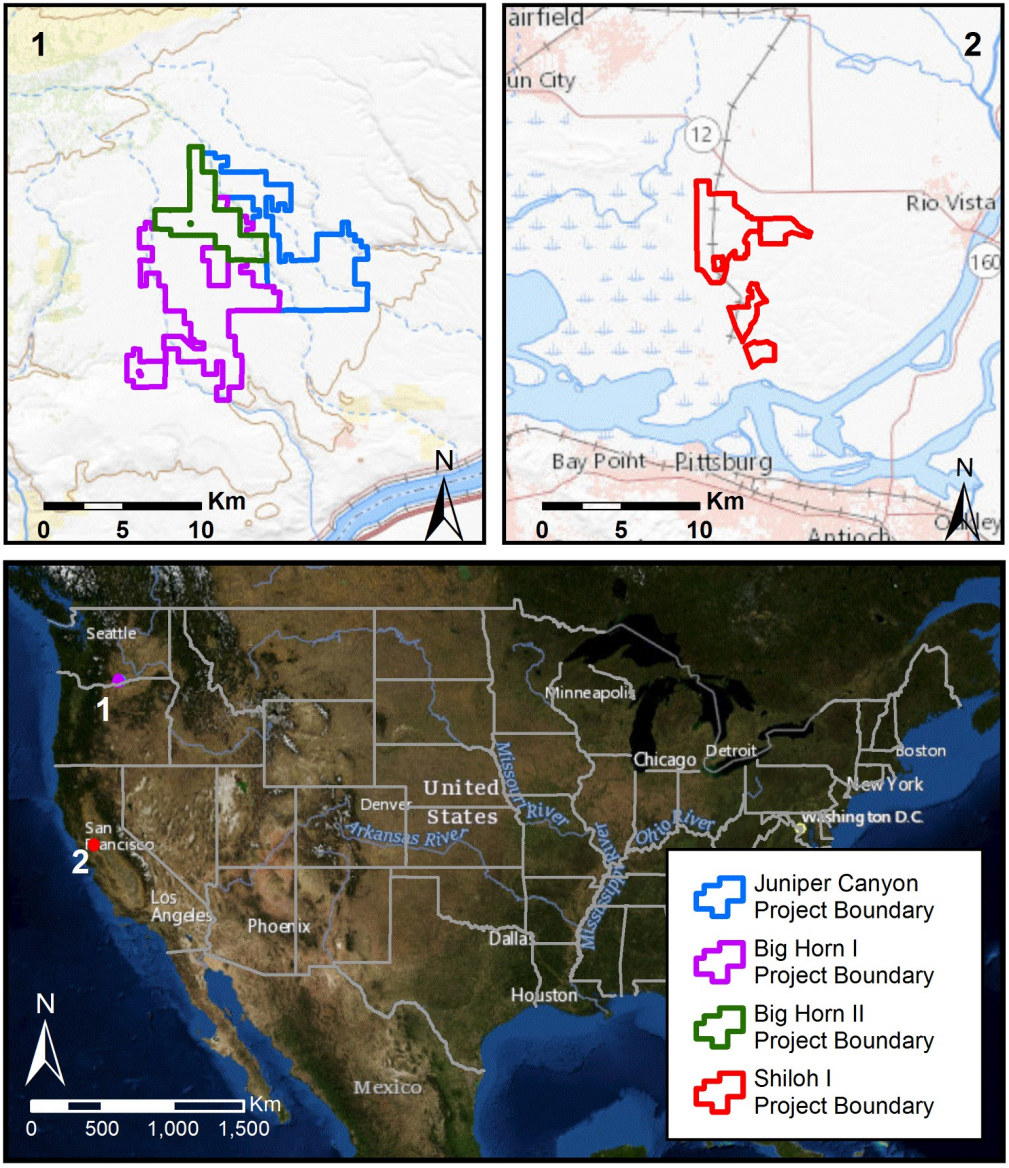
Locations of the Juniper Canyon, Big Horn I, and Big Horn II wind energy facilities in Washington, USA, and the Shiloh I wind energy facility in California, USA.

We included three wind energy facilities in Washington, USA, and one facility in California, USA, in the study. Juniper Canyon (JC), Big Horn I (BH I), and Big Horn II (BH II), three adjacent facilities in southeastern Klickitat County, Washington, lie within the Columbia Plateau Ecoregion (Fig 1). The facilities consist of 221 turbines, with maximum turbine heights ranging from 118.5–127.5 m (ground to tip of the rotor blade). Dominant land uses include dryland agriculture (primarily winter wheat [*Triticum aestivum*]) and cattle (*Bos taurus*) grazing. Non-agricultural lands within the facilities include grasslands and small patches of shrub-steppe. Small isolated patches of deciduous trees and junipers (*Juniperus* spp.) are scattered throughout the area, primarily within canyons that border the facilities.

Shiloh I lies within southeastern Solano County in the Sacramento Valley of California (Fig 1). This area is characterized by a relatively uniform pattern of broad ridgelines with narrow valleys and drainages. This facility consists of 100 turbines, which are no more than 104 m in height from base to blade tip. Approximately 75% of the Shiloh I project area is currently used for crop production (e.g., safflower [*Carthamus tinctorius*], and winter wheat). The remaining 25% is predominantly non-native grasses used for livestock grazing.

## Field methods

We conducted scanning searches for carcasses and bias trials (searcher efficiency and carcass persistence) in the field to gather data necessary to estimate large raptor fatality rates. Methods were broadly similar from trial to trial; however, we made protocol adjustments (e.g., expanded search radii, size of search team reduced) after encouraging results from early trials. These protocol adjustments limit our ability to compare the estimated detection probabilities from site to site; however, such comparisons were never our objective. Rather, our estimated detection probabilities should be taken to illustrate the range of outcomes that may be obtained under a range of field conditions. Despite the protocol adjustments, data from each trial were amenable to analysis using the methods described herein to estimate detection probability. Due to the exploratory nature of our study, we did not conduct trials during all seasons. Table 1 describes the differences between the study periods.

**Table 1:** Field method details for the four study periods. We conducted scanning searches at the Juniper Canyon (JC), Big Horn I (BH I), Big Horn II (BH II), and Shiloh I wind energy facilities. Due to the exploratory nature of our study, we made minor protocol adjustments (e.g., expanded search radii, size of search team reduced) after encouraging results from early trials.

	Fall 2013	Summer 2014	Fall 2014	Summer/Fall 2015
**Study Site**	JC	BH I / BH II	Shiloh I	JC / BH I / BH II
**Searched turbines**	All 63, every search	60 turbines each search, with a rotating selection to include all 158 turbines in the study	All 100 turbines, every search	113 turbines, every search
**Search team size (employer)**	Two searchers (Avangrid Renewables personnel)	Two searchers (Avangrid Renewables personnel)	Two searchers (Avangrid Renewables personnel)	One searcher; (Western EcoSystems Technology, Inc. and Avangrid Renewables personnel)
**Maximum search radius**	100 m	100 m	150 m	150 m
**Search frequency**	Monthly	Twice monthly^a^	Twice weekly^b^	Twice monthly
**Decoys placed**	220	291	296	243
**Carcass persistence trial dates**	Fall/Winter 2014	Used data from JC 2013	Used 2012–2013 data from NextEra Energy’s Montezuma II facility	Summer/Fall 2015
**Carcass persistence bird types and sample sizes**	100 game birds 12 large raptors		26 large raptors	30 game birds 28 large raptors

^a^ We searched each turbine five times during the 12-week trial

^b^ We used a short search interval to increase our searcher efficiency bias trial sample size during this relatively short study period

### Scanning search method

**Fig 2:**
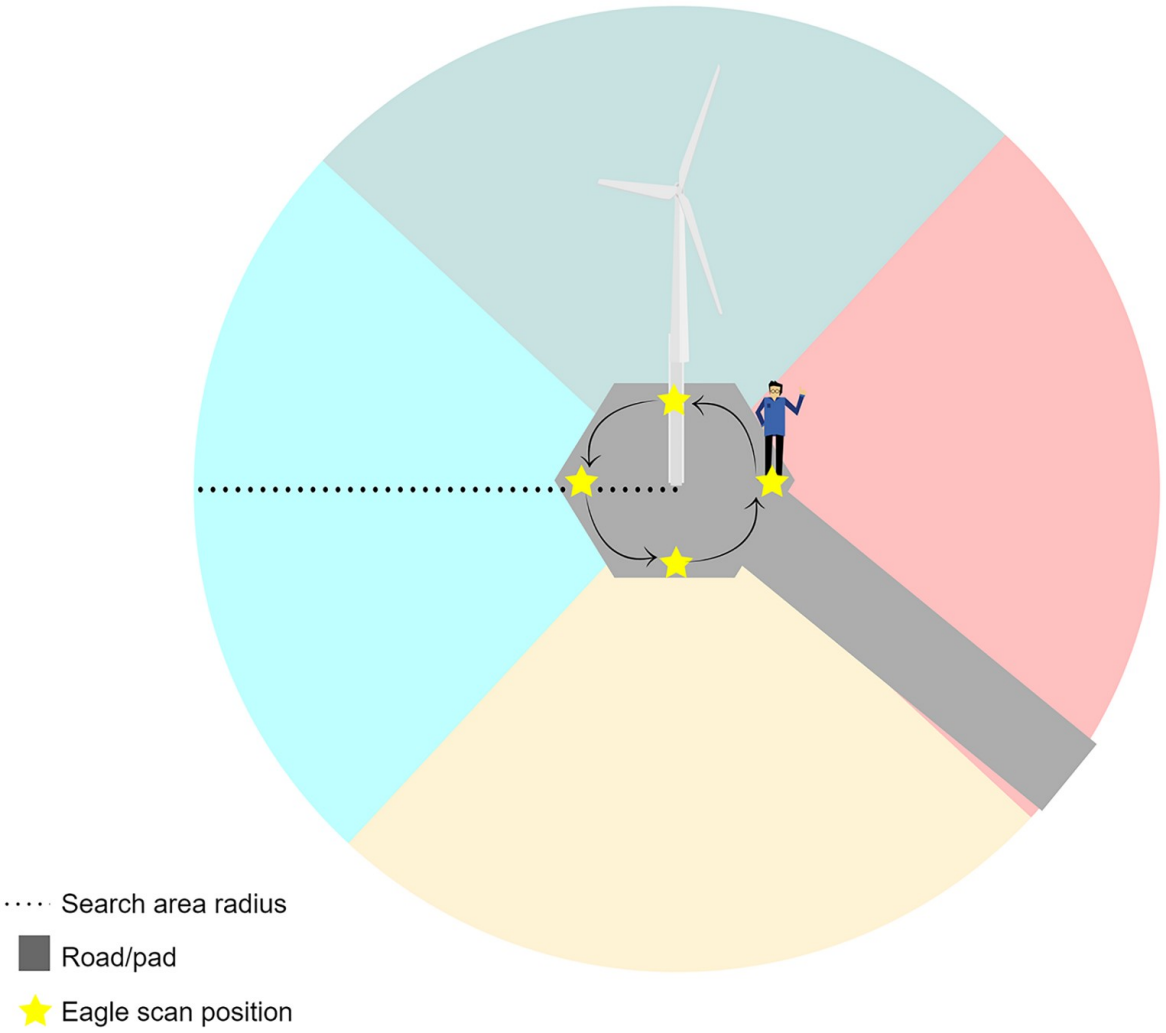
Diagram of eagle scan positions around a turbine (not to scale).

We conducted scanning searches for carcasses at the four facilities between 2013 and 2015 (Table 1). One (2015) or two (2013 and 2014) searchers conducted scanning searches. Both Western EcoSystems Technology, Inc. (WEST) and Avangrid Renewables employees participated in the study (Table 1); we trained all searchers in detection methods and data recording prior to conducting searches. At each turbine base, the searchers scanned the terrain from the four cardinal directions (e.g., 0°, 90°, 180° and 270° from the turbine; Fig 2). For scans using two searchers, the searchers started on opposite sides of the turbine, walked counterclockwise, and scanned each quadrant out to the designated maximum search radius (Table 1) with binoculars until each searcher inspected all four quadrants. For scans using one searcher, the searcher used the same protocol, but each quadrant was only searched once. Searchers were trained to scan the closest third (foreground) of the search plots with their naked eye, and then use binoculars for additional scans (mid-ground, background) until the plot edge was reached (as determined using rangefinders). Scans took less than 10 minutes per turbine. During 2015, searchers also walked transects through areas not visible from the turbine pads (“obstructed view areas”; see *Visibility Mapping* below) to evaluate the additional benefits of comprehensive search plot coverage. Most obstructed view areas consisted of thin bands of shrub-steppe or grasslands that were downslope from the turbine bases, and composed roughly 4% of the overall search area (Table 2). Using a handheld Global Positioning System unit that displayed the obstructed view area boundaries, we conducted only one transect survey within each obstructed view area during each search round; the searcher typically scanned up to 10–15 m on either side of the transect. For all scanning searches, searchers recorded the time, date, nearest turbine number, land cover, visibility class, and vegetation height for each decoy detected (see *Bias Trials* section, below).

**Table 2:** Visibility class definitions and percent coverages within searched areas^a^ during searcher efficiency trials at the Juniper Canyon (JC), Big Horn I (BH I), Big Horn II (BH II), and Shiloh I wind energy facilities.

Visibility Class	Definition	JC 2013 (100-m radius)	BH I/BH II 2014 (100-m radius)	Shiloh I 2014 (150-m radius)	JC and BH I/BH II 2015 (150-m radius)
**Easy**	>90% bare ground, sparse ground cover <15 cm tall	52%	9%	66%	17%
**Moderate**	25–90% bare ground, cover >15 cm tall	34%	79%	3%	66%
**Difficult**	<25% bare ground, >25% cover >30 cm tall	--	--	0%	13%
**Obstructed view**	Out of sight from turbine base due to topography or physical obstruction	--	--	31%	4%

^a^ Missing values indicate areas not searched during a trial period.

### Bias trials

We conducted searcher efficiency trials simultaneously with scanning searches at all four study sites in order to determine searcher efficiency using the scanning search method (Table 1). Searchers conducted carcass persistence trials at JC and BH I/BH II (Table 1), but for Shiloh I, we used previously collected carcass persistence data by H.T. Harvey & Associates at a nearby NextEra Energy (NextEra) facility (Table 1).

#### Searcher efficiency

**Fig 3:**
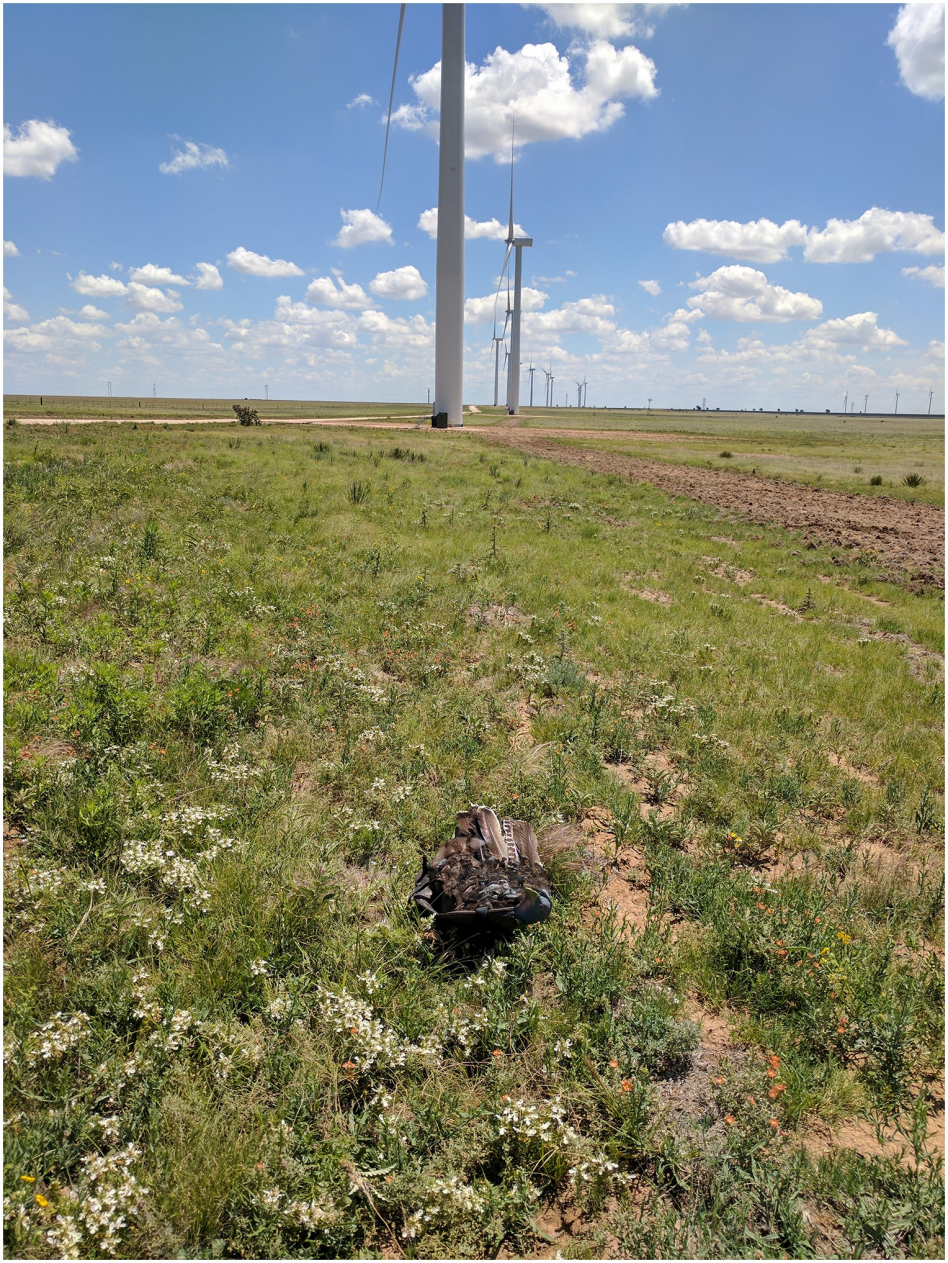
Turkey decoys with feather harnesses were used as a proxy for eagle carcasses during searcher efficiency trials.

Our objective for the searcher efficiency trials was to estimate the percentage of large bird carcasses found by searchers. We used estimates of searcher efficiency to adjust the total number of carcasses found for those potentially missed by searchers, thereby accounting for detection bias [16, 17]. For this study, we based searcher efficiency on decoys detected during scanning searches. We used conventional hunters’ decoys with a feather (feathers from wild turkey [*Meleagris gallopavo*]) harness (hereafter, decoys) as a proxy for large avian carcasses for searcher efficiency trials. The modified decoys were similar to eagle carcasses in terms of size and coloring (Fig 3), with the advantage of not attracting scavengers to areas near turbines. We placed 18 to 23 decoys per search round. Although searchers were aware that their detection rates were being tested, to be consistent with protocols used in searcher efficiency trials conducted as part of standard fatality monitoring studies [16, 17], we prevented searchers from knowing where, when, or exactly how many decoys were deployed. We stratified decoy placement to ensure similar numbers of decoys per team of searchers, and representation of all visibility classes. We selected decoy distances from turbines using a weighted random distribution that put relatively more decoys at greater distances from turbines (where detection was expected to be lower) to improve the precision of searcher efficiency estimates. Following each round of searches, we collected all decoys and placed them in new locations for the subsequent round.

#### Visibility mapping

**Fig 4:**
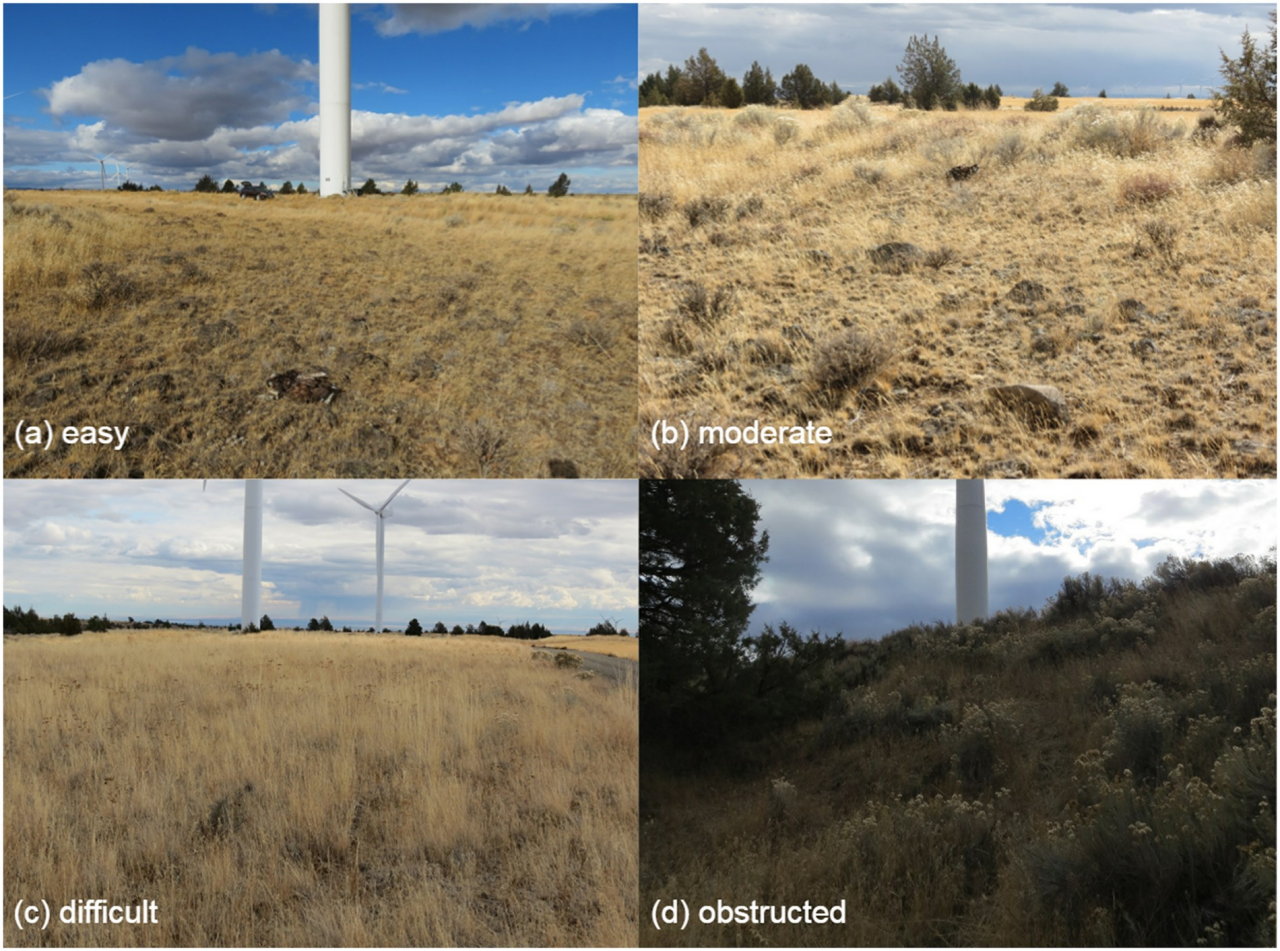
Examples of visibility classes present with search plots. (a) easy, (b) moderate, (c) difficult, (d) obstructed.

We mapped all areas within the search radius of turbines into four visibility classes (easy, moderate, difficult, and obstructed view areas; definitions in Table 2; representative photographs in Fig 4), as we expected detectability to vary by visibility class as well as distance from the searcher. We digitized land cover data from hand-marked aerial photograph field maps using Geographic Information System software (ArcGIS 10.x, Redlands, California). Some land cover types progress through visibility classes during an annual cycle (e.g., agricultural fields), or may vary with precipitation levels in a given year (e.g., grasslands) [28]. For simplicity, our analyses assumed consistent class designations based on the classifications present at the beginning of each of our trials.

#### Carcass persistence

Not all carcasses that fall within search areas persist until the next search. Scavengers remove some carcasses, some carcasses decompose beyond recognition, and other processes (e.g., plowed under by farming equipment) may remove carcasses [16, 17, 25, 26]. Our objective for carcass persistence trials was to estimate the probability that a carcass that arrived in a search area persisted until a scheduled search.

Researchers commonly use game birds in persistence trials associated with standard fatality monitoring and therefore we included game birds in this study. However, medium- to large-sized raptor carcasses likely provide more accurate representation of eagle carcass persistence than game birds [29]; therefore, we also used raptor carcasses in the carcass persistence trials. We obtained raptor carcasses from local rehabbers and airports; similar to our game bird carcasses, all raptor carcasses were intact with no evidence of decomposition or infestation. We placed and monitored game bird (ring-necked pheasant [*Phasianus colchicus*] and hen mallard [*Anas platyrhynchos*]) and medium- to large-sized raptor carcasses (Cooper’s hawk [*Accipiter cooperii*] or larger) within each of the visibility classes at JC and BH I/BH II (Table 1). Researchers collected carcass persistence data from the Montezuma II facility (as applied to Shiloh I; see Table 1) without respect to visibility class. Regardless of bird type (raptor or game bird), we only used intact carcasses with no signs of decomposition.

We placed all persistence trial carcasses in areas similar to the decoy search plots, but outside of the standard search area at distances more than 200 m from turbine bases. We placed trial carcasses within the facilities, but away from turbines to avoid attracting avian scavengers to areas with turbines. We monitored the trial carcasses over a 63-day trial period during fall trials, checking the carcasses every day for the first four days, and then on days seven, 10, 14, 21, 28, 35, 42, 49, 56, and 63. This schedule reduces trial costs (relative to checking carcasses every day), while offering sufficient precision of the persistence estimate using the analysis method described below. NextEra provided carcass data collected from Montezuma II using similar protocols, but using a 28-day trial period. We recorded the location of persistence trial carcasses with a handheld Global Positioning System unit and discreetly marked carcasses with electrical tape to distinguish persistence trial carcasses from non-trial carcasses. Our criteria for determining whether carcasses were still “present” (available for detection) varied by trial; during 2013 and 2014 trials, we considered carcasses that were intact, scavenged, partial, or a feather spot (at least 10 feathers [or at least two primary feathers]) available for detection. During 2015 trials, the feather spot had to cover a 60 cm x 60 cm area (roughly the size of an intact carcass) for us to consider it available. We recorded the day on which the carcass was no longer present, or any remaining evidence of the carcass (e.g., portions of the carcass, feather spots) that remained during each search. We removed any carcasses or remaining evidence on the last day of trials.

### Analysis methods

Analytic methods included estimating: 1) searcher efficiency for the scanning searches, 2) carcass density distribution, 3) probability of carcass persistence, and 4) overall probability of detection. We summarize the analytic methods here and cover them in more detail in the supplemental information (S1 and S2 Appendices).

#### Searcher efficiency

We defined searcher efficiency as the probability that a searcher detects a carcass, given that the carcass is available to be detected. For our scanning search method, we estimated searcher efficiency based on carcass detection (i.e., the number of decoys found in relation to the number placed in the field), which we expected to be a function of distance from the searcher [30]. We performed scans from the turbine pads out to distances of 100 m or 150 m (see Table 1) and used logistic regression to estimate carcass detection as the log-odds that a decoy was detected. Covariates evaluated included distance from turbine, year, visibility class, facility, and region. We fit all possible combinations of covariates with interaction terms, but always retained the main effects involved in higher-order interactions. We used sample-size corrected Akaike’s Information Criterion (AICc) to rank the models [31]. We selected the most parsimonious model from among those within two AICc points of the top model (based on AICc rank). In the obstructed view areas, we estimated the decoy detection as the proportion of available decoys (i.e., not blown away by wind or removed by other means) detected during transects through the obstructed view visibility class.

We expected decoy detection to decrease with distance from the searcher, but the density of carcasses also changed with distance from the turbine (where the searcher stood). To correctly model searcher efficiency for the scanning search method, we combined carcass detection and the carcass density distribution within the search area (see *Carcass Density Distribution* below) to estimate searcher efficiency (see complete methods in S1 and S2 Appendices).

#### Carcass density distribution

As noted above, we expected non-uniform carcass density with respect to distance from turbines [18]. We modeled the distance distribution of carcasses relative to the turbines (hereafter, “density distribution”) both to estimate the fraction of carcasses that occurred within searched areas, and also to calculate searcher efficiency.

We detected no large avian carcasses in the current study, so we used data from several monitoring studies (Table B in S1 Appendix) to inform a density distribution model for large raptor carcasses. We included raptors that were found on both fully and partially searched plots, and we only included raptors found near turbines more than 78 m tall. We selected the height criterion as a compromise between making the distribution conservative (larger turbines may “throw” carcasses further from the turbine [32]), and obtaining enough carcass data to fit a distribution. The final dataset included 26 raptor carcasses found during 17 studies, and accounted for variable plot sizes and variable probabilities of detection in the different studies (listed in Table B in S1 Appendix).

The carcass distribution model assumed that raptors may fall as far as 175 m from turbines. We assumed that the density distribution followed one of six probability distributions (truncated normal, gamma, Weibull, log-logistic, Gompertz, or Rayleigh), which we fitted as weighted densities using maximum likelihood methods, and we used AICc to select the best model for the available data. We provide the complete methods in S1 and S2 Appendices.

#### Carcass persistence

We modeled carcass persistence as a function of bird type (raptor and game bird), visibility class, and the interaction between bird type and visibility. We estimated the average probability of persistence of a carcass using a survival model fitted to account for interval-censored data [33]. Exponential, log-logistic, lognormal, and Weibull distributions were fit using methods and with distribution parameterizations detailed in Dalthorp et al. [34], and we selected the best model using AICc. We fit models for JC, BH I, and BH II combined in 2013–2014, JC, BH I and BH II combined in 2015, and for Shiloh I (three models, total). We did not fit separate models for JC, BH I, and BH II because these facilities are adjacent to one another, and we assumed that the close spatial proximity resulted in similar carcass persistence characteristics between the three facilities.

#### Overall probability of detection

The overall probability of detection depends on the searcher efficiency, the fraction of carcasses that occur within searched areas, and the probability of carcass persistence. For this analysis, we also considered the possibility that carcasses missed once might be available for detection on subsequent searches. We assumed that a carcass missed at least once probably had a lower detectability than a carcass that had not been missed, possibly because it was in a less conspicuous location, because it was decaying between searches, or for other reasons. To account for detection that may change between searches, we included a *detection reduction factor* in our estimated probability of detection. The detection reduction factor can range from 1.0, which implies that a carcass has the same searcher efficiency over an infinite time frame, to 0, which implies that carcasses that are missed on the first search can never be found. We did not estimate the detection reduction factor for our analysis, but rather assumed a value of 0.67 [21, 35]. This assumption meant that carcass detectability was reduced by a factor of 0.67 with each successive search. We provide details in Table A in S1 Appendix.

## Results

### Bias trials

#### Searcher efficiency

We placed a total of 1,040 decoys during scanning searches to estimate searcher efficiency, of which searchers detected 790 (76%; Table 3). When stratified by study and visibility class, carcass detection ranged from 5% to 98% (Table 3).

**Table 3:** Searcher efficiency trial results as a function of year and visibility class at the Juniper Canyon (JC), Big Horn I (BH I), Big Horn II (BH II), and Shiloh I wind energy facilities. Overall, searchers detected 76% of all decoy placements.

Facility	Year	Visibility	Available	Found	Percent Found
**JC**	2013	Easy	87	82	94
**JC**	2013	Moderate	132	103	78
**BH I/BH II**	2014	Easy	98	96	98
**BH I/BH II**	2014	Moderate	193	143	74
**Shiloh I**	2015	Easy	244	236	97
**Shiloh I**	2015	Moderate	43	25	58
**JC**	2015	Easy	19	14	74
**JC**	2015	Moderate	21	7	33
**JC**	2015	Difficult	31	9	29
**JC**	2015	Obstructed view	24	13	54
**BH I/BH II**	2015	Easy	36	31	86
**BH I/BH II**	2015	Moderate	33	13	39
**BH I/BH II**	2015	Difficult	40	2	5
**BH I/BH II**	2015	Obstructed view	39	16	41

**Fig 5:**
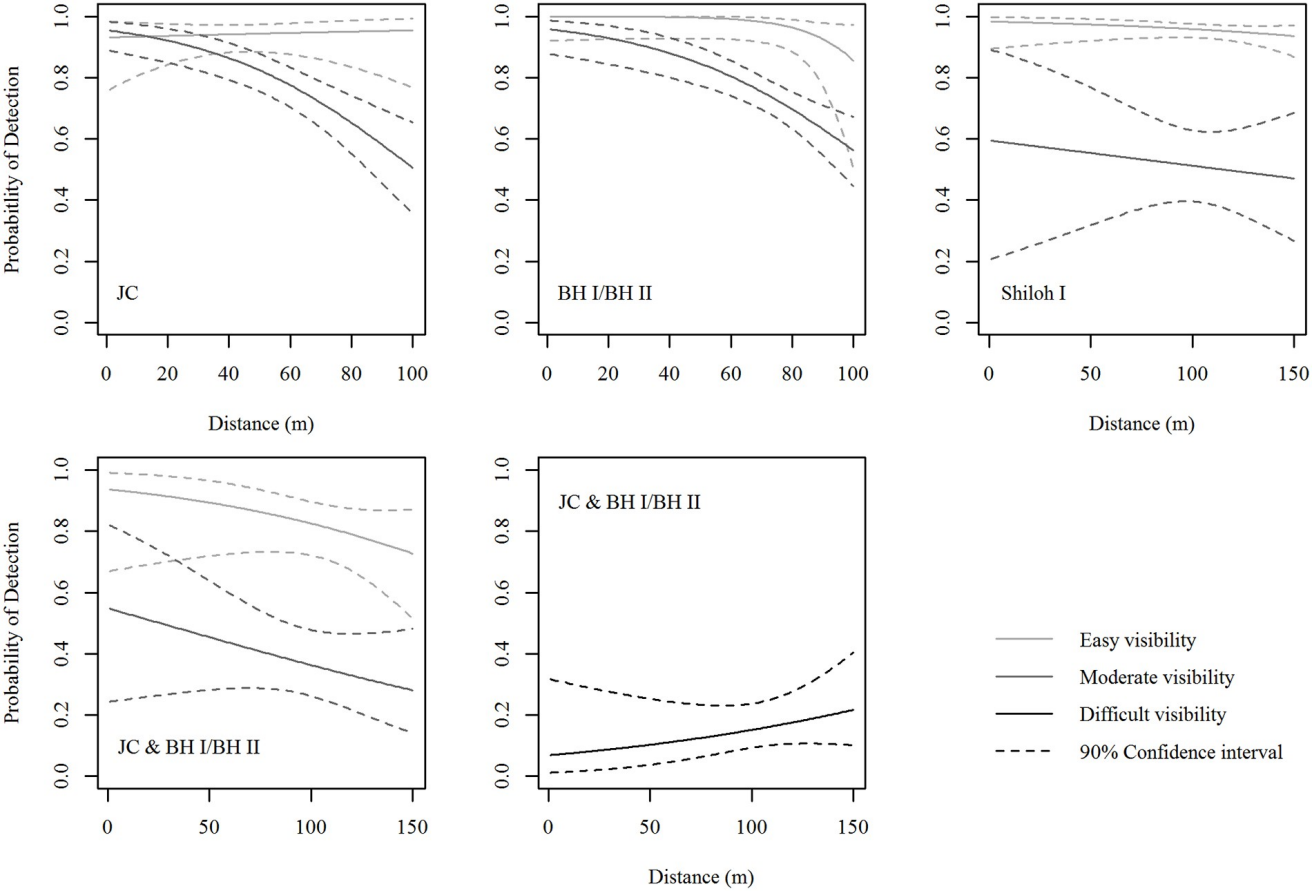
Decoy detection within visibility classes at the Juniper Canyon, Big Horn I/Big Horn II, and Shiloh I wind facilities.

Top models for decoy detection all included the main effects of distance, visibility, facility, and year, but the top model included four interaction terms, whereas the other two models within two AICc points included only three interaction terms (Table 4). We selected the third-ranked model because a distance by visibility interaction was more plausible than a distance by year interaction and because the information criterion differed little between the second- and third-ranked models (Table 4). Other interactions in the selected model were distance by facility and visibility by facility. Logistic regression models of decoy detection (Tables 4 and 5) indicated that at both JC in 2013 and BH I/BH II in 2014, searchers detected decoys consistently with respect to distance in the easy visibility class and decoy detection remained above 50% out to 100 m in the moderate visibility class (Fig 5). Searchers detected decoys consistently at Shiloh I with respect to distance in both easy and moderate visibility areas (Fig 5). At JC and BH I/BH II in 2015, decoy detection decreased gradually with respect to distance in the easy and moderate visibility areas, but increased with respect to distance in the difficult visibility areas (Fig 5).

**Table 4:** Searcher efficiency models with delta (Δ) sample-size Akaike’s information criterion (AICc) ≤2. Top models for decoy detection all included the main effects of distance, visibility, facility, and year. We selected the third-ranked model because a distance by visibility interaction was more plausible than a distance by year interaction and because the information criterion differed little between the second- and third-ranked models.

Model Form	AICc	ΔAICc
Distance + visibility + facility + year + (distance * visibility) + (distance * facility) + (visibility * facility) + (distance * year)	720.8	0
Distance + visibility + facility + year + (distance * facility) + (visibility * facility) + (distance * year)	722.5	1.7
Distance + visibility + facility + year + (distance * visibility) + (distance * facility) + (visibility * facility)	722.7	1.9^a^

^a^ We used this model in the analysis.

**Table 5:** Model parameters for logistic regression of searcher efficiency (response was the log-odds of a detection) at the Juniper Canyon (JC), Big Horn I (BH I), Big Horn II (BH II), and Shiloh I wind energy facilities. The reference parameter (i.e., the intercept) predicts detection probability at zero distance in difficult visibility during the first year at the Big Horn I (BH I) and Big Horn II (BH II) wind energy facilities. Note that this reference condition does not occur in the data because difficult visibility areas were not searched at the Big Horn I (BH I) and Big Horn II (BH II) wind energy facilities.

Parameter	Estimate	Standard Error
**(Intercept)**	-3.137	2.178
**Distance**^a^	0.005	0.018
**Easy visibility**^a^	8.367	2.031
**Moderate visibility**^a^	6.255	2.013
**JC**	2.575	1.319
**Shiloh I**	-2.724	1.043
**Second year at facility**	-2.594	0.812
Distance * easy visibility	-0.031	0.015
Distance * moderate visibility	-0.034	0.015
Distance * JC	0.001	0.008
Distance * Shiloh I	0.028	0.011
Easy visibility * JC	-3.645	1.094
Moderate visibility * JC	-2.867	1.022
Easy visibility * Shiloh I	0.624	0.662
Distance * second year at facility	0.018	0.009

^a^ Distance and visibility classes apply to all four facilities.

Searcher efficiency was greater than 0.70 at all studies, except at JC and BH I/BH II in 2015, when it was 0.63 and 0.50, respectively (Table 6).

**Table 6:** Searcher efficiencies^a^ for the different studies at the Juniper Canyon (JC), Big Horn I (BH I), Big Horn II (BH II), and Shiloh I wind energy facilities.

Facility	Year	Searcher Efficiency	Lower Confidence Bound	Upper Confidence Bound
**JC**	2013	0.80	0.66	0.87
**BH I/BH II**	2014	0.74	0.62	0.81
**Shiloh I**	2015	0.79	0.71	0.85
**JC**	2015	0.63	0.48	0.76
**BH I/BH II**	2015	0.50	0.34	0.65

^a^ Confidence bounds are for a 90% confidence interval.

#### Carcass density distribution

We tested several large bird carcass density distributions, and we used AICc to compare model fit. The log-logistic distribution provided the best fit to these data (Table 7).

**Table 7:** Tested carcass density distribution models with corrected Akaike’s information criterion (AICc) and delta (Δ) AICc, and fitted parameters.

Distribution	AICc	ΔAICc	Model Parameters
**Log-logistic**	-110.1	0	shape = 0.2839 scale = 3.8056
**Gamma**	-108.1	2.1	shape = 4.4558 rate = 0.0877
**Rayleigh**	-106.3	3.8	scale = 41.5200
**Truncated normal**	-101.3	8.8	location = 46.9607 scale = 29.2583
**Gompertz**	-98.3	11.9	scale = 0.0173 shape = 0.0087
**Weibull**	-71.5	38.6	shape = 0.0010 scale = 10.0556

**Fig 6:**
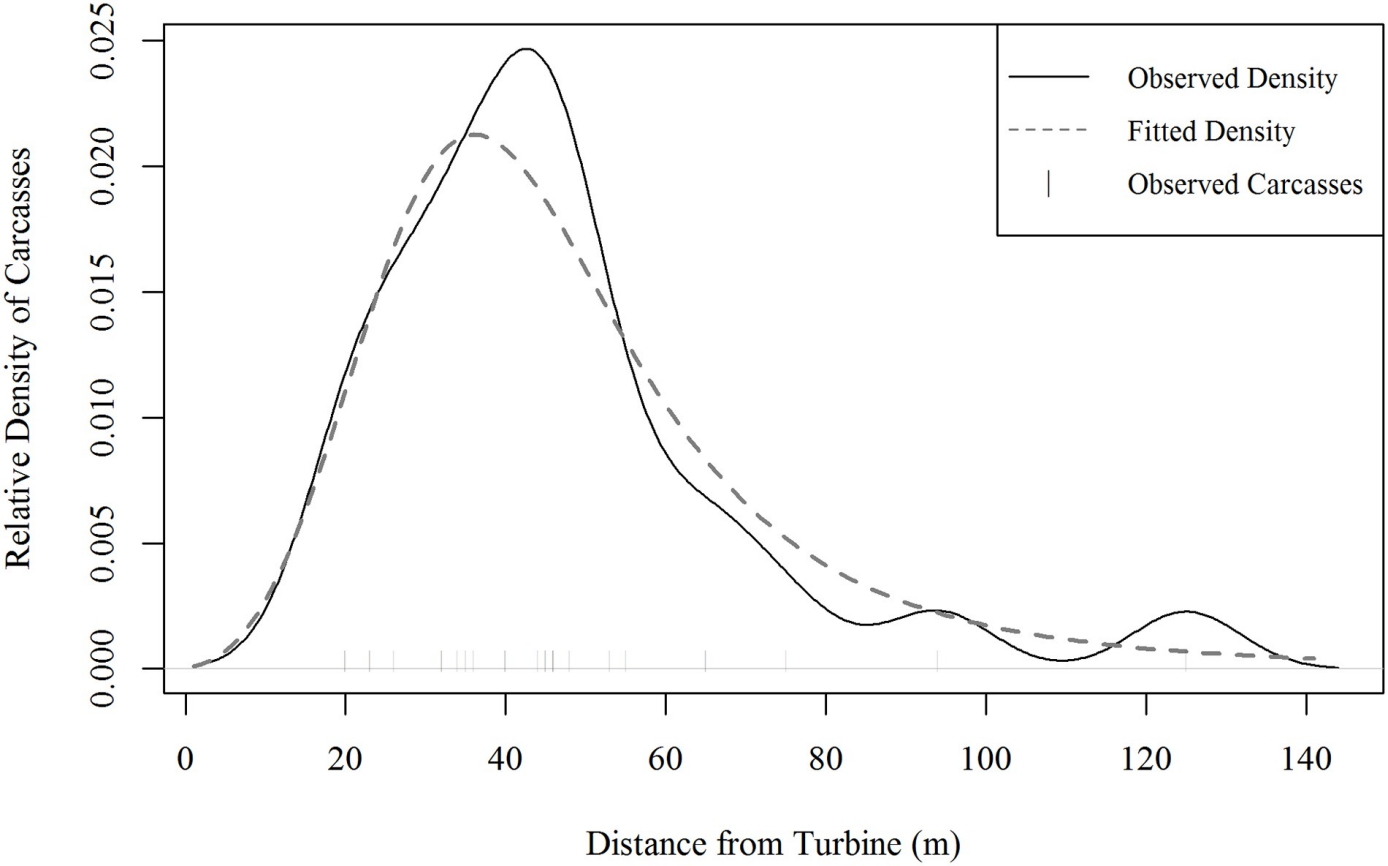
Fitted and observed density distribution of raptor carcasses.

The fitted log-logistic distribution predicted 87% of large bird carcasses land within 75 m of the turbine, 95% within 100 m of the turbine, and 99% within 150 m of the turbine (Fig 6). We expect the proportion of carcasses within searched areas to range from 83% to 87% when restricting search areas to easy and moderate visibility areas within 100 m of turbines, and to be over 99% when including all areas within 150 m of turbines in searched areas (Table 8).

**Table 8:** Proportion of large raptor carcasses expected within each visibility class^a^ at the Juniper Canyon (JC), Big Horn I (BH I), Big Horn II (BH II), and Shiloh I wind energy facilities.

Facility	Year	Visibility	Proportion of Carcasses	Lower Confidence Bound	Upper Confidence Bound
**JC**	2013	Easy	0.63	0.51	0.71
		Moderate	0.24	0.21	0.26
		All searched areas	0.88	0.74	0.93
**BH I/BH II**	2014	Easy	0.11	0.09	0.14
		Moderate	0.75	0.64	0.79
		All searched areas	0.86	0.74	0.90
**Shiloh I**	2015	Easy	0.79	0.72	0.83
		Moderate	0.05	0.04	0.05
		All searched areas	0.83	0.76	0.88
**JC and BH I/BH II**	2015	Easy	0.26	0.23	0.29
		Moderate	0.64	0.61	0.65
		Difficult	0.09	0.09	0.10
		Obstructed view	0.01	0.00	0.01
		All searched areas	0.99	0.97	1.00

^a^ Confidence bounds are for a 90% confidence interval.

#### Carcass persistence

**Fig 7:**
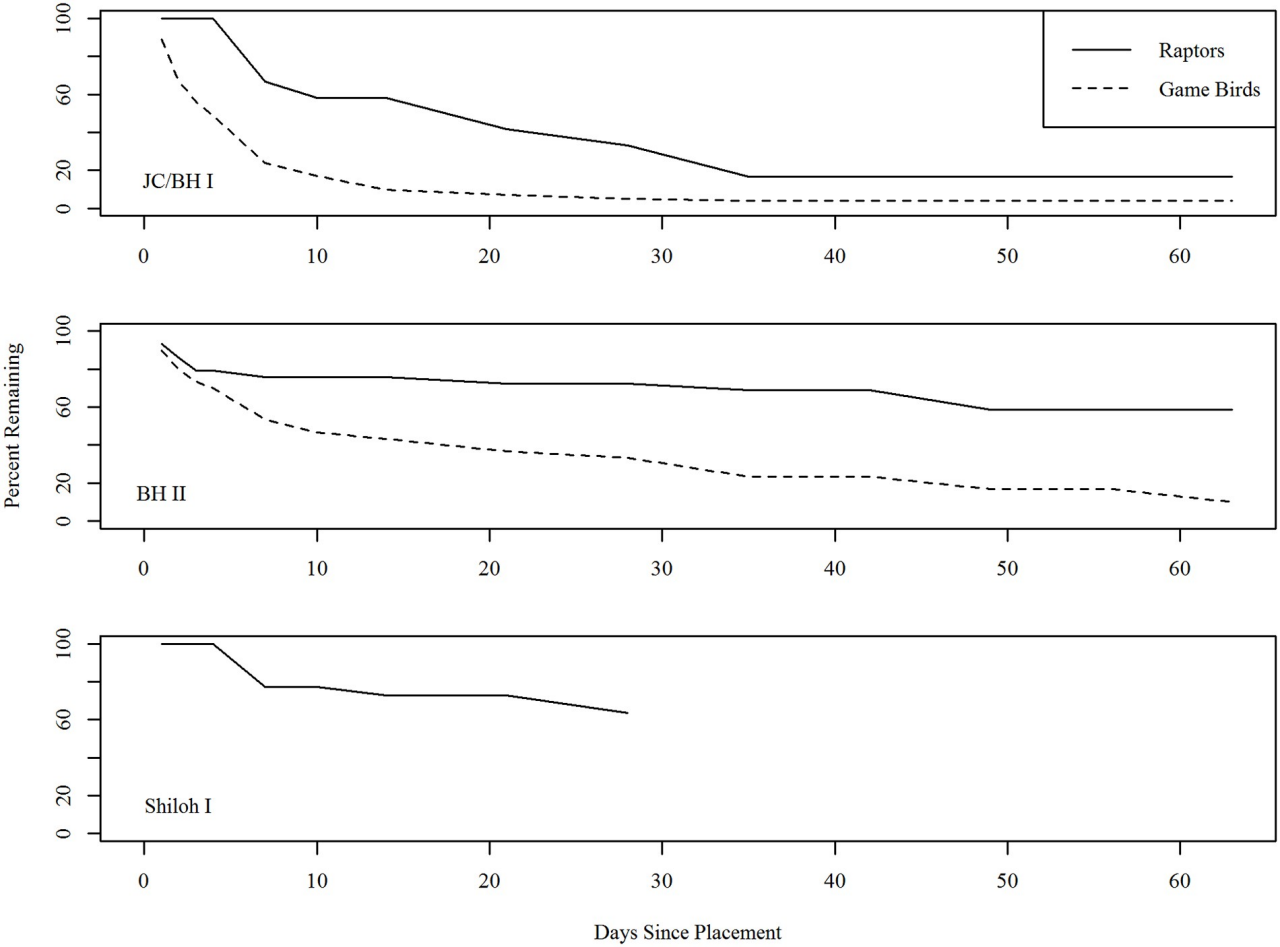
Empirical carcass persistence for raptors and game birds, by facility.

The best carcass persistence models at JC and BH I/BH II across years contained bird type (raptor or game bird) as the single explanatory variable (Table 9). For Shiloh I, we only utilized raptors in the model as the dataset did not include any game birds. Model distributions and variables varied by facility and bird type (Table 10). We found that all point estimates for raptor persistence probability were higher than all point estimates for game bird persistence probability, and where we tested game birds and raptors simultaneously, 90% confidence intervals did not overlap between the bird types. JC and BH I/BH II during 2013–2014 exhibited the lowest probability of persistence for both raptors and game birds, while Shiloh I exhibited the highest probability of persistence (Table 10, Fig 7).

**Table 9:** Carcass persistence models^a^ with corrected Akaike’s information criterion (AICc) and delta (Δ) AICc by facility and year for the Juniper Canyon (JC), Big Horn I (BH I), Big Horn II (BH II), and Shiloh I wind energy facilities.

Facility	Year	Distribution	Variables	AICc	ΔAICc
**JC and BH I/BH II**	2013–2014	Log-normal	Type	457.5	0
		Log-normal	Type + Visibility + (Type * Visibility)	458.1	0.6
		Exponential	Type + Visibility + (Type * Visibility)	459.2	1.7
**Shiloh I**	2015	Exponential	Intercept-only	73.7	0
		Log-normal	Intercept-only	74.7	1.0
		Log-logistic	Intercept-only	75.7	2.0
**JC and BH I/BH II**	2015	Log-normal	Type	457.5	0
		Log-normal	Type + Visibility + (Type * Visibility)	458.1	0.6

^a^ Only models with ΔAICc ≤2 are shown. The “Type” variable refers to bird type (i.e. raptor or game bird) and “Visibility” refers to visibility class.

**Table 10:** Carcass persistence distributions, model parameters and probabilities of persistence^a^ by facility, bird type, and search interval for the Juniper Canyon (JC), Big Horn I (BH I), Big Horn II (BH II), and Shiloh I wind energy facilities.

Facility	Year	Bird Type	Model Distribution	Location Parameter	Scale Parameter	Search Interval (days)	Probability of Persistence	Lower Confidence Bound	Upper Confidence Bound
**JC and BH I/BH II**	2013–2014	Raptor	Exponential	27.81	--	15	0.77	0.62	0.86
		Game bird	Lognormal	3.38	0.99		0.32	0.28	0.36
**JC and BH I/BH II**	2015	Raptor	Weibull	75.86	1.32	15	0.85	0.72	0.96
		Game bird	Lognormal	9.32	1.72		0.59	0.49	0.71
**Shiloh I**	2015	Raptor	Exponential	60.28	--	15	0.89	0.82	0.95
**JC and BH I/BH II**	2013–2014	Raptor	Exponential	27.81	--	30	0.61	0.42	0.75
		Game bird	Lognormal	3.38	0.99		0.18	0.15	0.21
**JC and BH I/BH II**	2015	Raptor	Weibull	75.86	1.32	30	0.76	0.62	0.90
		Game bird	Lognormal	9.32	1.72		0.45	0.34	0.55
**Shiloh I**	2015	Raptor	Exponential	60.28	--	30	0.79	0.68	0.89

^a^ Confidence bounds are for a 90% confidence interval.

### Overall probability of detection

We found the overall probability of detection was higher when we used raptor persistence probability than when we used game bird persistence probability, and shorter search intervals resulted in higher detection probabilities for all carcass types. We found that the overall probability that a carcass on the site would be detected, when we assumed 30-day search intervals, ranged from a low of 0.12 at BH I/BH II (using game birds for carcass persistence trials) to a high of 0.69 at Shiloh I (using raptors for carcass persistence trials; Table 11). When we assumed 15-day search intervals, we found the overall probability that a carcass would be detected increased to 0.22 at BH I/BH II (using game birds for carcass persistence trials; Table 11), and to 0.82 at Shiloh I (using raptors for carcass persistence trials; Table 11).

**Table 11:** Overall probability of detection^a^ by facility, bird type, and search interval for Juniper Canyon (JC), Big Horn I (BH I), Big Horn II (BH II), and Shiloh I.

Facility	Year	Bird Type	Search Interval (days)	Overall Probability of Detection	Lower Confidence Bound	Upper Confidence Bound
**JC**	2013	Game bird	15	0.23	0.18	0.28
**BH I/BH II**	2014	Game bird	15	0.22	0.16	0.26
**JC**	2015	Game bird	15	0.65	0.34	0.79
**BH I/BH II**	2015	Game bird	15	0.53	0.27	0.69
**JC**	2013	Game bird	30	0.13	0.09	0.16
**BH I/BH II**	2014	Game bird	30	0.12	0.08	0.15
**JC**	2015	Game bird	30	0.47	0.23	0.68
**BH I/BH II**	2015	Game bird	30	0.38	0.18	0.58
**JC**	2013	Raptor	15	0.77	0.50	0.78
**BH I/BH II**	2014	Raptor	15	0.70	0.47	0.75
**JC**	2015	Raptor	15	0.80	0.52	0.81
**BH I/BH II**	2015	Raptor	15	0.66	0.42	0.74
**Shiloh I**	2015	Raptor	15	0.82	0.70	0.87
**JC**	2013	Raptor	30	0.54	0.32	0.65
**BH I/BH II**	2014	Raptor	30	0.50	0.30	0.61
**JC**	2015	Raptor	30	0.63	0.42	0.71
**BH I/BH II**	2015	Raptor	30	0.52	0.33	0.63
**Shiloh I**	2015	Raptor	30	0.69	0.55	0.79

^a^ Confidence bounds are for a 90% confidence interval.

## Discussion

### Evaluating bias corrections

Practitioners can demonstrate eagle take permit compliance by using optimized search protocols that balance detection probability with monitoring effort and costs. An understanding of the bias corrections that influence the estimated overall probability of detection is useful when optimizing details of the scanning search method.

#### Searcher efficiency

The first factor that influences overall probability of detection is searcher efficiency. We found relatively high searcher efficiency in this study: 50–80%, with 76% of all decoys found. Although our sites were chosen because they had relatively open, flat landscapes, our searcher efficiency values are comparable with what has been found in other studies of large birds. Barrientos et al. [27] found a searcher efficiency rate of approximately 76% for eagle-sized birds (1000–4000 grams) in their review of 50 fatality monitoring studies that included wind and solar facilities, fences, communication towers, and power lines. Similarly, Bay et al. [36] found searcher efficiencies ranged from roughly 43% to 100% for large bird carcasses in 26 fatality monitoring studies at wind facilities, with a mean searcher efficiency of approximately 74%. These comparisons indicate comparable searcher efficiency results from studies employing transect and other search methods to that of our study using the scanning search method.

Carcass detection (as estimated by logistic regression) remained relatively constant regardless of distance from the turbine in the easy visibility class and decreased with distance in the moderate and difficult visibility classes (Tables 3 and 4, Fig 5). Although this distance effect on detection needs to be accounted for when using a scanning search method, the carcass distribution for large raptors indicates that over 75% of carcasses fall within 60 m from turbines. In other words, a large majority of large raptor carcasses will fall within areas where carcass detection remains relatively high (Fig 5). Therefore, the decrease in carcass detection when scanning areas further than 60 m from turbines has a smaller influence on the searcher efficiency and the overall probability of detection than a comparable loss of detection closer to the turbines would.

#### Carcass density distribution

Another factor influencing overall probability of detection is the carcass density distribution, which we used to estimate the proportion of carcasses falling within the searched areas and in the estimation of searcher efficiency for the scanning search method. Methods to fit carcass density distributions are an area of active research; although theoretical estimates for fall distances have been calculated [32], data for large raptor carcasses are scarce. Our analysis of existing data found that about 87% of large raptor carcasses fell within 75 m of turbines, 95% fell within 100 m of turbines, and 99% fell within 150 m of turbines (Fig 6; Table B in S1 and S2 Appendices). Our results show that the bias adjustment to account for carcasses falling outside of search areas was low across the range of search distances we tested.

#### Carcass persistence

We used carcass persistence rates to adjust carcass counts for removal bias. In our study, raptor carcasses persisted substantially longer than game bird carcasses regardless of season, year, or facility. Therefore, game birds do not accurately represent persistence of eagle carcasses because utilizing game bird persistence data would consistently underestimate carcass persistence of medium and large raptors (Table 10). This disparity results in much lower overall probabilities of detection using game birds (0.12 to 0.65) instead of raptors (0.50 to 0.82), even when modeling shorter search intervals (15 days) at facilities with higher searcher efficiency rates (Table 11). Our results for game bird persistence are consistent with a recent study in Scotland [29], which found an average probability of persisting through a 30-day search interval of 0.98 for common buzzard (*Buteo buteo*) carcasses and 0.26 for ring-necked pheasant carcasses. In addition to our finding that raptor carcasses persisted longer than game carcasses, we suggest substantially longer eagle carcass persistence than the persistence of other large raptors as well. A persistence study in Utah and Colorado resulted in estimated mean removal rates (percentage of carcasses removed by scavengers) of about 7% and 56% per month for eagles and other large raptors, respectively [37]. Assuming an exponential carcass persistence time, those rates translated to an average probability of persisting through a 30-day search interval of 0.97 for eagle carcasses and 0.76 for other large raptor carcasses. The above findings suggest that game bird persistence data are likely to be overly conservative when considering persistence of large raptor carcasses, such as eagles, and would result in inflated estimates if used in eagle fatality modeling.

### Using the overall probability of detection from the scanning search method to develop take estimates

Our research shows that an eagle carcass monitoring program using the scanning search method can be an efficient approach for gathering fatality monitoring data necessary to meet eagle take permit requirements. Here, we reported overall probability of detection estimated in the trials between 0.50 and 0.82 when we used raptors to inform carcass persistence (Table 11). One advantage of the scanning search method is that all facility turbines can be efficiently searched during each search round, which minimizes the bias adjustments and provides comprehensive spatial coverage. Maximum spatial coverage is desirable when monitoring for rare events (such as eagle fatalities) [38]. In standard transect searches following the guidance in Strickland et al. [16] and the land-based wind energy guidelines [17], the recommended proportion of turbines searched (30%) results in a maximum overall probability of detection of 0.30, substantially lower than the overall probabilities of detection in our study (Table 11). Even if searchers conduct transect searches at all turbines at a facility, the overall probability of detection might still be similar to those found in our study because carcass density distribution and carcass persistence are independent of search type, and searcher efficiency during our scanning searches (50–80%) is comparable to those found during transect-based searches (about 75%) [27, 36].

Researchers can use the overall probability of detection in two ways to evaluate if a facility is within their permitted eagle take or if adaptive management may be needed. First, researchers can use it as a metric to determine “how well” a facility was searched for eagle fatalities attributable to turbine collisions. Although detection probability by itself does not provide actionable information about permit compliance, researchers will find it is an intuitive measure of the efficacy of a monitoring program. Second, the researchers can use the overall probability of detection to adjust the number of eagles found during scanning searches to an estimated number of eagle fatalities. The US Geological Survey developed an estimator called Evidence of Absence, designed specifically to generate a fatality estimate for rare events [19, 34]. Estimating fatalities in a rare-event context (particularly when zero or few carcasses are found) is largely an exercise determining which fatality rates can be ruled out, and higher detection probabilities provide more power to constrain estimated fatality rates within a low range [39]. If we apply the Evidence of Absence estimator for a facility that found one eagle carcass during a monitoring year and we use estimated overall probabilities of detection for transect searches (0.30) and scanning searches (low = 0.50, high = 0.82), this results in 4, 2, and 1 estimated eagle fatalities, respectively (Table 12). Researchers’ ability to constrain estimated eagle fatality rates when detection rates are relatively high has practical implications with respect to demonstrating eagle take permit compliance, level of mitigation required, and likelihood for adaptive management actions if facilities exceed permitted take.

**Table 12:** Evidence of Absence results using a range of overall probability of detection estimates and carcass counts. Higher detection probabilities result in lower take estimates for a fixed carcass count.

Overall Probability of Detection	Eagle Carcasses Found	Eagle Carcasses Estimated
0.30	1	4
0.50	1	2
0.82	1	1
0.30	2	7
0.50	2	4
0.82	2	2
0.30	3	10
0.50	3	6
0.82	3	4

We have shown that the scanning search method produces quantifiable detection probability estimates that are high enough to constrain fatality estimates to one to two times the number of carcasses observed. Other benefits of the scanning search method include reduced monitoring costs. Although all fatality monitoring searches incur costs associated with travel time (both to the facility and within the facility), data collection, data management, and analyses, researchers will gain efficiency using scanning searches by spending less time searching for carcasses. For example, a typical search plot (240 m by 240 m) takes approximately 120 minutes to search using transects spaced 8 m apart (actual duration varies with terrain and ground cover). Researchers could search this same plot in less than 10 minutes using our scanning search method, reducing search effort by more than 90% per searched turbine. Assuming a 100-turbine facility, one round of transect searches at 30 turbines would require approximately 60 hours of search time; however, searchers could survey all 100 turbines in less than 17 hours using our scanning search method. Efficiency becomes even more substantial over 12 or more search rounds per year over multiple years of monitoring that will likely be required for eagle take permit compliance.

### Evaluating the appropriateness of the scanning search method at a wind facility

We believe our results show that scanning searches can be part of a viable eagle fatality monitoring protocol for eagle take permits, but facility operators should carefully evaluate each facility to ensure that overall probability of detection will meet their objectives. Assessing the potential for this monitoring protocol at a facility involves a few simple steps. First, we recommend viewshed mapping as a preliminary step in assessing the viability of a scan-based search protocol at a given facility. Areas that are not visible from turbine bases (i.e., obstructed view visibility class) may reduce detection to the point where searchers may need to include transect searches over parts of the search area. Then, researchers should assign visibility classes to all search areas visible from the turbine bases. Easy and moderate visibility areas close to turbine bases will produce the highest searcher efficiency, so our scanning search method may not be suitable for a facility containing a tall, thick scrub-shrub plant community within areas close to turbine bases. We suggest that researchers consider our carcass density distribution model (or an updated version, if available) when evaluating visibility classes and determining an appropriate search radius; if researchers find limited difficult or obstructed visibility classes, or these classes are generally far (>60 m) from turbine bases, transect searches may not be necessary to maintain minimal bias corrections. Researchers may also need to complete visibility mapping more than once if visibility conditions are changing due to seasonal vegetation changes.

Next, researchers should use raptor carcass persistence data to determine the appropriate search interval for a facility, as the overall probability of detection will increase by reducing the search interval. For example, the overall probability of detection at Shiloh I increased from 0.69 to 0.82 when the search interval was reduced from 30 days to 15 days (Table 11). A facility operator might choose to decrease their search interval if the eagle take permit threshold supports maximizing detection, despite the additional expense of more frequent searches.

It is worth noting that Avangrid Renewables’ operational staff conducted the majority of searches included in this study, and detection rates were consistent between Avangrid Renewables and WEST searchers. When practicable, integrating these searches into the regular maintenance visits performed by operations staff provides the highest cost-effectiveness, particularly when considering contractor mobilization costs for remote sites. Regardless of the approach taken, searchers should be thoroughly trained in carcass detection and reporting, as well as testing through field trials to determine true detection and persistence rates at the facility.

Facility operators must evaluate eagle take permit requirements, staff availability, and study objectives when determining whether operational staff or a contractor is best suited to perform the scanning searches. Furthermore, we do not expect the product of this work to replace standard post-construction monitoring protocols; rather, we expect the method to provide a cost-effective alternative when the monitoring objectives are focused on eagles or other large birds.

### Next steps

Fatality estimation of eagles is a developing science and multiple opportunities exist to refine our understanding. First, in our study we assumed that decoys are a reasonable surrogate to evaluate searcher efficiency for eagle carcasses. Ideally, researchers would test searcher efficiency using eagle carcasses. However, given the sensitivity regarding eagles, this type of testing is unlikely. In the absence of eagle carcasses, researchers may choose to evaluate decoys as a reasonable surrogate for testing detection rates of large raptor carcasses. Second, some fatality estimators (including Evidence of Absence) incorporate the detection reduction factor that describes how searcher efficiency changes between searches. In our study, we assumed a probability of 0.67 for this factor, consistent with Huso et al.’s [35] analysis of bat data from the northeastern US. We also considered partial carcasses and feather spots as available for detection; we acknowledge this as a source of bias for these trials, because partial carcasses and feather spots likely have different detection probabilities at 100 m compared to intact decoys used during the searcher efficiency trials. However, feather spots can often cover a much larger area than a decoy or an eagle carcass, resulting in an increased detection probability at this stage of decomposition. We do not know the factor by which eagle detection changes between searches or exactly where the “available for detection” line should be drawn for scanning searches, and suggest further studies to estimate these factors for the purpose of refining fatality estimates. Third, bias adjustments require carcass density distribution as an essential component; however, because we often find limited large bird fatalities per facility, we cannot estimate carcass density distributions from data at a single facility. Aside from this publication, compilations of these data across facilities do not exist and research should generate them. As additional data become available, analysis should determine how variations in site-specific (e.g., region, topography, land cover, wind direction, wind speed) or species-specific (e.g., size, weight) characteristics influence carcass density distribution. Finally, researchers need additional information with respect to raptor carcass persistence because game birds are not an appropriate surrogate for eagles. Studies would be most beneficial if they evaluated raptor carcass persistence across a diversity of regions to determine regionally appropriate persistence rates. Alternatively, researchers could evaluate game birds and raptors simultaneously to determine if a consistent index or scalar exists between game bird and raptor carcass persistence rates; researchers could then apply the index or scalar to site-specific carcass persistence rates. For either option, researchers should ensure appropriate state and federal permits before using raptor carcasses for bias trials.

## Supporting information

S1 Appendix Detailed methods of the analysis. (DOCX)

S2 Appendix Carcass density distribution dataset. (DOCX)
