# Supplementary material for: Developing an efficient protocol for monitoring eagle fatalities at wind energy facilities
Source: PLoS One. 2018 Dec 12;13(12):e0208700. doi: 10.1371/journal.pone.0208700 (PMC6291117; doi:10.1371/journal.pone.0208700)
Supplement: S1 Appendix — (DOCX) [file pone.0208700.s001.docx]

## Supplement 1. Detailed Methods of the Analysis

The effectiveness of the sampling protocol was evaluated based on the overall probability that a carcass will be detected (*g*), itself a function of searcher efficiency, carcass persistence, and carcass density distribution (Table A). This supplement details the methods used to estimate *g*.

Table A. Parameters and indices used in the calculation of *g,* which models they inform, and how they were obtained.

| **Parameter** | **Definition** | **How Obtained ^a^** | **Models in Which it is Used** |
| --- | --- | --- | --- |
| ***a_v,x_*** | Proportion of area in visibility class *v* between *x* – 1 m and *x* m from the turbine | Field mapping and geographic information system (GIS) from current and earlier studies | Searcher efficiency |
| ***b*** | Number of visibility classes | Field mapping and GIS from current and earlier studies | Searcher efficiency |
| ***c_i_*** | Fraction of carcasses arriving during the *i^th^* arrival interval | Assumed ^b^ (uniform over arrival intervals) | Overall probability of detection |
| ***d*** | Time (days) to carcass removal | Function input | Carcass persistence |
| ***f(d)*** | Probability distribution function for persistence times (*d*; days) of carcasses | Estimated | Carcass persistence |
| ***F(d)*** | Cumulative distribution function for persistence times (*d*; days) of carcasses | Estimated | Carcass persistence |
| ***g*** | Overall probability that a carcass arriving at the facility persists and is detected by searchers | Estimated | Overall probability of detection |
| ***g_i,j_*** | Probability that a carcass arriving during the *i^th^* interval persists until and is discovered during the *j^th^* search, conditional on having persisted until the *j – 1^th^* search | Estimated | Overall probability of detection |
| ***h*(*x\|*θ)** | Probability distribution function for distances (*x*; m) of carcasses from turbines | Estimated | Carcass density distribution |
| ***h^*^*(*x\|*θ)** | Weighted probability distribution function for distances (*x*; m) of carcasses from turbines | Estimated | Carcass density distribution |
| ***i*** | Index for carcass arrival intervals;  0 < *i ≤ j* *≤ q* | Index | Carcass persistence, overall probability of detection |
| ***I*** | Search interval; number of days between searches | Known | Carcass persistence |
| ***j*** | Index for carcass search events;  0 < *i ≤ j* *≤ q* | Index | Carcass persistence, overall probability of detection |
| ***k*** | Detection reduction factor; factor by which searcher efficiency (*s_v_* (*x*)) changes between searches | Assumed  (*k =* 0.67) | Overall probability of detection |
| **λ_z_** | Fatality rate (birds per turbine) at study *z* | Estimated in earlier studies | Carcass density distribution |
| ***n*** | Number of studies contributing data to the carcass density distribution (*h^*^*(*x* θ)) | Known | Carcass density distribution |
| ***p*** | Searcher efficiency; this is the probability that a carcass that is in a search area and available during a search is detected by a searcher; incorporates carcass detection, carcass distribution on the landscape, and the pattern of visibility on the landscape | Estimated | Overall probability of detection |
| **π_z_** | Overall probability of detection for a carcass at study *z* | Estimated in earlier studies | Carcass density distribution |
| ***q*** | Number of searches and search intervals during the study;  0 < *i ≤ j* *≤ q* | Known from field data | Overall probability of detection |
| ***r_i,j_*** | Average probability that a carcass arriving during interval *i* persists until search *j* | Estimated | Carcass persistence, overall probability of detection |
| ***s_v_*(*x*)** | Logistic regression function describing carcass detection as a function of distance (*x*; m) in visibility class *v* | Estimated | Carcass detection, searcher efficiency |
| **σ_z,x_** | Average proportion of area searched between *x* – 1 m and *x* m from the turbine at study *z* | GIS and field data from earlier studies | Carcass density distribution |
| ***t_z_*** | Number of turbines included in study *z* | Known from field data | Carcass density distribution |
| ***θ*** | Parameters associated with the probability distribution function for distances of carcasses from turbines *h*(*x*\|θ) | Estimated | Carcass density distribution |
| $\hat{\boldsymbol{\theta}}$ | Estimated parameters associated with the weighted probability distribution function for distances of carcasses from turbines  *h^*^*(*x*\|θ) | Estimated | Carcass density distribution |
| ***u*** | Maximum search distance (m) | Known from field data | Searcher efficiency |
| ***w*(*x*)** | Weighting function (of distance, *x*; m) used to fit the weighted distance distribution of carcasses from turbines (*h^*^*(*x*\|θ)) | Estimated | Carcass density distribution |
| ***x*** | Distance (m) of carcasses from turbines | Function input | Carcass density distribution, searcher efficiency |

^a^ We obtained “known” directly from field data; “assumed” values are in principle estimable but for the current study we used assumed values; methods for “estimated” values are given below.

^b^ This assumption is implicit in most studies estimating mortality from carcass search data.

^c^ This value of *k* is due to a an analysis of bats [1], but *k* has never been estimated for raptors. Estimating *k* is difficult because it requires a large number of carcasses to be placed in the field and tracked through multiple searches.

### Searcher Efficiency

Searcher efficiency (*p*) is the probability that a searcher discovers a carcass, given that the carcass is available to be discovered. For the scanning search method, searchers performed their scans from the turbine pads and searched distances out to 100 or 150 m, so carcass detection was expected to be a function of distance from the turbine. We used logistic regression to estimate carcass detection for the scanning search method. Potential covariates included distance from turbine, year, visibility, facility, and state (as a proxy for geographic region). We fit all possible combinations of covariates with interaction terms, but always retained the main effects involved in higher-order interactions. We used sample size corrected Akaike’s Information Criterion (AICc) to rank the models [2]. We selected the most parsimonious model from among those within two AICc points of the top model (based on AICc rank). In the obstructed view areas, we estimated carcass detection as the proportion of available decoys detected during meandering transects through the obstructed view visibility class.

Searcher efficiency for the scanning method is the density distribution-weighted carcass detection, and unlike the carcass detection function, is a single value. In the simplest case,

$p= \int_{0}^{u} s\left( x \right) \times h\left( x|\hat{\theta} \right)dx$ (1)

where $u$ is the maximum search radius, $h\left( x|\hat{\theta} \right)$ is the density distribution of carcasses with estimated parameter vector $\hat{\theta}$ (see *Density Distribution* section, below), and *s*(*x*) is the function describing carcass detection. Carcass detection is a logistic regression function for areas that were searched with a scanning search method, or a constant proportion of detected carcasses for areas that were searched with a transect search (i.e., the obstructed view areas; see *Field Methods* section in the main text). In practice, *s*(*x*) may be partitioned by visibility class, *s_v_*(*x*), with relative abundances of visibility classes calculated in 1-m annuli using GIS. It is simpler to calculate searcher efficiency as a sum of discrete values rather than of continuous functions,

$p= \sum_{v=1}^{b} \sum_{x=1}^{u} a_{v,x} \times\int_{x-1}^{x} s_{v}\left( y \right) \times h\left( y \right)dy$ (2)

where *b* is the number of visibility classes, *u* is the maximum search radius, and *a_v,x_* is the proportion of area in visibility class *v* between *x – 1* and *x* m from the turbine. The calculation can be further simplified by taking the searcher efficiency from *s_v_*(*y*) at a single distance, *x* within each 1-m wide band, rather than taking the average, as in the first integral above:

$p= \sum_{v=1}^{b} \sum_{x=1}^{u} a_{v,x} \times s_{v}\left( x \right) \times\int_{x-1}^{x} h\left( y \right)dy$ (3).

### Carcass Density Distribution

Carcass density is not uniform with respect to distance from turbines. It is necessary to model the distance distribution of carcasses relative to the turbines (hereafter, “carcass density distribution”) both to estimate the fraction of carcasses that occur within searched areas, and also to calculate searcher efficiency (*Searcher Efficiency* section, above).

We did not detect large avian carcasses during the current study, so we used data from several monitoring studies (Table B and S2 Appendix) to inform a carcass density distribution model for large raptor carcasses. We included raptors that found on both fully and partially searched plots, and we only included raptors found near turbines more than 78 m tall. The height criterion was selected as a compromise between making the distribution conservative (larger turbines are believed to ‘throw’ carcasses further from the turbine [3]), and obtaining enough carcass data to fit a distribution. Ten species of raptor were deemed suitable (large enough) for inclusion in the analysis, but only four of them occurred in our data set (Table C). The final dataset included 26 raptor carcasses.

Table B. Studies, facilities, and carcasses contributing data to the raptor carcass density distribution.

| **Facility name, state, and study year(s)** | **Species** | **Distance from turbine (m)** |
| --- | --- | --- |
| Alta VIII, CA (2012-2013) [4] | Ferruginous hawk | 125 |
| Alta Wind II-V, CA (2011) [5] | Red-tailed hawk | 65 |
| Alta Wind II-V, CA (2011) [5] | Red-tailed hawk | 45 |
| Barton Chapel, TX [6] | Red-tailed hawk | 20 |
| Big Horn, WA [7] | Red-tailed hawk | 35 |
| Big Horn, WA [7] | Ferruginous hawk | 46 |
| Biglow Canyon, OR (Phase I; 2008) [8] | Rough-legged hawk | 75 |
| Biglow Canyon, OR (Phase I; 2008) [8] | Red-tailed hawk | 48 |
| Biglow Canyon, OR (Phase III; 2010/2011) [9] | Red-tailed hawk | 32 |
| Buffalo Ridge I, SD (2010) [10] | Red-tailed hawk | 55 |
| Anonymous Wind Facility, MO | Red-tailed hawk | 44 |
| Anonymous Wind Facility, MO | Unknown buteo | 32 |
| Leaning Juniper, OR [11] | Swainson’s hawk | 94 |
| Leaning Juniper, OR [11] | Swainson’s hawk | 23 |
| Leaning Juniper, OR [11] | Ferruginous hawk | 65 |
| Prairie Winds SD1, SD (2013-2014) [12] | Red-tailed hawk | 53 |
| Red Hills, OK [13] | Red-tailed hawk | 40 |
| Rugby, ND [14] | Swainson’s hawk | 45 |
| Anonymous Wind Facility, CO | Swainson’s hawk | 20 |
| Juniper Canyon, OR (2011-2012) [15] | Red-tailed hawk | 34 |
| Juniper Canyon, OR (2011-2012) [15] | Red-tailed hawk | 23 |
| Juniper Canyon, OR (2011-2012) [15] | Red-tailed hawk | 26 |
| Anonymous Wind Facility, NH (2013) | Red-tailed hawk | 40 |
| Anonymous Wind Facility, NH (2015) | Red-tailed hawk | 46 |
| Anonymous Wind Facility, NH (2015) | Broad-winged hawk | 46 |
| Anonymous Wind Facility, OH (2015) | Red-tailed hawk | 36 |

Table C. Morphometric measurements [16] of raptor species included in carcass distribution analysis.

| **Common name** | **Length  (min-max, cm)** | **Weight  (min-max, g)** |
| --- | --- | --- |
| Swainson's hawk | 48-56 | 693-1397 |
| Red-tailed hawk | 45-56 | 900-1460 |
| Ferruginous hawk | 56-69 | 977-2074 |
| Rough-legged hawk | 47-52 | 715-1400 |

We assumed that the carcass density distribution followed one of six probability distributions (truncated normal, gamma, Weibull, log-logistic, Gompertz, or Rayleigh), and used AICc to select the best model for the available data. The raw observed distances of carcasses from turbines (hereafter, “observed density distribution”) do not represent the true underlying density distribution because the proportion of searchable area varied with distance from the turbine. Also, the carcass distance data came from studies that differed with respect to probability of detection within search areas and overall fatality rates, both of which can influence the observed distance distribution when plot sizes are not equal. We used a maximum likelihood estimation approach to fit a weighted distribution (D. Dalthorp, USGS, pers. comm.) to the data, where the weights reflect relative probabilities of detection, and relative fatality rates to account for the divergence between the observed and underlying density distributions.

If the underlying density distribution is described by some probability density function, *h*(*x*|θ), where *x* is distance from the turbine, θ is the associated parameter vector, and the weights are described by a function, *w*(*x*), then the weighted distribution is,

$h^{*}\left( x|\theta\right)= \frac{w\left( x \right) \times h\left( x|\theta\right)}{\int_{0}^{\infty} w\left( y \right) \times h\left( y|\theta\right)dy}$ (4)

where the *w*(*x*) in the numerator reflects the distortion of the underlying density distribution, *h*(*x*|θ), that is due to variable detection probability, and the integral in the denominator ensures that the weighted distribution is still a valid probability function. $\hat{\theta}$ is obtained by maximizing the likelihood associated with *h^*^*(*x*|θ), and the underlying density distribution is approximated as *h*(*x*|θ).

The weight function needs to include any factor that influences the probability to detect a carcass. Although some components of the weight function are not individually distance-dependent, they become so when combined with data across several studies with different search radii. The weight function is difficult to develop because most of its components need to be estimated. We approximated the weight function,

$w\left( x \right)= max\left( \varepsilon, \frac{\sum_{z=1}^{n} \pi_{z} \times\lambda_{z} \times\sigma_{z, x} \times t_{z}}{\sum_{z=1}^{n} t_{z}} \right)$, (5)

for distances from 0 ≤ *x ≤* 175 m, and assigned it a value of 0 for all other distances. In Equation 5, ε is a very small non-zero value (taken here to be one 1/1000^th^ of the smallest non-zero weight), *n* is the number of studies represented in the sample, $\pi_{z}$ is the detection probability for a carcass at study *z*, *λ_z_* is the large bird fatality rate at study *z,* *t_z_* is the number of turbines included in study *z*, and *σ_z,x_* is the average proportion of area searched in the *x^th^* annulus at facility *z.* Forcing *w*(*x*) > 0 for all *x* ≤ 175 m effectively “claims” that there was (perhaps negligible) search effort out to a distance of 175 m. Claiming this negligible search effort accounts for carcasses that may have been missed because they fell between the search plot boundary and 175 m from the turbine because *h^*^*(*x*|θ) includes non-zero carcass density to a distance of 175 m. The cut-off value of 175 m for the density distribution is 1.5 times the maximum fall distance predicted for large carcasses at large turbines [3].

Where whole plots were searched, *σ_i,x_* was assumed to be 1.0 for all distances out to the radius (circular plots) or half the side length (square plots) of the plots, and 0 between the searched plot and 175 m. Where plots were incompletely searched, *σ_i,x_* was calculated at each turbine for discrete, 1-m increments of *x* using GIS software, and averaged over turbines at each facility. Weights and their components for the weighted distribution fitted here are given as a table in the supplementary information (S2 Appendix). Note that although it is possible and sometimes informative to estimate the proportion of carcasses occurring within the search radius: ($\int_{0}^{u} h\left( x|\hat{\theta} \right)dx$where *u* is the maximum search radius and $h\left( x|\hat{\theta} \right)$ is the density distribution of carcasses with estimated parameter vector $\hat{\theta}$), the adjustment occurs implicitly in the estimation of searcher efficiency (Equations 1, 2 and 3).

### Carcass Persistence

Carcass persistence was modeled as a function of bird type (raptor or game bird), visibility class, and the interaction between bird type and visibility. The average probability of persistence of a carcass was estimated from an interval censored carcass persistence survival model [3]. Exponential, log-logistic, lognormal, and Weibull distributions were fit using methods and with distribution parameterizations detailed in Dalthorp et al. [17] and the best model was selected using AICc. We fit models for Juniper Canyon (JC), Big Horn (BH) I, and BH II combined in 2013-2014; JC, BH I and BH II combined in 2015; and for Shiloh I (three models, total). We did not fit separate models for JC, BH I, and BH II because these facilities are adjacent to one another. We assumed that the three facilities had similar carcass persistence characteristics due to the close spatial proximity.

For a search interval, *I*, Dalthorp et al.’s [17] approach produces an estimate of the average probability that a carcass arriving {0, 1, 2,…, *I*} days before the search will persist until the search, rather than be removed by biotic (e.g., scavenging or decomposition) or abiotic (e.g., burial by farm equipment) means. Assuming carcass persistence times follow a probability distribution, *f*(*d*) with a cumulative probability function, *F*(*d*), the probability of persistence until time *d* is 1 – *F*(*d*). If carcass arrival is uniform in time so that the probability of arrival is uniform between days 0 and *I* prior to a search, the average persistence probability for a carcass arriving in the first interval and persisting until the first search is

$r_{1, 1}= \frac{\int_{0}^{I} 1-F\left( d \right) dd}{I}$ (6)

A minor modification of this formula accommodates carcasses that may be missed on the first search and discovered on a subsequent search (the *j^th^* search). The average probability of persistence until the *j^th^* search for a carcass that arrived during the first interval and persisted until the *j – 1^th^* search:

$r_{1, j}= \frac{\int_{\left( j-1 \right) \times I}^{j \times I} 1-F\left( d \right) dd}{\int_{\left( j-2 \right) \times I}^{\left( j -1 \right) \times I} 1-F\left( d \right) dd}$ (7)

Where *j* ≥ 2.

### Overall Probability of Carcass Detection

The detection probability for any particular carcass depends on when it arrives at the facility because carcasses that arrive earlier during the study period have the potential to persist through more searches, and therefore have more opportunities to be discovered, than carcasses arriving later during the study period. We assume that there are *q* searches during the study period that occur on days {*d*_1_, *d*_2_, … *d_q_*}, and we assume there are no carcasses available when the study period begins on day *d_0_* = 0. The time interval *d_i-1_* to *d_i_* is the *i^th^* arrival interval, and the fraction of carcasses arriving during the *i^th^* arrival interval is *c_i_*, where,

$\sum_{i=1}^{q} c_{i}=1.0$ (8)

If all of the *c_i_* are equal, that implies the same relative arrival rate of carcasses during each search interval. This would be the case if, for example, the arrival phenology of carcasses is uniform in time and the search interval is constant between searches. The *c_i_* can be adjusted to reflect non-constant arrival phenology, non-constant search interval, or both, but for simplicity the *c_i_* are often assumed to be equal.

The probability that the carcasses arriving during the *i^th^* interval persist and are detected on the *j^th^* or subsequent searches (*j* ≥ *i*; *interval-specific detection probability*) is calculated recursively for each search from *i* to *q*. The probability that a carcass persists and is detected on the first search after arrival (i.e., for *j = i*) is,

*g_i,j_* = *r_i,j_* × *p* (9)

where *r_i,j_* is the probability of persistence (Equation 7) and *p*is the searcher efficiency (Equation 3). The probability that the carcass persists and is detected on the second or subsequent searches after arrival (i.e. for *j* > *i*) is,

$g_{i, q}=g_{i, j}+\sum_{\psi=i+1}^{q} \left( 1- g_{i,\psi- 1} \right) \times\left( r_{i,\psi}\times p \times k^{\psi-i} \right)$ (10)

where *g_i,j_* is the probability that a carcass arriving during the *i^th^* interval persists and is detected during the *j^th^* search and *k* is the detection reduction factor. For a study with a total of $q$ search intervals, *g_i,*_* can be calculated for any 0 < *i* ≤ * ≤ *q*, but in practice we are interested in the probability that a carcass arriving during the *i^th^* interval is detected at *some* point before the end of the study, i.e., *g_i,q_*.

For this study, *k* was assumed to be 0.67 [1]; *k* is a difficult parameter to estimate because it requires the placement and tracking of a large number of trial carcasses through multiple searches. Searcher efficiency may change from one search to the next because carcasses decay as they age and because searchers may discover all of the easy-to-see carcasses during earlier searches. The first element of the product in the summand from Equation 10 represents the probability that the carcass was missed during all previous searches and the second element of the product in the summand from Equation 10 represents the probability that the carcass is discovered during the *j^th^* search.

The overall probability of detection for a carcass is the arrival-fraction weighted average of the interval-specific arrival probabilities is,

$g = \sum_{i=1}^{q} g_{i, q}\times c_{i}$ (11)

### Variance Estimation

There are known variance estimators for many of the variables that inform *g*, but the variance of *g* is not known. Confidence intervals for *g*, and all other estimated quantities, were approximated by taking 1,000 bootstrap resamples [18] from all of the data, refitting all of the models, and recalculating 1,000 iterations of *g*. Bootstrap samples were stratified by covariates included in the models (searcher efficiency and carcass persistence models), and model selection was not repeated during the bootstrap process. We constructed 90% confidence intervals by taking the 5^th^ and 95^th^ quantiles from the bootstrap samples.

### References

1. Huso M, Dalthorp D, Korner-Nievergelt F. Statistical principles of post-construction fatality monitoring design. In: Perrow M. editor. Wildlife and wind farms, conflicts and solutions, Volume 2, Onshore: monitoring and mitigation. Exeter: Pelagic Publishing; 2017. Chapter 4.

2. Burnham KP, Anderson DR. Model selection and multimodel inference: a practical information-theoretic approach. 2nd ed. New York: Springer; 2002.

3. Hull CL, Muir S. 2010. Search areas for monitoring bird and bat carcasses at wind farms using a Monte-Carlo model. Australasian J of Environ Manage. 2016; 17: 77-87.

4. Chatfield A, Bay K. Post-construction studies for the Mustang Hills and Alta VIII wind energy facilities, Kern County, California. Final report for the first year of operation: July 2012 – October 2013; 2014.

5. Chatfield A, Sonnenberg M, Bay K. Avian and bat mortality monitoring at the Alta-Oak Creek Mojave project, Kern County, California. Final report for the first year of operation March 22, 2011 – June 15, 2012; 2012.

6. Western EcoSystems Technology, Inc. Post-construction fatality surveys for the Barton Chapel wind project: Iberdrola Renewables; 2011.

7. Kronner K, Gritski B, Downes S. Big Horn wind power project wildlife fatality monitoring study: 2006 – 2007; 2008.

8. Jeffrey JD, Bay K, Erickson WP, Sonneberg M, Baker J, Kesterke M, et al. Portland General Electric Biglow Canyon wind farm phase I post-construction avian and bat monitoring first annual report, Sherman County, Oregon: January 2008 - December 2008; 2009.

9. Enk T, Bay K, Sonnenberg M, Boehrs JR. Year 2 avian and bat monitoring report: Biglow Canyon wind farm phase II, Sherman County, Oregon. September 13, 2010 – September 15, 2011; 2012.

10. Derby C, Chodachek K, Bay K, Merrill A. Post-construction fatality survey for the Buffalo Ridge I wind project: May 2009 – May 2010. 2010.

11. Gritski R, Kronner K, Downes S. Leaning Juniper wind power project, 2006 – 2008: wildlife monitoring final report; 2008.

12. Derby C, Dahl A, DiDonato G. Post-construction fatality monitoring studies for the PrairieWinds SD1 Wind energy facility, South Dakota. Final report: March 2013 - February 2014; 2014.

13. Derby C, Iskali G, Kauffman M, Thorn T, Lyon T, Dahl A. Post-construction monitoring results, Red Hills wind farm, Roger Mills and Custer counties, Oklahoma. Final report: March 2012 – March 2013; 2013.

14. Derby C, Chodachek K, Bay K, Nomani S. Post-construction fatality surveys for the Rugby wind project: Iberdrola Renewables, Inc.: March 2010 – March 2011; 2011.

15. Enz T, Bay K. 2012. Post-construction fatality surveys for the Juniper Canyon Wind Project, May 2011 – May 2012; 2012.

16. All About Birds. Species profiles. Cornell Lab of Ornithology; 2016. Available from: http://www.allaboutbirds.org

17. Dalthorp D, Huso M, Dail D, Kenyon J. Evidence of absence software user guide. US Geological Survey Data Series 881. 2014. Available from: http://pubs.usgs.gov/ds/0881/pdf/ds881.pdf

18. Manly BFJ. Randomization, bootstrap, and Monte Carlo methods in biology. 2nd ed. London; Chapman and Hall; 1997.
