# Supplementary material for: Developing an efficient protocol for monitoring eagle fatalities at wind energy facilities
Source: PLoS One. 2018 Dec 12;13(12):e0208700. doi: 10.1371/journal.pone.0208700 (PMC6291117; doi:10.1371/journal.pone.0208700)
Supplement: S2 Appendix — (DOCX) [file pone.0208700.s002.docx]

## Supplement 2. Carcass density distribution dataset

**Table A. Components of the weights for the weighted distribution describing carcass distances from turbines.**

| **Study** | **Distance from Turbine (meters; outer edge of annulus)^a^** | **Detection Probability** | **Proportion of Area Searched** | **Number of Turbines (scaled)** | **Fatality Rate (scaled)** | **Final Weight** |
| --- | --- | --- | --- | --- | --- | --- |
| **Alta VIII (2012-2013) [1]** | 3 | 0.68785848 | 1 | 0.037792895 | 0.009852217 | 0.00025612 |
| **Alta VIII (2012-2013) [1]** | 4 | 0.68785848 | 1 | 0.037792895 | 0.009852217 | 0.00025612 |
| **Alta VIII (2012-2013) [1]** | 5 | 0.68785848 | 1 | 0.037792895 | 0.009852217 | 0.00025612 |
| **Alta VIII (2012-2013) [1]** | 6 | 0.68785848 | 1 | 0.037792895 | 0.009852217 | 0.00025612 |
| **Alta VIII (2012-2013) [1]** | 7 | 0.68785848 | 1 | 0.037792895 | 0.009852217 | 0.00025612 |
| **Alta VIII (2012-2013) [1]** | 8 | 0.68785848 | 1 | 0.037792895 | 0.009852217 | 0.00025612 |
| **Alta VIII (2012-2013) [1]** | 9 | 0.68785848 | 1 | 0.037792895 | 0.009852217 | 0.00025612 |
| **Alta VIII (2012-2013) [1]** | 10 | 0.68785848 | 1 | 0.037792895 | 0.009852217 | 0.00025612 |
| **Alta VIII (2012-2013) [1]** | 11 | 0.68785848 | 1 | 0.037792895 | 0.009852217 | 0.00025612 |
| **Alta VIII (2012-2013) [1]** | 12 | 0.68785848 | 1 | 0.037792895 | 0.009852217 | 0.00025612 |
| **Alta VIII (2012-2013) [1]** | 13 | 0.68785848 | 1 | 0.037792895 | 0.009852217 | 0.00025612 |
| **Alta VIII (2012-2013) [1]** | 14 | 0.68785848 | 1 | 0.037792895 | 0.009852217 | 0.00025612 |
| **Alta VIII (2012-2013) [1]** | 15 | 0.68785848 | 1 | 0.037792895 | 0.009852217 | 0.00025612 |
| **Alta VIII (2012-2013) [1]** | 16 | 0.68785848 | 1 | 0.037792895 | 0.009852217 | 0.00025612 |
| **Alta VIII (2012-2013) [1]** | 17 | 0.68785848 | 1 | 0.037792895 | 0.009852217 | 0.00025612 |
| **Alta VIII (2012-2013) [1]** | 18 | 0.68785848 | 1 | 0.037792895 | 0.009852217 | 0.00025612 |
| **Alta VIII (2012-2013) [1]** | 19 | 0.68785848 | 1 | 0.037792895 | 0.009852217 | 0.00025612 |
| **Alta VIII (2012-2013) [1]** | 20 | 0.68785848 | 1 | 0.037792895 | 0.009852217 | 0.00025612 |
| **Alta VIII (2012-2013) [1]** | 21 | 0.68785848 | 1 | 0.037792895 | 0.009852217 | 0.00025612 |
| **Alta VIII (2012-2013) [1]** | 22 | 0.68785848 | 1 | 0.037792895 | 0.009852217 | 0.00025612 |
| **Alta VIII (2012-2013) [1]** | 23 | 0.68785848 | 1 | 0.037792895 | 0.009852217 | 0.00025612 |
| **Alta VIII (2012-2013) [1]** | 24 | 0.68785848 | 1 | 0.037792895 | 0.009852217 | 0.00025612 |
| **Alta VIII (2012-2013) [1]** | 25 | 0.68785848 | 1 | 0.037792895 | 0.009852217 | 0.00025612 |
| **Alta VIII (2012-2013) [1]** | 26 | 0.68785848 | 1 | 0.037792895 | 0.009852217 | 0.00025612 |
| **Alta VIII (2012-2013) [1]** | 27 | 0.68785848 | 1 | 0.037792895 | 0.009852217 | 0.00025612 |
| **Alta VIII (2012-2013) [1]** | 28 | 0.68785848 | 1 | 0.037792895 | 0.009852217 | 0.00025612 |
| **Alta VIII (2012-2013) [1]** | 29 | 0.68785848 | 1 | 0.037792895 | 0.009852217 | 0.00025612 |
| **Alta VIII (2012-2013) [1]** | 30 | 0.68785848 | 1 | 0.037792895 | 0.009852217 | 0.00025612 |
| **Alta VIII (2012-2013) [1]** | 31 | 0.68785848 | 1 | 0.037792895 | 0.009852217 | 0.00025612 |
| **Alta VIII (2012-2013) [1]** | 32 | 0.68785848 | 1 | 0.037792895 | 0.009852217 | 0.00025612 |
| **Alta VIII (2012-2013) [1]** | 33 | 0.68785848 | 1 | 0.037792895 | 0.009852217 | 0.00025612 |
| **Alta VIII (2012-2013) [1]** | 34 | 0.68785848 | 1 | 0.037792895 | 0.009852217 | 0.00025612 |
| **Alta VIII (2012-2013) [1]** | 35 | 0.68785848 | 1 | 0.037792895 | 0.009852217 | 0.00025612 |
| **Alta VIII (2012-2013) [1]** | 36 | 0.68785848 | 1 | 0.037792895 | 0.009852217 | 0.00025612 |
| **Alta VIII (2012-2013) [1]** | 37 | 0.68785848 | 1 | 0.037792895 | 0.009852217 | 0.00025612 |
| **Alta VIII (2012-2013) [1]** | 38 | 0.68785848 | 1 | 0.037792895 | 0.009852217 | 0.00025612 |
| **Alta VIII (2012-2013) [1]** | 39 | 0.68785848 | 1 | 0.037792895 | 0.009852217 | 0.00025612 |
| **Alta VIII (2012-2013) [1]** | 40 | 0.68785848 | 1 | 0.037792895 | 0.009852217 | 0.00025612 |
| **Alta VIII (2012-2013) [1]** | 41 | 0.68785848 | 1 | 0.037792895 | 0.009852217 | 0.00025612 |
| **Alta VIII (2012-2013) [1]** | 42 | 0.68785848 | 1 | 0.037792895 | 0.009852217 | 0.00025612 |
| **Alta VIII (2012-2013) [1]** | 43 | 0.68785848 | 1 | 0.037792895 | 0.009852217 | 0.00025612 |
| **Alta VIII (2012-2013) [1]** | 44 | 0.68785848 | 1 | 0.037792895 | 0.009852217 | 0.00025612 |
| **Alta VIII (2012-2013) [1]** | 45 | 0.68785848 | 1 | 0.037792895 | 0.009852217 | 0.00025612 |
| **Alta VIII (2012-2013) [1]** | 46 | 0.68785848 | 1 | 0.037792895 | 0.009852217 | 0.00025612 |
| **Alta VIII (2012-2013) [1]** | 47 | 0.68785848 | 1 | 0.037792895 | 0.009852217 | 0.00025612 |
| **Alta VIII (2012-2013) [1]** | 48 | 0.68785848 | 1 | 0.037792895 | 0.009852217 | 0.00025612 |
| **Alta VIII (2012-2013) [1]** | 49 | 0.68785848 | 1 | 0.037792895 | 0.009852217 | 0.00025612 |
| **Alta VIII (2012-2013) [1]** | 50 | 0.68785848 | 1 | 0.037792895 | 0.009852217 | 0.00025612 |
| **Alta VIII (2012-2013) [1]** | 51 | 0.68785848 | 1 | 0.037792895 | 0.009852217 | 0.00025612 |
| **Alta VIII (2012-2013) [1]** | 52 | 0.68785848 | 1 | 0.037792895 | 0.009852217 | 0.00025612 |
| **Alta VIII (2012-2013) [1]** | 53 | 0.68785848 | 1 | 0.037792895 | 0.009852217 | 0.00025612 |
| **Alta VIII (2012-2013) [1]** | 54 | 0.68785848 | 1 | 0.037792895 | 0.009852217 | 0.00025612 |
| **Alta VIII (2012-2013) [1]** | 55 | 0.68785848 | 1 | 0.037792895 | 0.009852217 | 0.00025612 |
| **Alta VIII (2012-2013) [1]** | 56 | 0.68785848 | 1 | 0.037792895 | 0.009852217 | 0.00025612 |
| **Alta VIII (2012-2013) [1]** | 57 | 0.68785848 | 1 | 0.037792895 | 0.009852217 | 0.00025612 |
| **Alta VIII (2012-2013) [1]** | 58 | 0.68785848 | 1 | 0.037792895 | 0.009852217 | 0.00025612 |
| **Alta VIII (2012-2013) [1]** | 59 | 0.68785848 | 1 | 0.037792895 | 0.009852217 | 0.00025612 |
| **Alta VIII (2012-2013) [1]** | 60 | 0.68785848 | 1 | 0.037792895 | 0.009852217 | 0.00025612 |
| **Alta VIII (2012-2013) [1]** | 61 | 0.68785848 | 1 | 0.037792895 | 0.009852217 | 0.00025612 |
| **Alta VIII (2012-2013) [1]** | 62 | 0.68785848 | 1 | 0.037792895 | 0.009852217 | 0.00025612 |
| **Alta VIII (2012-2013) [1]** | 63 | 0.68785848 | 1 | 0.037792895 | 0.009852217 | 0.00025612 |
| **Alta VIII (2012-2013) [1]** | 64 | 0.68785848 | 1 | 0.037792895 | 0.009852217 | 0.00025612 |
| **Alta VIII (2012-2013) [1]** | 65 | 0.68785848 | 1 | 0.037792895 | 0.009852217 | 0.00025612 |
| **Alta VIII (2012-2013) [1]** | 66 | 0.68785848 | 1 | 0.037792895 | 0.009852217 | 0.00025612 |
| **Alta VIII (2012-2013) [1]** | 67 | 0.68785848 | 1 | 0.037792895 | 0.009852217 | 0.00025612 |
| **Alta VIII (2012-2013) [1]** | 68 | 0.68785848 | 1 | 0.037792895 | 0.009852217 | 0.00025612 |
| **Alta VIII (2012-2013) [1]** | 69 | 0.68785848 | 1 | 0.037792895 | 0.009852217 | 0.00025612 |
| **Alta VIII (2012-2013) [1]** | 70 | 0.68785848 | 1 | 0.037792895 | 0.009852217 | 0.00025612 |
| **Alta VIII (2012-2013) [1]** | 71 | 0.68785848 | 1 | 0.037792895 | 0.009852217 | 0.00025612 |
| **Alta VIII (2012-2013) [1]** | 72 | 0.68785848 | 1 | 0.037792895 | 0.009852217 | 0.00025612 |
| **Alta VIII (2012-2013) [1]** | 73 | 0.68785848 | 1 | 0.037792895 | 0.009852217 | 0.00025612 |
| **Alta VIII (2012-2013) [1]** | 74 | 0.68785848 | 1 | 0.037792895 | 0.009852217 | 0.00025612 |
| **Alta VIII (2012-2013) [1]** | 75 | 0.68785848 | 1 | 0.037792895 | 0.009852217 | 0.00025612 |
| **Alta VIII (2012-2013) [1]** | 76 | 0.68785848 | 1 | 0.037792895 | 0.009852217 | 0.00025612 |
| **Alta VIII (2012-2013) [1]** | 77 | 0.68785848 | 1 | 0.037792895 | 0.009852217 | 0.00025612 |
| **Alta VIII (2012-2013) [1]** | 78 | 0.68785848 | 1 | 0.037792895 | 0.009852217 | 0.00025612 |
| **Alta VIII (2012-2013) [1]** | 79 | 0.68785848 | 1 | 0.037792895 | 0.009852217 | 0.00025612 |
| **Alta VIII (2012-2013) [1]** | 80 | 0.68785848 | 1 | 0.037792895 | 0.009852217 | 0.00025612 |
| **Alta VIII (2012-2013) [1]** | 81 | 0.68785848 | 1 | 0.037792895 | 0.009852217 | 0.00025612 |
| **Alta VIII (2012-2013) [1]** | 82 | 0.68785848 | 1 | 0.037792895 | 0.009852217 | 0.00025612 |
| **Alta VIII (2012-2013) [1]** | 83 | 0.68785848 | 1 | 0.037792895 | 0.009852217 | 0.00025612 |
| **Alta VIII (2012-2013) [1]** | 84 | 0.68785848 | 1 | 0.037792895 | 0.009852217 | 0.00025612 |
| **Alta VIII (2012-2013) [1]** | 85 | 0.68785848 | 1 | 0.037792895 | 0.009852217 | 0.00025612 |
| **Alta VIII (2012-2013) [1]** | 86 | 0.68785848 | 1 | 0.037792895 | 0.009852217 | 0.00025612 |
| **Alta VIII (2012-2013) [1]** | 87 | 0.68785848 | 1 | 0.037792895 | 0.009852217 | 0.00025612 |
| **Alta VIII (2012-2013) [1]** | 88 | 0.68785848 | 1 | 0.037792895 | 0.009852217 | 0.00025612 |
| **Alta VIII (2012-2013) [1]** | 89 | 0.68785848 | 1 | 0.037792895 | 0.009852217 | 0.00025612 |
| **Alta VIII (2012-2013) [1]** | 90 | 0.68785848 | 1 | 0.037792895 | 0.009852217 | 0.00025612 |
| **Alta VIII (2012-2013) [1]** | 91 | 0.68785848 | 1 | 0.037792895 | 0.009852217 | 0.00025612 |
| **Alta VIII (2012-2013) [1]** | 92 | 0.68785848 | 1 | 0.037792895 | 0.009852217 | 0.00025612 |
| **Alta VIII (2012-2013) [1]** | 93 | 0.68785848 | 1 | 0.037792895 | 0.009852217 | 0.00025612 |
| **Alta VIII (2012-2013) [1]** | 94 | 0.68785848 | 1 | 0.037792895 | 0.009852217 | 0.00025612 |
| **Alta VIII (2012-2013) [1]** | 95 | 0.68785848 | 1 | 0.037792895 | 0.009852217 | 0.00025612 |
| **Alta VIII (2012-2013) [1]** | 96 | 0.68785848 | 1 | 0.037792895 | 0.009852217 | 0.00025612 |
| **Alta VIII (2012-2013) [1]** | 97 | 0.68785848 | 1 | 0.037792895 | 0.009852217 | 0.00025612 |
| **Alta VIII (2012-2013) [1]** | 98 | 0.68785848 | 1 | 0.037792895 | 0.009852217 | 0.00025612 |
| **Alta VIII (2012-2013) [1]** | 99 | 0.68785848 | 1 | 0.037792895 | 0.009852217 | 0.00025612 |
| **Alta VIII (2012-2013) [1]** | 100 | 0.68785848 | 1 | 0.037792895 | 0.009852217 | 0.00025612 |
| **Alta VIII (2012-2013) [1]** | 101 | 0.68785848 | 1 | 0.037792895 | 0.009852217 | 0.00025612 |
| **Alta VIII (2012-2013) [1]** | 102 | 0.68785848 | 1 | 0.037792895 | 0.009852217 | 0.00025612 |
| **Alta VIII (2012-2013) [1]** | 103 | 0.68785848 | 1 | 0.037792895 | 0.009852217 | 0.00025612 |
| **Alta VIII (2012-2013) [1]** | 104 | 0.68785848 | 1 | 0.037792895 | 0.009852217 | 0.00025612 |
| **Alta VIII (2012-2013) [1]** | 105 | 0.68785848 | 1 | 0.037792895 | 0.009852217 | 0.00025612 |
| **Alta VIII (2012-2013) [1]** | 106 | 0.68785848 | 1 | 0.037792895 | 0.009852217 | 0.00025612 |
| **Alta VIII (2012-2013) [1]** | 107 | 0.68785848 | 1 | 0.037792895 | 0.009852217 | 0.00025612 |
| **Alta VIII (2012-2013) [1]** | 108 | 0.68785848 | 1 | 0.037792895 | 0.009852217 | 0.00025612 |
| **Alta VIII (2012-2013) [1]** | 109 | 0.68785848 | 1 | 0.037792895 | 0.009852217 | 0.00025612 |
| **Alta VIII (2012-2013) [1]** | 110 | 0.68785848 | 1 | 0.037792895 | 0.009852217 | 0.00025612 |
| **Alta VIII (2012-2013) [1]** | 111 | 0.68785848 | 1 | 0.037792895 | 0.009852217 | 0.00025612 |
| **Alta VIII (2012-2013) [1]** | 112 | 0.68785848 | 1 | 0.037792895 | 0.009852217 | 0.00025612 |
| **Alta VIII (2012-2013) [1]** | 113 | 0.68785848 | 1 | 0.037792895 | 0.009852217 | 0.00025612 |
| **Alta VIII (2012-2013) [1]** | 114 | 0.68785848 | 1 | 0.037792895 | 0.009852217 | 0.00025612 |
| **Alta VIII (2012-2013) [1]** | 115 | 0.68785848 | 1 | 0.037792895 | 0.009852217 | 0.00025612 |
| **Alta VIII (2012-2013) [1]** | 116 | 0.68785848 | 1 | 0.037792895 | 0.009852217 | 0.00025612 |
| **Alta VIII (2012-2013) [1]** | 117 | 0.68785848 | 1 | 0.037792895 | 0.009852217 | 0.00025612 |
| **Alta VIII (2012-2013) [1]** | 118 | 0.68785848 | 1 | 0.037792895 | 0.009852217 | 0.00025612 |
| **Alta VIII (2012-2013) [1]** | 119 | 0.68785848 | 1 | 0.037792895 | 0.009852217 | 0.00025612 |
| **Alta VIII (2012-2013) [1]** | 120 | 0.68785848 | 1 | 0.037792895 | 0.009852217 | 0.00025612 |
| **Alta VIII (2012-2013) [1]** | 121 | 0.68785848 | 1 | 0.037792895 | 0.009852217 | 0.00025612 |
| **Alta VIII (2012-2013) [1]** | 122 | 0.68785848 | 1 | 0.037792895 | 0.009852217 | 0.00025612 |
| **Alta VIII (2012-2013) [1]** | 123 | 0.68785848 | 0 | 0.037792895 | 0.009852217 | 6.64E-10 |
| **Alta VIII (2012-2013) [1]** | 124 | 0.68785848 | 0 | 0.037792895 | 0.009852217 | 6.64E-10 |
| **Alta VIII (2012-2013) [1]** | 125 | 0.68785848 | 0 | 0.037792895 | 0.009852217 | 6.64E-10 |
| **Alta VIII (2012-2013) [1]** | 126 | 0.68785848 | 0 | 0.037792895 | 0.009852217 | 6.64E-10 |
| **Alta VIII (2012-2013) [1]** | 127 | 0.68785848 | 0 | 0.037792895 | 0.009852217 | 6.64E-10 |
| **Alta VIII (2012-2013) [1]** | 128 | 0.68785848 | 0 | 0.037792895 | 0.009852217 | 6.64E-10 |
| **Alta VIII (2012-2013) [1]** | 129 | 0.68785848 | 0 | 0.037792895 | 0.009852217 | 6.64E-10 |
| **Alta VIII (2012-2013) [1]** | 130 | 0.68785848 | 0 | 0.037792895 | 0.009852217 | 6.64E-10 |
| **Alta VIII (2012-2013) [1]** | 131 | 0.68785848 | 0 | 0.037792895 | 0.009852217 | 6.64E-10 |
| **Alta VIII (2012-2013) [1]** | 132 | 0.68785848 | 0 | 0.037792895 | 0.009852217 | 6.64E-10 |
| **Alta VIII (2012-2013) [1]** | 133 | 0.68785848 | 0 | 0.037792895 | 0.009852217 | 6.64E-10 |
| **Alta VIII (2012-2013) [1]** | 134 | 0.68785848 | 0 | 0.037792895 | 0.009852217 | 6.64E-10 |
| **Alta VIII (2012-2013) [1]** | 135 | 0.68785848 | 0 | 0.037792895 | 0.009852217 | 6.64E-10 |
| **Alta VIII (2012-2013) [1]** | 136 | 0.68785848 | 0 | 0.037792895 | 0.009852217 | 6.64E-10 |
| **Alta VIII (2012-2013) [1]** | 137 | 0.68785848 | 0 | 0.037792895 | 0.009852217 | 6.64E-10 |
| **Alta VIII (2012-2013) [1]** | 138 | 0.68785848 | 0 | 0.037792895 | 0.009852217 | 6.64E-10 |
| **Alta VIII (2012-2013) [1]** | 139 | 0.68785848 | 0 | 0.037792895 | 0.009852217 | 6.64E-10 |
| **Alta VIII (2012-2013) [1]** | 140 | 0.68785848 | 0 | 0.037792895 | 0.009852217 | 6.64E-10 |
| **Alta VIII (2012-2013) [1]** | 141 | 0.68785848 | 0 | 0.037792895 | 0.009852217 | 6.64E-10 |
| **Alta VIII (2012-2013) [1]** | 142 | 0.68785848 | 0 | 0.037792895 | 0.009852217 | 6.64E-10 |
| **Alta VIII (2012-2013) [1]** | 143 | 0.68785848 | 0 | 0.037792895 | 0.009852217 | 6.64E-10 |
| **Alta VIII (2012-2013) [1]** | 144 | 0.68785848 | 0 | 0.037792895 | 0.009852217 | 6.64E-10 |
| **Alta Wind II-V (2011) [2]** | 3 | 0.49823985 | 1 | 0.143613001 | 0.024630542 | 0.001762407 |
| **Alta Wind II-V (2011) [2]** | 4 | 0.49823985 | 1 | 0.143613001 | 0.024630542 | 0.001762407 |
| **Alta Wind II-V (2011) [2]** | 5 | 0.49823985 | 1 | 0.143613001 | 0.024630542 | 0.001762407 |
| **Alta Wind II-V (2011) [2]** | 6 | 0.49823985 | 1 | 0.143613001 | 0.024630542 | 0.001762407 |
| **Alta Wind II-V (2011) [2]** | 7 | 0.49823985 | 1 | 0.143613001 | 0.024630542 | 0.001762407 |
| **Alta Wind II-V (2011) [2]** | 8 | 0.49823985 | 1 | 0.143613001 | 0.024630542 | 0.001762407 |
| **Alta Wind II-V (2011) [2]** | 9 | 0.49823985 | 1 | 0.143613001 | 0.024630542 | 0.001762407 |
| **Alta Wind II-V (2011) [2]** | 10 | 0.49823985 | 1 | 0.143613001 | 0.024630542 | 0.001762407 |
| **Alta Wind II-V (2011) [2]** | 11 | 0.49823985 | 1 | 0.143613001 | 0.024630542 | 0.001762407 |
| **Alta Wind II-V (2011) [2]** | 12 | 0.49823985 | 1 | 0.143613001 | 0.024630542 | 0.001762407 |
| **Alta Wind II-V (2011) [2]** | 13 | 0.49823985 | 1 | 0.143613001 | 0.024630542 | 0.001762407 |
| **Alta Wind II-V (2011) [2]** | 14 | 0.49823985 | 1 | 0.143613001 | 0.024630542 | 0.001762407 |
| **Alta Wind II-V (2011) [2]** | 15 | 0.49823985 | 1 | 0.143613001 | 0.024630542 | 0.001762407 |
| **Alta Wind II-V (2011) [2]** | 16 | 0.49823985 | 1 | 0.143613001 | 0.024630542 | 0.001762407 |
| **Alta Wind II-V (2011) [2]** | 17 | 0.49823985 | 1 | 0.143613001 | 0.024630542 | 0.001762407 |
| **Alta Wind II-V (2011) [2]** | 18 | 0.49823985 | 1 | 0.143613001 | 0.024630542 | 0.001762407 |
| **Alta Wind II-V (2011) [2]** | 19 | 0.49823985 | 1 | 0.143613001 | 0.024630542 | 0.001762407 |
| **Alta Wind II-V (2011) [2]** | 20 | 0.49823985 | 1 | 0.143613001 | 0.024630542 | 0.001762407 |
| **Alta Wind II-V (2011) [2]** | 21 | 0.49823985 | 1 | 0.143613001 | 0.024630542 | 0.001762407 |
| **Alta Wind II-V (2011) [2]** | 22 | 0.49823985 | 1 | 0.143613001 | 0.024630542 | 0.001762407 |
| **Alta Wind II-V (2011) [2]** | 23 | 0.49823985 | 1 | 0.143613001 | 0.024630542 | 0.001762407 |
| **Alta Wind II-V (2011) [2]** | 24 | 0.49823985 | 1 | 0.143613001 | 0.024630542 | 0.001762407 |
| **Alta Wind II-V (2011) [2]** | 25 | 0.49823985 | 1 | 0.143613001 | 0.024630542 | 0.001762407 |
| **Alta Wind II-V (2011) [2]** | 26 | 0.49823985 | 1 | 0.143613001 | 0.024630542 | 0.001762407 |
| **Alta Wind II-V (2011) [2]** | 27 | 0.49823985 | 1 | 0.143613001 | 0.024630542 | 0.001762407 |
| **Alta Wind II-V (2011) [2]** | 28 | 0.49823985 | 1 | 0.143613001 | 0.024630542 | 0.001762407 |
| **Alta Wind II-V (2011) [2]** | 29 | 0.49823985 | 1 | 0.143613001 | 0.024630542 | 0.001762407 |
| **Alta Wind II-V (2011) [2]** | 30 | 0.49823985 | 1 | 0.143613001 | 0.024630542 | 0.001762407 |
| **Alta Wind II-V (2011) [2]** | 31 | 0.49823985 | 1 | 0.143613001 | 0.024630542 | 0.001762407 |
| **Alta Wind II-V (2011) [2]** | 32 | 0.49823985 | 1 | 0.143613001 | 0.024630542 | 0.001762407 |
| **Alta Wind II-V (2011) [2]** | 33 | 0.49823985 | 1 | 0.143613001 | 0.024630542 | 0.001762407 |
| **Alta Wind II-V (2011) [2]** | 34 | 0.49823985 | 1 | 0.143613001 | 0.024630542 | 0.001762407 |
| **Alta Wind II-V (2011) [2]** | 35 | 0.49823985 | 1 | 0.143613001 | 0.024630542 | 0.001762407 |
| **Alta Wind II-V (2011) [2]** | 36 | 0.49823985 | 1 | 0.143613001 | 0.024630542 | 0.001762407 |
| **Alta Wind II-V (2011) [2]** | 37 | 0.49823985 | 1 | 0.143613001 | 0.024630542 | 0.001762407 |
| **Alta Wind II-V (2011) [2]** | 38 | 0.49823985 | 1 | 0.143613001 | 0.024630542 | 0.001762407 |
| **Alta Wind II-V (2011) [2]** | 39 | 0.49823985 | 1 | 0.143613001 | 0.024630542 | 0.001762407 |
| **Alta Wind II-V (2011) [2]** | 40 | 0.49823985 | 1 | 0.143613001 | 0.024630542 | 0.001762407 |
| **Alta Wind II-V (2011) [2]** | 41 | 0.49823985 | 1 | 0.143613001 | 0.024630542 | 0.001762407 |
| **Alta Wind II-V (2011) [2]** | 42 | 0.49823985 | 1 | 0.143613001 | 0.024630542 | 0.001762407 |
| **Alta Wind II-V (2011) [2]** | 43 | 0.49823985 | 1 | 0.143613001 | 0.024630542 | 0.001762407 |
| **Alta Wind II-V (2011) [2]** | 44 | 0.49823985 | 1 | 0.143613001 | 0.024630542 | 0.001762407 |
| **Alta Wind II-V (2011) [2]** | 45 | 0.49823985 | 1 | 0.143613001 | 0.024630542 | 0.001762407 |
| **Alta Wind II-V (2011) [2]** | 46 | 0.49823985 | 1 | 0.143613001 | 0.024630542 | 0.001762407 |
| **Alta Wind II-V (2011) [2]** | 47 | 0.49823985 | 1 | 0.143613001 | 0.024630542 | 0.001762407 |
| **Alta Wind II-V (2011) [2]** | 48 | 0.49823985 | 1 | 0.143613001 | 0.024630542 | 0.001762407 |
| **Alta Wind II-V (2011) [2]** | 49 | 0.49823985 | 1 | 0.143613001 | 0.024630542 | 0.001762407 |
| **Alta Wind II-V (2011) [2]** | 50 | 0.49823985 | 1 | 0.143613001 | 0.024630542 | 0.001762407 |
| **Alta Wind II-V (2011) [2]** | 51 | 0.49823985 | 1 | 0.143613001 | 0.024630542 | 0.001762407 |
| **Alta Wind II-V (2011) [2]** | 52 | 0.49823985 | 1 | 0.143613001 | 0.024630542 | 0.001762407 |
| **Alta Wind II-V (2011) [2]** | 53 | 0.49823985 | 1 | 0.143613001 | 0.024630542 | 0.001762407 |
| **Alta Wind II-V (2011) [2]** | 54 | 0.49823985 | 1 | 0.143613001 | 0.024630542 | 0.001762407 |
| **Alta Wind II-V (2011) [2]** | 55 | 0.49823985 | 1 | 0.143613001 | 0.024630542 | 0.001762407 |
| **Alta Wind II-V (2011) [2]** | 56 | 0.49823985 | 1 | 0.143613001 | 0.024630542 | 0.001762407 |
| **Alta Wind II-V (2011) [2]** | 57 | 0.49823985 | 1 | 0.143613001 | 0.024630542 | 0.001762407 |
| **Alta Wind II-V (2011) [2]** | 58 | 0.49823985 | 1 | 0.143613001 | 0.024630542 | 0.001762407 |
| **Alta Wind II-V (2011) [2]** | 59 | 0.49823985 | 1 | 0.143613001 | 0.024630542 | 0.001762407 |
| **Alta Wind II-V (2011) [2]** | 60 | 0.49823985 | 1 | 0.143613001 | 0.024630542 | 0.001762407 |
| **Alta Wind II-V (2011) [2]** | 61 | 0.49823985 | 1 | 0.143613001 | 0.024630542 | 0.001762407 |
| **Alta Wind II-V (2011) [2]** | 62 | 0.49823985 | 1 | 0.143613001 | 0.024630542 | 0.001762407 |
| **Alta Wind II-V (2011) [2]** | 63 | 0.49823985 | 1 | 0.143613001 | 0.024630542 | 0.001762407 |
| **Alta Wind II-V (2011) [2]** | 64 | 0.49823985 | 1 | 0.143613001 | 0.024630542 | 0.001762407 |
| **Alta Wind II-V (2011) [2]** | 65 | 0.49823985 | 1 | 0.143613001 | 0.024630542 | 0.001762407 |
| **Alta Wind II-V (2011) [2]** | 66 | 0.49823985 | 1 | 0.143613001 | 0.024630542 | 0.001762407 |
| **Alta Wind II-V (2011) [2]** | 67 | 0.49823985 | 1 | 0.143613001 | 0.024630542 | 0.001762407 |
| **Alta Wind II-V (2011) [2]** | 68 | 0.49823985 | 1 | 0.143613001 | 0.024630542 | 0.001762407 |
| **Alta Wind II-V (2011) [2]** | 69 | 0.49823985 | 1 | 0.143613001 | 0.024630542 | 0.001762407 |
| **Alta Wind II-V (2011) [2]** | 70 | 0.49823985 | 1 | 0.143613001 | 0.024630542 | 0.001762407 |
| **Alta Wind II-V (2011) [2]** | 71 | 0.49823985 | 1 | 0.143613001 | 0.024630542 | 0.001762407 |
| **Alta Wind II-V (2011) [2]** | 72 | 0.49823985 | 1 | 0.143613001 | 0.024630542 | 0.001762407 |
| **Alta Wind II-V (2011) [2]** | 73 | 0.49823985 | 1 | 0.143613001 | 0.024630542 | 0.001762407 |
| **Alta Wind II-V (2011) [2]** | 74 | 0.49823985 | 1 | 0.143613001 | 0.024630542 | 0.001762407 |
| **Alta Wind II-V (2011) [2]** | 75 | 0.49823985 | 1 | 0.143613001 | 0.024630542 | 0.001762407 |
| **Alta Wind II-V (2011) [2]** | 76 | 0.49823985 | 1 | 0.143613001 | 0.024630542 | 0.001762407 |
| **Alta Wind II-V (2011) [2]** | 77 | 0.49823985 | 1 | 0.143613001 | 0.024630542 | 0.001762407 |
| **Alta Wind II-V (2011) [2]** | 78 | 0.49823985 | 1 | 0.143613001 | 0.024630542 | 0.001762407 |
| **Alta Wind II-V (2011) [2]** | 79 | 0.49823985 | 1 | 0.143613001 | 0.024630542 | 0.001762407 |
| **Alta Wind II-V (2011) [2]** | 80 | 0.49823985 | 1 | 0.143613001 | 0.024630542 | 0.001762407 |
| **Alta Wind II-V (2011) [2]** | 81 | 0.49823985 | 1 | 0.143613001 | 0.024630542 | 0.001762407 |
| **Alta Wind II-V (2011) [2]** | 82 | 0.49823985 | 1 | 0.143613001 | 0.024630542 | 0.001762407 |
| **Alta Wind II-V (2011) [2]** | 83 | 0.49823985 | 1 | 0.143613001 | 0.024630542 | 0.001762407 |
| **Alta Wind II-V (2011) [2]** | 84 | 0.49823985 | 1 | 0.143613001 | 0.024630542 | 0.001762407 |
| **Alta Wind II-V (2011) [2]** | 85 | 0.49823985 | 1 | 0.143613001 | 0.024630542 | 0.001762407 |
| **Alta Wind II-V (2011) [2]** | 86 | 0.49823985 | 1 | 0.143613001 | 0.024630542 | 0.001762407 |
| **Alta Wind II-V (2011) [2]** | 87 | 0.49823985 | 1 | 0.143613001 | 0.024630542 | 0.001762407 |
| **Alta Wind II-V (2011) [2]** | 88 | 0.49823985 | 1 | 0.143613001 | 0.024630542 | 0.001762407 |
| **Alta Wind II-V (2011) [2]** | 89 | 0.49823985 | 1 | 0.143613001 | 0.024630542 | 0.001762407 |
| **Alta Wind II-V (2011) [2]** | 90 | 0.49823985 | 1 | 0.143613001 | 0.024630542 | 0.001762407 |
| **Alta Wind II-V (2011) [2]** | 91 | 0.49823985 | 1 | 0.143613001 | 0.024630542 | 0.001762407 |
| **Alta Wind II-V (2011) [2]** | 92 | 0.49823985 | 1 | 0.143613001 | 0.024630542 | 0.001762407 |
| **Alta Wind II-V (2011) [2]** | 93 | 0.49823985 | 1 | 0.143613001 | 0.024630542 | 0.001762407 |
| **Alta Wind II-V (2011) [2]** | 94 | 0.49823985 | 1 | 0.143613001 | 0.024630542 | 0.001762407 |
| **Alta Wind II-V (2011) [2]** | 95 | 0.49823985 | 1 | 0.143613001 | 0.024630542 | 0.001762407 |
| **Alta Wind II-V (2011) [2]** | 96 | 0.49823985 | 1 | 0.143613001 | 0.024630542 | 0.001762407 |
| **Alta Wind II-V (2011) [2]** | 97 | 0.49823985 | 1 | 0.143613001 | 0.024630542 | 0.001762407 |
| **Alta Wind II-V (2011) [2]** | 98 | 0.49823985 | 1 | 0.143613001 | 0.024630542 | 0.001762407 |
| **Alta Wind II-V (2011) [2]** | 99 | 0.49823985 | 1 | 0.143613001 | 0.024630542 | 0.001762407 |
| **Alta Wind II-V (2011) [2]** | 100 | 0.49823985 | 1 | 0.143613001 | 0.024630542 | 0.001762407 |
| **Alta Wind II-V (2011) [2]** | 101 | 0.49823985 | 1 | 0.143613001 | 0.024630542 | 0.001762407 |
| **Alta Wind II-V (2011) [2]** | 102 | 0.49823985 | 1 | 0.143613001 | 0.024630542 | 0.001762407 |
| **Alta Wind II-V (2011) [2]** | 103 | 0.49823985 | 1 | 0.143613001 | 0.024630542 | 0.001762407 |
| **Alta Wind II-V (2011) [2]** | 104 | 0.49823985 | 1 | 0.143613001 | 0.024630542 | 0.001762407 |
| **Alta Wind II-V (2011) [2]** | 105 | 0.49823985 | 1 | 0.143613001 | 0.024630542 | 0.001762407 |
| **Alta Wind II-V (2011) [2]** | 106 | 0.49823985 | 1 | 0.143613001 | 0.024630542 | 0.001762407 |
| **Alta Wind II-V (2011) [2]** | 107 | 0.49823985 | 1 | 0.143613001 | 0.024630542 | 0.001762407 |
| **Alta Wind II-V (2011) [2]** | 108 | 0.49823985 | 1 | 0.143613001 | 0.024630542 | 0.001762407 |
| **Alta Wind II-V (2011) [2]** | 109 | 0.49823985 | 1 | 0.143613001 | 0.024630542 | 0.001762407 |
| **Alta Wind II-V (2011) [2]** | 110 | 0.49823985 | 1 | 0.143613001 | 0.024630542 | 0.001762407 |
| **Alta Wind II-V (2011) [2]** | 111 | 0.49823985 | 1 | 0.143613001 | 0.024630542 | 0.001762407 |
| **Alta Wind II-V (2011) [2]** | 112 | 0.49823985 | 1 | 0.143613001 | 0.024630542 | 0.001762407 |
| **Alta Wind II-V (2011) [2]** | 113 | 0.49823985 | 1 | 0.143613001 | 0.024630542 | 0.001762407 |
| **Alta Wind II-V (2011) [2]** | 114 | 0.49823985 | 1 | 0.143613001 | 0.024630542 | 0.001762407 |
| **Alta Wind II-V (2011) [2]** | 115 | 0.49823985 | 1 | 0.143613001 | 0.024630542 | 0.001762407 |
| **Alta Wind II-V (2011) [2]** | 116 | 0.49823985 | 1 | 0.143613001 | 0.024630542 | 0.001762407 |
| **Alta Wind II-V (2011) [2]** | 117 | 0.49823985 | 1 | 0.143613001 | 0.024630542 | 0.001762407 |
| **Alta Wind II-V (2011) [2]** | 118 | 0.49823985 | 1 | 0.143613001 | 0.024630542 | 0.001762407 |
| **Alta Wind II-V (2011) [2]** | 119 | 0.49823985 | 1 | 0.143613001 | 0.024630542 | 0.001762407 |
| **Alta Wind II-V (2011) [2]** | 120 | 0.49823985 | 1 | 0.143613001 | 0.024630542 | 0.001762407 |
| **Alta Wind II-V (2011) [2]** | 121 | 0.49823985 | 1 | 0.143613001 | 0.024630542 | 0.001762407 |
| **Alta Wind II-V (2011) [2]** | 122 | 0.49823985 | 1 | 0.143613001 | 0.024630542 | 0.001762407 |
| **Alta Wind II-V (2011) [2]** | 123 | 0.49823985 | 0 | 0.143613001 | 0.024630542 | 6.64E-10 |
| **Alta Wind II-V (2011) [2]** | 124 | 0.49823985 | 0 | 0.143613001 | 0.024630542 | 6.64E-10 |
| **Alta Wind II-V (2011) [2]** | 125 | 0.49823985 | 0 | 0.143613001 | 0.024630542 | 6.64E-10 |
| **Alta Wind II-V (2011) [2]** | 126 | 0.49823985 | 0 | 0.143613001 | 0.024630542 | 6.64E-10 |
| **Alta Wind II-V (2011) [2]** | 127 | 0.49823985 | 0 | 0.143613001 | 0.024630542 | 6.64E-10 |
| **Alta Wind II-V (2011) [2]** | 128 | 0.49823985 | 0 | 0.143613001 | 0.024630542 | 6.64E-10 |
| **Alta Wind II-V (2011) [2]** | 129 | 0.49823985 | 0 | 0.143613001 | 0.024630542 | 6.64E-10 |
| **Alta Wind II-V (2011) [2]** | 130 | 0.49823985 | 0 | 0.143613001 | 0.024630542 | 6.64E-10 |
| **Alta Wind II-V (2011) [2]** | 131 | 0.49823985 | 0 | 0.143613001 | 0.024630542 | 6.64E-10 |
| **Alta Wind II-V (2011) [2]** | 132 | 0.49823985 | 0 | 0.143613001 | 0.024630542 | 6.64E-10 |
| **Alta Wind II-V (2011) [2]** | 133 | 0.49823985 | 0 | 0.143613001 | 0.024630542 | 6.64E-10 |
| **Alta Wind II-V (2011) [2]** | 134 | 0.49823985 | 0 | 0.143613001 | 0.024630542 | 6.64E-10 |
| **Alta Wind II-V (2011) [2]** | 135 | 0.49823985 | 0 | 0.143613001 | 0.024630542 | 6.64E-10 |
| **Alta Wind II-V (2011) [2]** | 136 | 0.49823985 | 0 | 0.143613001 | 0.024630542 | 6.64E-10 |
| **Alta Wind II-V (2011) [2]** | 137 | 0.49823985 | 0 | 0.143613001 | 0.024630542 | 6.64E-10 |
| **Alta Wind II-V (2011) [2]** | 138 | 0.49823985 | 0 | 0.143613001 | 0.024630542 | 6.64E-10 |
| **Alta Wind II-V (2011) [2]** | 139 | 0.49823985 | 0 | 0.143613001 | 0.024630542 | 6.64E-10 |
| **Alta Wind II-V (2011) [2]** | 140 | 0.49823985 | 0 | 0.143613001 | 0.024630542 | 6.64E-10 |
| **Alta Wind II-V (2011) [2]** | 141 | 0.49823985 | 0 | 0.143613001 | 0.024630542 | 6.64E-10 |
| **Alta Wind II-V (2011) [2]** | 142 | 0.49823985 | 0 | 0.143613001 | 0.024630542 | 6.64E-10 |
| **Alta Wind II-V (2011) [2]** | 143 | 0.49823985 | 0 | 0.143613001 | 0.024630542 | 6.64E-10 |
| **Alta Wind II-V (2011) [2]** | 144 | 0.49823985 | 0 | 0.143613001 | 0.024630542 | 6.64E-10 |
| **Barton Chapel [3]** | 3 | 0.42485581 | 1 | 0.045351474 | 0.123152709 | 0.002372886 |
| **Barton Chapel [3]** | 4 | 0.42485581 | 1 | 0.045351474 | 0.123152709 | 0.002372886 |
| **Barton Chapel [3]** | 5 | 0.42485581 | 1 | 0.045351474 | 0.123152709 | 0.002372886 |
| **Barton Chapel [3]** | 6 | 0.42485581 | 1 | 0.045351474 | 0.123152709 | 0.002372886 |
| **Barton Chapel [3]** | 7 | 0.42485581 | 1 | 0.045351474 | 0.123152709 | 0.002372886 |
| **Barton Chapel [3]** | 8 | 0.42485581 | 1 | 0.045351474 | 0.123152709 | 0.002372886 |
| **Barton Chapel [3]** | 9 | 0.42485581 | 1 | 0.045351474 | 0.123152709 | 0.002372886 |
| **Barton Chapel [3]** | 10 | 0.42485581 | 1 | 0.045351474 | 0.123152709 | 0.002372886 |
| **Barton Chapel [3]** | 11 | 0.42485581 | 1 | 0.045351474 | 0.123152709 | 0.002372886 |
| **Barton Chapel [3]** | 12 | 0.42485581 | 1 | 0.045351474 | 0.123152709 | 0.002372886 |
| **Barton Chapel [3]** | 13 | 0.42485581 | 1 | 0.045351474 | 0.123152709 | 0.002372886 |
| **Barton Chapel [3]** | 14 | 0.42485581 | 1 | 0.045351474 | 0.123152709 | 0.002372886 |
| **Barton Chapel [3]** | 15 | 0.42485581 | 1 | 0.045351474 | 0.123152709 | 0.002372886 |
| **Barton Chapel [3]** | 16 | 0.42485581 | 1 | 0.045351474 | 0.123152709 | 0.002372886 |
| **Barton Chapel [3]** | 17 | 0.42485581 | 1 | 0.045351474 | 0.123152709 | 0.002372886 |
| **Barton Chapel [3]** | 18 | 0.42485581 | 1 | 0.045351474 | 0.123152709 | 0.002372886 |
| **Barton Chapel [3]** | 19 | 0.42485581 | 1 | 0.045351474 | 0.123152709 | 0.002372886 |
| **Barton Chapel [3]** | 20 | 0.42485581 | 1 | 0.045351474 | 0.123152709 | 0.002372886 |
| **Barton Chapel [3]** | 21 | 0.42485581 | 1 | 0.045351474 | 0.123152709 | 0.002372886 |
| **Barton Chapel [3]** | 22 | 0.42485581 | 1 | 0.045351474 | 0.123152709 | 0.002372886 |
| **Barton Chapel [3]** | 23 | 0.42485581 | 1 | 0.045351474 | 0.123152709 | 0.002372886 |
| **Barton Chapel [3]** | 24 | 0.42485581 | 1 | 0.045351474 | 0.123152709 | 0.002372886 |
| **Barton Chapel [3]** | 25 | 0.42485581 | 1 | 0.045351474 | 0.123152709 | 0.002372886 |
| **Barton Chapel [3]** | 26 | 0.42485581 | 1 | 0.045351474 | 0.123152709 | 0.002372886 |
| **Barton Chapel [3]** | 27 | 0.42485581 | 1 | 0.045351474 | 0.123152709 | 0.002372886 |
| **Barton Chapel [3]** | 28 | 0.42485581 | 1 | 0.045351474 | 0.123152709 | 0.002372886 |
| **Barton Chapel [3]** | 29 | 0.42485581 | 1 | 0.045351474 | 0.123152709 | 0.002372886 |
| **Barton Chapel [3]** | 30 | 0.42485581 | 1 | 0.045351474 | 0.123152709 | 0.002372886 |
| **Barton Chapel [3]** | 31 | 0.42485581 | 1 | 0.045351474 | 0.123152709 | 0.002372886 |
| **Barton Chapel [3]** | 32 | 0.42485581 | 1 | 0.045351474 | 0.123152709 | 0.002372886 |
| **Barton Chapel [3]** | 33 | 0.42485581 | 1 | 0.045351474 | 0.123152709 | 0.002372886 |
| **Barton Chapel [3]** | 34 | 0.42485581 | 1 | 0.045351474 | 0.123152709 | 0.002372886 |
| **Barton Chapel [3]** | 35 | 0.42485581 | 1 | 0.045351474 | 0.123152709 | 0.002372886 |
| **Barton Chapel [3]** | 36 | 0.42485581 | 1 | 0.045351474 | 0.123152709 | 0.002372886 |
| **Barton Chapel [3]** | 37 | 0.42485581 | 1 | 0.045351474 | 0.123152709 | 0.002372886 |
| **Barton Chapel [3]** | 38 | 0.42485581 | 1 | 0.045351474 | 0.123152709 | 0.002372886 |
| **Barton Chapel [3]** | 39 | 0.42485581 | 1 | 0.045351474 | 0.123152709 | 0.002372886 |
| **Barton Chapel [3]** | 40 | 0.42485581 | 1 | 0.045351474 | 0.123152709 | 0.002372886 |
| **Barton Chapel [3]** | 41 | 0.42485581 | 1 | 0.045351474 | 0.123152709 | 0.002372886 |
| **Barton Chapel [3]** | 42 | 0.42485581 | 1 | 0.045351474 | 0.123152709 | 0.002372886 |
| **Barton Chapel [3]** | 43 | 0.42485581 | 1 | 0.045351474 | 0.123152709 | 0.002372886 |
| **Barton Chapel [3]** | 44 | 0.42485581 | 1 | 0.045351474 | 0.123152709 | 0.002372886 |
| **Barton Chapel [3]** | 45 | 0.42485581 | 1 | 0.045351474 | 0.123152709 | 0.002372886 |
| **Barton Chapel [3]** | 46 | 0.42485581 | 1 | 0.045351474 | 0.123152709 | 0.002372886 |
| **Barton Chapel [3]** | 47 | 0.42485581 | 1 | 0.045351474 | 0.123152709 | 0.002372886 |
| **Barton Chapel [3]** | 48 | 0.42485581 | 1 | 0.045351474 | 0.123152709 | 0.002372886 |
| **Barton Chapel [3]** | 49 | 0.42485581 | 1 | 0.045351474 | 0.123152709 | 0.002372886 |
| **Barton Chapel [3]** | 50 | 0.42485581 | 1 | 0.045351474 | 0.123152709 | 0.002372886 |
| **Barton Chapel [3]** | 51 | 0.42485581 | 1 | 0.045351474 | 0.123152709 | 0.002372886 |
| **Barton Chapel [3]** | 52 | 0.42485581 | 1 | 0.045351474 | 0.123152709 | 0.002372886 |
| **Barton Chapel [3]** | 53 | 0.42485581 | 1 | 0.045351474 | 0.123152709 | 0.002372886 |
| **Barton Chapel [3]** | 54 | 0.42485581 | 1 | 0.045351474 | 0.123152709 | 0.002372886 |
| **Barton Chapel [3]** | 55 | 0.42485581 | 1 | 0.045351474 | 0.123152709 | 0.002372886 |
| **Barton Chapel [3]** | 56 | 0.42485581 | 1 | 0.045351474 | 0.123152709 | 0.002372886 |
| **Barton Chapel [3]** | 57 | 0.42485581 | 1 | 0.045351474 | 0.123152709 | 0.002372886 |
| **Barton Chapel [3]** | 58 | 0.42485581 | 1 | 0.045351474 | 0.123152709 | 0.002372886 |
| **Barton Chapel [3]** | 59 | 0.42485581 | 1 | 0.045351474 | 0.123152709 | 0.002372886 |
| **Barton Chapel [3]** | 60 | 0.42485581 | 1 | 0.045351474 | 0.123152709 | 0.002372886 |
| **Barton Chapel [3]** | 61 | 0.42485581 | 1 | 0.045351474 | 0.123152709 | 0.002372886 |
| **Barton Chapel [3]** | 62 | 0.42485581 | 1 | 0.045351474 | 0.123152709 | 0.002372886 |
| **Barton Chapel [3]** | 63 | 0.42485581 | 1 | 0.045351474 | 0.123152709 | 0.002372886 |
| **Barton Chapel [3]** | 64 | 0.42485581 | 1 | 0.045351474 | 0.123152709 | 0.002372886 |
| **Barton Chapel [3]** | 65 | 0.42485581 | 1 | 0.045351474 | 0.123152709 | 0.002372886 |
| **Barton Chapel [3]** | 66 | 0.42485581 | 1 | 0.045351474 | 0.123152709 | 0.002372886 |
| **Barton Chapel [3]** | 67 | 0.42485581 | 1 | 0.045351474 | 0.123152709 | 0.002372886 |
| **Barton Chapel [3]** | 68 | 0.42485581 | 1 | 0.045351474 | 0.123152709 | 0.002372886 |
| **Barton Chapel [3]** | 69 | 0.42485581 | 1 | 0.045351474 | 0.123152709 | 0.002372886 |
| **Barton Chapel [3]** | 70 | 0.42485581 | 1 | 0.045351474 | 0.123152709 | 0.002372886 |
| **Barton Chapel [3]** | 71 | 0.42485581 | 1 | 0.045351474 | 0.123152709 | 0.002372886 |
| **Barton Chapel [3]** | 72 | 0.42485581 | 1 | 0.045351474 | 0.123152709 | 0.002372886 |
| **Barton Chapel [3]** | 73 | 0.42485581 | 1 | 0.045351474 | 0.123152709 | 0.002372886 |
| **Barton Chapel [3]** | 74 | 0.42485581 | 1 | 0.045351474 | 0.123152709 | 0.002372886 |
| **Barton Chapel [3]** | 75 | 0.42485581 | 1 | 0.045351474 | 0.123152709 | 0.002372886 |
| **Barton Chapel [3]** | 76 | 0.42485581 | 1 | 0.045351474 | 0.123152709 | 0.002372886 |
| **Barton Chapel [3]** | 77 | 0.42485581 | 1 | 0.045351474 | 0.123152709 | 0.002372886 |
| **Barton Chapel [3]** | 78 | 0.42485581 | 1 | 0.045351474 | 0.123152709 | 0.002372886 |
| **Barton Chapel [3]** | 79 | 0.42485581 | 1 | 0.045351474 | 0.123152709 | 0.002372886 |
| **Barton Chapel [3]** | 80 | 0.42485581 | 1 | 0.045351474 | 0.123152709 | 0.002372886 |
| **Barton Chapel [3]** | 81 | 0.42485581 | 1 | 0.045351474 | 0.123152709 | 0.002372886 |
| **Barton Chapel [3]** | 82 | 0.42485581 | 1 | 0.045351474 | 0.123152709 | 0.002372886 |
| **Barton Chapel [3]** | 83 | 0.42485581 | 1 | 0.045351474 | 0.123152709 | 0.002372886 |
| **Barton Chapel [3]** | 84 | 0.42485581 | 1 | 0.045351474 | 0.123152709 | 0.002372886 |
| **Barton Chapel [3]** | 85 | 0.42485581 | 1 | 0.045351474 | 0.123152709 | 0.002372886 |
| **Barton Chapel [3]** | 86 | 0.42485581 | 1 | 0.045351474 | 0.123152709 | 0.002372886 |
| **Barton Chapel [3]** | 87 | 0.42485581 | 1 | 0.045351474 | 0.123152709 | 0.002372886 |
| **Barton Chapel [3]** | 88 | 0.42485581 | 1 | 0.045351474 | 0.123152709 | 0.002372886 |
| **Barton Chapel [3]** | 89 | 0.42485581 | 1 | 0.045351474 | 0.123152709 | 0.002372886 |
| **Barton Chapel [3]** | 90 | 0.42485581 | 1 | 0.045351474 | 0.123152709 | 0.002372886 |
| **Barton Chapel [3]** | 91 | 0.42485581 | 1 | 0.045351474 | 0.123152709 | 0.002372886 |
| **Barton Chapel [3]** | 92 | 0.42485581 | 1 | 0.045351474 | 0.123152709 | 0.002372886 |
| **Barton Chapel [3]** | 93 | 0.42485581 | 1 | 0.045351474 | 0.123152709 | 0.002372886 |
| **Barton Chapel [3]** | 94 | 0.42485581 | 1 | 0.045351474 | 0.123152709 | 0.002372886 |
| **Barton Chapel [3]** | 95 | 0.42485581 | 1 | 0.045351474 | 0.123152709 | 0.002372886 |
| **Barton Chapel [3]** | 96 | 0.42485581 | 1 | 0.045351474 | 0.123152709 | 0.002372886 |
| **Barton Chapel [3]** | 97 | 0.42485581 | 1 | 0.045351474 | 0.123152709 | 0.002372886 |
| **Barton Chapel [3]** | 98 | 0.42485581 | 1 | 0.045351474 | 0.123152709 | 0.002372886 |
| **Barton Chapel [3]** | 99 | 0.42485581 | 1 | 0.045351474 | 0.123152709 | 0.002372886 |
| **Barton Chapel [3]** | 100 | 0.42485581 | 1 | 0.045351474 | 0.123152709 | 0.002372886 |
| **Barton Chapel [3]** | 101 | 0.42485581 | 1 | 0.045351474 | 0.123152709 | 0.002372886 |
| **Barton Chapel [3]** | 102 | 0.42485581 | 1 | 0.045351474 | 0.123152709 | 0.002372886 |
| **Barton Chapel [3]** | 103 | 0.42485581 | 0 | 0.045351474 | 0.123152709 | 6.64E-10 |
| **Barton Chapel [3]** | 104 | 0.42485581 | 0 | 0.045351474 | 0.123152709 | 6.64E-10 |
| **Barton Chapel [3]** | 105 | 0.42485581 | 0 | 0.045351474 | 0.123152709 | 6.64E-10 |
| **Barton Chapel [3]** | 106 | 0.42485581 | 0 | 0.045351474 | 0.123152709 | 6.64E-10 |
| **Barton Chapel [3]** | 107 | 0.42485581 | 0 | 0.045351474 | 0.123152709 | 6.64E-10 |
| **Barton Chapel [3]** | 108 | 0.42485581 | 0 | 0.045351474 | 0.123152709 | 6.64E-10 |
| **Barton Chapel [3]** | 109 | 0.42485581 | 0 | 0.045351474 | 0.123152709 | 6.64E-10 |
| **Barton Chapel [3]** | 110 | 0.42485581 | 0 | 0.045351474 | 0.123152709 | 6.64E-10 |
| **Barton Chapel [3]** | 111 | 0.42485581 | 0 | 0.045351474 | 0.123152709 | 6.64E-10 |
| **Barton Chapel [3]** | 112 | 0.42485581 | 0 | 0.045351474 | 0.123152709 | 6.64E-10 |
| **Barton Chapel [3]** | 113 | 0.42485581 | 0 | 0.045351474 | 0.123152709 | 6.64E-10 |
| **Barton Chapel [3]** | 114 | 0.42485581 | 0 | 0.045351474 | 0.123152709 | 6.64E-10 |
| **Barton Chapel [3]** | 115 | 0.42485581 | 0 | 0.045351474 | 0.123152709 | 6.64E-10 |
| **Barton Chapel [3]** | 116 | 0.42485581 | 0 | 0.045351474 | 0.123152709 | 6.64E-10 |
| **Barton Chapel [3]** | 117 | 0.42485581 | 0 | 0.045351474 | 0.123152709 | 6.64E-10 |
| **Barton Chapel [3]** | 118 | 0.42485581 | 0 | 0.045351474 | 0.123152709 | 6.64E-10 |
| **Barton Chapel [3]** | 119 | 0.42485581 | 0 | 0.045351474 | 0.123152709 | 6.64E-10 |
| **Barton Chapel [3]** | 120 | 0.42485581 | 0 | 0.045351474 | 0.123152709 | 6.64E-10 |
| **Barton Chapel [3]** | 121 | 0.42485581 | 0 | 0.045351474 | 0.123152709 | 6.64E-10 |
| **Barton Chapel [3]** | 122 | 0.42485581 | 0 | 0.045351474 | 0.123152709 | 6.64E-10 |
| **Barton Chapel [3]** | 123 | 0.42485581 | 0 | 0.045351474 | 0.123152709 | 6.64E-10 |
| **Barton Chapel [3]** | 124 | 0.42485581 | 0 | 0.045351474 | 0.123152709 | 6.64E-10 |
| **Barton Chapel [3]** | 125 | 0.42485581 | 0 | 0.045351474 | 0.123152709 | 6.64E-10 |
| **Barton Chapel [3]** | 126 | 0.42485581 | 0 | 0.045351474 | 0.123152709 | 6.64E-10 |
| **Barton Chapel [3]** | 127 | 0.42485581 | 0 | 0.045351474 | 0.123152709 | 6.64E-10 |
| **Barton Chapel [3]** | 128 | 0.42485581 | 0 | 0.045351474 | 0.123152709 | 6.64E-10 |
| **Barton Chapel [3]** | 129 | 0.42485581 | 0 | 0.045351474 | 0.123152709 | 6.64E-10 |
| **Barton Chapel [3]** | 130 | 0.42485581 | 0 | 0.045351474 | 0.123152709 | 6.64E-10 |
| **Barton Chapel [3]** | 131 | 0.42485581 | 0 | 0.045351474 | 0.123152709 | 6.64E-10 |
| **Barton Chapel [3]** | 132 | 0.42485581 | 0 | 0.045351474 | 0.123152709 | 6.64E-10 |
| **Barton Chapel [3]** | 133 | 0.42485581 | 0 | 0.045351474 | 0.123152709 | 6.64E-10 |
| **Barton Chapel [3]** | 134 | 0.42485581 | 0 | 0.045351474 | 0.123152709 | 6.64E-10 |
| **Barton Chapel [3]** | 135 | 0.42485581 | 0 | 0.045351474 | 0.123152709 | 6.64E-10 |
| **Barton Chapel [3]** | 136 | 0.42485581 | 0 | 0.045351474 | 0.123152709 | 6.64E-10 |
| **Barton Chapel [3]** | 137 | 0.42485581 | 0 | 0.045351474 | 0.123152709 | 6.64E-10 |
| **Barton Chapel [3]** | 138 | 0.42485581 | 0 | 0.045351474 | 0.123152709 | 6.64E-10 |
| **Barton Chapel [3]** | 139 | 0.42485581 | 0 | 0.045351474 | 0.123152709 | 6.64E-10 |
| **Barton Chapel [3]** | 140 | 0.42485581 | 0 | 0.045351474 | 0.123152709 | 6.64E-10 |
| **Barton Chapel [3]** | 141 | 0.42485581 | 0 | 0.045351474 | 0.123152709 | 6.64E-10 |
| **Barton Chapel [3]** | 142 | 0.42485581 | 0 | 0.045351474 | 0.123152709 | 6.64E-10 |
| **Barton Chapel [3]** | 143 | 0.42485581 | 0 | 0.045351474 | 0.123152709 | 6.64E-10 |
| **Barton Chapel [3]** | 144 | 0.42485581 | 0 | 0.045351474 | 0.123152709 | 6.64E-10 |
| **Big Horn [4]** | 3 | 0.57707637 | 1 | 0.100529101 | 0.054187192 | 0.00314356 |
| **Big Horn [4]** | 4 | 0.57707637 | 1 | 0.100529101 | 0.054187192 | 0.00314356 |
| **Big Horn [4]** | 5 | 0.57707637 | 1 | 0.100529101 | 0.054187192 | 0.00314356 |
| **Big Horn [4]** | 6 | 0.57707637 | 1 | 0.100529101 | 0.054187192 | 0.00314356 |
| **Big Horn [4]** | 7 | 0.57707637 | 1 | 0.100529101 | 0.054187192 | 0.00314356 |
| **Big Horn [4]** | 8 | 0.57707637 | 1 | 0.100529101 | 0.054187192 | 0.00314356 |
| **Big Horn [4]** | 9 | 0.57707637 | 1 | 0.100529101 | 0.054187192 | 0.00314356 |
| **Big Horn [4]** | 10 | 0.57707637 | 1 | 0.100529101 | 0.054187192 | 0.00314356 |
| **Big Horn [4]** | 11 | 0.57707637 | 1 | 0.100529101 | 0.054187192 | 0.00314356 |
| **Big Horn [4]** | 12 | 0.57707637 | 1 | 0.100529101 | 0.054187192 | 0.00314356 |
| **Big Horn [4]** | 13 | 0.57707637 | 1 | 0.100529101 | 0.054187192 | 0.00314356 |
| **Big Horn [4]** | 14 | 0.57707637 | 1 | 0.100529101 | 0.054187192 | 0.00314356 |
| **Big Horn [4]** | 15 | 0.57707637 | 1 | 0.100529101 | 0.054187192 | 0.00314356 |
| **Big Horn [4]** | 16 | 0.57707637 | 1 | 0.100529101 | 0.054187192 | 0.00314356 |
| **Big Horn [4]** | 17 | 0.57707637 | 1 | 0.100529101 | 0.054187192 | 0.00314356 |
| **Big Horn [4]** | 18 | 0.57707637 | 1 | 0.100529101 | 0.054187192 | 0.00314356 |
| **Big Horn [4]** | 19 | 0.57707637 | 1 | 0.100529101 | 0.054187192 | 0.00314356 |
| **Big Horn [4]** | 20 | 0.57707637 | 1 | 0.100529101 | 0.054187192 | 0.00314356 |
| **Big Horn [4]** | 21 | 0.57707637 | 1 | 0.100529101 | 0.054187192 | 0.00314356 |
| **Big Horn [4]** | 22 | 0.57707637 | 1 | 0.100529101 | 0.054187192 | 0.00314356 |
| **Big Horn [4]** | 23 | 0.57707637 | 1 | 0.100529101 | 0.054187192 | 0.00314356 |
| **Big Horn [4]** | 24 | 0.57707637 | 1 | 0.100529101 | 0.054187192 | 0.00314356 |
| **Big Horn [4]** | 25 | 0.57707637 | 1 | 0.100529101 | 0.054187192 | 0.00314356 |
| **Big Horn [4]** | 26 | 0.57707637 | 1 | 0.100529101 | 0.054187192 | 0.00314356 |
| **Big Horn [4]** | 27 | 0.57707637 | 1 | 0.100529101 | 0.054187192 | 0.00314356 |
| **Big Horn [4]** | 28 | 0.57707637 | 1 | 0.100529101 | 0.054187192 | 0.00314356 |
| **Big Horn [4]** | 29 | 0.57707637 | 1 | 0.100529101 | 0.054187192 | 0.00314356 |
| **Big Horn [4]** | 30 | 0.57707637 | 1 | 0.100529101 | 0.054187192 | 0.00314356 |
| **Big Horn [4]** | 31 | 0.57707637 | 1 | 0.100529101 | 0.054187192 | 0.00314356 |
| **Big Horn [4]** | 32 | 0.57707637 | 1 | 0.100529101 | 0.054187192 | 0.00314356 |
| **Big Horn [4]** | 33 | 0.57707637 | 1 | 0.100529101 | 0.054187192 | 0.00314356 |
| **Big Horn [4]** | 34 | 0.57707637 | 1 | 0.100529101 | 0.054187192 | 0.00314356 |
| **Big Horn [4]** | 35 | 0.57707637 | 1 | 0.100529101 | 0.054187192 | 0.00314356 |
| **Big Horn [4]** | 36 | 0.57707637 | 1 | 0.100529101 | 0.054187192 | 0.00314356 |
| **Big Horn [4]** | 37 | 0.57707637 | 1 | 0.100529101 | 0.054187192 | 0.00314356 |
| **Big Horn [4]** | 38 | 0.57707637 | 1 | 0.100529101 | 0.054187192 | 0.00314356 |
| **Big Horn [4]** | 39 | 0.57707637 | 1 | 0.100529101 | 0.054187192 | 0.00314356 |
| **Big Horn [4]** | 40 | 0.57707637 | 1 | 0.100529101 | 0.054187192 | 0.00314356 |
| **Big Horn [4]** | 41 | 0.57707637 | 1 | 0.100529101 | 0.054187192 | 0.00314356 |
| **Big Horn [4]** | 42 | 0.57707637 | 1 | 0.100529101 | 0.054187192 | 0.00314356 |
| **Big Horn [4]** | 43 | 0.57707637 | 1 | 0.100529101 | 0.054187192 | 0.00314356 |
| **Big Horn [4]** | 44 | 0.57707637 | 1 | 0.100529101 | 0.054187192 | 0.00314356 |
| **Big Horn [4]** | 45 | 0.57707637 | 1 | 0.100529101 | 0.054187192 | 0.00314356 |
| **Big Horn [4]** | 46 | 0.57707637 | 1 | 0.100529101 | 0.054187192 | 0.00314356 |
| **Big Horn [4]** | 47 | 0.57707637 | 1 | 0.100529101 | 0.054187192 | 0.00314356 |
| **Big Horn [4]** | 48 | 0.57707637 | 1 | 0.100529101 | 0.054187192 | 0.00314356 |
| **Big Horn [4]** | 49 | 0.57707637 | 1 | 0.100529101 | 0.054187192 | 0.00314356 |
| **Big Horn [4]** | 50 | 0.57707637 | 1 | 0.100529101 | 0.054187192 | 0.00314356 |
| **Big Horn [4]** | 51 | 0.57707637 | 1 | 0.100529101 | 0.054187192 | 0.00314356 |
| **Big Horn [4]** | 52 | 0.57707637 | 1 | 0.100529101 | 0.054187192 | 0.00314356 |
| **Big Horn [4]** | 53 | 0.57707637 | 1 | 0.100529101 | 0.054187192 | 0.00314356 |
| **Big Horn [4]** | 54 | 0.57707637 | 1 | 0.100529101 | 0.054187192 | 0.00314356 |
| **Big Horn [4]** | 55 | 0.57707637 | 1 | 0.100529101 | 0.054187192 | 0.00314356 |
| **Big Horn [4]** | 56 | 0.57707637 | 1 | 0.100529101 | 0.054187192 | 0.00314356 |
| **Big Horn [4]** | 57 | 0.57707637 | 1 | 0.100529101 | 0.054187192 | 0.00314356 |
| **Big Horn [4]** | 58 | 0.57707637 | 1 | 0.100529101 | 0.054187192 | 0.00314356 |
| **Big Horn [4]** | 59 | 0.57707637 | 1 | 0.100529101 | 0.054187192 | 0.00314356 |
| **Big Horn [4]** | 60 | 0.57707637 | 1 | 0.100529101 | 0.054187192 | 0.00314356 |
| **Big Horn [4]** | 61 | 0.57707637 | 1 | 0.100529101 | 0.054187192 | 0.00314356 |
| **Big Horn [4]** | 62 | 0.57707637 | 1 | 0.100529101 | 0.054187192 | 0.00314356 |
| **Big Horn [4]** | 63 | 0.57707637 | 1 | 0.100529101 | 0.054187192 | 0.00314356 |
| **Big Horn [4]** | 64 | 0.57707637 | 1 | 0.100529101 | 0.054187192 | 0.00314356 |
| **Big Horn [4]** | 65 | 0.57707637 | 1 | 0.100529101 | 0.054187192 | 0.00314356 |
| **Big Horn [4]** | 66 | 0.57707637 | 1 | 0.100529101 | 0.054187192 | 0.00314356 |
| **Big Horn [4]** | 67 | 0.57707637 | 1 | 0.100529101 | 0.054187192 | 0.00314356 |
| **Big Horn [4]** | 68 | 0.57707637 | 1 | 0.100529101 | 0.054187192 | 0.00314356 |
| **Big Horn [4]** | 69 | 0.57707637 | 1 | 0.100529101 | 0.054187192 | 0.00314356 |
| **Big Horn [4]** | 70 | 0.57707637 | 1 | 0.100529101 | 0.054187192 | 0.00314356 |
| **Big Horn [4]** | 71 | 0.57707637 | 1 | 0.100529101 | 0.054187192 | 0.00314356 |
| **Big Horn [4]** | 72 | 0.57707637 | 1 | 0.100529101 | 0.054187192 | 0.00314356 |
| **Big Horn [4]** | 73 | 0.57707637 | 1 | 0.100529101 | 0.054187192 | 0.00314356 |
| **Big Horn [4]** | 74 | 0.57707637 | 1 | 0.100529101 | 0.054187192 | 0.00314356 |
| **Big Horn [4]** | 75 | 0.57707637 | 1 | 0.100529101 | 0.054187192 | 0.00314356 |
| **Big Horn [4]** | 76 | 0.57707637 | 1 | 0.100529101 | 0.054187192 | 0.00314356 |
| **Big Horn [4]** | 77 | 0.57707637 | 1 | 0.100529101 | 0.054187192 | 0.00314356 |
| **Big Horn [4]** | 78 | 0.57707637 | 1 | 0.100529101 | 0.054187192 | 0.00314356 |
| **Big Horn [4]** | 79 | 0.57707637 | 1 | 0.100529101 | 0.054187192 | 0.00314356 |
| **Big Horn [4]** | 80 | 0.57707637 | 1 | 0.100529101 | 0.054187192 | 0.00314356 |
| **Big Horn [4]** | 81 | 0.57707637 | 1 | 0.100529101 | 0.054187192 | 0.00314356 |
| **Big Horn [4]** | 82 | 0.57707637 | 1 | 0.100529101 | 0.054187192 | 0.00314356 |
| **Big Horn [4]** | 83 | 0.57707637 | 1 | 0.100529101 | 0.054187192 | 0.00314356 |
| **Big Horn [4]** | 84 | 0.57707637 | 1 | 0.100529101 | 0.054187192 | 0.00314356 |
| **Big Horn [4]** | 85 | 0.57707637 | 1 | 0.100529101 | 0.054187192 | 0.00314356 |
| **Big Horn [4]** | 86 | 0.57707637 | 1 | 0.100529101 | 0.054187192 | 0.00314356 |
| **Big Horn [4]** | 87 | 0.57707637 | 1 | 0.100529101 | 0.054187192 | 0.00314356 |
| **Big Horn [4]** | 88 | 0.57707637 | 1 | 0.100529101 | 0.054187192 | 0.00314356 |
| **Big Horn [4]** | 89 | 0.57707637 | 1 | 0.100529101 | 0.054187192 | 0.00314356 |
| **Big Horn [4]** | 90 | 0.57707637 | 1 | 0.100529101 | 0.054187192 | 0.00314356 |
| **Big Horn [4]** | 91 | 0.57707637 | 1 | 0.100529101 | 0.054187192 | 0.00314356 |
| **Big Horn [4]** | 92 | 0.57707637 | 1 | 0.100529101 | 0.054187192 | 0.00314356 |
| **Big Horn [4]** | 93 | 0.57707637 | 0 | 0.100529101 | 0.054187192 | 6.64E-10 |
| **Big Horn [4]** | 94 | 0.57707637 | 0 | 0.100529101 | 0.054187192 | 6.64E-10 |
| **Big Horn [4]** | 95 | 0.57707637 | 0 | 0.100529101 | 0.054187192 | 6.64E-10 |
| **Big Horn [4]** | 96 | 0.57707637 | 0 | 0.100529101 | 0.054187192 | 6.64E-10 |
| **Big Horn [4]** | 97 | 0.57707637 | 0 | 0.100529101 | 0.054187192 | 6.64E-10 |
| **Big Horn [4]** | 98 | 0.57707637 | 0 | 0.100529101 | 0.054187192 | 6.64E-10 |
| **Big Horn [4]** | 99 | 0.57707637 | 0 | 0.100529101 | 0.054187192 | 6.64E-10 |
| **Big Horn [4]** | 100 | 0.57707637 | 0 | 0.100529101 | 0.054187192 | 6.64E-10 |
| **Big Horn [4]** | 101 | 0.57707637 | 0 | 0.100529101 | 0.054187192 | 6.64E-10 |
| **Big Horn [4]** | 102 | 0.57707637 | 0 | 0.100529101 | 0.054187192 | 6.64E-10 |
| **Big Horn [4]** | 103 | 0.57707637 | 0 | 0.100529101 | 0.054187192 | 6.64E-10 |
| **Big Horn [4]** | 104 | 0.57707637 | 0 | 0.100529101 | 0.054187192 | 6.64E-10 |
| **Big Horn [4]** | 105 | 0.57707637 | 0 | 0.100529101 | 0.054187192 | 6.64E-10 |
| **Big Horn [4]** | 106 | 0.57707637 | 0 | 0.100529101 | 0.054187192 | 6.64E-10 |
| **Big Horn [4]** | 107 | 0.57707637 | 0 | 0.100529101 | 0.054187192 | 6.64E-10 |
| **Big Horn [4]** | 108 | 0.57707637 | 0 | 0.100529101 | 0.054187192 | 6.64E-10 |
| **Big Horn [4]** | 109 | 0.57707637 | 0 | 0.100529101 | 0.054187192 | 6.64E-10 |
| **Big Horn [4]** | 110 | 0.57707637 | 0 | 0.100529101 | 0.054187192 | 6.64E-10 |
| **Big Horn [4]** | 111 | 0.57707637 | 0 | 0.100529101 | 0.054187192 | 6.64E-10 |
| **Big Horn [4]** | 112 | 0.57707637 | 0 | 0.100529101 | 0.054187192 | 6.64E-10 |
| **Big Horn [4]** | 113 | 0.57707637 | 0 | 0.100529101 | 0.054187192 | 6.64E-10 |
| **Big Horn [4]** | 114 | 0.57707637 | 0 | 0.100529101 | 0.054187192 | 6.64E-10 |
| **Big Horn [4]** | 115 | 0.57707637 | 0 | 0.100529101 | 0.054187192 | 6.64E-10 |
| **Big Horn [4]** | 116 | 0.57707637 | 0 | 0.100529101 | 0.054187192 | 6.64E-10 |
| **Big Horn [4]** | 117 | 0.57707637 | 0 | 0.100529101 | 0.054187192 | 6.64E-10 |
| **Big Horn [4]** | 118 | 0.57707637 | 0 | 0.100529101 | 0.054187192 | 6.64E-10 |
| **Big Horn [4]** | 119 | 0.57707637 | 0 | 0.100529101 | 0.054187192 | 6.64E-10 |
| **Big Horn [4]** | 120 | 0.57707637 | 0 | 0.100529101 | 0.054187192 | 6.64E-10 |
| **Big Horn [4]** | 121 | 0.57707637 | 0 | 0.100529101 | 0.054187192 | 6.64E-10 |
| **Big Horn [4]** | 122 | 0.57707637 | 0 | 0.100529101 | 0.054187192 | 6.64E-10 |
| **Big Horn [4]** | 123 | 0.57707637 | 0 | 0.100529101 | 0.054187192 | 6.64E-10 |
| **Big Horn [4]** | 124 | 0.57707637 | 0 | 0.100529101 | 0.054187192 | 6.64E-10 |
| **Big Horn [4]** | 125 | 0.57707637 | 0 | 0.100529101 | 0.054187192 | 6.64E-10 |
| **Big Horn [4]** | 126 | 0.57707637 | 0 | 0.100529101 | 0.054187192 | 6.64E-10 |
| **Big Horn [4]** | 127 | 0.57707637 | 0 | 0.100529101 | 0.054187192 | 6.64E-10 |
| **Big Horn [4]** | 128 | 0.57707637 | 0 | 0.100529101 | 0.054187192 | 6.64E-10 |
| **Big Horn [4]** | 129 | 0.57707637 | 0 | 0.100529101 | 0.054187192 | 6.64E-10 |
| **Big Horn [4]** | 130 | 0.57707637 | 0 | 0.100529101 | 0.054187192 | 6.64E-10 |
| **Big Horn [4]** | 131 | 0.57707637 | 0 | 0.100529101 | 0.054187192 | 6.64E-10 |
| **Big Horn [4]** | 132 | 0.57707637 | 0 | 0.100529101 | 0.054187192 | 6.64E-10 |
| **Big Horn [4]** | 133 | 0.57707637 | 0 | 0.100529101 | 0.054187192 | 6.64E-10 |
| **Big Horn [4]** | 134 | 0.57707637 | 0 | 0.100529101 | 0.054187192 | 6.64E-10 |
| **Big Horn [4]** | 135 | 0.57707637 | 0 | 0.100529101 | 0.054187192 | 6.64E-10 |
| **Big Horn [4]** | 136 | 0.57707637 | 0 | 0.100529101 | 0.054187192 | 6.64E-10 |
| **Big Horn [4]** | 137 | 0.57707637 | 0 | 0.100529101 | 0.054187192 | 6.64E-10 |
| **Big Horn [4]** | 138 | 0.57707637 | 0 | 0.100529101 | 0.054187192 | 6.64E-10 |
| **Big Horn [4]** | 139 | 0.57707637 | 0 | 0.100529101 | 0.054187192 | 6.64E-10 |
| **Big Horn [4]** | 140 | 0.57707637 | 0 | 0.100529101 | 0.054187192 | 6.64E-10 |
| **Big Horn [4]** | 141 | 0.57707637 | 0 | 0.100529101 | 0.054187192 | 6.64E-10 |
| **Big Horn [4]** | 142 | 0.57707637 | 0 | 0.100529101 | 0.054187192 | 6.64E-10 |
| **Big Horn [4]** | 143 | 0.57707637 | 0 | 0.100529101 | 0.054187192 | 6.64E-10 |
| **Big Horn [4]** | 144 | 0.57707637 | 0 | 0.100529101 | 0.054187192 | 6.64E-10 |
| **Biglow Canyon (phase I 2008) [5]** | 3 | 0.62314555 | 1 | 0.0574452 | 0.014778325 | 0.000529016 |
| **Biglow Canyon (phase I 2008) [5]** | 4 | 0.62314555 | 1 | 0.0574452 | 0.014778325 | 0.000529016 |
| **Biglow Canyon (phase I 2008) [5]** | 5 | 0.62314555 | 1 | 0.0574452 | 0.014778325 | 0.000529016 |
| **Biglow Canyon (phase I 2008) [5]** | 6 | 0.62314555 | 1 | 0.0574452 | 0.014778325 | 0.000529016 |
| **Biglow Canyon (phase I 2008) [5]** | 7 | 0.62314555 | 1 | 0.0574452 | 0.014778325 | 0.000529016 |
| **Biglow Canyon (phase I 2008) [5]** | 8 | 0.62314555 | 1 | 0.0574452 | 0.014778325 | 0.000529016 |
| **Biglow Canyon (phase I 2008) [5]** | 9 | 0.62314555 | 1 | 0.0574452 | 0.014778325 | 0.000529016 |
| **Biglow Canyon (phase I 2008) [5]** | 10 | 0.62314555 | 1 | 0.0574452 | 0.014778325 | 0.000529016 |
| **Biglow Canyon (phase I 2008) [5]** | 11 | 0.62314555 | 1 | 0.0574452 | 0.014778325 | 0.000529016 |
| **Biglow Canyon (phase I 2008) [5]** | 12 | 0.62314555 | 1 | 0.0574452 | 0.014778325 | 0.000529016 |
| **Biglow Canyon (phase I 2008) [5]** | 13 | 0.62314555 | 1 | 0.0574452 | 0.014778325 | 0.000529016 |
| **Biglow Canyon (phase I 2008) [5]** | 14 | 0.62314555 | 1 | 0.0574452 | 0.014778325 | 0.000529016 |
| **Biglow Canyon (phase I 2008) [5]** | 15 | 0.62314555 | 1 | 0.0574452 | 0.014778325 | 0.000529016 |
| **Biglow Canyon (phase I 2008) [5]** | 16 | 0.62314555 | 1 | 0.0574452 | 0.014778325 | 0.000529016 |
| **Biglow Canyon (phase I 2008) [5]** | 17 | 0.62314555 | 1 | 0.0574452 | 0.014778325 | 0.000529016 |
| **Biglow Canyon (phase I 2008) [5]** | 18 | 0.62314555 | 1 | 0.0574452 | 0.014778325 | 0.000529016 |
| **Biglow Canyon (phase I 2008) [5]** | 19 | 0.62314555 | 1 | 0.0574452 | 0.014778325 | 0.000529016 |
| **Biglow Canyon (phase I 2008) [5]** | 20 | 0.62314555 | 1 | 0.0574452 | 0.014778325 | 0.000529016 |
| **Biglow Canyon (phase I 2008) [5]** | 21 | 0.62314555 | 1 | 0.0574452 | 0.014778325 | 0.000529016 |
| **Biglow Canyon (phase I 2008) [5]** | 22 | 0.62314555 | 1 | 0.0574452 | 0.014778325 | 0.000529016 |
| **Biglow Canyon (phase I 2008) [5]** | 23 | 0.62314555 | 1 | 0.0574452 | 0.014778325 | 0.000529016 |
| **Biglow Canyon (phase I 2008) [5]** | 24 | 0.62314555 | 1 | 0.0574452 | 0.014778325 | 0.000529016 |
| **Biglow Canyon (phase I 2008) [5]** | 25 | 0.62314555 | 1 | 0.0574452 | 0.014778325 | 0.000529016 |
| **Biglow Canyon (phase I 2008) [5]** | 26 | 0.62314555 | 1 | 0.0574452 | 0.014778325 | 0.000529016 |
| **Biglow Canyon (phase I 2008) [5]** | 27 | 0.62314555 | 1 | 0.0574452 | 0.014778325 | 0.000529016 |
| **Biglow Canyon (phase I 2008) [5]** | 28 | 0.62314555 | 1 | 0.0574452 | 0.014778325 | 0.000529016 |
| **Biglow Canyon (phase I 2008) [5]** | 29 | 0.62314555 | 1 | 0.0574452 | 0.014778325 | 0.000529016 |
| **Biglow Canyon (phase I 2008) [5]** | 30 | 0.62314555 | 1 | 0.0574452 | 0.014778325 | 0.000529016 |
| **Biglow Canyon (phase I 2008) [5]** | 31 | 0.62314555 | 1 | 0.0574452 | 0.014778325 | 0.000529016 |
| **Biglow Canyon (phase I 2008) [5]** | 32 | 0.62314555 | 1 | 0.0574452 | 0.014778325 | 0.000529016 |
| **Biglow Canyon (phase I 2008) [5]** | 33 | 0.62314555 | 1 | 0.0574452 | 0.014778325 | 0.000529016 |
| **Biglow Canyon (phase I 2008) [5]** | 34 | 0.62314555 | 1 | 0.0574452 | 0.014778325 | 0.000529016 |
| **Biglow Canyon (phase I 2008) [5]** | 35 | 0.62314555 | 1 | 0.0574452 | 0.014778325 | 0.000529016 |
| **Biglow Canyon (phase I 2008) [5]** | 36 | 0.62314555 | 1 | 0.0574452 | 0.014778325 | 0.000529016 |
| **Biglow Canyon (phase I 2008) [5]** | 37 | 0.62314555 | 1 | 0.0574452 | 0.014778325 | 0.000529016 |
| **Biglow Canyon (phase I 2008) [5]** | 38 | 0.62314555 | 1 | 0.0574452 | 0.014778325 | 0.000529016 |
| **Biglow Canyon (phase I 2008) [5]** | 39 | 0.62314555 | 1 | 0.0574452 | 0.014778325 | 0.000529016 |
| **Biglow Canyon (phase I 2008) [5]** | 40 | 0.62314555 | 1 | 0.0574452 | 0.014778325 | 0.000529016 |
| **Biglow Canyon (phase I 2008) [5]** | 41 | 0.62314555 | 1 | 0.0574452 | 0.014778325 | 0.000529016 |
| **Biglow Canyon (phase I 2008) [5]** | 42 | 0.62314555 | 1 | 0.0574452 | 0.014778325 | 0.000529016 |
| **Biglow Canyon (phase I 2008) [5]** | 43 | 0.62314555 | 1 | 0.0574452 | 0.014778325 | 0.000529016 |
| **Biglow Canyon (phase I 2008) [5]** | 44 | 0.62314555 | 1 | 0.0574452 | 0.014778325 | 0.000529016 |
| **Biglow Canyon (phase I 2008) [5]** | 45 | 0.62314555 | 1 | 0.0574452 | 0.014778325 | 0.000529016 |
| **Biglow Canyon (phase I 2008) [5]** | 46 | 0.62314555 | 1 | 0.0574452 | 0.014778325 | 0.000529016 |
| **Biglow Canyon (phase I 2008) [5]** | 47 | 0.62314555 | 1 | 0.0574452 | 0.014778325 | 0.000529016 |
| **Biglow Canyon (phase I 2008) [5]** | 48 | 0.62314555 | 1 | 0.0574452 | 0.014778325 | 0.000529016 |
| **Biglow Canyon (phase I 2008) [5]** | 49 | 0.62314555 | 1 | 0.0574452 | 0.014778325 | 0.000529016 |
| **Biglow Canyon (phase I 2008) [5]** | 50 | 0.62314555 | 1 | 0.0574452 | 0.014778325 | 0.000529016 |
| **Biglow Canyon (phase I 2008) [5]** | 51 | 0.62314555 | 1 | 0.0574452 | 0.014778325 | 0.000529016 |
| **Biglow Canyon (phase I 2008) [5]** | 52 | 0.62314555 | 1 | 0.0574452 | 0.014778325 | 0.000529016 |
| **Biglow Canyon (phase I 2008) [5]** | 53 | 0.62314555 | 1 | 0.0574452 | 0.014778325 | 0.000529016 |
| **Biglow Canyon (phase I 2008) [5]** | 54 | 0.62314555 | 1 | 0.0574452 | 0.014778325 | 0.000529016 |
| **Biglow Canyon (phase I 2008) [5]** | 55 | 0.62314555 | 1 | 0.0574452 | 0.014778325 | 0.000529016 |
| **Biglow Canyon (phase I 2008) [5]** | 56 | 0.62314555 | 1 | 0.0574452 | 0.014778325 | 0.000529016 |
| **Biglow Canyon (phase I 2008) [5]** | 57 | 0.62314555 | 1 | 0.0574452 | 0.014778325 | 0.000529016 |
| **Biglow Canyon (phase I 2008) [5]** | 58 | 0.62314555 | 0 | 0.0574452 | 0.014778325 | 6.64E-10 |
| **Biglow Canyon (phase I 2008) [5]** | 59 | 0.62314555 | 0 | 0.0574452 | 0.014778325 | 6.64E-10 |
| **Biglow Canyon (phase I 2008) [5]** | 60 | 0.62314555 | 0 | 0.0574452 | 0.014778325 | 6.64E-10 |
| **Biglow Canyon (phase I 2008) [5]** | 61 | 0.62314555 | 0 | 0.0574452 | 0.014778325 | 6.64E-10 |
| **Biglow Canyon (phase I 2008) [5]** | 62 | 0.62314555 | 0 | 0.0574452 | 0.014778325 | 6.64E-10 |
| **Biglow Canyon (phase I 2008) [5]** | 63 | 0.62314555 | 0 | 0.0574452 | 0.014778325 | 6.64E-10 |
| **Biglow Canyon (phase I 2008) [5]** | 64 | 0.62314555 | 0 | 0.0574452 | 0.014778325 | 6.64E-10 |
| **Biglow Canyon (phase I 2008) [5]** | 65 | 0.62314555 | 0 | 0.0574452 | 0.014778325 | 6.64E-10 |
| **Biglow Canyon (phase I 2008) [5]** | 66 | 0.62314555 | 0 | 0.0574452 | 0.014778325 | 6.64E-10 |
| **Biglow Canyon (phase I 2008) [5]** | 67 | 0.62314555 | 0 | 0.0574452 | 0.014778325 | 6.64E-10 |
| **Biglow Canyon (phase I 2008) [5]** | 68 | 0.62314555 | 0 | 0.0574452 | 0.014778325 | 6.64E-10 |
| **Biglow Canyon (phase I 2008) [5]** | 69 | 0.62314555 | 0 | 0.0574452 | 0.014778325 | 6.64E-10 |
| **Biglow Canyon (phase I 2008) [5]** | 70 | 0.62314555 | 0 | 0.0574452 | 0.014778325 | 6.64E-10 |
| **Biglow Canyon (phase I 2008) [5]** | 71 | 0.62314555 | 0 | 0.0574452 | 0.014778325 | 6.64E-10 |
| **Biglow Canyon (phase I 2008) [5]** | 72 | 0.62314555 | 0 | 0.0574452 | 0.014778325 | 6.64E-10 |
| **Biglow Canyon (phase I 2008) [5]** | 73 | 0.62314555 | 0 | 0.0574452 | 0.014778325 | 6.64E-10 |
| **Biglow Canyon (phase I 2008) [5]** | 74 | 0.62314555 | 0 | 0.0574452 | 0.014778325 | 6.64E-10 |
| **Biglow Canyon (phase I 2008) [5]** | 75 | 0.62314555 | 0 | 0.0574452 | 0.014778325 | 6.64E-10 |
| **Biglow Canyon (phase I 2008) [5]** | 76 | 0.62314555 | 0 | 0.0574452 | 0.014778325 | 6.64E-10 |
| **Biglow Canyon (phase I 2008) [5]** | 77 | 0.62314555 | 0 | 0.0574452 | 0.014778325 | 6.64E-10 |
| **Biglow Canyon (phase I 2008) [5]** | 78 | 0.62314555 | 0 | 0.0574452 | 0.014778325 | 6.64E-10 |
| **Biglow Canyon (phase I 2008) [5]** | 79 | 0.62314555 | 0 | 0.0574452 | 0.014778325 | 6.64E-10 |
| **Biglow Canyon (phase I 2008) [5]** | 80 | 0.62314555 | 0 | 0.0574452 | 0.014778325 | 6.64E-10 |
| **Biglow Canyon (phase I 2008) [5]** | 81 | 0.62314555 | 0 | 0.0574452 | 0.014778325 | 6.64E-10 |
| **Biglow Canyon (phase I 2008) [5]** | 82 | 0.62314555 | 0 | 0.0574452 | 0.014778325 | 6.64E-10 |
| **Biglow Canyon (phase I 2008) [5]** | 83 | 0.62314555 | 0 | 0.0574452 | 0.014778325 | 6.64E-10 |
| **Biglow Canyon (phase I 2008) [5]** | 84 | 0.62314555 | 0 | 0.0574452 | 0.014778325 | 6.64E-10 |
| **Biglow Canyon (phase I 2008) [5]** | 85 | 0.62314555 | 0 | 0.0574452 | 0.014778325 | 6.64E-10 |
| **Biglow Canyon (phase I 2008) [5]** | 86 | 0.62314555 | 0 | 0.0574452 | 0.014778325 | 6.64E-10 |
| **Biglow Canyon (phase I 2008) [5]** | 87 | 0.62314555 | 0 | 0.0574452 | 0.014778325 | 6.64E-10 |
| **Biglow Canyon (phase I 2008) [5]** | 88 | 0.62314555 | 0 | 0.0574452 | 0.014778325 | 6.64E-10 |
| **Biglow Canyon (phase I 2008) [5]** | 89 | 0.62314555 | 0 | 0.0574452 | 0.014778325 | 6.64E-10 |
| **Biglow Canyon (phase I 2008) [5]** | 90 | 0.62314555 | 0 | 0.0574452 | 0.014778325 | 6.64E-10 |
| **Biglow Canyon (phase I 2008) [5]** | 91 | 0.62314555 | 0 | 0.0574452 | 0.014778325 | 6.64E-10 |
| **Biglow Canyon (phase I 2008) [5]** | 92 | 0.62314555 | 0 | 0.0574452 | 0.014778325 | 6.64E-10 |
| **Biglow Canyon (phase I 2008) [5]** | 93 | 0.62314555 | 0 | 0.0574452 | 0.014778325 | 6.64E-10 |
| **Biglow Canyon (phase I 2008) [5]** | 94 | 0.62314555 | 0 | 0.0574452 | 0.014778325 | 6.64E-10 |
| **Biglow Canyon (phase I 2008) [5]** | 95 | 0.62314555 | 0 | 0.0574452 | 0.014778325 | 6.64E-10 |
| **Biglow Canyon (phase I 2008) [5]** | 96 | 0.62314555 | 0 | 0.0574452 | 0.014778325 | 6.64E-10 |
| **Biglow Canyon (phase I 2008) [5]** | 97 | 0.62314555 | 0 | 0.0574452 | 0.014778325 | 6.64E-10 |
| **Biglow Canyon (phase I 2008) [5]** | 98 | 0.62314555 | 0 | 0.0574452 | 0.014778325 | 6.64E-10 |
| **Biglow Canyon (phase I 2008) [5]** | 99 | 0.62314555 | 0 | 0.0574452 | 0.014778325 | 6.64E-10 |
| **Biglow Canyon (phase I 2008) [5]** | 100 | 0.62314555 | 0 | 0.0574452 | 0.014778325 | 6.64E-10 |
| **Biglow Canyon (phase I 2008) [5]** | 101 | 0.62314555 | 0 | 0.0574452 | 0.014778325 | 6.64E-10 |
| **Biglow Canyon (phase I 2008) [5]** | 102 | 0.62314555 | 0 | 0.0574452 | 0.014778325 | 6.64E-10 |
| **Biglow Canyon (phase I 2008) [5]** | 103 | 0.62314555 | 0 | 0.0574452 | 0.014778325 | 6.64E-10 |
| **Biglow Canyon (phase I 2008) [5]** | 104 | 0.62314555 | 0 | 0.0574452 | 0.014778325 | 6.64E-10 |
| **Biglow Canyon (phase I 2008) [5]** | 105 | 0.62314555 | 0 | 0.0574452 | 0.014778325 | 6.64E-10 |
| **Biglow Canyon (phase I 2008) [5]** | 106 | 0.62314555 | 0 | 0.0574452 | 0.014778325 | 6.64E-10 |
| **Biglow Canyon (phase I 2008) [5]** | 107 | 0.62314555 | 0 | 0.0574452 | 0.014778325 | 6.64E-10 |
| **Biglow Canyon (phase I 2008) [5]** | 108 | 0.62314555 | 0 | 0.0574452 | 0.014778325 | 6.64E-10 |
| **Biglow Canyon (phase I 2008) [5]** | 109 | 0.62314555 | 0 | 0.0574452 | 0.014778325 | 6.64E-10 |
| **Biglow Canyon (phase I 2008) [5]** | 110 | 0.62314555 | 0 | 0.0574452 | 0.014778325 | 6.64E-10 |
| **Biglow Canyon (phase I 2008) [5]** | 111 | 0.62314555 | 0 | 0.0574452 | 0.014778325 | 6.64E-10 |
| **Biglow Canyon (phase I 2008) [5]** | 112 | 0.62314555 | 0 | 0.0574452 | 0.014778325 | 6.64E-10 |
| **Biglow Canyon (phase I 2008) [5]** | 113 | 0.62314555 | 0 | 0.0574452 | 0.014778325 | 6.64E-10 |
| **Biglow Canyon (phase I 2008) [5]** | 114 | 0.62314555 | 0 | 0.0574452 | 0.014778325 | 6.64E-10 |
| **Biglow Canyon (phase I 2008) [5]** | 115 | 0.62314555 | 0 | 0.0574452 | 0.014778325 | 6.64E-10 |
| **Biglow Canyon (phase I 2008) [5]** | 116 | 0.62314555 | 0 | 0.0574452 | 0.014778325 | 6.64E-10 |
| **Biglow Canyon (phase I 2008) [5]** | 117 | 0.62314555 | 0 | 0.0574452 | 0.014778325 | 6.64E-10 |
| **Biglow Canyon (phase I 2008) [5]** | 118 | 0.62314555 | 0 | 0.0574452 | 0.014778325 | 6.64E-10 |
| **Biglow Canyon (phase I 2008) [5]** | 119 | 0.62314555 | 0 | 0.0574452 | 0.014778325 | 6.64E-10 |
| **Biglow Canyon (phase I 2008) [5]** | 120 | 0.62314555 | 0 | 0.0574452 | 0.014778325 | 6.64E-10 |
| **Biglow Canyon (phase I 2008) [5]** | 121 | 0.62314555 | 0 | 0.0574452 | 0.014778325 | 6.64E-10 |
| **Biglow Canyon (phase I 2008) [5]** | 122 | 0.62314555 | 0 | 0.0574452 | 0.014778325 | 6.64E-10 |
| **Biglow Canyon (phase I 2008) [5]** | 123 | 0.62314555 | 0 | 0.0574452 | 0.014778325 | 6.64E-10 |
| **Biglow Canyon (phase I 2008) [5]** | 124 | 0.62314555 | 0 | 0.0574452 | 0.014778325 | 6.64E-10 |
| **Biglow Canyon (phase I 2008) [5]** | 125 | 0.62314555 | 0 | 0.0574452 | 0.014778325 | 6.64E-10 |
| **Biglow Canyon (phase I 2008) [5]** | 126 | 0.62314555 | 0 | 0.0574452 | 0.014778325 | 6.64E-10 |
| **Biglow Canyon (phase I 2008) [5]** | 127 | 0.62314555 | 0 | 0.0574452 | 0.014778325 | 6.64E-10 |
| **Biglow Canyon (phase I 2008) [5]** | 128 | 0.62314555 | 0 | 0.0574452 | 0.014778325 | 6.64E-10 |
| **Biglow Canyon (phase I 2008) [5]** | 129 | 0.62314555 | 0 | 0.0574452 | 0.014778325 | 6.64E-10 |
| **Biglow Canyon (phase I 2008) [5]** | 130 | 0.62314555 | 0 | 0.0574452 | 0.014778325 | 6.64E-10 |
| **Biglow Canyon (phase I 2008) [5]** | 131 | 0.62314555 | 0 | 0.0574452 | 0.014778325 | 6.64E-10 |
| **Biglow Canyon (phase I 2008) [5]** | 132 | 0.62314555 | 0 | 0.0574452 | 0.014778325 | 6.64E-10 |
| **Biglow Canyon (phase I 2008) [5]** | 133 | 0.62314555 | 0 | 0.0574452 | 0.014778325 | 6.64E-10 |
| **Biglow Canyon (phase I 2008) [5]** | 134 | 0.62314555 | 0 | 0.0574452 | 0.014778325 | 6.64E-10 |
| **Biglow Canyon (phase I 2008) [5]** | 135 | 0.62314555 | 0 | 0.0574452 | 0.014778325 | 6.64E-10 |
| **Biglow Canyon (phase I 2008) [5]** | 136 | 0.62314555 | 0 | 0.0574452 | 0.014778325 | 6.64E-10 |
| **Biglow Canyon (phase I 2008) [5]** | 137 | 0.62314555 | 0 | 0.0574452 | 0.014778325 | 6.64E-10 |
| **Biglow Canyon (phase I 2008) [5]** | 138 | 0.62314555 | 0 | 0.0574452 | 0.014778325 | 6.64E-10 |
| **Biglow Canyon (phase I 2008) [5]** | 139 | 0.62314555 | 0 | 0.0574452 | 0.014778325 | 6.64E-10 |
| **Biglow Canyon (phase I 2008) [5]** | 140 | 0.62314555 | 0 | 0.0574452 | 0.014778325 | 6.64E-10 |
| **Biglow Canyon (phase I 2008) [5]** | 141 | 0.62314555 | 0 | 0.0574452 | 0.014778325 | 6.64E-10 |
| **Biglow Canyon (phase I 2008) [5]** | 142 | 0.62314555 | 0 | 0.0574452 | 0.014778325 | 6.64E-10 |
| **Biglow Canyon (phase I 2008) [5]** | 143 | 0.62314555 | 0 | 0.0574452 | 0.014778325 | 6.64E-10 |
| **Biglow Canyon (phase I 2008) [5]** | 144 | 0.62314555 | 0 | 0.0574452 | 0.014778325 | 6.64E-10 |
| **Biglow Canyon (phase III; 2010/2011) [6]** | 3 | 0.55746877 | 1 | 0.0574452 | 0.024630542 | 0.000788766 |
| **Biglow Canyon (phase III; 2010/2011) [6]** | 4 | 0.55746877 | 1 | 0.0574452 | 0.024630542 | 0.000788766 |
| **Biglow Canyon (phase III; 2010/2011) [6]** | 5 | 0.55746877 | 1 | 0.0574452 | 0.024630542 | 0.000788766 |
| **Biglow Canyon (phase III; 2010/2011) [6]** | 6 | 0.55746877 | 1 | 0.0574452 | 0.024630542 | 0.000788766 |
| **Biglow Canyon (phase III; 2010/2011) [6]** | 7 | 0.55746877 | 1 | 0.0574452 | 0.024630542 | 0.000788766 |
| **Biglow Canyon (phase III; 2010/2011) [6]** | 8 | 0.55746877 | 1 | 0.0574452 | 0.024630542 | 0.000788766 |
| **Biglow Canyon (phase III; 2010/2011) [6]** | 9 | 0.55746877 | 1 | 0.0574452 | 0.024630542 | 0.000788766 |
| **Biglow Canyon (phase III; 2010/2011) [6]** | 10 | 0.55746877 | 1 | 0.0574452 | 0.024630542 | 0.000788766 |
| **Biglow Canyon (phase III; 2010/2011) [6]** | 11 | 0.55746877 | 1 | 0.0574452 | 0.024630542 | 0.000788766 |
| **Biglow Canyon (phase III; 2010/2011) [6]** | 12 | 0.55746877 | 1 | 0.0574452 | 0.024630542 | 0.000788766 |
| **Biglow Canyon (phase III; 2010/2011) [6]** | 13 | 0.55746877 | 1 | 0.0574452 | 0.024630542 | 0.000788766 |
| **Biglow Canyon (phase III; 2010/2011) [6]** | 14 | 0.55746877 | 1 | 0.0574452 | 0.024630542 | 0.000788766 |
| **Biglow Canyon (phase III; 2010/2011) [6]** | 15 | 0.55746877 | 1 | 0.0574452 | 0.024630542 | 0.000788766 |
| **Biglow Canyon (phase III; 2010/2011) [6]** | 16 | 0.55746877 | 1 | 0.0574452 | 0.024630542 | 0.000788766 |
| **Biglow Canyon (phase III; 2010/2011) [6]** | 17 | 0.55746877 | 1 | 0.0574452 | 0.024630542 | 0.000788766 |
| **Biglow Canyon (phase III; 2010/2011) [6]** | 18 | 0.55746877 | 1 | 0.0574452 | 0.024630542 | 0.000788766 |
| **Biglow Canyon (phase III; 2010/2011) [6]** | 19 | 0.55746877 | 1 | 0.0574452 | 0.024630542 | 0.000788766 |
| **Biglow Canyon (phase III; 2010/2011) [6]** | 20 | 0.55746877 | 1 | 0.0574452 | 0.024630542 | 0.000788766 |
| **Biglow Canyon (phase III; 2010/2011) [6]** | 21 | 0.55746877 | 1 | 0.0574452 | 0.024630542 | 0.000788766 |
| **Biglow Canyon (phase III; 2010/2011) [6]** | 22 | 0.55746877 | 1 | 0.0574452 | 0.024630542 | 0.000788766 |
| **Biglow Canyon (phase III; 2010/2011) [6]** | 23 | 0.55746877 | 1 | 0.0574452 | 0.024630542 | 0.000788766 |
| **Biglow Canyon (phase III; 2010/2011) [6]** | 24 | 0.55746877 | 1 | 0.0574452 | 0.024630542 | 0.000788766 |
| **Biglow Canyon (phase III; 2010/2011) [6]** | 25 | 0.55746877 | 1 | 0.0574452 | 0.024630542 | 0.000788766 |
| **Biglow Canyon (phase III; 2010/2011) [6]** | 26 | 0.55746877 | 1 | 0.0574452 | 0.024630542 | 0.000788766 |
| **Biglow Canyon (phase III; 2010/2011) [6]** | 27 | 0.55746877 | 1 | 0.0574452 | 0.024630542 | 0.000788766 |
| **Biglow Canyon (phase III; 2010/2011) [6]** | 28 | 0.55746877 | 1 | 0.0574452 | 0.024630542 | 0.000788766 |
| **Biglow Canyon (phase III; 2010/2011) [6]** | 29 | 0.55746877 | 1 | 0.0574452 | 0.024630542 | 0.000788766 |
| **Biglow Canyon (phase III; 2010/2011) [6]** | 30 | 0.55746877 | 1 | 0.0574452 | 0.024630542 | 0.000788766 |
| **Biglow Canyon (phase III; 2010/2011) [6]** | 31 | 0.55746877 | 1 | 0.0574452 | 0.024630542 | 0.000788766 |
| **Biglow Canyon (phase III; 2010/2011) [6]** | 32 | 0.55746877 | 1 | 0.0574452 | 0.024630542 | 0.000788766 |
| **Biglow Canyon (phase III; 2010/2011) [6]** | 33 | 0.55746877 | 1 | 0.0574452 | 0.024630542 | 0.000788766 |
| **Biglow Canyon (phase III; 2010/2011) [6]** | 34 | 0.55746877 | 1 | 0.0574452 | 0.024630542 | 0.000788766 |
| **Biglow Canyon (phase III; 2010/2011) [6]** | 35 | 0.55746877 | 1 | 0.0574452 | 0.024630542 | 0.000788766 |
| **Biglow Canyon (phase III; 2010/2011) [6]** | 36 | 0.55746877 | 1 | 0.0574452 | 0.024630542 | 0.000788766 |
| **Biglow Canyon (phase III; 2010/2011) [6]** | 37 | 0.55746877 | 1 | 0.0574452 | 0.024630542 | 0.000788766 |
| **Biglow Canyon (phase III; 2010/2011) [6]** | 38 | 0.55746877 | 1 | 0.0574452 | 0.024630542 | 0.000788766 |
| **Biglow Canyon (phase III; 2010/2011) [6]** | 39 | 0.55746877 | 1 | 0.0574452 | 0.024630542 | 0.000788766 |
| **Biglow Canyon (phase III; 2010/2011) [6]** | 40 | 0.55746877 | 1 | 0.0574452 | 0.024630542 | 0.000788766 |
| **Biglow Canyon (phase III; 2010/2011) [6]** | 41 | 0.55746877 | 1 | 0.0574452 | 0.024630542 | 0.000788766 |
| **Biglow Canyon (phase III; 2010/2011) [6]** | 42 | 0.55746877 | 1 | 0.0574452 | 0.024630542 | 0.000788766 |
| **Biglow Canyon (phase III; 2010/2011) [6]** | 43 | 0.55746877 | 1 | 0.0574452 | 0.024630542 | 0.000788766 |
| **Biglow Canyon (phase III; 2010/2011) [6]** | 44 | 0.55746877 | 1 | 0.0574452 | 0.024630542 | 0.000788766 |
| **Biglow Canyon (phase III; 2010/2011) [6]** | 45 | 0.55746877 | 1 | 0.0574452 | 0.024630542 | 0.000788766 |
| **Biglow Canyon (phase III; 2010/2011) [6]** | 46 | 0.55746877 | 1 | 0.0574452 | 0.024630542 | 0.000788766 |
| **Biglow Canyon (phase III; 2010/2011) [6]** | 47 | 0.55746877 | 1 | 0.0574452 | 0.024630542 | 0.000788766 |
| **Biglow Canyon (phase III; 2010/2011) [6]** | 48 | 0.55746877 | 1 | 0.0574452 | 0.024630542 | 0.000788766 |
| **Biglow Canyon (phase III; 2010/2011) [6]** | 49 | 0.55746877 | 1 | 0.0574452 | 0.024630542 | 0.000788766 |
| **Biglow Canyon (phase III; 2010/2011) [6]** | 50 | 0.55746877 | 1 | 0.0574452 | 0.024630542 | 0.000788766 |
| **Biglow Canyon (phase III; 2010/2011) [6]** | 51 | 0.55746877 | 1 | 0.0574452 | 0.024630542 | 0.000788766 |
| **Biglow Canyon (phase III; 2010/2011) [6]** | 52 | 0.55746877 | 1 | 0.0574452 | 0.024630542 | 0.000788766 |
| **Biglow Canyon (phase III; 2010/2011) [6]** | 53 | 0.55746877 | 1 | 0.0574452 | 0.024630542 | 0.000788766 |
| **Biglow Canyon (phase III; 2010/2011) [6]** | 54 | 0.55746877 | 1 | 0.0574452 | 0.024630542 | 0.000788766 |
| **Biglow Canyon (phase III; 2010/2011) [6]** | 55 | 0.55746877 | 1 | 0.0574452 | 0.024630542 | 0.000788766 |
| **Biglow Canyon (phase III; 2010/2011) [6]** | 56 | 0.55746877 | 1 | 0.0574452 | 0.024630542 | 0.000788766 |
| **Biglow Canyon (phase III; 2010/2011) [6]** | 57 | 0.55746877 | 1 | 0.0574452 | 0.024630542 | 0.000788766 |
| **Biglow Canyon (phase III; 2010/2011) [6]** | 58 | 0.55746877 | 1 | 0.0574452 | 0.024630542 | 0.000788766 |
| **Biglow Canyon (phase III; 2010/2011) [6]** | 59 | 0.55746877 | 1 | 0.0574452 | 0.024630542 | 0.000788766 |
| **Biglow Canyon (phase III; 2010/2011) [6]** | 60 | 0.55746877 | 1 | 0.0574452 | 0.024630542 | 0.000788766 |
| **Biglow Canyon (phase III; 2010/2011) [6]** | 61 | 0.55746877 | 1 | 0.0574452 | 0.024630542 | 0.000788766 |
| **Biglow Canyon (phase III; 2010/2011) [6]** | 62 | 0.55746877 | 1 | 0.0574452 | 0.024630542 | 0.000788766 |
| **Biglow Canyon (phase III; 2010/2011) [6]** | 63 | 0.55746877 | 1 | 0.0574452 | 0.024630542 | 0.000788766 |
| **Biglow Canyon (phase III; 2010/2011) [6]** | 64 | 0.55746877 | 1 | 0.0574452 | 0.024630542 | 0.000788766 |
| **Biglow Canyon (phase III; 2010/2011) [6]** | 65 | 0.55746877 | 1 | 0.0574452 | 0.024630542 | 0.000788766 |
| **Biglow Canyon (phase III; 2010/2011) [6]** | 66 | 0.55746877 | 1 | 0.0574452 | 0.024630542 | 0.000788766 |
| **Biglow Canyon (phase III; 2010/2011) [6]** | 67 | 0.55746877 | 1 | 0.0574452 | 0.024630542 | 0.000788766 |
| **Biglow Canyon (phase III; 2010/2011) [6]** | 68 | 0.55746877 | 1 | 0.0574452 | 0.024630542 | 0.000788766 |
| **Biglow Canyon (phase III; 2010/2011) [6]** | 69 | 0.55746877 | 1 | 0.0574452 | 0.024630542 | 0.000788766 |
| **Biglow Canyon (phase III; 2010/2011) [6]** | 70 | 0.55746877 | 1 | 0.0574452 | 0.024630542 | 0.000788766 |
| **Biglow Canyon (phase III; 2010/2011) [6]** | 71 | 0.55746877 | 1 | 0.0574452 | 0.024630542 | 0.000788766 |
| **Biglow Canyon (phase III; 2010/2011) [6]** | 72 | 0.55746877 | 1 | 0.0574452 | 0.024630542 | 0.000788766 |
| **Biglow Canyon (phase III; 2010/2011) [6]** | 73 | 0.55746877 | 1 | 0.0574452 | 0.024630542 | 0.000788766 |
| **Biglow Canyon (phase III; 2010/2011) [6]** | 74 | 0.55746877 | 1 | 0.0574452 | 0.024630542 | 0.000788766 |
| **Biglow Canyon (phase III; 2010/2011) [6]** | 75 | 0.55746877 | 1 | 0.0574452 | 0.024630542 | 0.000788766 |
| **Biglow Canyon (phase III; 2010/2011) [6]** | 76 | 0.55746877 | 1 | 0.0574452 | 0.024630542 | 0.000788766 |
| **Biglow Canyon (phase III; 2010/2011) [6]** | 77 | 0.55746877 | 1 | 0.0574452 | 0.024630542 | 0.000788766 |
| **Biglow Canyon (phase III; 2010/2011) [6]** | 78 | 0.55746877 | 1 | 0.0574452 | 0.024630542 | 0.000788766 |
| **Biglow Canyon (phase III; 2010/2011) [6]** | 79 | 0.55746877 | 1 | 0.0574452 | 0.024630542 | 0.000788766 |
| **Biglow Canyon (phase III; 2010/2011) [6]** | 80 | 0.55746877 | 1 | 0.0574452 | 0.024630542 | 0.000788766 |
| **Biglow Canyon (phase III; 2010/2011) [6]** | 81 | 0.55746877 | 1 | 0.0574452 | 0.024630542 | 0.000788766 |
| **Biglow Canyon (phase III; 2010/2011) [6]** | 82 | 0.55746877 | 1 | 0.0574452 | 0.024630542 | 0.000788766 |
| **Biglow Canyon (phase III; 2010/2011) [6]** | 83 | 0.55746877 | 1 | 0.0574452 | 0.024630542 | 0.000788766 |
| **Biglow Canyon (phase III; 2010/2011) [6]** | 84 | 0.55746877 | 1 | 0.0574452 | 0.024630542 | 0.000788766 |
| **Biglow Canyon (phase III; 2010/2011) [6]** | 85 | 0.55746877 | 1 | 0.0574452 | 0.024630542 | 0.000788766 |
| **Biglow Canyon (phase III; 2010/2011) [6]** | 86 | 0.55746877 | 1 | 0.0574452 | 0.024630542 | 0.000788766 |
| **Biglow Canyon (phase III; 2010/2011) [6]** | 87 | 0.55746877 | 1 | 0.0574452 | 0.024630542 | 0.000788766 |
| **Biglow Canyon (phase III; 2010/2011) [6]** | 88 | 0.55746877 | 1 | 0.0574452 | 0.024630542 | 0.000788766 |
| **Biglow Canyon (phase III; 2010/2011) [6]** | 89 | 0.55746877 | 1 | 0.0574452 | 0.024630542 | 0.000788766 |
| **Biglow Canyon (phase III; 2010/2011) [6]** | 90 | 0.55746877 | 1 | 0.0574452 | 0.024630542 | 0.000788766 |
| **Biglow Canyon (phase III; 2010/2011) [6]** | 91 | 0.55746877 | 1 | 0.0574452 | 0.024630542 | 0.000788766 |
| **Biglow Canyon (phase III; 2010/2011) [6]** | 92 | 0.55746877 | 1 | 0.0574452 | 0.024630542 | 0.000788766 |
| **Biglow Canyon (phase III; 2010/2011) [6]** | 93 | 0.55746877 | 1 | 0.0574452 | 0.024630542 | 0.000788766 |
| **Biglow Canyon (phase III; 2010/2011) [6]** | 94 | 0.55746877 | 1 | 0.0574452 | 0.024630542 | 0.000788766 |
| **Biglow Canyon (phase III; 2010/2011) [6]** | 95 | 0.55746877 | 1 | 0.0574452 | 0.024630542 | 0.000788766 |
| **Biglow Canyon (phase III; 2010/2011) [6]** | 96 | 0.55746877 | 1 | 0.0574452 | 0.024630542 | 0.000788766 |
| **Biglow Canyon (phase III; 2010/2011) [6]** | 97 | 0.55746877 | 1 | 0.0574452 | 0.024630542 | 0.000788766 |
| **Biglow Canyon (phase III; 2010/2011) [6]** | 98 | 0.55746877 | 1 | 0.0574452 | 0.024630542 | 0.000788766 |
| **Biglow Canyon (phase III; 2010/2011) [6]** | 99 | 0.55746877 | 1 | 0.0574452 | 0.024630542 | 0.000788766 |
| **Biglow Canyon (phase III; 2010/2011) [6]** | 100 | 0.55746877 | 1 | 0.0574452 | 0.024630542 | 0.000788766 |
| **Biglow Canyon (phase III; 2010/2011) [6]** | 101 | 0.55746877 | 1 | 0.0574452 | 0.024630542 | 0.000788766 |
| **Biglow Canyon (phase III; 2010/2011) [6]** | 102 | 0.55746877 | 1 | 0.0574452 | 0.024630542 | 0.000788766 |
| **Biglow Canyon (phase III; 2010/2011) [6]** | 103 | 0.55746877 | 1 | 0.0574452 | 0.024630542 | 0.000788766 |
| **Biglow Canyon (phase III; 2010/2011) [6]** | 104 | 0.55746877 | 1 | 0.0574452 | 0.024630542 | 0.000788766 |
| **Biglow Canyon (phase III; 2010/2011) [6]** | 105 | 0.55746877 | 1 | 0.0574452 | 0.024630542 | 0.000788766 |
| **Biglow Canyon (phase III; 2010/2011) [6]** | 106 | 0.55746877 | 1 | 0.0574452 | 0.024630542 | 0.000788766 |
| **Biglow Canyon (phase III; 2010/2011) [6]** | 107 | 0.55746877 | 1 | 0.0574452 | 0.024630542 | 0.000788766 |
| **Biglow Canyon (phase III; 2010/2011) [6]** | 108 | 0.55746877 | 1 | 0.0574452 | 0.024630542 | 0.000788766 |
| **Biglow Canyon (phase III; 2010/2011) [6]** | 109 | 0.55746877 | 1 | 0.0574452 | 0.024630542 | 0.000788766 |
| **Biglow Canyon (phase III; 2010/2011) [6]** | 110 | 0.55746877 | 1 | 0.0574452 | 0.024630542 | 0.000788766 |
| **Biglow Canyon (phase III; 2010/2011) [6]** | 111 | 0.55746877 | 1 | 0.0574452 | 0.024630542 | 0.000788766 |
| **Biglow Canyon (phase III; 2010/2011) [6]** | 112 | 0.55746877 | 1 | 0.0574452 | 0.024630542 | 0.000788766 |
| **Biglow Canyon (phase III; 2010/2011) [6]** | 113 | 0.55746877 | 1 | 0.0574452 | 0.024630542 | 0.000788766 |
| **Biglow Canyon (phase III; 2010/2011) [6]** | 114 | 0.55746877 | 1 | 0.0574452 | 0.024630542 | 0.000788766 |
| **Biglow Canyon (phase III; 2010/2011) [6]** | 115 | 0.55746877 | 1 | 0.0574452 | 0.024630542 | 0.000788766 |
| **Biglow Canyon (phase III; 2010/2011) [6]** | 116 | 0.55746877 | 1 | 0.0574452 | 0.024630542 | 0.000788766 |
| **Biglow Canyon (phase III; 2010/2011) [6]** | 117 | 0.55746877 | 1 | 0.0574452 | 0.024630542 | 0.000788766 |
| **Biglow Canyon (phase III; 2010/2011) [6]** | 118 | 0.55746877 | 1 | 0.0574452 | 0.024630542 | 0.000788766 |
| **Biglow Canyon (phase III; 2010/2011) [6]** | 119 | 0.55746877 | 1 | 0.0574452 | 0.024630542 | 0.000788766 |
| **Biglow Canyon (phase III; 2010/2011) [6]** | 120 | 0.55746877 | 1 | 0.0574452 | 0.024630542 | 0.000788766 |
| **Biglow Canyon (phase III; 2010/2011) [6]** | 121 | 0.55746877 | 1 | 0.0574452 | 0.024630542 | 0.000788766 |
| **Biglow Canyon (phase III; 2010/2011) [6]** | 122 | 0.55746877 | 1 | 0.0574452 | 0.024630542 | 0.000788766 |
| **Biglow Canyon (phase III; 2010/2011) [6]** | 123 | 0.55746877 | 1 | 0.0574452 | 0.024630542 | 0.000788766 |
| **Biglow Canyon (phase III; 2010/2011) [6]** | 124 | 0.55746877 | 1 | 0.0574452 | 0.024630542 | 0.000788766 |
| **Biglow Canyon (phase III; 2010/2011) [6]** | 125 | 0.55746877 | 1 | 0.0574452 | 0.024630542 | 0.000788766 |
| **Biglow Canyon (phase III; 2010/2011) [6]** | 126 | 0.55746877 | 1 | 0.0574452 | 0.024630542 | 0.000788766 |
| **Biglow Canyon (phase III; 2010/2011) [6]** | 127 | 0.55746877 | 1 | 0.0574452 | 0.024630542 | 0.000788766 |
| **Biglow Canyon (phase III; 2010/2011) [6]** | 128 | 0.55746877 | 1 | 0.0574452 | 0.024630542 | 0.000788766 |
| **Biglow Canyon (phase III; 2010/2011) [6]** | 129 | 0.55746877 | 0 | 0.0574452 | 0.024630542 | 6.64E-10 |
| **Biglow Canyon (phase III; 2010/2011) [6]** | 130 | 0.55746877 | 0 | 0.0574452 | 0.024630542 | 6.64E-10 |
| **Biglow Canyon (phase III; 2010/2011) [6]** | 131 | 0.55746877 | 0 | 0.0574452 | 0.024630542 | 6.64E-10 |
| **Biglow Canyon (phase III; 2010/2011) [6]** | 132 | 0.55746877 | 0 | 0.0574452 | 0.024630542 | 6.64E-10 |
| **Biglow Canyon (phase III; 2010/2011) [6]** | 133 | 0.55746877 | 0 | 0.0574452 | 0.024630542 | 6.64E-10 |
| **Biglow Canyon (phase III; 2010/2011) [6]** | 134 | 0.55746877 | 0 | 0.0574452 | 0.024630542 | 6.64E-10 |
| **Biglow Canyon (phase III; 2010/2011) [6]** | 135 | 0.55746877 | 0 | 0.0574452 | 0.024630542 | 6.64E-10 |
| **Biglow Canyon (phase III; 2010/2011) [6]** | 136 | 0.55746877 | 0 | 0.0574452 | 0.024630542 | 6.64E-10 |
| **Biglow Canyon (phase III; 2010/2011) [6]** | 137 | 0.55746877 | 0 | 0.0574452 | 0.024630542 | 6.64E-10 |
| **Biglow Canyon (phase III; 2010/2011) [6]** | 138 | 0.55746877 | 0 | 0.0574452 | 0.024630542 | 6.64E-10 |
| **Biglow Canyon (phase III; 2010/2011) [6]** | 139 | 0.55746877 | 0 | 0.0574452 | 0.024630542 | 6.64E-10 |
| **Biglow Canyon (phase III; 2010/2011) [6]** | 140 | 0.55746877 | 0 | 0.0574452 | 0.024630542 | 6.64E-10 |
| **Biglow Canyon (phase III; 2010/2011) [6]** | 141 | 0.55746877 | 0 | 0.0574452 | 0.024630542 | 6.64E-10 |
| **Biglow Canyon (phase III; 2010/2011) [6]** | 142 | 0.55746877 | 0 | 0.0574452 | 0.024630542 | 6.64E-10 |
| **Biglow Canyon (phase III; 2010/2011) [6]** | 143 | 0.55746877 | 0 | 0.0574452 | 0.024630542 | 6.64E-10 |
| **Biglow Canyon (phase III; 2010/2011) [6]** | 144 | 0.55746877 | 0 | 0.0574452 | 0.024630542 | 6.64E-10 |
| **Anonymous Wind Facility, OH (2015)** | 3 | 0.98436566 | 0.999835515 | 0.114890401 | 0.0591133 | 0.00668427 |
| **Anonymous Wind Facility, OH (2015)** | 4 | 0.98436566 | 0.999835515 | 0.114890401 | 0.0591133 | 0.00668427 |
| **Anonymous Wind Facility, OH (2015)** | 5 | 0.98436566 | 0.999835515 | 0.114890401 | 0.0591133 | 0.00668427 |
| **Anonymous Wind Facility, OH (2015)** | 6 | 0.98436566 | 0.999835515 | 0.114890401 | 0.0591133 | 0.00668427 |
| **Anonymous Wind Facility, OH (2015)** | 7 | 0.98436566 | 0.997583043 | 0.114890401 | 0.0591133 | 0.006669211 |
| **Anonymous Wind Facility, OH (2015)** | 8 | 0.98436566 | 0.990919503 | 0.114890401 | 0.0591133 | 0.006624663 |
| **Anonymous Wind Facility, OH (2015)** | 9 | 0.98436566 | 0.977564283 | 0.114890401 | 0.0591133 | 0.006535378 |
| **Anonymous Wind Facility, OH (2015)** | 10 | 0.98436566 | 0.964699574 | 0.114890401 | 0.0591133 | 0.006449373 |
| **Anonymous Wind Facility, OH (2015)** | 11 | 0.98436566 | 0.958627242 | 0.114890401 | 0.0591133 | 0.006408777 |
| **Anonymous Wind Facility, OH (2015)** | 12 | 0.98436566 | 0.956056391 | 0.114890401 | 0.0591133 | 0.00639159 |
| **Anonymous Wind Facility, OH (2015)** | 13 | 0.98436566 | 0.954677442 | 0.114890401 | 0.0591133 | 0.006382371 |
| **Anonymous Wind Facility, OH (2015)** | 14 | 0.98436566 | 0.953620261 | 0.114890401 | 0.0591133 | 0.006375304 |
| **Anonymous Wind Facility, OH (2015)** | 15 | 0.98436566 | 0.952773819 | 0.114890401 | 0.0591133 | 0.006369645 |
| **Anonymous Wind Facility, OH (2015)** | 16 | 0.98436566 | 0.952165304 | 0.114890401 | 0.0591133 | 0.006365577 |
| **Anonymous Wind Facility, OH (2015)** | 17 | 0.98436566 | 0.951848343 | 0.114890401 | 0.0591133 | 0.006363458 |
| **Anonymous Wind Facility, OH (2015)** | 18 | 0.98436566 | 0.951625906 | 0.114890401 | 0.0591133 | 0.006361971 |
| **Anonymous Wind Facility, OH (2015)** | 19 | 0.98436566 | 0.951431197 | 0.114890401 | 0.0591133 | 0.006360669 |
| **Anonymous Wind Facility, OH (2015)** | 20 | 0.98436566 | 0.951259269 | 0.114890401 | 0.0591133 | 0.00635952 |
| **Anonymous Wind Facility, OH (2015)** | 21 | 0.98436566 | 0.951106326 | 0.114890401 | 0.0591133 | 0.006358497 |
| **Anonymous Wind Facility, OH (2015)** | 22 | 0.98436566 | 0.950972097 | 0.114890401 | 0.0591133 | 0.0063576 |
| **Anonymous Wind Facility, OH (2015)** | 23 | 0.98436566 | 0.95088389 | 0.114890401 | 0.0591133 | 0.00635701 |
| **Anonymous Wind Facility, OH (2015)** | 24 | 0.98436566 | 0.95081284 | 0.114890401 | 0.0591133 | 0.006356535 |
| **Anonymous Wind Facility, OH (2015)** | 25 | 0.98436566 | 0.950748204 | 0.114890401 | 0.0591133 | 0.006356103 |
| **Anonymous Wind Facility, OH (2015)** | 26 | 0.98436566 | 0.950758871 | 0.114890401 | 0.0591133 | 0.006356174 |
| **Anonymous Wind Facility, OH (2015)** | 27 | 0.98436566 | 0.951760928 | 0.114890401 | 0.0591133 | 0.006362873 |
| **Anonymous Wind Facility, OH (2015)** | 28 | 0.98436566 | 0.952640194 | 0.114890401 | 0.0591133 | 0.006368752 |
| **Anonymous Wind Facility, OH (2015)** | 29 | 0.98436566 | 0.953260512 | 0.114890401 | 0.0591133 | 0.006372899 |
| **Anonymous Wind Facility, OH (2015)** | 30 | 0.98436566 | 0.953763403 | 0.114890401 | 0.0591133 | 0.006376261 |
| **Anonymous Wind Facility, OH (2015)** | 31 | 0.98436566 | 0.954189068 | 0.114890401 | 0.0591133 | 0.006379106 |
| **Anonymous Wind Facility, OH (2015)** | 32 | 0.98436566 | 0.954513852 | 0.114890401 | 0.0591133 | 0.006381278 |
| **Anonymous Wind Facility, OH (2015)** | 33 | 0.98436566 | 0.953316636 | 0.114890401 | 0.0591133 | 0.006373274 |
| **Anonymous Wind Facility, OH (2015)** | 34 | 0.98436566 | 0.952553352 | 0.114890401 | 0.0591133 | 0.006368171 |
| **Anonymous Wind Facility, OH (2015)** | 35 | 0.98436566 | 0.952121417 | 0.114890401 | 0.0591133 | 0.006365283 |
| **Anonymous Wind Facility, OH (2015)** | 36 | 0.98436566 | 0.95180853 | 0.114890401 | 0.0591133 | 0.006363192 |
| **Anonymous Wind Facility, OH (2015)** | 37 | 0.98436566 | 0.951560253 | 0.114890401 | 0.0591133 | 0.006361532 |
| **Anonymous Wind Facility, OH (2015)** | 38 | 0.98436566 | 0.951355922 | 0.114890401 | 0.0591133 | 0.006360166 |
| **Anonymous Wind Facility, OH (2015)** | 39 | 0.98436566 | 0.951183255 | 0.114890401 | 0.0591133 | 0.006359011 |
| **Anonymous Wind Facility, OH (2015)** | 40 | 0.98436566 | 0.951033559 | 0.114890401 | 0.0591133 | 0.006358011 |
| **Anonymous Wind Facility, OH (2015)** | 41 | 0.98436566 | 0.950901623 | 0.114890401 | 0.0591133 | 0.006357129 |
| **Anonymous Wind Facility, OH (2015)** | 42 | 0.98436566 | 0.950784147 | 0.114890401 | 0.0591133 | 0.006356343 |
| **Anonymous Wind Facility, OH (2015)** | 43 | 0.98436566 | 0.950678171 | 0.114890401 | 0.0591133 | 0.006355635 |
| **Anonymous Wind Facility, OH (2015)** | 44 | 0.98436566 | 0.950581885 | 0.114890401 | 0.0591133 | 0.006354991 |
| **Anonymous Wind Facility, OH (2015)** | 45 | 0.98436566 | 0.950494037 | 0.114890401 | 0.0591133 | 0.006354404 |
| **Anonymous Wind Facility, OH (2015)** | 46 | 0.98436566 | 0.950412916 | 0.114890401 | 0.0591133 | 0.006353861 |
| **Anonymous Wind Facility, OH (2015)** | 47 | 0.98436566 | 0.950337776 | 0.114890401 | 0.0591133 | 0.006353359 |
| **Anonymous Wind Facility, OH (2015)** | 48 | 0.98436566 | 0.950268306 | 0.114890401 | 0.0591133 | 0.006352895 |
| **Anonymous Wind Facility, OH (2015)** | 49 | 0.98436566 | 0.950203179 | 0.114890401 | 0.0591133 | 0.006352459 |
| **Anonymous Wind Facility, OH (2015)** | 50 | 0.98436566 | 0.949441804 | 0.114890401 | 0.0591133 | 0.006347369 |
| **Anonymous Wind Facility, OH (2015)** | 51 | 0.98436566 | 0.948357068 | 0.114890401 | 0.0591133 | 0.006340117 |
| **Anonymous Wind Facility, OH (2015)** | 52 | 0.98436566 | 0.947723933 | 0.114890401 | 0.0591133 | 0.006335885 |
| **Anonymous Wind Facility, OH (2015)** | 53 | 0.98436566 | 0.947206707 | 0.114890401 | 0.0591133 | 0.006332427 |
| **Anonymous Wind Facility, OH (2015)** | 54 | 0.98436566 | 0.946667204 | 0.114890401 | 0.0591133 | 0.00632882 |
| **Anonymous Wind Facility, OH (2015)** | 55 | 0.98436566 | 0.946205822 | 0.114890401 | 0.0591133 | 0.006325735 |
| **Anonymous Wind Facility, OH (2015)** | 56 | 0.98436566 | 0.945703975 | 0.114890401 | 0.0591133 | 0.00632238 |
| **Anonymous Wind Facility, OH (2015)** | 57 | 0.98436566 | 0.942126789 | 0.114890401 | 0.0591133 | 0.006298466 |
| **Anonymous Wind Facility, OH (2015)** | 58 | 0.98436566 | 0.935113482 | 0.114890401 | 0.0591133 | 0.006251579 |
| **Anonymous Wind Facility, OH (2015)** | 59 | 0.98436566 | 0.925263574 | 0.114890401 | 0.0591133 | 0.006185729 |
| **Anonymous Wind Facility, OH (2015)** | 60 | 0.98436566 | 0.897903897 | 0.114890401 | 0.0591133 | 0.006002819 |
| **Anonymous Wind Facility, OH (2015)** | 61 | 0.98436566 | 0.843799423 | 0.114890401 | 0.0591133 | 0.005641111 |
| **Anonymous Wind Facility, OH (2015)** | 62 | 0.98436566 | 0.763635823 | 0.114890401 | 0.0591133 | 0.005105188 |
| **Anonymous Wind Facility, OH (2015)** | 63 | 0.98436566 | 0.651838926 | 0.114890401 | 0.0591133 | 0.004357784 |
| **Anonymous Wind Facility, OH (2015)** | 64 | 0.98436566 | 0.546233676 | 0.114890401 | 0.0591133 | 0.003651774 |
| **Anonymous Wind Facility, OH (2015)** | 65 | 0.98436566 | 0.472826642 | 0.114890401 | 0.0591133 | 0.003161021 |
| **Anonymous Wind Facility, OH (2015)** | 66 | 0.98436566 | 0.428530509 | 0.114890401 | 0.0591133 | 0.002864885 |
| **Anonymous Wind Facility, OH (2015)** | 67 | 0.98436566 | 0.412153098 | 0.114890401 | 0.0591133 | 0.002755396 |
| **Anonymous Wind Facility, OH (2015)** | 68 | 0.98436566 | 0.406163182 | 0.114890401 | 0.0591133 | 0.002715351 |
| **Anonymous Wind Facility, OH (2015)** | 69 | 0.98436566 | 0.402277925 | 0.114890401 | 0.0591133 | 0.002689377 |
| **Anonymous Wind Facility, OH (2015)** | 70 | 0.98436566 | 0.39892294 | 0.114890401 | 0.0591133 | 0.002666947 |
| **Anonymous Wind Facility, OH (2015)** | 71 | 0.98436566 | 0.396866634 | 0.114890401 | 0.0591133 | 0.0026532 |
| **Anonymous Wind Facility, OH (2015)** | 72 | 0.98436566 | 0.396019201 | 0.114890401 | 0.0591133 | 0.002647535 |
| **Anonymous Wind Facility, OH (2015)** | 73 | 0.98436566 | 0.395473191 | 0.114890401 | 0.0591133 | 0.002643884 |
| **Anonymous Wind Facility, OH (2015)** | 74 | 0.98436566 | 0.394990107 | 0.114890401 | 0.0591133 | 0.002640655 |
| **Anonymous Wind Facility, OH (2015)** | 75 | 0.98436566 | 0.394591175 | 0.114890401 | 0.0591133 | 0.002637988 |
| **Anonymous Wind Facility, OH (2015)** | 76 | 0.98436566 | 0.39422638 | 0.114890401 | 0.0591133 | 0.002635549 |
| **Anonymous Wind Facility, OH (2015)** | 77 | 0.98436566 | 0.393904857 | 0.114890401 | 0.0591133 | 0.002633399 |
| **Anonymous Wind Facility, OH (2015)** | 78 | 0.98436566 | 0.39380164 | 0.114890401 | 0.0591133 | 0.002632709 |
| **Anonymous Wind Facility, OH (2015)** | 79 | 0.98436566 | 0.393798523 | 0.114890401 | 0.0591133 | 0.002632689 |
| **Anonymous Wind Facility, OH (2015)** | 80 | 0.98436566 | 0.393856871 | 0.114890401 | 0.0591133 | 0.002633079 |
| **Anonymous Wind Facility, OH (2015)** | 81 | 0.98436566 | 0.393916095 | 0.114890401 | 0.0591133 | 0.002633475 |
| **Anonymous Wind Facility, OH (2015)** | 82 | 0.98436566 | 0.394082206 | 0.114890401 | 0.0591133 | 0.002634585 |
| **Anonymous Wind Facility, OH (2015)** | 83 | 0.98436566 | 0.394696465 | 0.114890401 | 0.0591133 | 0.002638692 |
| **Anonymous Wind Facility, OH (2015)** | 84 | 0.98436566 | 0.395348315 | 0.114890401 | 0.0591133 | 0.00264305 |
| **Anonymous Wind Facility, OH (2015)** | 85 | 0.98436566 | 0.395801022 | 0.114890401 | 0.0591133 | 0.002646076 |
| **Anonymous Wind Facility, OH (2015)** | 86 | 0.98436566 | 0.395400862 | 0.114890401 | 0.0591133 | 0.002643401 |
| **Anonymous Wind Facility, OH (2015)** | 87 | 0.98436566 | 0.393837304 | 0.114890401 | 0.0591133 | 0.002632948 |
| **Anonymous Wind Facility, OH (2015)** | 88 | 0.98436566 | 0.392627891 | 0.114890401 | 0.0591133 | 0.002624862 |
| **Anonymous Wind Facility, OH (2015)** | 89 | 0.98436566 | 0.389862351 | 0.114890401 | 0.0591133 | 0.002606374 |
| **Anonymous Wind Facility, OH (2015)** | 90 | 0.98436566 | 0.380720625 | 0.114890401 | 0.0591133 | 0.002545258 |
| **Anonymous Wind Facility, OH (2015)** | 91 | 0.98436566 | 0.359215327 | 0.114890401 | 0.0591133 | 0.002401487 |
| **Anonymous Wind Facility, OH (2015)** | 92 | 0.98436566 | 0.319298411 | 0.114890401 | 0.0591133 | 0.002134628 |
| **Anonymous Wind Facility, OH (2015)** | 93 | 0.98436566 | 0.257289395 | 0.114890401 | 0.0591133 | 0.001720075 |
| **Anonymous Wind Facility, OH (2015)** | 94 | 0.98436566 | 0.187033466 | 0.114890401 | 0.0591133 | 0.001250388 |
| **Anonymous Wind Facility, OH (2015)** | 95 | 0.98436566 | 0.119597201 | 0.114890401 | 0.0591133 | 0.000799551 |
| **Anonymous Wind Facility, OH (2015)** | 96 | 0.98436566 | 0.069533035 | 0.114890401 | 0.0591133 | 0.000464854 |
| **Anonymous Wind Facility, OH (2015)** | 97 | 0.98436566 | 0.040079291 | 0.114890401 | 0.0591133 | 0.000267945 |
| **Anonymous Wind Facility, OH (2015)** | 98 | 0.98436566 | 0.026950453 | 0.114890401 | 0.0591133 | 0.000180174 |
| **Anonymous Wind Facility, OH (2015)** | 99 | 0.98436566 | 0.021033926 | 0.114890401 | 0.0591133 | 0.00014062 |
| **Anonymous Wind Facility, OH (2015)** | 100 | 0.98436566 | 0.018105798 | 0.114890401 | 0.0591133 | 0.000121044 |
| **Anonymous Wind Facility, OH (2015)** | 101 | 0.98436566 | 0.016720218 | 0.114890401 | 0.0591133 | 0.000111781 |
| **Anonymous Wind Facility, OH (2015)** | 102 | 0.98436566 | 0.016041345 | 0.114890401 | 0.0591133 | 0.000107242 |
| **Anonymous Wind Facility, OH (2015)** | 103 | 0.98436566 | 0.015672781 | 0.114890401 | 0.0591133 | 0.000104778 |
| **Anonymous Wind Facility, OH (2015)** | 104 | 0.98436566 | 0.015353922 | 0.114890401 | 0.0591133 | 0.000102647 |
| **Anonymous Wind Facility, OH (2015)** | 105 | 0.98436566 | 0.015067614 | 0.114890401 | 0.0591133 | 0.000100733 |
| **Anonymous Wind Facility, OH (2015)** | 106 | 0.98436566 | 0.014776172 | 0.114890401 | 0.0591133 | 9.88E-05 |
| **Anonymous Wind Facility, OH (2015)** | 107 | 0.98436566 | 0.01427032 | 0.114890401 | 0.0591133 | 9.54E-05 |
| **Anonymous Wind Facility, OH (2015)** | 108 | 0.98436566 | 0.013435379 | 0.114890401 | 0.0591133 | 8.98E-05 |
| **Anonymous Wind Facility, OH (2015)** | 109 | 0.98436566 | 0.012890338 | 0.114890401 | 0.0591133 | 8.62E-05 |
| **Anonymous Wind Facility, OH (2015)** | 110 | 0.98436566 | 0.012506772 | 0.114890401 | 0.0591133 | 8.36E-05 |
| **Anonymous Wind Facility, OH (2015)** | 111 | 0.98436566 | 0.01137796 | 0.114890401 | 0.0591133 | 7.61E-05 |
| **Anonymous Wind Facility, OH (2015)** | 112 | 0.98436566 | 0.010709596 | 0.114890401 | 0.0591133 | 7.16E-05 |
| **Anonymous Wind Facility, OH (2015)** | 113 | 0.98436566 | 0.010526268 | 0.114890401 | 0.0591133 | 7.04E-05 |
| **Anonymous Wind Facility, OH (2015)** | 114 | 0.98436566 | 0.009044135 | 0.114890401 | 0.0591133 | 6.05E-05 |
| **Anonymous Wind Facility, OH (2015)** | 115 | 0.98436566 | 0.007756427 | 0.114890401 | 0.0591133 | 5.19E-05 |
| **Anonymous Wind Facility, OH (2015)** | 116 | 0.98436566 | 0.007570269 | 0.114890401 | 0.0591133 | 5.06E-05 |
| **Anonymous Wind Facility, OH (2015)** | 117 | 0.98436566 | 0.006154644 | 0.114890401 | 0.0591133 | 4.11E-05 |
| **Anonymous Wind Facility, OH (2015)** | 118 | 0.98436566 | 0.007065715 | 0.114890401 | 0.0591133 | 4.72E-05 |
| **Anonymous Wind Facility, OH (2015)** | 119 | 0.98436566 | 0.007046555 | 0.114890401 | 0.0591133 | 4.71E-05 |
| **Anonymous Wind Facility, OH (2015)** | 120 | 0.98436566 | 0.007867124 | 0.114890401 | 0.0591133 | 5.26E-05 |
| **Anonymous Wind Facility, OH (2015)** | 121 | 0.98436566 | 0.007100981 | 0.114890401 | 0.0591133 | 4.75E-05 |
| **Anonymous Wind Facility, OH (2015)** | 122 | 0.98436566 | 0.006456296 | 0.114890401 | 0.0591133 | 4.32E-05 |
| **Anonymous Wind Facility, OH (2015)** | 123 | 0.98436566 | 0.006421056 | 0.114890401 | 0.0591133 | 4.29E-05 |
| **Anonymous Wind Facility, OH (2015)** | 124 | 0.98436566 | 0.009819148 | 0.114890401 | 0.0591133 | 6.56E-05 |
| **Anonymous Wind Facility, OH (2015)** | 125 | 0.98436566 | 0.009719727 | 0.114890401 | 0.0591133 | 6.50E-05 |
| **Anonymous Wind Facility, OH (2015)** | 126 | 0.98436566 | 0.009623666 | 0.114890401 | 0.0591133 | 6.43E-05 |
| **Anonymous Wind Facility, OH (2015)** | 127 | 0.98436566 | 0.008890635 | 0.114890401 | 0.0591133 | 5.94E-05 |
| **Anonymous Wind Facility, OH (2015)** | 128 | 0.98436566 | 0.007254564 | 0.114890401 | 0.0591133 | 4.85E-05 |
| **Anonymous Wind Facility, OH (2015)** | 129 | 0.98436566 | 0.007768232 | 0.114890401 | 0.0591133 | 5.19E-05 |
| **Anonymous Wind Facility, OH (2015)** | 130 | 0.98436566 | 0.006570916 | 0.114890401 | 0.0591133 | 4.39E-05 |
| **Anonymous Wind Facility, OH (2015)** | 131 | 0.98436566 | 0.007446974 | 0.114890401 | 0.0591133 | 4.98E-05 |
| **Anonymous Wind Facility, OH (2015)** | 132 | 0.98436566 | 0.006369231 | 0.114890401 | 0.0591133 | 4.26E-05 |
| **Anonymous Wind Facility, OH (2015)** | 133 | 0.98436566 | 0.008750703 | 0.114890401 | 0.0591133 | 5.85E-05 |
| **Anonymous Wind Facility, OH (2015)** | 134 | 0.98436566 | 0.008642602 | 0.114890401 | 0.0591133 | 5.78E-05 |
| **Anonymous Wind Facility, OH (2015)** | 135 | 0.98436566 | 0.008537006 | 0.114890401 | 0.0591133 | 5.71E-05 |
| **Anonymous Wind Facility, OH (2015)** | 136 | 0.98436566 | 0.008437823 | 0.114890401 | 0.0591133 | 5.64E-05 |
| **Anonymous Wind Facility, OH (2015)** | 137 | 0.98436566 | 0.008340858 | 0.114890401 | 0.0591133 | 5.58E-05 |
| **Anonymous Wind Facility, OH (2015)** | 138 | 0.98436566 | 0.008244886 | 0.114890401 | 0.0591133 | 5.51E-05 |
| **Anonymous Wind Facility, OH (2015)** | 139 | 0.98436566 | 0.008151914 | 0.114890401 | 0.0591133 | 5.45E-05 |
| **Anonymous Wind Facility, OH (2015)** | 140 | 0.98436566 | 0.008070024 | 0.114890401 | 0.0591133 | 5.40E-05 |
| **Anonymous Wind Facility, OH (2015)** | 141 | 0.98436566 | 0.00798966 | 0.114890401 | 0.0591133 | 5.34E-05 |
| **Anonymous Wind Facility, OH (2015)** | 142 | 0.98436566 | 0.007910447 | 0.114890401 | 0.0591133 | 5.29E-05 |
| **Anonymous Wind Facility, OH (2015)** | 143 | 0.98436566 | 0.007834544 | 0.114890401 | 0.0591133 | 5.24E-05 |
| **Anonymous Wind Facility, OH (2015)** | 144 | 0.98436566 | 0.007762726 | 0.114890401 | 0.0591133 | 5.19E-05 |
| **Buffalo Ridge I (2010) [7]** | 3 | 0.57079616 | 0.999835515 | 0.01814059 | 0.098522167 | 0.001019988 |
| **Buffalo Ridge I (2010) [7]** | 4 | 0.57079616 | 0.999835515 | 0.01814059 | 0.098522167 | 0.001019988 |
| **Buffalo Ridge I (2010) [7]** | 5 | 0.57079616 | 0.999835515 | 0.01814059 | 0.098522167 | 0.001019988 |
| **Buffalo Ridge I (2010) [7]** | 6 | 0.57079616 | 0.999835515 | 0.01814059 | 0.098522167 | 0.001019988 |
| **Buffalo Ridge I (2010) [7]** | 7 | 0.57079616 | 0.996621694 | 0.01814059 | 0.098522167 | 0.001016709 |
| **Buffalo Ridge I (2010) [7]** | 8 | 0.57079616 | 0.976389621 | 0.01814059 | 0.098522167 | 0.000996069 |
| **Buffalo Ridge I (2010) [7]** | 9 | 0.57079616 | 0.913513896 | 0.01814059 | 0.098522167 | 0.000931926 |
| **Buffalo Ridge I (2010) [7]** | 10 | 0.57079616 | 0.834471579 | 0.01814059 | 0.098522167 | 0.000851291 |
| **Buffalo Ridge I (2010) [7]** | 11 | 0.57079616 | 0.762210056 | 0.01814059 | 0.098522167 | 0.000777573 |
| **Buffalo Ridge I (2010) [7]** | 12 | 0.57079616 | 0.716919668 | 0.01814059 | 0.098522167 | 0.00073137 |
| **Buffalo Ridge I (2010) [7]** | 13 | 0.57079616 | 0.69283476 | 0.01814059 | 0.098522167 | 0.000706799 |
| **Buffalo Ridge I (2010) [7]** | 14 | 0.57079616 | 0.678684416 | 0.01814059 | 0.098522167 | 0.000692364 |
| **Buffalo Ridge I (2010) [7]** | 15 | 0.57079616 | 0.670921299 | 0.01814059 | 0.098522167 | 0.000684444 |
| **Buffalo Ridge I (2010) [7]** | 16 | 0.57079616 | 0.672433227 | 0.01814059 | 0.098522167 | 0.000685986 |
| **Buffalo Ridge I (2010) [7]** | 17 | 0.57079616 | 0.674686764 | 0.01814059 | 0.098522167 | 0.000688285 |
| **Buffalo Ridge I (2010) [7]** | 18 | 0.57079616 | 0.67513047 | 0.01814059 | 0.098522167 | 0.000688738 |
| **Buffalo Ridge I (2010) [7]** | 19 | 0.57079616 | 0.676804495 | 0.01814059 | 0.098522167 | 0.000690446 |
| **Buffalo Ridge I (2010) [7]** | 20 | 0.57079616 | 0.67474047 | 0.01814059 | 0.098522167 | 0.00068834 |
| **Buffalo Ridge I (2010) [7]** | 21 | 0.57079616 | 0.6651985 | 0.01814059 | 0.098522167 | 0.000678606 |
| **Buffalo Ridge I (2010) [7]** | 22 | 0.57079616 | 0.656318177 | 0.01814059 | 0.098522167 | 0.000669547 |
| **Buffalo Ridge I (2010) [7]** | 23 | 0.57079616 | 0.650268339 | 0.01814059 | 0.098522167 | 0.000663375 |
| **Buffalo Ridge I (2010) [7]** | 24 | 0.57079616 | 0.646034227 | 0.01814059 | 0.098522167 | 0.000659055 |
| **Buffalo Ridge I (2010) [7]** | 25 | 0.57079616 | 0.643521829 | 0.01814059 | 0.098522167 | 0.000656492 |
| **Buffalo Ridge I (2010) [7]** | 26 | 0.57079616 | 0.642099476 | 0.01814059 | 0.098522167 | 0.000655041 |
| **Buffalo Ridge I (2010) [7]** | 27 | 0.57079616 | 0.645615165 | 0.01814059 | 0.098522167 | 0.000658628 |
| **Buffalo Ridge I (2010) [7]** | 28 | 0.57079616 | 0.647604622 | 0.01814059 | 0.098522167 | 0.000660657 |
| **Buffalo Ridge I (2010) [7]** | 29 | 0.57079616 | 0.646252161 | 0.01814059 | 0.098522167 | 0.000659278 |
| **Buffalo Ridge I (2010) [7]** | 30 | 0.57079616 | 0.644146203 | 0.01814059 | 0.098522167 | 0.000657129 |
| **Buffalo Ridge I (2010) [7]** | 31 | 0.57079616 | 0.641653155 | 0.01814059 | 0.098522167 | 0.000654586 |
| **Buffalo Ridge I (2010) [7]** | 32 | 0.57079616 | 0.635045923 | 0.01814059 | 0.098522167 | 0.000647846 |
| **Buffalo Ridge I (2010) [7]** | 33 | 0.57079616 | 0.629529798 | 0.01814059 | 0.098522167 | 0.000642218 |
| **Buffalo Ridge I (2010) [7]** | 34 | 0.57079616 | 0.625226726 | 0.01814059 | 0.098522167 | 0.000637829 |
| **Buffalo Ridge I (2010) [7]** | 35 | 0.57079616 | 0.621633069 | 0.01814059 | 0.098522167 | 0.000634162 |
| **Buffalo Ridge I (2010) [7]** | 36 | 0.57079616 | 0.618484371 | 0.01814059 | 0.098522167 | 0.00063095 |
| **Buffalo Ridge I (2010) [7]** | 37 | 0.57079616 | 0.615574084 | 0.01814059 | 0.098522167 | 0.000627981 |
| **Buffalo Ridge I (2010) [7]** | 38 | 0.57079616 | 0.612922635 | 0.01814059 | 0.098522167 | 0.000625276 |
| **Buffalo Ridge I (2010) [7]** | 39 | 0.57079616 | 0.610502293 | 0.01814059 | 0.098522167 | 0.000622807 |
| **Buffalo Ridge I (2010) [7]** | 40 | 0.57079616 | 0.608906786 | 0.01814059 | 0.098522167 | 0.00062118 |
| **Buffalo Ridge I (2010) [7]** | 41 | 0.57079616 | 0.611787215 | 0.01814059 | 0.098522167 | 0.000624118 |
| **Buffalo Ridge I (2010) [7]** | 42 | 0.57079616 | 0.615027353 | 0.01814059 | 0.098522167 | 0.000627424 |
| **Buffalo Ridge I (2010) [7]** | 43 | 0.57079616 | 0.617073548 | 0.01814059 | 0.098522167 | 0.000629511 |
| **Buffalo Ridge I (2010) [7]** | 44 | 0.57079616 | 0.618621791 | 0.01814059 | 0.098522167 | 0.00063109 |
| **Buffalo Ridge I (2010) [7]** | 45 | 0.57079616 | 0.619106727 | 0.01814059 | 0.098522167 | 0.000631585 |
| **Buffalo Ridge I (2010) [7]** | 46 | 0.57079616 | 0.612863447 | 0.01814059 | 0.098522167 | 0.000625216 |
| **Buffalo Ridge I (2010) [7]** | 47 | 0.57079616 | 0.60448548 | 0.01814059 | 0.098522167 | 0.000616669 |
| **Buffalo Ridge I (2010) [7]** | 48 | 0.57079616 | 0.599486409 | 0.01814059 | 0.098522167 | 0.000611569 |
| **Buffalo Ridge I (2010) [7]** | 49 | 0.57079616 | 0.598425211 | 0.01814059 | 0.098522167 | 0.000610487 |
| **Buffalo Ridge I (2010) [7]** | 50 | 0.57079616 | 0.595669096 | 0.01814059 | 0.098522167 | 0.000607675 |
| **Buffalo Ridge I (2010) [7]** | 51 | 0.57079616 | 0.592829721 | 0.01814059 | 0.098522167 | 0.000604779 |
| **Buffalo Ridge I (2010) [7]** | 52 | 0.57079616 | 0.590024408 | 0.01814059 | 0.098522167 | 0.000601917 |
| **Buffalo Ridge I (2010) [7]** | 53 | 0.57079616 | 0.589204856 | 0.01814059 | 0.098522167 | 0.000601081 |
| **Buffalo Ridge I (2010) [7]** | 54 | 0.57079616 | 0.585392468 | 0.01814059 | 0.098522167 | 0.000597191 |
| **Buffalo Ridge I (2010) [7]** | 55 | 0.57079616 | 0.581729036 | 0.01814059 | 0.098522167 | 0.000593454 |
| **Buffalo Ridge I (2010) [7]** | 56 | 0.57079616 | 0.577021932 | 0.01814059 | 0.098522167 | 0.000588652 |
| **Buffalo Ridge I (2010) [7]** | 57 | 0.57079616 | 0.566504194 | 0.01814059 | 0.098522167 | 0.000577922 |
| **Buffalo Ridge I (2010) [7]** | 58 | 0.57079616 | 0.553147927 | 0.01814059 | 0.098522167 | 0.000564297 |
| **Buffalo Ridge I (2010) [7]** | 59 | 0.57079616 | 0.541836375 | 0.01814059 | 0.098522167 | 0.000552757 |
| **Buffalo Ridge I (2010) [7]** | 60 | 0.57079616 | 0.533003108 | 0.01814059 | 0.098522167 | 0.000543746 |
| **Buffalo Ridge I (2010) [7]** | 61 | 0.57079616 | 0.525918627 | 0.01814059 | 0.098522167 | 0.000536519 |
| **Buffalo Ridge I (2010) [7]** | 62 | 0.57079616 | 0.519731867 | 0.01814059 | 0.098522167 | 0.000530207 |
| **Buffalo Ridge I (2010) [7]** | 63 | 0.57079616 | 0.514139592 | 0.01814059 | 0.098522167 | 0.000524502 |
| **Buffalo Ridge I (2010) [7]** | 64 | 0.57079616 | 0.509024569 | 0.01814059 | 0.098522167 | 0.000519284 |
| **Buffalo Ridge I (2010) [7]** | 65 | 0.57079616 | 0.507046442 | 0.01814059 | 0.098522167 | 0.000517266 |
| **Buffalo Ridge I (2010) [7]** | 66 | 0.57079616 | 0.509961503 | 0.01814059 | 0.098522167 | 0.00052024 |
| **Buffalo Ridge I (2010) [7]** | 67 | 0.57079616 | 0.513194174 | 0.01814059 | 0.098522167 | 0.000523538 |
| **Buffalo Ridge I (2010) [7]** | 68 | 0.57079616 | 0.514594057 | 0.01814059 | 0.098522167 | 0.000524966 |
| **Buffalo Ridge I (2010) [7]** | 69 | 0.57079616 | 0.514665899 | 0.01814059 | 0.098522167 | 0.000525039 |
| **Buffalo Ridge I (2010) [7]** | 70 | 0.57079616 | 0.511892954 | 0.01814059 | 0.098522167 | 0.00052221 |
| **Buffalo Ridge I (2010) [7]** | 71 | 0.57079616 | 0.504837284 | 0.01814059 | 0.098522167 | 0.000515013 |
| **Buffalo Ridge I (2010) [7]** | 72 | 0.57079616 | 0.498476862 | 0.01814059 | 0.098522167 | 0.000508524 |
| **Buffalo Ridge I (2010) [7]** | 73 | 0.57079616 | 0.49409899 | 0.01814059 | 0.098522167 | 0.000504058 |
| **Buffalo Ridge I (2010) [7]** | 74 | 0.57079616 | 0.490510302 | 0.01814059 | 0.098522167 | 0.000500397 |
| **Buffalo Ridge I (2010) [7]** | 75 | 0.57079616 | 0.48806828 | 0.01814059 | 0.098522167 | 0.000497906 |
| **Buffalo Ridge I (2010) [7]** | 76 | 0.57079616 | 0.488050749 | 0.01814059 | 0.098522167 | 0.000497888 |
| **Buffalo Ridge I (2010) [7]** | 77 | 0.57079616 | 0.488974875 | 0.01814059 | 0.098522167 | 0.00049883 |
| **Buffalo Ridge I (2010) [7]** | 78 | 0.57079616 | 0.490825558 | 0.01814059 | 0.098522167 | 0.000500718 |
| **Buffalo Ridge I (2010) [7]** | 79 | 0.57079616 | 0.513373436 | 0.01814059 | 0.098522167 | 0.000523721 |
| **Buffalo Ridge I (2010) [7]** | 80 | 0.57079616 | 0.512028102 | 0.01814059 | 0.098522167 | 0.000522348 |
| **Buffalo Ridge I (2010) [7]** | 81 | 0.57079616 | 0.531438282 | 0.01814059 | 0.098522167 | 0.00054215 |
| **Buffalo Ridge I (2010) [7]** | 82 | 0.57079616 | 0.527893899 | 0.01814059 | 0.098522167 | 0.000538534 |
| **Buffalo Ridge I (2010) [7]** | 83 | 0.57079616 | 0.522596177 | 0.01814059 | 0.098522167 | 0.000533129 |
| **Buffalo Ridge I (2010) [7]** | 84 | 0.57079616 | 0.519114279 | 0.01814059 | 0.098522167 | 0.000529577 |
| **Buffalo Ridge I (2010) [7]** | 85 | 0.57079616 | 0.517010222 | 0.01814059 | 0.098522167 | 0.000527431 |
| **Buffalo Ridge I (2010) [7]** | 86 | 0.57079616 | 0.515113766 | 0.01814059 | 0.098522167 | 0.000525496 |
| **Buffalo Ridge I (2010) [7]** | 87 | 0.57079616 | 0.513156 | 0.01814059 | 0.098522167 | 0.000523499 |
| **Buffalo Ridge I (2010) [7]** | 88 | 0.57079616 | 0.511838188 | 0.01814059 | 0.098522167 | 0.000522155 |
| **Buffalo Ridge I (2010) [7]** | 89 | 0.57079616 | 0.513051257 | 0.01814059 | 0.098522167 | 0.000523392 |
| **Buffalo Ridge I (2010) [7]** | 90 | 0.57079616 | 0.515565342 | 0.01814059 | 0.098522167 | 0.000525957 |
| **Buffalo Ridge I (2010) [7]** | 91 | 0.57079616 | 0.518052276 | 0.01814059 | 0.098522167 | 0.000528494 |
| **Buffalo Ridge I (2010) [7]** | 92 | 0.57079616 | 0.522537112 | 0.01814059 | 0.098522167 | 0.000533069 |
| **Buffalo Ridge I (2010) [7]** | 93 | 0.57079616 | 0.525854689 | 0.01814059 | 0.098522167 | 0.000536454 |
| **Buffalo Ridge I (2010) [7]** | 94 | 0.57079616 | 0.525648645 | 0.01814059 | 0.098522167 | 0.000536243 |
| **Buffalo Ridge I (2010) [7]** | 95 | 0.57079616 | 0.523629132 | 0.01814059 | 0.098522167 | 0.000534183 |
| **Buffalo Ridge I (2010) [7]** | 96 | 0.57079616 | 0.521398004 | 0.01814059 | 0.098522167 | 0.000531907 |
| **Buffalo Ridge I (2010) [7]** | 97 | 0.57079616 | 0.51536106 | 0.01814059 | 0.098522167 | 0.000525748 |
| **Buffalo Ridge I (2010) [7]** | 98 | 0.57079616 | 0.508506703 | 0.01814059 | 0.098522167 | 0.000518756 |
| **Buffalo Ridge I (2010) [7]** | 99 | 0.57079616 | 0.500985139 | 0.01814059 | 0.098522167 | 0.000511083 |
| **Buffalo Ridge I (2010) [7]** | 100 | 0.57079616 | 0.494072128 | 0.01814059 | 0.098522167 | 0.00050403 |
| **Buffalo Ridge I (2010) [7]** | 101 | 0.57079616 | 0.480771923 | 0.01814059 | 0.098522167 | 0.000490462 |
| **Buffalo Ridge I (2010) [7]** | 102 | 0.57079616 | 0.46493128 | 0.01814059 | 0.098522167 | 0.000474302 |
| **Buffalo Ridge I (2010) [7]** | 103 | 0.57079616 | 0.44273507 | 0.01814059 | 0.098522167 | 0.000451659 |
| **Buffalo Ridge I (2010) [7]** | 104 | 0.57079616 | 0.417258968 | 0.01814059 | 0.098522167 | 0.000425669 |
| **Buffalo Ridge I (2010) [7]** | 105 | 0.57079616 | 0.387948018 | 0.01814059 | 0.098522167 | 0.000395767 |
| **Buffalo Ridge I (2010) [7]** | 106 | 0.57079616 | 0.36069191 | 0.01814059 | 0.098522167 | 0.000367962 |
| **Buffalo Ridge I (2010) [7]** | 107 | 0.57079616 | 0.337415806 | 0.01814059 | 0.098522167 | 0.000344217 |
| **Buffalo Ridge I (2010) [7]** | 108 | 0.57079616 | 0.312146644 | 0.01814059 | 0.098522167 | 0.000318438 |
| **Buffalo Ridge I (2010) [7]** | 109 | 0.57079616 | 0.290984254 | 0.01814059 | 0.098522167 | 0.000296849 |
| **Buffalo Ridge I (2010) [7]** | 110 | 0.57079616 | 0.272320286 | 0.01814059 | 0.098522167 | 0.000277809 |
| **Buffalo Ridge I (2010) [7]** | 111 | 0.57079616 | 0.256038765 | 0.01814059 | 0.098522167 | 0.000261199 |
| **Buffalo Ridge I (2010) [7]** | 112 | 0.57079616 | 0.240741602 | 0.01814059 | 0.098522167 | 0.000245594 |
| **Buffalo Ridge I (2010) [7]** | 113 | 0.57079616 | 0.226930228 | 0.01814059 | 0.098522167 | 0.000231504 |
| **Buffalo Ridge I (2010) [7]** | 114 | 0.57079616 | 0.21433972 | 0.01814059 | 0.098522167 | 0.00021866 |
| **Buffalo Ridge I (2010) [7]** | 115 | 0.57079616 | 0.202579914 | 0.01814059 | 0.098522167 | 0.000206663 |
| **Buffalo Ridge I (2010) [7]** | 116 | 0.57079616 | 0.191731399 | 0.01814059 | 0.098522167 | 0.000195596 |
| **Buffalo Ridge I (2010) [7]** | 117 | 0.57079616 | 0.181980915 | 0.01814059 | 0.098522167 | 0.000185649 |
| **Buffalo Ridge I (2010) [7]** | 118 | 0.57079616 | 0.172954488 | 0.01814059 | 0.098522167 | 0.00017644 |
| **Buffalo Ridge I (2010) [7]** | 119 | 0.57079616 | 0.164042164 | 0.01814059 | 0.098522167 | 0.000167349 |
| **Buffalo Ridge I (2010) [7]** | 120 | 0.57079616 | 0.155011319 | 0.01814059 | 0.098522167 | 0.000158136 |
| **Buffalo Ridge I (2010) [7]** | 121 | 0.57079616 | 0.14591307 | 0.01814059 | 0.098522167 | 0.000148854 |
| **Buffalo Ridge I (2010) [7]** | 122 | 0.57079616 | 0.137009182 | 0.01814059 | 0.098522167 | 0.000139771 |
| **Buffalo Ridge I (2010) [7]** | 123 | 0.57079616 | 0.128526578 | 0.01814059 | 0.098522167 | 0.000131117 |
| **Buffalo Ridge I (2010) [7]** | 124 | 0.57079616 | 0.120726783 | 0.01814059 | 0.098522167 | 0.00012316 |
| **Buffalo Ridge I (2010) [7]** | 125 | 0.57079616 | 0.113539231 | 0.01814059 | 0.098522167 | 0.000115828 |
| **Buffalo Ridge I (2010) [7]** | 126 | 0.57079616 | 0.10605193 | 0.01814059 | 0.098522167 | 0.000108189 |
| **Buffalo Ridge I (2010) [7]** | 127 | 0.57079616 | 0.098590409 | 0.01814059 | 0.098522167 | 0.000100578 |
| **Buffalo Ridge I (2010) [7]** | 128 | 0.57079616 | 0.091276252 | 0.01814059 | 0.098522167 | 9.31E-05 |
| **Buffalo Ridge I (2010) [7]** | 129 | 0.57079616 | 0.084202214 | 0.01814059 | 0.098522167 | 8.59E-05 |
| **Buffalo Ridge I (2010) [7]** | 130 | 0.57079616 | 0.077802016 | 0.01814059 | 0.098522167 | 7.94E-05 |
| **Buffalo Ridge I (2010) [7]** | 131 | 0.57079616 | 0.071854523 | 0.01814059 | 0.098522167 | 7.33E-05 |
| **Buffalo Ridge I (2010) [7]** | 132 | 0.57079616 | 0.066136687 | 0.01814059 | 0.098522167 | 6.75E-05 |
| **Buffalo Ridge I (2010) [7]** | 133 | 0.57079616 | 0.060649688 | 0.01814059 | 0.098522167 | 6.19E-05 |
| **Buffalo Ridge I (2010) [7]** | 134 | 0.57079616 | 0.055141112 | 0.01814059 | 0.098522167 | 5.63E-05 |
| **Buffalo Ridge I (2010) [7]** | 135 | 0.57079616 | 0.049460387 | 0.01814059 | 0.098522167 | 5.05E-05 |
| **Buffalo Ridge I (2010) [7]** | 136 | 0.57079616 | 0.043602725 | 0.01814059 | 0.098522167 | 4.45E-05 |
| **Buffalo Ridge I (2010) [7]** | 137 | 0.57079616 | 0.03764945 | 0.01814059 | 0.098522167 | 3.84E-05 |
| **Buffalo Ridge I (2010) [7]** | 138 | 0.57079616 | 0.031990742 | 0.01814059 | 0.098522167 | 3.26E-05 |
| **Buffalo Ridge I (2010) [7]** | 139 | 0.57079616 | 0.029693999 | 0.01814059 | 0.098522167 | 3.03E-05 |
| **Buffalo Ridge I (2010) [7]** | 140 | 0.57079616 | 0.027614034 | 0.01814059 | 0.098522167 | 2.82E-05 |
| **Buffalo Ridge I (2010) [7]** | 141 | 0.57079616 | 0.023876538 | 0.01814059 | 0.098522167 | 2.44E-05 |
| **Buffalo Ridge I (2010) [7]** | 142 | 0.57079616 | 0.019745601 | 0.01814059 | 0.098522167 | 2.01E-05 |
| **Buffalo Ridge I (2010) [7]** | 143 | 0.57079616 | 0.014422586 | 0.01814059 | 0.098522167 | 1.47E-05 |
| **Buffalo Ridge I (2010) [7]** | 144 | 0.57079616 | 0.009621275 | 0.01814059 | 0.098522167 | 9.82E-06 |
| **Anonymous Wind Facility, MO** | 3 | 0.46099032 | 0.898000032 | 0.055177627 | 0.044334975 | 0.001012693 |
| **Anonymous Wind Facility, MO** | 4 | 0.46099032 | 0.795177621 | 0.055177627 | 0.044334975 | 0.000896738 |
| **Anonymous Wind Facility, MO** | 5 | 0.46099032 | 0.665636883 | 0.055177627 | 0.044334975 | 0.000750652 |
| **Anonymous Wind Facility, MO** | 6 | 0.46099032 | 0.622505212 | 0.055177627 | 0.044334975 | 0.000702012 |
| **Anonymous Wind Facility, MO** | 7 | 0.46099032 | 0.536633865 | 0.055177627 | 0.044334975 | 0.000605173 |
| **Anonymous Wind Facility, MO** | 8 | 0.46099032 | 0.478661939 | 0.055177627 | 0.044334975 | 0.000539797 |
| **Anonymous Wind Facility, MO** | 9 | 0.46099032 | 0.424384057 | 0.055177627 | 0.044334975 | 0.000478586 |
| **Anonymous Wind Facility, MO** | 10 | 0.46099032 | 0.394587421 | 0.055177627 | 0.044334975 | 0.000444984 |
| **Anonymous Wind Facility, MO** | 11 | 0.46099032 | 0.365499557 | 0.055177627 | 0.044334975 | 0.000412181 |
| **Anonymous Wind Facility, MO** | 12 | 0.46099032 | 0.346639414 | 0.055177627 | 0.044334975 | 0.000390912 |
| **Anonymous Wind Facility, MO** | 13 | 0.46099032 | 0.329696166 | 0.055177627 | 0.044334975 | 0.000371805 |
| **Anonymous Wind Facility, MO** | 14 | 0.46099032 | 0.320963386 | 0.055177627 | 0.044334975 | 0.000361957 |
| **Anonymous Wind Facility, MO** | 15 | 0.46099032 | 0.31040819 | 0.055177627 | 0.044334975 | 0.000350054 |
| **Anonymous Wind Facility, MO** | 16 | 0.46099032 | 0.298821447 | 0.055177627 | 0.044334975 | 0.000336987 |
| **Anonymous Wind Facility, MO** | 17 | 0.46099032 | 0.288393909 | 0.055177627 | 0.044334975 | 0.000325228 |
| **Anonymous Wind Facility, MO** | 18 | 0.46099032 | 0.278868039 | 0.055177627 | 0.044334975 | 0.000314485 |
| **Anonymous Wind Facility, MO** | 19 | 0.46099032 | 0.266607322 | 0.055177627 | 0.044334975 | 0.000300658 |
| **Anonymous Wind Facility, MO** | 20 | 0.46099032 | 0.261530674 | 0.055177627 | 0.044334975 | 0.000294933 |
| **Anonymous Wind Facility, MO** | 21 | 0.46099032 | 0.264378965 | 0.055177627 | 0.044334975 | 0.000298145 |
| **Anonymous Wind Facility, MO** | 22 | 0.46099032 | 0.275981151 | 0.055177627 | 0.044334975 | 0.000311229 |
| **Anonymous Wind Facility, MO** | 23 | 0.46099032 | 0.289139535 | 0.055177627 | 0.044334975 | 0.000326068 |
| **Anonymous Wind Facility, MO** | 24 | 0.46099032 | 0.302681996 | 0.055177627 | 0.044334975 | 0.000341341 |
| **Anonymous Wind Facility, MO** | 25 | 0.46099032 | 0.312980824 | 0.055177627 | 0.044334975 | 0.000352955 |
| **Anonymous Wind Facility, MO** | 26 | 0.46099032 | 0.318475587 | 0.055177627 | 0.044334975 | 0.000359151 |
| **Anonymous Wind Facility, MO** | 27 | 0.46099032 | 0.314717752 | 0.055177627 | 0.044334975 | 0.000354914 |
| **Anonymous Wind Facility, MO** | 28 | 0.46099032 | 0.312483229 | 0.055177627 | 0.044334975 | 0.000352394 |
| **Anonymous Wind Facility, MO** | 29 | 0.46099032 | 0.311365842 | 0.055177627 | 0.044334975 | 0.000351133 |
| **Anonymous Wind Facility, MO** | 30 | 0.46099032 | 0.30737352 | 0.055177627 | 0.044334975 | 0.000346631 |
| **Anonymous Wind Facility, MO** | 31 | 0.46099032 | 0.300426121 | 0.055177627 | 0.044334975 | 0.000338797 |
| **Anonymous Wind Facility, MO** | 32 | 0.46099032 | 0.294637693 | 0.055177627 | 0.044334975 | 0.000332269 |
| **Anonymous Wind Facility, MO** | 33 | 0.46099032 | 0.29008742 | 0.055177627 | 0.044334975 | 0.000327137 |
| **Anonymous Wind Facility, MO** | 34 | 0.46099032 | 0.283985059 | 0.055177627 | 0.044334975 | 0.000320256 |
| **Anonymous Wind Facility, MO** | 35 | 0.46099032 | 0.279358219 | 0.055177627 | 0.044334975 | 0.000315038 |
| **Anonymous Wind Facility, MO** | 36 | 0.46099032 | 0.276872971 | 0.055177627 | 0.044334975 | 0.000312235 |
| **Anonymous Wind Facility, MO** | 37 | 0.46099032 | 0.274301021 | 0.055177627 | 0.044334975 | 0.000309335 |
| **Anonymous Wind Facility, MO** | 38 | 0.46099032 | 0.267841344 | 0.055177627 | 0.044334975 | 0.00030205 |
| **Anonymous Wind Facility, MO** | 39 | 0.46099032 | 0.262929834 | 0.055177627 | 0.044334975 | 0.000296511 |
| **Anonymous Wind Facility, MO** | 40 | 0.46099032 | 0.259679988 | 0.055177627 | 0.044334975 | 0.000292846 |
| **Anonymous Wind Facility, MO** | 41 | 0.46099032 | 0.256731404 | 0.055177627 | 0.044334975 | 0.000289521 |
| **Anonymous Wind Facility, MO** | 42 | 0.46099032 | 0.255955461 | 0.055177627 | 0.044334975 | 0.000288646 |
| **Anonymous Wind Facility, MO** | 43 | 0.46099032 | 0.255232491 | 0.055177627 | 0.044334975 | 0.000287831 |
| **Anonymous Wind Facility, MO** | 44 | 0.46099032 | 0.25709045 | 0.055177627 | 0.044334975 | 0.000289926 |
| **Anonymous Wind Facility, MO** | 45 | 0.46099032 | 0.259082963 | 0.055177627 | 0.044334975 | 0.000292173 |
| **Anonymous Wind Facility, MO** | 46 | 0.46099032 | 0.261285312 | 0.055177627 | 0.044334975 | 0.000294657 |
| **Anonymous Wind Facility, MO** | 47 | 0.46099032 | 0.26095822 | 0.055177627 | 0.044334975 | 0.000294288 |
| **Anonymous Wind Facility, MO** | 48 | 0.46099032 | 0.260836067 | 0.055177627 | 0.044334975 | 0.00029415 |
| **Anonymous Wind Facility, MO** | 49 | 0.46099032 | 0.258599683 | 0.055177627 | 0.044334975 | 0.000291628 |
| **Anonymous Wind Facility, MO** | 50 | 0.46099032 | 0.255609373 | 0.055177627 | 0.044334975 | 0.000288256 |
| **Anonymous Wind Facility, MO** | 51 | 0.46099032 | 0.251686333 | 0.055177627 | 0.044334975 | 0.000283832 |
| **Anonymous Wind Facility, MO** | 52 | 0.46099032 | 0.25068691 | 0.055177627 | 0.044334975 | 0.000282705 |
| **Anonymous Wind Facility, MO** | 53 | 0.46099032 | 0.25186677 | 0.055177627 | 0.044334975 | 0.000284035 |
| **Anonymous Wind Facility, MO** | 54 | 0.46099032 | 0.250238477 | 0.055177627 | 0.044334975 | 0.000282199 |
| **Anonymous Wind Facility, MO** | 55 | 0.46099032 | 0.247610958 | 0.055177627 | 0.044334975 | 0.000279236 |
| **Anonymous Wind Facility, MO** | 56 | 0.46099032 | 0.246059544 | 0.055177627 | 0.044334975 | 0.000277486 |
| **Anonymous Wind Facility, MO** | 57 | 0.46099032 | 0.243870369 | 0.055177627 | 0.044334975 | 0.000275018 |
| **Anonymous Wind Facility, MO** | 58 | 0.46099032 | 0.240984489 | 0.055177627 | 0.044334975 | 0.000271763 |
| **Anonymous Wind Facility, MO** | 59 | 0.46099032 | 0.239381304 | 0.055177627 | 0.044334975 | 0.000269955 |
| **Anonymous Wind Facility, MO** | 60 | 0.46099032 | 0.239510947 | 0.055177627 | 0.044334975 | 0.000270101 |
| **Anonymous Wind Facility, MO** | 61 | 0.46099032 | 0.24150931 | 0.055177627 | 0.044334975 | 0.000272355 |
| **Anonymous Wind Facility, MO** | 62 | 0.46099032 | 0.240791943 | 0.055177627 | 0.044334975 | 0.000271546 |
| **Anonymous Wind Facility, MO** | 63 | 0.46099032 | 0.238333866 | 0.055177627 | 0.044334975 | 0.000268774 |
| **Anonymous Wind Facility, MO** | 64 | 0.46099032 | 0.236676641 | 0.055177627 | 0.044334975 | 0.000266905 |
| **Anonymous Wind Facility, MO** | 65 | 0.46099032 | 0.235553888 | 0.055177627 | 0.044334975 | 0.000265639 |
| **Anonymous Wind Facility, MO** | 66 | 0.46099032 | 0.231356529 | 0.055177627 | 0.044334975 | 0.000260905 |
| **Anonymous Wind Facility, MO** | 67 | 0.46099032 | 0.229440157 | 0.055177627 | 0.044334975 | 0.000258744 |
| **Anonymous Wind Facility, MO** | 68 | 0.46099032 | 0.228103421 | 0.055177627 | 0.044334975 | 0.000257237 |
| **Anonymous Wind Facility, MO** | 69 | 0.46099032 | 0.226174392 | 0.055177627 | 0.044334975 | 0.000255061 |
| **Anonymous Wind Facility, MO** | 70 | 0.46099032 | 0.223394068 | 0.055177627 | 0.044334975 | 0.000251926 |
| **Anonymous Wind Facility, MO** | 71 | 0.46099032 | 0.221815606 | 0.055177627 | 0.044334975 | 0.000250146 |
| **Anonymous Wind Facility, MO** | 72 | 0.46099032 | 0.21850862 | 0.055177627 | 0.044334975 | 0.000246417 |
| **Anonymous Wind Facility, MO** | 73 | 0.46099032 | 0.217778913 | 0.055177627 | 0.044334975 | 0.000245594 |
| **Anonymous Wind Facility, MO** | 74 | 0.46099032 | 0.217405685 | 0.055177627 | 0.044334975 | 0.000245173 |
| **Anonymous Wind Facility, MO** | 75 | 0.46099032 | 0.215455867 | 0.055177627 | 0.044334975 | 0.000242974 |
| **Anonymous Wind Facility, MO** | 76 | 0.46099032 | 0.215640164 | 0.055177627 | 0.044334975 | 0.000243182 |
| **Anonymous Wind Facility, MO** | 77 | 0.46099032 | 0.216920259 | 0.055177627 | 0.044334975 | 0.000244625 |
| **Anonymous Wind Facility, MO** | 78 | 0.46099032 | 0.214780108 | 0.055177627 | 0.044334975 | 0.000242212 |
| **Anonymous Wind Facility, MO** | 79 | 0.46099032 | 0.213595986 | 0.055177627 | 0.044334975 | 0.000240876 |
| **Anonymous Wind Facility, MO** | 80 | 0.46099032 | 0.214126288 | 0.055177627 | 0.044334975 | 0.000241475 |
| **Anonymous Wind Facility, MO** | 81 | 0.46099032 | 0.212386642 | 0.055177627 | 0.044334975 | 0.000239513 |
| **Anonymous Wind Facility, MO** | 82 | 0.46099032 | 0.208443956 | 0.055177627 | 0.044334975 | 0.000235066 |
| **Anonymous Wind Facility, MO** | 83 | 0.46099032 | 0.207037445 | 0.055177627 | 0.044334975 | 0.00023348 |
| **Anonymous Wind Facility, MO** | 84 | 0.46099032 | 0.206734106 | 0.055177627 | 0.044334975 | 0.000233138 |
| **Anonymous Wind Facility, MO** | 85 | 0.46099032 | 0.205306479 | 0.055177627 | 0.044334975 | 0.000231528 |
| **Anonymous Wind Facility, MO** | 86 | 0.46099032 | 0.202342503 | 0.055177627 | 0.044334975 | 0.000228186 |
| **Anonymous Wind Facility, MO** | 87 | 0.46099032 | 0.200813344 | 0.055177627 | 0.044334975 | 0.000226461 |
| **Anonymous Wind Facility, MO** | 88 | 0.46099032 | 0.198831063 | 0.055177627 | 0.044334975 | 0.000224226 |
| **Anonymous Wind Facility, MO** | 89 | 0.46099032 | 0.194868082 | 0.055177627 | 0.044334975 | 0.000219757 |
| **Anonymous Wind Facility, MO** | 90 | 0.46099032 | 0.190621883 | 0.055177627 | 0.044334975 | 0.000214968 |
| **Anonymous Wind Facility, MO** | 91 | 0.46099032 | 0.186738158 | 0.055177627 | 0.044334975 | 0.000210588 |
| **Anonymous Wind Facility, MO** | 92 | 0.46099032 | 0.183481211 | 0.055177627 | 0.044334975 | 0.000206915 |
| **Anonymous Wind Facility, MO** | 93 | 0.46099032 | 0.179279906 | 0.055177627 | 0.044334975 | 0.000202178 |
| **Anonymous Wind Facility, MO** | 94 | 0.46099032 | 0.175276659 | 0.055177627 | 0.044334975 | 0.000197663 |
| **Anonymous Wind Facility, MO** | 95 | 0.46099032 | 0.170937282 | 0.055177627 | 0.044334975 | 0.000192769 |
| **Anonymous Wind Facility, MO** | 96 | 0.46099032 | 0.167327625 | 0.055177627 | 0.044334975 | 0.000188699 |
| **Anonymous Wind Facility, MO** | 97 | 0.46099032 | 0.162888078 | 0.055177627 | 0.044334975 | 0.000183692 |
| **Anonymous Wind Facility, MO** | 98 | 0.46099032 | 0.160073833 | 0.055177627 | 0.044334975 | 0.000180518 |
| **Anonymous Wind Facility, MO** | 99 | 0.46099032 | 0.156378471 | 0.055177627 | 0.044334975 | 0.000176351 |
| **Anonymous Wind Facility, MO** | 100 | 0.46099032 | 0.151413723 | 0.055177627 | 0.044334975 | 0.000170752 |
| **Anonymous Wind Facility, MO** | 101 | 0.46099032 | 0.147659607 | 0.055177627 | 0.044334975 | 0.000166519 |
| **Anonymous Wind Facility, MO** | 102 | 0.46099032 | 0.144176569 | 0.055177627 | 0.044334975 | 0.000162591 |
| **Anonymous Wind Facility, MO** | 103 | 0.46099032 | 0.139728505 | 0.055177627 | 0.044334975 | 0.000157575 |
| **Anonymous Wind Facility, MO** | 104 | 0.46099032 | 0.135754582 | 0.055177627 | 0.044334975 | 0.000153093 |
| **Anonymous Wind Facility, MO** | 105 | 0.46099032 | 0.131490982 | 0.055177627 | 0.044334975 | 0.000148285 |
| **Anonymous Wind Facility, MO** | 106 | 0.46099032 | 0.126899313 | 0.055177627 | 0.044334975 | 0.000143107 |
| **Anonymous Wind Facility, MO** | 107 | 0.46099032 | 0.122423145 | 0.055177627 | 0.044334975 | 0.000138059 |
| **Anonymous Wind Facility, MO** | 108 | 0.46099032 | 0.117964973 | 0.055177627 | 0.044334975 | 0.000133031 |
| **Anonymous Wind Facility, MO** | 109 | 0.46099032 | 0.115078975 | 0.055177627 | 0.044334975 | 0.000129777 |
| **Anonymous Wind Facility, MO** | 110 | 0.46099032 | 0.11156707 | 0.055177627 | 0.044334975 | 0.000125816 |
| **Anonymous Wind Facility, MO** | 111 | 0.46099032 | 0.10750837 | 0.055177627 | 0.044334975 | 0.000121239 |
| **Anonymous Wind Facility, MO** | 112 | 0.46099032 | 0.103152616 | 0.055177627 | 0.044334975 | 0.000116327 |
| **Anonymous Wind Facility, MO** | 113 | 0.46099032 | 0.100764296 | 0.055177627 | 0.044334975 | 0.000113634 |
| **Anonymous Wind Facility, MO** | 114 | 0.46099032 | 0.098262689 | 0.055177627 | 0.044334975 | 0.000110813 |
| **Anonymous Wind Facility, MO** | 115 | 0.46099032 | 0.096047721 | 0.055177627 | 0.044334975 | 0.000108315 |
| **Anonymous Wind Facility, MO** | 116 | 0.46099032 | 0.092645339 | 0.055177627 | 0.044334975 | 0.000104478 |
| **Anonymous Wind Facility, MO** | 117 | 0.46099032 | 0 | 0.055177627 | 0.044334975 | 6.64E-10 |
| **Anonymous Wind Facility, MO** | 118 | 0.46099032 | 0 | 0.055177627 | 0.044334975 | 6.64E-10 |
| **Anonymous Wind Facility, MO** | 119 | 0.46099032 | 0 | 0.055177627 | 0.044334975 | 6.64E-10 |
| **Anonymous Wind Facility, MO** | 120 | 0.46099032 | 0 | 0.055177627 | 0.044334975 | 6.64E-10 |
| **Anonymous Wind Facility, MO** | 121 | 0.46099032 | 0 | 0.055177627 | 0.044334975 | 6.64E-10 |
| **Anonymous Wind Facility, MO** | 122 | 0.46099032 | 0 | 0.055177627 | 0.044334975 | 6.64E-10 |
| **Anonymous Wind Facility, MO** | 123 | 0.46099032 | 0 | 0.055177627 | 0.044334975 | 6.64E-10 |
| **Anonymous Wind Facility, MO** | 124 | 0.46099032 | 0 | 0.055177627 | 0.044334975 | 6.64E-10 |
| **Anonymous Wind Facility, MO** | 125 | 0.46099032 | 0 | 0.055177627 | 0.044334975 | 6.64E-10 |
| **Anonymous Wind Facility, MO** | 126 | 0.46099032 | 0 | 0.055177627 | 0.044334975 | 6.64E-10 |
| **Anonymous Wind Facility, MO** | 127 | 0.46099032 | 0 | 0.055177627 | 0.044334975 | 6.64E-10 |
| **Anonymous Wind Facility, MO** | 128 | 0.46099032 | 0 | 0.055177627 | 0.044334975 | 6.64E-10 |
| **Anonymous Wind Facility, MO** | 129 | 0.46099032 | 0 | 0.055177627 | 0.044334975 | 6.64E-10 |
| **Anonymous Wind Facility, MO** | 130 | 0.46099032 | 0 | 0.055177627 | 0.044334975 | 6.64E-10 |
| **Anonymous Wind Facility, MO** | 131 | 0.46099032 | 0 | 0.055177627 | 0.044334975 | 6.64E-10 |
| **Anonymous Wind Facility, MO** | 132 | 0.46099032 | 0 | 0.055177627 | 0.044334975 | 6.64E-10 |
| **Anonymous Wind Facility, MO** | 133 | 0.46099032 | 0 | 0.055177627 | 0.044334975 | 6.64E-10 |
| **Anonymous Wind Facility, MO** | 134 | 0.46099032 | 0 | 0.055177627 | 0.044334975 | 6.64E-10 |
| **Anonymous Wind Facility, MO** | 135 | 0.46099032 | 0 | 0.055177627 | 0.044334975 | 6.64E-10 |
| **Anonymous Wind Facility, MO** | 136 | 0.46099032 | 0 | 0.055177627 | 0.044334975 | 6.64E-10 |
| **Anonymous Wind Facility, MO** | 137 | 0.46099032 | 0 | 0.055177627 | 0.044334975 | 6.64E-10 |
| **Anonymous Wind Facility, MO** | 138 | 0.46099032 | 0 | 0.055177627 | 0.044334975 | 6.64E-10 |
| **Anonymous Wind Facility, MO** | 139 | 0.46099032 | 0 | 0.055177627 | 0.044334975 | 6.64E-10 |
| **Anonymous Wind Facility, MO** | 140 | 0.46099032 | 0 | 0.055177627 | 0.044334975 | 6.64E-10 |
| **Anonymous Wind Facility, MO** | 141 | 0.46099032 | 0 | 0.055177627 | 0.044334975 | 6.64E-10 |
| **Anonymous Wind Facility, MO** | 142 | 0.46099032 | 0 | 0.055177627 | 0.044334975 | 6.64E-10 |
| **Anonymous Wind Facility, MO** | 143 | 0.46099032 | 0 | 0.055177627 | 0.044334975 | 6.64E-10 |
| **Anonymous Wind Facility, MO** | 144 | 0.46099032 | 0 | 0.055177627 | 0.044334975 | 6.64E-10 |
| **Anonymous Wind Facility, NH (2013)** | 3 | 0.83662488 | 0.999835515 | 0.01814059 | 0.02955665 | 0.000448504 |
| **Anonymous Wind Facility, NH (2013)** | 4 | 0.83662488 | 0.998061175 | 0.01814059 | 0.02955665 | 0.000447708 |
| **Anonymous Wind Facility, NH (2013)** | 5 | 0.83662488 | 0.984584077 | 0.01814059 | 0.02955665 | 0.000441662 |
| **Anonymous Wind Facility, NH (2013)** | 6 | 0.83662488 | 0.974848838 | 0.01814059 | 0.02955665 | 0.000437295 |
| **Anonymous Wind Facility, NH (2013)** | 7 | 0.83662488 | 0.969402847 | 0.01814059 | 0.02955665 | 0.000434852 |
| **Anonymous Wind Facility, NH (2013)** | 8 | 0.83662488 | 0.953314044 | 0.01814059 | 0.02955665 | 0.000427635 |
| **Anonymous Wind Facility, NH (2013)** | 9 | 0.83662488 | 0.931699231 | 0.01814059 | 0.02955665 | 0.000417939 |
| **Anonymous Wind Facility, NH (2013)** | 10 | 0.83662488 | 0.912154438 | 0.01814059 | 0.02955665 | 0.000409172 |
| **Anonymous Wind Facility, NH (2013)** | 11 | 0.83662488 | 0.89250242 | 0.01814059 | 0.02955665 | 0.000400356 |
| **Anonymous Wind Facility, NH (2013)** | 12 | 0.83662488 | 0.864448602 | 0.01814059 | 0.02955665 | 0.000387772 |
| **Anonymous Wind Facility, NH (2013)** | 13 | 0.83662488 | 0.824971108 | 0.01814059 | 0.02955665 | 0.000370063 |
| **Anonymous Wind Facility, NH (2013)** | 14 | 0.83662488 | 0.789385017 | 0.01814059 | 0.02955665 | 0.0003541 |
| **Anonymous Wind Facility, NH (2013)** | 15 | 0.83662488 | 0.745826467 | 0.01814059 | 0.02955665 | 0.000334561 |
| **Anonymous Wind Facility, NH (2013)** | 16 | 0.83662488 | 0.703974927 | 0.01814059 | 0.02955665 | 0.000315787 |
| **Anonymous Wind Facility, NH (2013)** | 17 | 0.83662488 | 0.673844236 | 0.01814059 | 0.02955665 | 0.000302271 |
| **Anonymous Wind Facility, NH (2013)** | 18 | 0.83662488 | 0.645521231 | 0.01814059 | 0.02955665 | 0.000289566 |
| **Anonymous Wind Facility, NH (2013)** | 19 | 0.83662488 | 0.618460725 | 0.01814059 | 0.02955665 | 0.000277428 |
| **Anonymous Wind Facility, NH (2013)** | 20 | 0.83662488 | 0.594780414 | 0.01814059 | 0.02955665 | 0.000266805 |
| **Anonymous Wind Facility, NH (2013)** | 21 | 0.83662488 | 0.577723193 | 0.01814059 | 0.02955665 | 0.000259154 |
| **Anonymous Wind Facility, NH (2013)** | 22 | 0.83662488 | 0.563152717 | 0.01814059 | 0.02955665 | 0.000252618 |
| **Anonymous Wind Facility, NH (2013)** | 23 | 0.83662488 | 0.545637279 | 0.01814059 | 0.02955665 | 0.000244761 |
| **Anonymous Wind Facility, NH (2013)** | 24 | 0.83662488 | 0.524483818 | 0.01814059 | 0.02955665 | 0.000235272 |
| **Anonymous Wind Facility, NH (2013)** | 25 | 0.83662488 | 0.505755874 | 0.01814059 | 0.02955665 | 0.000226871 |
| **Anonymous Wind Facility, NH (2013)** | 26 | 0.83662488 | 0.488117541 | 0.01814059 | 0.02955665 | 0.000218958 |
| **Anonymous Wind Facility, NH (2013)** | 27 | 0.83662488 | 0.468327945 | 0.01814059 | 0.02955665 | 0.000210081 |
| **Anonymous Wind Facility, NH (2013)** | 28 | 0.83662488 | 0.449469106 | 0.01814059 | 0.02955665 | 0.000201622 |
| **Anonymous Wind Facility, NH (2013)** | 29 | 0.83662488 | 0.424337099 | 0.01814059 | 0.02955665 | 0.000190348 |
| **Anonymous Wind Facility, NH (2013)** | 30 | 0.83662488 | 0.406139437 | 0.01814059 | 0.02955665 | 0.000182185 |
| **Anonymous Wind Facility, NH (2013)** | 31 | 0.83662488 | 0.393373381 | 0.01814059 | 0.02955665 | 0.000176458 |
| **Anonymous Wind Facility, NH (2013)** | 32 | 0.83662488 | 0.381921956 | 0.01814059 | 0.02955665 | 0.000171322 |
| **Anonymous Wind Facility, NH (2013)** | 33 | 0.83662488 | 0.36995702 | 0.01814059 | 0.02955665 | 0.000165954 |
| **Anonymous Wind Facility, NH (2013)** | 34 | 0.83662488 | 0.357508561 | 0.01814059 | 0.02955665 | 0.00016037 |
| **Anonymous Wind Facility, NH (2013)** | 35 | 0.83662488 | 0.343712062 | 0.01814059 | 0.02955665 | 0.000154181 |
| **Anonymous Wind Facility, NH (2013)** | 36 | 0.83662488 | 0.32789541 | 0.01814059 | 0.02955665 | 0.000147086 |
| **Anonymous Wind Facility, NH (2013)** | 37 | 0.83662488 | 0.312517875 | 0.01814059 | 0.02955665 | 0.000140188 |
| **Anonymous Wind Facility, NH (2013)** | 38 | 0.83662488 | 0.295513184 | 0.01814059 | 0.02955665 | 0.000132561 |
| **Anonymous Wind Facility, NH (2013)** | 39 | 0.83662488 | 0.282212933 | 0.01814059 | 0.02955665 | 0.000126594 |
| **Anonymous Wind Facility, NH (2013)** | 40 | 0.83662488 | 0.271258399 | 0.01814059 | 0.02955665 | 0.00012168 |
| **Anonymous Wind Facility, NH (2013)** | 41 | 0.83662488 | 0.259122809 | 0.01814059 | 0.02955665 | 0.000116237 |
| **Anonymous Wind Facility, NH (2013)** | 42 | 0.83662488 | 0.245281577 | 0.01814059 | 0.02955665 | 0.000110028 |
| **Anonymous Wind Facility, NH (2013)** | 43 | 0.83662488 | 0.236294767 | 0.01814059 | 0.02955665 | 0.000105996 |
| **Anonymous Wind Facility, NH (2013)** | 44 | 0.83662488 | 0.226457079 | 0.01814059 | 0.02955665 | 0.000101584 |
| **Anonymous Wind Facility, NH (2013)** | 45 | 0.83662488 | 0.217949221 | 0.01814059 | 0.02955665 | 9.78E-05 |
| **Anonymous Wind Facility, NH (2013)** | 46 | 0.83662488 | 0.208742047 | 0.01814059 | 0.02955665 | 9.36E-05 |
| **Anonymous Wind Facility, NH (2013)** | 47 | 0.83662488 | 0.210217986 | 0.01814059 | 0.02955665 | 9.43E-05 |
| **Anonymous Wind Facility, NH (2013)** | 48 | 0.83662488 | 0.200377014 | 0.01814059 | 0.02955665 | 8.99E-05 |
| **Anonymous Wind Facility, NH (2013)** | 49 | 0.83662488 | 0.189797714 | 0.01814059 | 0.02955665 | 8.51E-05 |
| **Anonymous Wind Facility, NH (2013)** | 50 | 0.83662488 | 0.18198284 | 0.01814059 | 0.02955665 | 8.16E-05 |
| **Anonymous Wind Facility, NH (2013)** | 51 | 0.83662488 | 0.174319961 | 0.01814059 | 0.02955665 | 7.82E-05 |
| **Anonymous Wind Facility, NH (2013)** | 52 | 0.83662488 | 0.165749362 | 0.01814059 | 0.02955665 | 7.44E-05 |
| **Anonymous Wind Facility, NH (2013)** | 53 | 0.83662488 | 0.163095288 | 0.01814059 | 0.02955665 | 7.32E-05 |
| **Anonymous Wind Facility, NH (2013)** | 54 | 0.83662488 | 0.161340394 | 0.01814059 | 0.02955665 | 7.24E-05 |
| **Anonymous Wind Facility, NH (2013)** | 55 | 0.83662488 | 0.158048867 | 0.01814059 | 0.02955665 | 7.09E-05 |
| **Anonymous Wind Facility, NH (2013)** | 56 | 0.83662488 | 0.151268895 | 0.01814059 | 0.02955665 | 6.79E-05 |
| **Anonymous Wind Facility, NH (2013)** | 57 | 0.83662488 | 0.143360581 | 0.01814059 | 0.02955665 | 6.43E-05 |
| **Anonymous Wind Facility, NH (2013)** | 58 | 0.83662488 | 0.133881915 | 0.01814059 | 0.02955665 | 6.01E-05 |
| **Anonymous Wind Facility, NH (2013)** | 59 | 0.83662488 | 0.123536466 | 0.01814059 | 0.02955665 | 5.54E-05 |
| **Anonymous Wind Facility, NH (2013)** | 60 | 0.83662488 | 0.114459904 | 0.01814059 | 0.02955665 | 5.13E-05 |
| **Anonymous Wind Facility, NH (2013)** | 61 | 0.83662488 | 0.10834956 | 0.01814059 | 0.02955665 | 4.86E-05 |
| **Anonymous Wind Facility, NH (2013)** | 62 | 0.83662488 | 0.101963029 | 0.01814059 | 0.02955665 | 4.57E-05 |
| **Anonymous Wind Facility, NH (2013)** | 63 | 0.83662488 | 0 | 0.01814059 | 0.02955665 | 6.64E-10 |
| **Anonymous Wind Facility, NH (2013)** | 64 | 0.83662488 | 0 | 0.01814059 | 0.02955665 | 6.64E-10 |
| **Anonymous Wind Facility, NH (2013)** | 65 | 0.83662488 | 0 | 0.01814059 | 0.02955665 | 6.64E-10 |
| **Anonymous Wind Facility, NH (2013)** | 66 | 0.83662488 | 0 | 0.01814059 | 0.02955665 | 6.64E-10 |
| **Anonymous Wind Facility, NH (2013)** | 67 | 0.83662488 | 0 | 0.01814059 | 0.02955665 | 6.64E-10 |
| **Anonymous Wind Facility, NH (2013)** | 68 | 0.83662488 | 0 | 0.01814059 | 0.02955665 | 6.64E-10 |
| **Anonymous Wind Facility, NH (2013)** | 69 | 0.83662488 | 0 | 0.01814059 | 0.02955665 | 6.64E-10 |
| **Anonymous Wind Facility, NH (2013)** | 70 | 0.83662488 | 0 | 0.01814059 | 0.02955665 | 6.64E-10 |
| **Anonymous Wind Facility, NH (2013)** | 71 | 0.83662488 | 0 | 0.01814059 | 0.02955665 | 6.64E-10 |
| **Anonymous Wind Facility, NH (2013)** | 72 | 0.83662488 | 0 | 0.01814059 | 0.02955665 | 6.64E-10 |
| **Anonymous Wind Facility, NH (2013)** | 73 | 0.83662488 | 0 | 0.01814059 | 0.02955665 | 6.64E-10 |
| **Anonymous Wind Facility, NH (2013)** | 74 | 0.83662488 | 0 | 0.01814059 | 0.02955665 | 6.64E-10 |
| **Anonymous Wind Facility, NH (2013)** | 75 | 0.83662488 | 0 | 0.01814059 | 0.02955665 | 6.64E-10 |
| **Anonymous Wind Facility, NH (2013)** | 76 | 0.83662488 | 0 | 0.01814059 | 0.02955665 | 6.64E-10 |
| **Anonymous Wind Facility, NH (2013)** | 77 | 0.83662488 | 0 | 0.01814059 | 0.02955665 | 6.64E-10 |
| **Anonymous Wind Facility, NH (2013)** | 78 | 0.83662488 | 0 | 0.01814059 | 0.02955665 | 6.64E-10 |
| **Anonymous Wind Facility, NH (2013)** | 79 | 0.83662488 | 0 | 0.01814059 | 0.02955665 | 6.64E-10 |
| **Anonymous Wind Facility, NH (2013)** | 80 | 0.83662488 | 0 | 0.01814059 | 0.02955665 | 6.64E-10 |
| **Anonymous Wind Facility, NH (2013)** | 81 | 0.83662488 | 0 | 0.01814059 | 0.02955665 | 6.64E-10 |
| **Anonymous Wind Facility, NH (2013)** | 82 | 0.83662488 | 0 | 0.01814059 | 0.02955665 | 6.64E-10 |
| **Anonymous Wind Facility, NH (2013)** | 83 | 0.83662488 | 0 | 0.01814059 | 0.02955665 | 6.64E-10 |
| **Anonymous Wind Facility, NH (2013)** | 84 | 0.83662488 | 0 | 0.01814059 | 0.02955665 | 6.64E-10 |
| **Anonymous Wind Facility, NH (2013)** | 85 | 0.83662488 | 0 | 0.01814059 | 0.02955665 | 6.64E-10 |
| **Anonymous Wind Facility, NH (2013)** | 86 | 0.83662488 | 0 | 0.01814059 | 0.02955665 | 6.64E-10 |
| **Anonymous Wind Facility, NH (2013)** | 87 | 0.83662488 | 0 | 0.01814059 | 0.02955665 | 6.64E-10 |
| **Anonymous Wind Facility, NH (2013)** | 88 | 0.83662488 | 0 | 0.01814059 | 0.02955665 | 6.64E-10 |
| **Anonymous Wind Facility, NH (2013)** | 89 | 0.83662488 | 0 | 0.01814059 | 0.02955665 | 6.64E-10 |
| **Anonymous Wind Facility, NH (2013)** | 90 | 0.83662488 | 0 | 0.01814059 | 0.02955665 | 6.64E-10 |
| **Anonymous Wind Facility, NH (2013)** | 91 | 0.83662488 | 0 | 0.01814059 | 0.02955665 | 6.64E-10 |
| **Anonymous Wind Facility, NH (2013)** | 92 | 0.83662488 | 0 | 0.01814059 | 0.02955665 | 6.64E-10 |
| **Anonymous Wind Facility, NH (2013)** | 93 | 0.83662488 | 0 | 0.01814059 | 0.02955665 | 6.64E-10 |
| **Anonymous Wind Facility, NH (2013)** | 94 | 0.83662488 | 0 | 0.01814059 | 0.02955665 | 6.64E-10 |
| **Anonymous Wind Facility, NH (2013)** | 95 | 0.83662488 | 0 | 0.01814059 | 0.02955665 | 6.64E-10 |
| **Anonymous Wind Facility, NH (2013)** | 96 | 0.83662488 | 0 | 0.01814059 | 0.02955665 | 6.64E-10 |
| **Anonymous Wind Facility, NH (2013)** | 97 | 0.83662488 | 0 | 0.01814059 | 0.02955665 | 6.64E-10 |
| **Anonymous Wind Facility, NH (2013)** | 98 | 0.83662488 | 0 | 0.01814059 | 0.02955665 | 6.64E-10 |
| **Anonymous Wind Facility, NH (2013)** | 99 | 0.83662488 | 0 | 0.01814059 | 0.02955665 | 6.64E-10 |
| **Anonymous Wind Facility, NH (2013)** | 100 | 0.83662488 | 0 | 0.01814059 | 0.02955665 | 6.64E-10 |
| **Anonymous Wind Facility, NH (2013)** | 101 | 0.83662488 | 0 | 0.01814059 | 0.02955665 | 6.64E-10 |
| **Anonymous Wind Facility, NH (2013)** | 102 | 0.83662488 | 0 | 0.01814059 | 0.02955665 | 6.64E-10 |
| **Anonymous Wind Facility, NH (2013)** | 103 | 0.83662488 | 0 | 0.01814059 | 0.02955665 | 6.64E-10 |
| **Anonymous Wind Facility, NH (2013)** | 104 | 0.83662488 | 0 | 0.01814059 | 0.02955665 | 6.64E-10 |
| **Anonymous Wind Facility, NH (2013)** | 105 | 0.83662488 | 0 | 0.01814059 | 0.02955665 | 6.64E-10 |
| **Anonymous Wind Facility, NH (2013)** | 106 | 0.83662488 | 0 | 0.01814059 | 0.02955665 | 6.64E-10 |
| **Anonymous Wind Facility, NH (2013)** | 107 | 0.83662488 | 0 | 0.01814059 | 0.02955665 | 6.64E-10 |
| **Anonymous Wind Facility, NH (2013)** | 108 | 0.83662488 | 0 | 0.01814059 | 0.02955665 | 6.64E-10 |
| **Anonymous Wind Facility, NH (2013)** | 109 | 0.83662488 | 0 | 0.01814059 | 0.02955665 | 6.64E-10 |
| **Anonymous Wind Facility, NH (2013)** | 110 | 0.83662488 | 0 | 0.01814059 | 0.02955665 | 6.64E-10 |
| **Anonymous Wind Facility, NH (2013)** | 111 | 0.83662488 | 0 | 0.01814059 | 0.02955665 | 6.64E-10 |
| **Anonymous Wind Facility, NH (2013)** | 112 | 0.83662488 | 0 | 0.01814059 | 0.02955665 | 6.64E-10 |
| **Anonymous Wind Facility, NH (2013)** | 113 | 0.83662488 | 0 | 0.01814059 | 0.02955665 | 6.64E-10 |
| **Anonymous Wind Facility, NH (2013)** | 114 | 0.83662488 | 0 | 0.01814059 | 0.02955665 | 6.64E-10 |
| **Anonymous Wind Facility, NH (2013)** | 115 | 0.83662488 | 0 | 0.01814059 | 0.02955665 | 6.64E-10 |
| **Anonymous Wind Facility, NH (2013)** | 116 | 0.83662488 | 0 | 0.01814059 | 0.02955665 | 6.64E-10 |
| **Anonymous Wind Facility, NH (2013)** | 117 | 0.83662488 | 0 | 0.01814059 | 0.02955665 | 6.64E-10 |
| **Anonymous Wind Facility, NH (2013)** | 118 | 0.83662488 | 0 | 0.01814059 | 0.02955665 | 6.64E-10 |
| **Anonymous Wind Facility, NH (2013)** | 119 | 0.83662488 | 0 | 0.01814059 | 0.02955665 | 6.64E-10 |
| **Anonymous Wind Facility, NH (2013)** | 120 | 0.83662488 | 0 | 0.01814059 | 0.02955665 | 6.64E-10 |
| **Anonymous Wind Facility, NH (2013)** | 121 | 0.83662488 | 0 | 0.01814059 | 0.02955665 | 6.64E-10 |
| **Anonymous Wind Facility, NH (2013)** | 122 | 0.83662488 | 0 | 0.01814059 | 0.02955665 | 6.64E-10 |
| **Anonymous Wind Facility, NH (2013)** | 123 | 0.83662488 | 0 | 0.01814059 | 0.02955665 | 6.64E-10 |
| **Anonymous Wind Facility, NH (2013)** | 124 | 0.83662488 | 0 | 0.01814059 | 0.02955665 | 6.64E-10 |
| **Anonymous Wind Facility, NH (2013)** | 125 | 0.83662488 | 0 | 0.01814059 | 0.02955665 | 6.64E-10 |
| **Anonymous Wind Facility, NH (2013)** | 126 | 0.83662488 | 0 | 0.01814059 | 0.02955665 | 6.64E-10 |
| **Anonymous Wind Facility, NH (2013)** | 127 | 0.83662488 | 0 | 0.01814059 | 0.02955665 | 6.64E-10 |
| **Anonymous Wind Facility, NH (2013)** | 128 | 0.83662488 | 0 | 0.01814059 | 0.02955665 | 6.64E-10 |
| **Anonymous Wind Facility, NH (2013)** | 129 | 0.83662488 | 0 | 0.01814059 | 0.02955665 | 6.64E-10 |
| **Anonymous Wind Facility, NH (2013)** | 130 | 0.83662488 | 0 | 0.01814059 | 0.02955665 | 6.64E-10 |
| **Anonymous Wind Facility, NH (2013)** | 131 | 0.83662488 | 0 | 0.01814059 | 0.02955665 | 6.64E-10 |
| **Anonymous Wind Facility, NH (2013)** | 132 | 0.83662488 | 0 | 0.01814059 | 0.02955665 | 6.64E-10 |
| **Anonymous Wind Facility, NH (2013)** | 133 | 0.83662488 | 0 | 0.01814059 | 0.02955665 | 6.64E-10 |
| **Anonymous Wind Facility, NH (2013)** | 134 | 0.83662488 | 0 | 0.01814059 | 0.02955665 | 6.64E-10 |
| **Anonymous Wind Facility, NH (2013)** | 135 | 0.83662488 | 0 | 0.01814059 | 0.02955665 | 6.64E-10 |
| **Anonymous Wind Facility, NH (2013)** | 136 | 0.83662488 | 0 | 0.01814059 | 0.02955665 | 6.64E-10 |
| **Anonymous Wind Facility, NH (2013)** | 137 | 0.83662488 | 0 | 0.01814059 | 0.02955665 | 6.64E-10 |
| **Anonymous Wind Facility, NH (2013)** | 138 | 0.83662488 | 0 | 0.01814059 | 0.02955665 | 6.64E-10 |
| **Anonymous Wind Facility, NH (2013)** | 139 | 0.83662488 | 0 | 0.01814059 | 0.02955665 | 6.64E-10 |
| **Anonymous Wind Facility, NH (2013)** | 140 | 0.83662488 | 0 | 0.01814059 | 0.02955665 | 6.64E-10 |
| **Anonymous Wind Facility, NH (2013)** | 141 | 0.83662488 | 0 | 0.01814059 | 0.02955665 | 6.64E-10 |
| **Anonymous Wind Facility, NH (2013)** | 142 | 0.83662488 | 0 | 0.01814059 | 0.02955665 | 6.64E-10 |
| **Anonymous Wind Facility, NH (2013)** | 143 | 0.83662488 | 0 | 0.01814059 | 0.02955665 | 6.64E-10 |
| **Anonymous Wind Facility, NH (2013)** | 144 | 0.83662488 | 0 | 0.01814059 | 0.02955665 | 6.64E-10 |
| **Anonymous Wind Facility, NH (2015)** | 3 | 0.46988293 | 0.999835515 | 0.01814059 | 0.108374384 | 0.000923626 |
| **Anonymous Wind Facility, NH (2015)** | 4 | 0.46988293 | 0.999502286 | 0.01814059 | 0.108374384 | 0.000923318 |
| **Anonymous Wind Facility, NH (2015)** | 5 | 0.46988293 | 0.98970658 | 0.01814059 | 0.108374384 | 0.000914269 |
| **Anonymous Wind Facility, NH (2015)** | 6 | 0.46988293 | 0.978052951 | 0.01814059 | 0.108374384 | 0.000903504 |
| **Anonymous Wind Facility, NH (2015)** | 7 | 0.46988293 | 0.966761667 | 0.01814059 | 0.108374384 | 0.000893073 |
| **Anonymous Wind Facility, NH (2015)** | 8 | 0.46988293 | 0.9476349 | 0.01814059 | 0.108374384 | 0.000875404 |
| **Anonymous Wind Facility, NH (2015)** | 9 | 0.46988293 | 0.931848284 | 0.01814059 | 0.108374384 | 0.000860821 |
| **Anonymous Wind Facility, NH (2015)** | 10 | 0.46988293 | 0.918053934 | 0.01814059 | 0.108374384 | 0.000848078 |
| **Anonymous Wind Facility, NH (2015)** | 11 | 0.46988293 | 0.902666355 | 0.01814059 | 0.108374384 | 0.000833864 |
| **Anonymous Wind Facility, NH (2015)** | 12 | 0.46988293 | 0.888083749 | 0.01814059 | 0.108374384 | 0.000820392 |
| **Anonymous Wind Facility, NH (2015)** | 13 | 0.46988293 | 0.871464951 | 0.01814059 | 0.108374384 | 0.00080504 |
| **Anonymous Wind Facility, NH (2015)** | 14 | 0.46988293 | 0.842619573 | 0.01814059 | 0.108374384 | 0.000778394 |
| **Anonymous Wind Facility, NH (2015)** | 15 | 0.46988293 | 0.803000003 | 0.01814059 | 0.108374384 | 0.000741794 |
| **Anonymous Wind Facility, NH (2015)** | 16 | 0.46988293 | 0.763403301 | 0.01814059 | 0.108374384 | 0.000705215 |
| **Anonymous Wind Facility, NH (2015)** | 17 | 0.46988293 | 0.722579685 | 0.01814059 | 0.108374384 | 0.000667503 |
| **Anonymous Wind Facility, NH (2015)** | 18 | 0.46988293 | 0.697582779 | 0.01814059 | 0.108374384 | 0.000644412 |
| **Anonymous Wind Facility, NH (2015)** | 19 | 0.46988293 | 0.673701063 | 0.01814059 | 0.108374384 | 0.00062235 |
| **Anonymous Wind Facility, NH (2015)** | 20 | 0.46988293 | 0.65217286 | 0.01814059 | 0.108374384 | 0.000602463 |
| **Anonymous Wind Facility, NH (2015)** | 21 | 0.46988293 | 0.637247883 | 0.01814059 | 0.108374384 | 0.000588676 |
| **Anonymous Wind Facility, NH (2015)** | 22 | 0.46988293 | 0.621452341 | 0.01814059 | 0.108374384 | 0.000574084 |
| **Anonymous Wind Facility, NH (2015)** | 23 | 0.46988293 | 0.597394523 | 0.01814059 | 0.108374384 | 0.00055186 |
| **Anonymous Wind Facility, NH (2015)** | 24 | 0.46988293 | 0.576522624 | 0.01814059 | 0.108374384 | 0.000532579 |
| **Anonymous Wind Facility, NH (2015)** | 25 | 0.46988293 | 0.56353158 | 0.01814059 | 0.108374384 | 0.000520578 |
| **Anonymous Wind Facility, NH (2015)** | 26 | 0.46988293 | 0.544981848 | 0.01814059 | 0.108374384 | 0.000503442 |
| **Anonymous Wind Facility, NH (2015)** | 27 | 0.46988293 | 0.522187566 | 0.01814059 | 0.108374384 | 0.000482385 |
| **Anonymous Wind Facility, NH (2015)** | 28 | 0.46988293 | 0.500043755 | 0.01814059 | 0.108374384 | 0.00046193 |
| **Anonymous Wind Facility, NH (2015)** | 29 | 0.46988293 | 0.478613878 | 0.01814059 | 0.108374384 | 0.000442133 |
| **Anonymous Wind Facility, NH (2015)** | 30 | 0.46988293 | 0.464876093 | 0.01814059 | 0.108374384 | 0.000429442 |
| **Anonymous Wind Facility, NH (2015)** | 31 | 0.46988293 | 0.450386125 | 0.01814059 | 0.108374384 | 0.000416057 |
| **Anonymous Wind Facility, NH (2015)** | 32 | 0.46988293 | 0.436564043 | 0.01814059 | 0.108374384 | 0.000403288 |
| **Anonymous Wind Facility, NH (2015)** | 33 | 0.46988293 | 0.424563781 | 0.01814059 | 0.108374384 | 0.000392203 |
| **Anonymous Wind Facility, NH (2015)** | 34 | 0.46988293 | 0.413702973 | 0.01814059 | 0.108374384 | 0.00038217 |
| **Anonymous Wind Facility, NH (2015)** | 35 | 0.46988293 | 0.400592436 | 0.01814059 | 0.108374384 | 0.000370059 |
| **Anonymous Wind Facility, NH (2015)** | 36 | 0.46988293 | 0.386326707 | 0.01814059 | 0.108374384 | 0.00035688 |
| **Anonymous Wind Facility, NH (2015)** | 37 | 0.46988293 | 0.373159011 | 0.01814059 | 0.108374384 | 0.000344716 |
| **Anonymous Wind Facility, NH (2015)** | 38 | 0.46988293 | 0.360120514 | 0.01814059 | 0.108374384 | 0.000332671 |
| **Anonymous Wind Facility, NH (2015)** | 39 | 0.46988293 | 0.342875907 | 0.01814059 | 0.108374384 | 0.000316741 |
| **Anonymous Wind Facility, NH (2015)** | 40 | 0.46988293 | 0.324640123 | 0.01814059 | 0.108374384 | 0.000299895 |
| **Anonymous Wind Facility, NH (2015)** | 41 | 0.46988293 | 0.306263536 | 0.01814059 | 0.108374384 | 0.00028292 |
| **Anonymous Wind Facility, NH (2015)** | 42 | 0.46988293 | 0.286617348 | 0.01814059 | 0.108374384 | 0.000264771 |
| **Anonymous Wind Facility, NH (2015)** | 43 | 0.46988293 | 0.271151333 | 0.01814059 | 0.108374384 | 0.000250484 |
| **Anonymous Wind Facility, NH (2015)** | 44 | 0.46988293 | 0.261559307 | 0.01814059 | 0.108374384 | 0.000241623 |
| **Anonymous Wind Facility, NH (2015)** | 45 | 0.46988293 | 0.253549603 | 0.01814059 | 0.108374384 | 0.000234224 |
| **Anonymous Wind Facility, NH (2015)** | 46 | 0.46988293 | 0.255294611 | 0.01814059 | 0.108374384 | 0.000235836 |
| **Anonymous Wind Facility, NH (2015)** | 47 | 0.46988293 | 0.24551764 | 0.01814059 | 0.108374384 | 0.000226804 |
| **Anonymous Wind Facility, NH (2015)** | 48 | 0.46988293 | 0.238688861 | 0.01814059 | 0.108374384 | 0.000220496 |
| **Anonymous Wind Facility, NH (2015)** | 49 | 0.46988293 | 0.232634857 | 0.01814059 | 0.108374384 | 0.000214903 |
| **Anonymous Wind Facility, NH (2015)** | 50 | 0.46988293 | 0.223253794 | 0.01814059 | 0.108374384 | 0.000206237 |
| **Anonymous Wind Facility, NH (2015)** | 51 | 0.46988293 | 0.216405527 | 0.01814059 | 0.108374384 | 0.000199911 |
| **Anonymous Wind Facility, NH (2015)** | 52 | 0.46988293 | 0.20580162 | 0.01814059 | 0.108374384 | 0.000190115 |
| **Anonymous Wind Facility, NH (2015)** | 53 | 0.46988293 | 0.195883079 | 0.01814059 | 0.108374384 | 0.000180953 |
| **Anonymous Wind Facility, NH (2015)** | 54 | 0.46988293 | 0.188385257 | 0.01814059 | 0.108374384 | 0.000174026 |
| **Anonymous Wind Facility, NH (2015)** | 55 | 0.46988293 | 0.178761408 | 0.01814059 | 0.108374384 | 0.000165136 |
| **Anonymous Wind Facility, NH (2015)** | 56 | 0.46988293 | 0.167473675 | 0.01814059 | 0.108374384 | 0.000154709 |
| **Anonymous Wind Facility, NH (2015)** | 57 | 0.46988293 | 0.157323246 | 0.01814059 | 0.108374384 | 0.000145332 |
| **Anonymous Wind Facility, NH (2015)** | 58 | 0.46988293 | 0.149509544 | 0.01814059 | 0.108374384 | 0.000138114 |
| **Anonymous Wind Facility, NH (2015)** | 59 | 0.46988293 | 0.141980599 | 0.01814059 | 0.108374384 | 0.000131159 |
| **Anonymous Wind Facility, NH (2015)** | 60 | 0.46988293 | 0.134684605 | 0.01814059 | 0.108374384 | 0.000124419 |
| **Anonymous Wind Facility, NH (2015)** | 61 | 0.46988293 | 0.12889889 | 0.01814059 | 0.108374384 | 0.000119074 |
| **Anonymous Wind Facility, NH (2015)** | 62 | 0.46988293 | 0.120911556 | 0.01814059 | 0.108374384 | 0.000111695 |
| **Anonymous Wind Facility, NH (2015)** | 63 | 0.46988293 | 0 | 0.01814059 | 0.108374384 | 6.64E-10 |
| **Anonymous Wind Facility, NH (2015)** | 64 | 0.46988293 | 0 | 0.01814059 | 0.108374384 | 6.64E-10 |
| **Anonymous Wind Facility, NH (2015)** | 65 | 0.46988293 | 0 | 0.01814059 | 0.108374384 | 6.64E-10 |
| **Anonymous Wind Facility, NH (2015)** | 66 | 0.46988293 | 0 | 0.01814059 | 0.108374384 | 6.64E-10 |
| **Anonymous Wind Facility, NH (2015)** | 67 | 0.46988293 | 0 | 0.01814059 | 0.108374384 | 6.64E-10 |
| **Anonymous Wind Facility, NH (2015)** | 68 | 0.46988293 | 0 | 0.01814059 | 0.108374384 | 6.64E-10 |
| **Anonymous Wind Facility, NH (2015)** | 69 | 0.46988293 | 0 | 0.01814059 | 0.108374384 | 6.64E-10 |
| **Anonymous Wind Facility, NH (2015)** | 70 | 0.46988293 | 0 | 0.01814059 | 0.108374384 | 6.64E-10 |
| **Anonymous Wind Facility, NH (2015)** | 71 | 0.46988293 | 0 | 0.01814059 | 0.108374384 | 6.64E-10 |
| **Anonymous Wind Facility, NH (2015)** | 72 | 0.46988293 | 0 | 0.01814059 | 0.108374384 | 6.64E-10 |
| **Anonymous Wind Facility, NH (2015)** | 73 | 0.46988293 | 0 | 0.01814059 | 0.108374384 | 6.64E-10 |
| **Anonymous Wind Facility, NH (2015)** | 74 | 0.46988293 | 0 | 0.01814059 | 0.108374384 | 6.64E-10 |
| **Anonymous Wind Facility, NH (2015)** | 75 | 0.46988293 | 0 | 0.01814059 | 0.108374384 | 6.64E-10 |
| **Anonymous Wind Facility, NH (2015)** | 76 | 0.46988293 | 0 | 0.01814059 | 0.108374384 | 6.64E-10 |
| **Anonymous Wind Facility, NH (2015)** | 77 | 0.46988293 | 0 | 0.01814059 | 0.108374384 | 6.64E-10 |
| **Anonymous Wind Facility, NH (2015)** | 78 | 0.46988293 | 0 | 0.01814059 | 0.108374384 | 6.64E-10 |
| **Anonymous Wind Facility, NH (2015)** | 79 | 0.46988293 | 0 | 0.01814059 | 0.108374384 | 6.64E-10 |
| **Anonymous Wind Facility, NH (2015)** | 80 | 0.46988293 | 0 | 0.01814059 | 0.108374384 | 6.64E-10 |
| **Anonymous Wind Facility, NH (2015)** | 81 | 0.46988293 | 0 | 0.01814059 | 0.108374384 | 6.64E-10 |
| **Anonymous Wind Facility, NH (2015)** | 82 | 0.46988293 | 0 | 0.01814059 | 0.108374384 | 6.64E-10 |
| **Anonymous Wind Facility, NH (2015)** | 83 | 0.46988293 | 0 | 0.01814059 | 0.108374384 | 6.64E-10 |
| **Anonymous Wind Facility, NH (2015)** | 84 | 0.46988293 | 0 | 0.01814059 | 0.108374384 | 6.64E-10 |
| **Anonymous Wind Facility, NH (2015)** | 85 | 0.46988293 | 0 | 0.01814059 | 0.108374384 | 6.64E-10 |
| **Anonymous Wind Facility, NH (2015)** | 86 | 0.46988293 | 0 | 0.01814059 | 0.108374384 | 6.64E-10 |
| **Anonymous Wind Facility, NH (2015)** | 87 | 0.46988293 | 0 | 0.01814059 | 0.108374384 | 6.64E-10 |
| **Anonymous Wind Facility, NH (2015)** | 88 | 0.46988293 | 0 | 0.01814059 | 0.108374384 | 6.64E-10 |
| **Anonymous Wind Facility, NH (2015)** | 89 | 0.46988293 | 0 | 0.01814059 | 0.108374384 | 6.64E-10 |
| **Anonymous Wind Facility, NH (2015)** | 90 | 0.46988293 | 0 | 0.01814059 | 0.108374384 | 6.64E-10 |
| **Anonymous Wind Facility, NH (2015)** | 91 | 0.46988293 | 0 | 0.01814059 | 0.108374384 | 6.64E-10 |
| **Anonymous Wind Facility, NH (2015)** | 92 | 0.46988293 | 0 | 0.01814059 | 0.108374384 | 6.64E-10 |
| **Anonymous Wind Facility, NH (2015)** | 93 | 0.46988293 | 0 | 0.01814059 | 0.108374384 | 6.64E-10 |
| **Anonymous Wind Facility, NH (2015)** | 94 | 0.46988293 | 0 | 0.01814059 | 0.108374384 | 6.64E-10 |
| **Anonymous Wind Facility, NH (2015)** | 95 | 0.46988293 | 0 | 0.01814059 | 0.108374384 | 6.64E-10 |
| **Anonymous Wind Facility, NH (2015)** | 96 | 0.46988293 | 0 | 0.01814059 | 0.108374384 | 6.64E-10 |
| **Anonymous Wind Facility, NH (2015)** | 97 | 0.46988293 | 0 | 0.01814059 | 0.108374384 | 6.64E-10 |
| **Anonymous Wind Facility, NH (2015)** | 98 | 0.46988293 | 0 | 0.01814059 | 0.108374384 | 6.64E-10 |
| **Anonymous Wind Facility, NH (2015)** | 99 | 0.46988293 | 0 | 0.01814059 | 0.108374384 | 6.64E-10 |
| **Anonymous Wind Facility, NH (2015)** | 100 | 0.46988293 | 0 | 0.01814059 | 0.108374384 | 6.64E-10 |
| **Anonymous Wind Facility, NH (2015)** | 101 | 0.46988293 | 0 | 0.01814059 | 0.108374384 | 6.64E-10 |
| **Anonymous Wind Facility, NH (2015)** | 102 | 0.46988293 | 0 | 0.01814059 | 0.108374384 | 6.64E-10 |
| **Anonymous Wind Facility, NH (2015)** | 103 | 0.46988293 | 0 | 0.01814059 | 0.108374384 | 6.64E-10 |
| **Anonymous Wind Facility, NH (2015)** | 104 | 0.46988293 | 0 | 0.01814059 | 0.108374384 | 6.64E-10 |
| **Anonymous Wind Facility, NH (2015)** | 105 | 0.46988293 | 0 | 0.01814059 | 0.108374384 | 6.64E-10 |
| **Anonymous Wind Facility, NH (2015)** | 106 | 0.46988293 | 0 | 0.01814059 | 0.108374384 | 6.64E-10 |
| **Anonymous Wind Facility, NH (2015)** | 107 | 0.46988293 | 0 | 0.01814059 | 0.108374384 | 6.64E-10 |
| **Anonymous Wind Facility, NH (2015)** | 108 | 0.46988293 | 0 | 0.01814059 | 0.108374384 | 6.64E-10 |
| **Anonymous Wind Facility, NH (2015)** | 109 | 0.46988293 | 0 | 0.01814059 | 0.108374384 | 6.64E-10 |
| **Anonymous Wind Facility, NH (2015)** | 110 | 0.46988293 | 0 | 0.01814059 | 0.108374384 | 6.64E-10 |
| **Anonymous Wind Facility, NH (2015)** | 111 | 0.46988293 | 0 | 0.01814059 | 0.108374384 | 6.64E-10 |
| **Anonymous Wind Facility, NH (2015)** | 112 | 0.46988293 | 0 | 0.01814059 | 0.108374384 | 6.64E-10 |
| **Anonymous Wind Facility, NH (2015)** | 113 | 0.46988293 | 0 | 0.01814059 | 0.108374384 | 6.64E-10 |
| **Anonymous Wind Facility, NH (2015)** | 114 | 0.46988293 | 0 | 0.01814059 | 0.108374384 | 6.64E-10 |
| **Anonymous Wind Facility, NH (2015)** | 115 | 0.46988293 | 0 | 0.01814059 | 0.108374384 | 6.64E-10 |
| **Anonymous Wind Facility, NH (2015)** | 116 | 0.46988293 | 0 | 0.01814059 | 0.108374384 | 6.64E-10 |
| **Anonymous Wind Facility, NH (2015)** | 117 | 0.46988293 | 0 | 0.01814059 | 0.108374384 | 6.64E-10 |
| **Anonymous Wind Facility, NH (2015)** | 118 | 0.46988293 | 0 | 0.01814059 | 0.108374384 | 6.64E-10 |
| **Anonymous Wind Facility, NH (2015)** | 119 | 0.46988293 | 0 | 0.01814059 | 0.108374384 | 6.64E-10 |
| **Anonymous Wind Facility, NH (2015)** | 120 | 0.46988293 | 0 | 0.01814059 | 0.108374384 | 6.64E-10 |
| **Anonymous Wind Facility, NH (2015)** | 121 | 0.46988293 | 0 | 0.01814059 | 0.108374384 | 6.64E-10 |
| **Anonymous Wind Facility, NH (2015)** | 122 | 0.46988293 | 0 | 0.01814059 | 0.108374384 | 6.64E-10 |
| **Anonymous Wind Facility, NH (2015)** | 123 | 0.46988293 | 0 | 0.01814059 | 0.108374384 | 6.64E-10 |
| **Anonymous Wind Facility, NH (2015)** | 124 | 0.46988293 | 0 | 0.01814059 | 0.108374384 | 6.64E-10 |
| **Anonymous Wind Facility, NH (2015)** | 125 | 0.46988293 | 0 | 0.01814059 | 0.108374384 | 6.64E-10 |
| **Anonymous Wind Facility, NH (2015)** | 126 | 0.46988293 | 0 | 0.01814059 | 0.108374384 | 6.64E-10 |
| **Anonymous Wind Facility, NH (2015)** | 127 | 0.46988293 | 0 | 0.01814059 | 0.108374384 | 6.64E-10 |
| **Anonymous Wind Facility, NH (2015)** | 128 | 0.46988293 | 0 | 0.01814059 | 0.108374384 | 6.64E-10 |
| **Anonymous Wind Facility, NH (2015)** | 129 | 0.46988293 | 0 | 0.01814059 | 0.108374384 | 6.64E-10 |
| **Anonymous Wind Facility, NH (2015)** | 130 | 0.46988293 | 0 | 0.01814059 | 0.108374384 | 6.64E-10 |
| **Anonymous Wind Facility, NH (2015)** | 131 | 0.46988293 | 0 | 0.01814059 | 0.108374384 | 6.64E-10 |
| **Anonymous Wind Facility, NH (2015)** | 132 | 0.46988293 | 0 | 0.01814059 | 0.108374384 | 6.64E-10 |
| **Anonymous Wind Facility, NH (2015)** | 133 | 0.46988293 | 0 | 0.01814059 | 0.108374384 | 6.64E-10 |
| **Anonymous Wind Facility, NH (2015)** | 134 | 0.46988293 | 0 | 0.01814059 | 0.108374384 | 6.64E-10 |
| **Anonymous Wind Facility, NH (2015)** | 135 | 0.46988293 | 0 | 0.01814059 | 0.108374384 | 6.64E-10 |
| **Anonymous Wind Facility, NH (2015)** | 136 | 0.46988293 | 0 | 0.01814059 | 0.108374384 | 6.64E-10 |
| **Anonymous Wind Facility, NH (2015)** | 137 | 0.46988293 | 0 | 0.01814059 | 0.108374384 | 6.64E-10 |
| **Anonymous Wind Facility, NH (2015)** | 138 | 0.46988293 | 0 | 0.01814059 | 0.108374384 | 6.64E-10 |
| **Anonymous Wind Facility, NH (2015)** | 139 | 0.46988293 | 0 | 0.01814059 | 0.108374384 | 6.64E-10 |
| **Anonymous Wind Facility, NH (2015)** | 140 | 0.46988293 | 0 | 0.01814059 | 0.108374384 | 6.64E-10 |
| **Anonymous Wind Facility, NH (2015)** | 141 | 0.46988293 | 0 | 0.01814059 | 0.108374384 | 6.64E-10 |
| **Anonymous Wind Facility, NH (2015)** | 142 | 0.46988293 | 0 | 0.01814059 | 0.108374384 | 6.64E-10 |
| **Anonymous Wind Facility, NH (2015)** | 143 | 0.46988293 | 0 | 0.01814059 | 0.108374384 | 6.64E-10 |
| **Anonymous Wind Facility, NH (2015)** | 144 | 0.46988293 | 0 | 0.01814059 | 0.108374384 | 6.64E-10 |
| **Juniper Canyon Wind Project (2011-2012) [8]** | 3 | 0.44752949 | 0.999835515 | 0.047619048 | 0.078817734 | 0.001679403 |
| **Juniper Canyon Wind Project (2011-2012) [8]** | 4 | 0.44752949 | 0.999835515 | 0.047619048 | 0.078817734 | 0.001679403 |
| **Juniper Canyon Wind Project (2011-2012) [8]** | 5 | 0.44752949 | 0.999835515 | 0.047619048 | 0.078817734 | 0.001679403 |
| **Juniper Canyon Wind Project (2011-2012) [8]** | 6 | 0.44752949 | 0.999835515 | 0.047619048 | 0.078817734 | 0.001679403 |
| **Juniper Canyon Wind Project (2011-2012) [8]** | 7 | 0.44752949 | 0.999835515 | 0.047619048 | 0.078817734 | 0.001679403 |
| **Juniper Canyon Wind Project (2011-2012) [8]** | 8 | 0.44752949 | 0.999835515 | 0.047619048 | 0.078817734 | 0.001679403 |
| **Juniper Canyon Wind Project (2011-2012) [8]** | 9 | 0.44752949 | 0.999835515 | 0.047619048 | 0.078817734 | 0.001679403 |
| **Juniper Canyon Wind Project (2011-2012) [8]** | 10 | 0.44752949 | 0.999835515 | 0.047619048 | 0.078817734 | 0.001679403 |
| **Juniper Canyon Wind Project (2011-2012) [8]** | 11 | 0.44752949 | 0.999835515 | 0.047619048 | 0.078817734 | 0.001679403 |
| **Juniper Canyon Wind Project (2011-2012) [8]** | 12 | 0.44752949 | 0.999835515 | 0.047619048 | 0.078817734 | 0.001679403 |
| **Juniper Canyon Wind Project (2011-2012) [8]** | 13 | 0.44752949 | 0.999835515 | 0.047619048 | 0.078817734 | 0.001679403 |
| **Juniper Canyon Wind Project (2011-2012) [8]** | 14 | 0.44752949 | 0.999835515 | 0.047619048 | 0.078817734 | 0.001679403 |
| **Juniper Canyon Wind Project (2011-2012) [8]** | 15 | 0.44752949 | 0.999835515 | 0.047619048 | 0.078817734 | 0.001679403 |
| **Juniper Canyon Wind Project (2011-2012) [8]** | 16 | 0.44752949 | 0.999835515 | 0.047619048 | 0.078817734 | 0.001679403 |
| **Juniper Canyon Wind Project (2011-2012) [8]** | 17 | 0.44752949 | 0.999835515 | 0.047619048 | 0.078817734 | 0.001679403 |
| **Juniper Canyon Wind Project (2011-2012) [8]** | 18 | 0.44752949 | 0.999835515 | 0.047619048 | 0.078817734 | 0.001679403 |
| **Juniper Canyon Wind Project (2011-2012) [8]** | 19 | 0.44752949 | 0.999835515 | 0.047619048 | 0.078817734 | 0.001679403 |
| **Juniper Canyon Wind Project (2011-2012) [8]** | 20 | 0.44752949 | 0.999835515 | 0.047619048 | 0.078817734 | 0.001679403 |
| **Juniper Canyon Wind Project (2011-2012) [8]** | 21 | 0.44752949 | 0.999835515 | 0.047619048 | 0.078817734 | 0.001679403 |
| **Juniper Canyon Wind Project (2011-2012) [8]** | 22 | 0.44752949 | 0.999835515 | 0.047619048 | 0.078817734 | 0.001679403 |
| **Juniper Canyon Wind Project (2011-2012) [8]** | 23 | 0.44752949 | 0.999835515 | 0.047619048 | 0.078817734 | 0.001679403 |
| **Juniper Canyon Wind Project (2011-2012) [8]** | 24 | 0.44752949 | 0.999835515 | 0.047619048 | 0.078817734 | 0.001679403 |
| **Juniper Canyon Wind Project (2011-2012) [8]** | 25 | 0.44752949 | 0.999835515 | 0.047619048 | 0.078817734 | 0.001679403 |
| **Juniper Canyon Wind Project (2011-2012) [8]** | 26 | 0.44752949 | 0.999835515 | 0.047619048 | 0.078817734 | 0.001679403 |
| **Juniper Canyon Wind Project (2011-2012) [8]** | 27 | 0.44752949 | 0.999835515 | 0.047619048 | 0.078817734 | 0.001679403 |
| **Juniper Canyon Wind Project (2011-2012) [8]** | 28 | 0.44752949 | 0.999835515 | 0.047619048 | 0.078817734 | 0.001679403 |
| **Juniper Canyon Wind Project (2011-2012) [8]** | 29 | 0.44752949 | 0.999835515 | 0.047619048 | 0.078817734 | 0.001679403 |
| **Juniper Canyon Wind Project (2011-2012) [8]** | 30 | 0.44752949 | 0.999835515 | 0.047619048 | 0.078817734 | 0.001679403 |
| **Juniper Canyon Wind Project (2011-2012) [8]** | 31 | 0.44752949 | 0.999835515 | 0.047619048 | 0.078817734 | 0.001679403 |
| **Juniper Canyon Wind Project (2011-2012) [8]** | 32 | 0.44752949 | 0.999835515 | 0.047619048 | 0.078817734 | 0.001679403 |
| **Juniper Canyon Wind Project (2011-2012) [8]** | 33 | 0.44752949 | 0.999835515 | 0.047619048 | 0.078817734 | 0.001679403 |
| **Juniper Canyon Wind Project (2011-2012) [8]** | 34 | 0.44752949 | 0.999835515 | 0.047619048 | 0.078817734 | 0.001679403 |
| **Juniper Canyon Wind Project (2011-2012) [8]** | 35 | 0.44752949 | 0.999835515 | 0.047619048 | 0.078817734 | 0.001679403 |
| **Juniper Canyon Wind Project (2011-2012) [8]** | 36 | 0.44752949 | 0.999835515 | 0.047619048 | 0.078817734 | 0.001679403 |
| **Juniper Canyon Wind Project (2011-2012) [8]** | 37 | 0.44752949 | 0.999835515 | 0.047619048 | 0.078817734 | 0.001679403 |
| **Juniper Canyon Wind Project (2011-2012) [8]** | 38 | 0.44752949 | 0.999835515 | 0.047619048 | 0.078817734 | 0.001679403 |
| **Juniper Canyon Wind Project (2011-2012) [8]** | 39 | 0.44752949 | 0.999835515 | 0.047619048 | 0.078817734 | 0.001679403 |
| **Juniper Canyon Wind Project (2011-2012) [8]** | 40 | 0.44752949 | 0.999835515 | 0.047619048 | 0.078817734 | 0.001679403 |
| **Juniper Canyon Wind Project (2011-2012) [8]** | 41 | 0.44752949 | 0.999835515 | 0.047619048 | 0.078817734 | 0.001679403 |
| **Juniper Canyon Wind Project (2011-2012) [8]** | 42 | 0.44752949 | 0.999835515 | 0.047619048 | 0.078817734 | 0.001679403 |
| **Juniper Canyon Wind Project (2011-2012) [8]** | 43 | 0.44752949 | 0.999835515 | 0.047619048 | 0.078817734 | 0.001679403 |
| **Juniper Canyon Wind Project (2011-2012) [8]** | 44 | 0.44752949 | 0.999835515 | 0.047619048 | 0.078817734 | 0.001679403 |
| **Juniper Canyon Wind Project (2011-2012) [8]** | 45 | 0.44752949 | 0.999835515 | 0.047619048 | 0.078817734 | 0.001679403 |
| **Juniper Canyon Wind Project (2011-2012) [8]** | 46 | 0.44752949 | 0.999835515 | 0.047619048 | 0.078817734 | 0.001679403 |
| **Juniper Canyon Wind Project (2011-2012) [8]** | 47 | 0.44752949 | 0.999835515 | 0.047619048 | 0.078817734 | 0.001679403 |
| **Juniper Canyon Wind Project (2011-2012) [8]** | 48 | 0.44752949 | 0.999835515 | 0.047619048 | 0.078817734 | 0.001679403 |
| **Juniper Canyon Wind Project (2011-2012) [8]** | 49 | 0.44752949 | 0.999835515 | 0.047619048 | 0.078817734 | 0.001679403 |
| **Juniper Canyon Wind Project (2011-2012) [8]** | 50 | 0.44752949 | 0.999835515 | 0.047619048 | 0.078817734 | 0.001679403 |
| **Juniper Canyon Wind Project (2011-2012) [8]** | 51 | 0.44752949 | 0.999835515 | 0.047619048 | 0.078817734 | 0.001679403 |
| **Juniper Canyon Wind Project (2011-2012) [8]** | 52 | 0.44752949 | 0.999835515 | 0.047619048 | 0.078817734 | 0.001679403 |
| **Juniper Canyon Wind Project (2011-2012) [8]** | 53 | 0.44752949 | 0.999835515 | 0.047619048 | 0.078817734 | 0.001679403 |
| **Juniper Canyon Wind Project (2011-2012) [8]** | 54 | 0.44752949 | 0.999835515 | 0.047619048 | 0.078817734 | 0.001679403 |
| **Juniper Canyon Wind Project (2011-2012) [8]** | 55 | 0.44752949 | 0.999835515 | 0.047619048 | 0.078817734 | 0.001679403 |
| **Juniper Canyon Wind Project (2011-2012) [8]** | 56 | 0.44752949 | 0.999835515 | 0.047619048 | 0.078817734 | 0.001679403 |
| **Juniper Canyon Wind Project (2011-2012) [8]** | 57 | 0.44752949 | 0.999835515 | 0.047619048 | 0.078817734 | 0.001679403 |
| **Juniper Canyon Wind Project (2011-2012) [8]** | 58 | 0.44752949 | 0.999835515 | 0.047619048 | 0.078817734 | 0.001679403 |
| **Juniper Canyon Wind Project (2011-2012) [8]** | 59 | 0.44752949 | 0.999835515 | 0.047619048 | 0.078817734 | 0.001679403 |
| **Juniper Canyon Wind Project (2011-2012) [8]** | 60 | 0.44752949 | 0.999835515 | 0.047619048 | 0.078817734 | 0.001679403 |
| **Juniper Canyon Wind Project (2011-2012) [8]** | 61 | 0.44752949 | 0.999835515 | 0.047619048 | 0.078817734 | 0.001679403 |
| **Juniper Canyon Wind Project (2011-2012) [8]** | 62 | 0.44752949 | 0.999835515 | 0.047619048 | 0.078817734 | 0.001679403 |
| **Juniper Canyon Wind Project (2011-2012) [8]** | 63 | 0.44752949 | 0.999835515 | 0.047619048 | 0.078817734 | 0.001679403 |
| **Juniper Canyon Wind Project (2011-2012) [8]** | 64 | 0.44752949 | 0.999835515 | 0.047619048 | 0.078817734 | 0.001679403 |
| **Juniper Canyon Wind Project (2011-2012) [8]** | 65 | 0.44752949 | 0.999835515 | 0.047619048 | 0.078817734 | 0.001679403 |
| **Juniper Canyon Wind Project (2011-2012) [8]** | 66 | 0.44752949 | 0.999835515 | 0.047619048 | 0.078817734 | 0.001679403 |
| **Juniper Canyon Wind Project (2011-2012) [8]** | 67 | 0.44752949 | 0.999835515 | 0.047619048 | 0.078817734 | 0.001679403 |
| **Juniper Canyon Wind Project (2011-2012) [8]** | 68 | 0.44752949 | 0.999835515 | 0.047619048 | 0.078817734 | 0.001679403 |
| **Juniper Canyon Wind Project (2011-2012) [8]** | 69 | 0.44752949 | 0.999835515 | 0.047619048 | 0.078817734 | 0.001679403 |
| **Juniper Canyon Wind Project (2011-2012) [8]** | 70 | 0.44752949 | 0.999835515 | 0.047619048 | 0.078817734 | 0.001679403 |
| **Juniper Canyon Wind Project (2011-2012) [8]** | 71 | 0.44752949 | 0.999835515 | 0.047619048 | 0.078817734 | 0.001679403 |
| **Juniper Canyon Wind Project (2011-2012) [8]** | 72 | 0.44752949 | 0.999835515 | 0.047619048 | 0.078817734 | 0.001679403 |
| **Juniper Canyon Wind Project (2011-2012) [8]** | 73 | 0.44752949 | 0.999835515 | 0.047619048 | 0.078817734 | 0.001679403 |
| **Juniper Canyon Wind Project (2011-2012) [8]** | 74 | 0.44752949 | 0.999835515 | 0.047619048 | 0.078817734 | 0.001679403 |
| **Juniper Canyon Wind Project (2011-2012) [8]** | 75 | 0.44752949 | 0.999835515 | 0.047619048 | 0.078817734 | 0.001679403 |
| **Juniper Canyon Wind Project (2011-2012) [8]** | 76 | 0.44752949 | 0.999835515 | 0.047619048 | 0.078817734 | 0.001679403 |
| **Juniper Canyon Wind Project (2011-2012) [8]** | 77 | 0.44752949 | 0.999835515 | 0.047619048 | 0.078817734 | 0.001679403 |
| **Juniper Canyon Wind Project (2011-2012) [8]** | 78 | 0.44752949 | 0.999835515 | 0.047619048 | 0.078817734 | 0.001679403 |
| **Juniper Canyon Wind Project (2011-2012) [8]** | 79 | 0.44752949 | 0.999835515 | 0.047619048 | 0.078817734 | 0.001679403 |
| **Juniper Canyon Wind Project (2011-2012) [8]** | 80 | 0.44752949 | 0.999835515 | 0.047619048 | 0.078817734 | 0.001679403 |
| **Juniper Canyon Wind Project (2011-2012) [8]** | 81 | 0.44752949 | 0.999835515 | 0.047619048 | 0.078817734 | 0.001679403 |
| **Juniper Canyon Wind Project (2011-2012) [8]** | 82 | 0.44752949 | 0.999835515 | 0.047619048 | 0.078817734 | 0.001679403 |
| **Juniper Canyon Wind Project (2011-2012) [8]** | 83 | 0.44752949 | 0.999835515 | 0.047619048 | 0.078817734 | 0.001679403 |
| **Juniper Canyon Wind Project (2011-2012) [8]** | 84 | 0.44752949 | 0.999835515 | 0.047619048 | 0.078817734 | 0.001679403 |
| **Juniper Canyon Wind Project (2011-2012) [8]** | 85 | 0.44752949 | 0.999835515 | 0.047619048 | 0.078817734 | 0.001679403 |
| **Juniper Canyon Wind Project (2011-2012) [8]** | 86 | 0.44752949 | 0.999835515 | 0.047619048 | 0.078817734 | 0.001679403 |
| **Juniper Canyon Wind Project (2011-2012) [8]** | 87 | 0.44752949 | 0.999835515 | 0.047619048 | 0.078817734 | 0.001679403 |
| **Juniper Canyon Wind Project (2011-2012) [8]** | 88 | 0.44752949 | 0.999835515 | 0.047619048 | 0.078817734 | 0.001679403 |
| **Juniper Canyon Wind Project (2011-2012) [8]** | 89 | 0.44752949 | 0.999835515 | 0.047619048 | 0.078817734 | 0.001679403 |
| **Juniper Canyon Wind Project (2011-2012) [8]** | 90 | 0.44752949 | 0.999835515 | 0.047619048 | 0.078817734 | 0.001679403 |
| **Juniper Canyon Wind Project (2011-2012) [8]** | 91 | 0.44752949 | 0.999835515 | 0.047619048 | 0.078817734 | 0.001679403 |
| **Juniper Canyon Wind Project (2011-2012) [8]** | 92 | 0.44752949 | 0.999835515 | 0.047619048 | 0.078817734 | 0.001679403 |
| **Juniper Canyon Wind Project (2011-2012) [8]** | 93 | 0.44752949 | 0.874955359 | 0.047619048 | 0.078817734 | 0.001469644 |
| **Juniper Canyon Wind Project (2011-2012) [8]** | 94 | 0.44752949 | 0.770634852 | 0.047619048 | 0.078817734 | 0.001294419 |
| **Juniper Canyon Wind Project (2011-2012) [8]** | 95 | 0.44752949 | 0.70406163 | 0.047619048 | 0.078817734 | 0.001182598 |
| **Juniper Canyon Wind Project (2011-2012) [8]** | 96 | 0.44752949 | 0.651059214 | 0.047619048 | 0.078817734 | 0.001093571 |
| **Juniper Canyon Wind Project (2011-2012) [8]** | 97 | 0.44752949 | 0.605908345 | 0.047619048 | 0.078817734 | 0.001017732 |
| **Juniper Canyon Wind Project (2011-2012) [8]** | 98 | 0.44752949 | 0.566104433 | 0.047619048 | 0.078817734 | 0.000950874 |
| **Juniper Canyon Wind Project (2011-2012) [8]** | 99 | 0.44752949 | 0.530291046 | 0.047619048 | 0.078817734 | 0.000890719 |
| **Juniper Canyon Wind Project (2011-2012) [8]** | 100 | 0.44752949 | 0.497626866 | 0.047619048 | 0.078817734 | 0.000835853 |
| **Juniper Canyon Wind Project (2011-2012) [8]** | 101 | 0.44752949 | 0.467399366 | 0.047619048 | 0.078817734 | 0.000785081 |
| **Juniper Canyon Wind Project (2011-2012) [8]** | 102 | 0.44752949 | 0.439244452 | 0.047619048 | 0.078817734 | 0.00073779 |
| **Juniper Canyon Wind Project (2011-2012) [8]** | 103 | 0.44752949 | 0.413002074 | 0.047619048 | 0.078817734 | 0.000693711 |
| **Juniper Canyon Wind Project (2011-2012) [8]** | 104 | 0.44752949 | 0.388216302 | 0.047619048 | 0.078817734 | 0.000652079 |
| **Juniper Canyon Wind Project (2011-2012) [8]** | 105 | 0.44752949 | 0.36473717 | 0.047619048 | 0.078817734 | 0.000612641 |
| **Juniper Canyon Wind Project (2011-2012) [8]** | 106 | 0.44752949 | 0.342618578 | 0.047619048 | 0.078817734 | 0.000575489 |
| **Juniper Canyon Wind Project (2011-2012) [8]** | 107 | 0.44752949 | 0.321342803 | 0.047619048 | 0.078817734 | 0.000539753 |
| **Juniper Canyon Wind Project (2011-2012) [8]** | 108 | 0.44752949 | 0.301280006 | 0.047619048 | 0.078817734 | 0.000506054 |
| **Juniper Canyon Wind Project (2011-2012) [8]** | 109 | 0.44752949 | 0.281840104 | 0.047619048 | 0.078817734 | 0.000473401 |
| **Juniper Canyon Wind Project (2011-2012) [8]** | 110 | 0.44752949 | 0.263447172 | 0.047619048 | 0.078817734 | 0.000442507 |
| **Juniper Canyon Wind Project (2011-2012) [8]** | 111 | 0.44752949 | 0.245568146 | 0.047619048 | 0.078817734 | 0.000412476 |
| **Juniper Canyon Wind Project (2011-2012) [8]** | 112 | 0.44752949 | 0.228529973 | 0.047619048 | 0.078817734 | 0.000383857 |
| **Juniper Canyon Wind Project (2011-2012) [8]** | 113 | 0.44752949 | 0.212061594 | 0.047619048 | 0.078817734 | 0.000356195 |
| **Juniper Canyon Wind Project (2011-2012) [8]** | 114 | 0.44752949 | 0.196081844 | 0.047619048 | 0.078817734 | 0.000329355 |
| **Juniper Canyon Wind Project (2011-2012) [8]** | 115 | 0.44752949 | 0.180848198 | 0.047619048 | 0.078817734 | 0.000303767 |
| **Juniper Canyon Wind Project (2011-2012) [8]** | 116 | 0.44752949 | 0.165906049 | 0.047619048 | 0.078817734 | 0.000278669 |
| **Juniper Canyon Wind Project (2011-2012) [8]** | 117 | 0.44752949 | 0.151566838 | 0.047619048 | 0.078817734 | 0.000254584 |
| **Juniper Canyon Wind Project (2011-2012) [8]** | 118 | 0.44752949 | 0.137710037 | 0.047619048 | 0.078817734 | 0.000231309 |
| **Juniper Canyon Wind Project (2011-2012) [8]** | 119 | 0.44752949 | 0.124100639 | 0.047619048 | 0.078817734 | 0.000208449 |
| **Juniper Canyon Wind Project (2011-2012) [8]** | 120 | 0.44752949 | 0.111049749 | 0.047619048 | 0.078817734 | 0.000186528 |
| **Juniper Canyon Wind Project (2011-2012) [8]** | 121 | 0.44752949 | 0.098379113 | 0.047619048 | 0.078817734 | 0.000165245 |
| **Juniper Canyon Wind Project (2011-2012) [8]** | 122 | 0.44752949 | 0.085920606 | 0.047619048 | 0.078817734 | 0.000144319 |
| **Juniper Canyon Wind Project (2011-2012) [8]** | 123 | 0.44752949 | 0.073889962 | 0.047619048 | 0.078817734 | 0.000124111 |
| **Juniper Canyon Wind Project (2011-2012) [8]** | 124 | 0.44752949 | 0.06226176 | 0.047619048 | 0.078817734 | 0.00010458 |
| **Juniper Canyon Wind Project (2011-2012) [8]** | 125 | 0.44752949 | 0.050823406 | 0.047619048 | 0.078817734 | 8.54E-05 |
| **Juniper Canyon Wind Project (2011-2012) [8]** | 126 | 0.44752949 | 0.039623441 | 0.047619048 | 0.078817734 | 6.66E-05 |
| **Juniper Canyon Wind Project (2011-2012) [8]** | 127 | 0.44752949 | 0.028876223 | 0.047619048 | 0.078817734 | 4.85E-05 |
| **Juniper Canyon Wind Project (2011-2012) [8]** | 128 | 0.44752949 | 0.018338879 | 0.047619048 | 0.078817734 | 3.08E-05 |
| **Juniper Canyon Wind Project (2011-2012) [8]** | 129 | 0.44752949 | 0.007968134 | 0.047619048 | 0.078817734 | 1.34E-05 |
| **Juniper Canyon Wind Project (2011-2012) [8]** | 130 | 0.44752949 | 0.000395495 | 0.047619048 | 0.078817734 | 6.64E-07 |
| **Juniper Canyon Wind Project (2011-2012) [8]** | 131 | 0.44752949 | 0 | 0.047619048 | 0.078817734 | 6.64E-10 |
| **Juniper Canyon Wind Project (2011-2012) [8]** | 132 | 0.44752949 | 0 | 0.047619048 | 0.078817734 | 6.64E-10 |
| **Juniper Canyon Wind Project (2011-2012) [8]** | 133 | 0.44752949 | 0 | 0.047619048 | 0.078817734 | 6.64E-10 |
| **Juniper Canyon Wind Project (2011-2012) [8]** | 134 | 0.44752949 | 0 | 0.047619048 | 0.078817734 | 6.64E-10 |
| **Juniper Canyon Wind Project (2011-2012) [8]** | 135 | 0.44752949 | 0 | 0.047619048 | 0.078817734 | 6.64E-10 |
| **Juniper Canyon Wind Project (2011-2012) [8]** | 136 | 0.44752949 | 0 | 0.047619048 | 0.078817734 | 6.64E-10 |
| **Juniper Canyon Wind Project (2011-2012) [8]** | 137 | 0.44752949 | 0 | 0.047619048 | 0.078817734 | 6.64E-10 |
| **Juniper Canyon Wind Project (2011-2012) [8]** | 138 | 0.44752949 | 0 | 0.047619048 | 0.078817734 | 6.64E-10 |
| **Juniper Canyon Wind Project (2011-2012) [8]** | 139 | 0.44752949 | 0 | 0.047619048 | 0.078817734 | 6.64E-10 |
| **Juniper Canyon Wind Project (2011-2012) [8]** | 140 | 0.44752949 | 0 | 0.047619048 | 0.078817734 | 6.64E-10 |
| **Juniper Canyon Wind Project (2011-2012) [8]** | 141 | 0.44752949 | 0 | 0.047619048 | 0.078817734 | 6.64E-10 |
| **Juniper Canyon Wind Project (2011-2012) [8]** | 142 | 0.44752949 | 0 | 0.047619048 | 0.078817734 | 6.64E-10 |
| **Juniper Canyon Wind Project (2011-2012) [8]** | 143 | 0.44752949 | 0 | 0.047619048 | 0.078817734 | 6.64E-10 |
| **Juniper Canyon Wind Project (2011-2012) [8]** | 144 | 0.44752949 | 0 | 0.047619048 | 0.078817734 | 6.64E-10 |
| **Leaning Juniper [9]** | 3 | 0.47332869 | 1 | 0.050642479 | 0.078817734 | 0.001889304 |
| **Leaning Juniper [9]** | 4 | 0.47332869 | 1 | 0.050642479 | 0.078817734 | 0.001889304 |
| **Leaning Juniper [9]** | 5 | 0.47332869 | 1 | 0.050642479 | 0.078817734 | 0.001889304 |
| **Leaning Juniper [9]** | 6 | 0.47332869 | 1 | 0.050642479 | 0.078817734 | 0.001889304 |
| **Leaning Juniper [9]** | 7 | 0.47332869 | 1 | 0.050642479 | 0.078817734 | 0.001889304 |
| **Leaning Juniper [9]** | 8 | 0.47332869 | 1 | 0.050642479 | 0.078817734 | 0.001889304 |
| **Leaning Juniper [9]** | 9 | 0.47332869 | 1 | 0.050642479 | 0.078817734 | 0.001889304 |
| **Leaning Juniper [9]** | 10 | 0.47332869 | 1 | 0.050642479 | 0.078817734 | 0.001889304 |
| **Leaning Juniper [9]** | 11 | 0.47332869 | 1 | 0.050642479 | 0.078817734 | 0.001889304 |
| **Leaning Juniper [9]** | 12 | 0.47332869 | 1 | 0.050642479 | 0.078817734 | 0.001889304 |
| **Leaning Juniper [9]** | 13 | 0.47332869 | 1 | 0.050642479 | 0.078817734 | 0.001889304 |
| **Leaning Juniper [9]** | 14 | 0.47332869 | 1 | 0.050642479 | 0.078817734 | 0.001889304 |
| **Leaning Juniper [9]** | 15 | 0.47332869 | 1 | 0.050642479 | 0.078817734 | 0.001889304 |
| **Leaning Juniper [9]** | 16 | 0.47332869 | 1 | 0.050642479 | 0.078817734 | 0.001889304 |
| **Leaning Juniper [9]** | 17 | 0.47332869 | 1 | 0.050642479 | 0.078817734 | 0.001889304 |
| **Leaning Juniper [9]** | 18 | 0.47332869 | 1 | 0.050642479 | 0.078817734 | 0.001889304 |
| **Leaning Juniper [9]** | 19 | 0.47332869 | 1 | 0.050642479 | 0.078817734 | 0.001889304 |
| **Leaning Juniper [9]** | 20 | 0.47332869 | 1 | 0.050642479 | 0.078817734 | 0.001889304 |
| **Leaning Juniper [9]** | 21 | 0.47332869 | 1 | 0.050642479 | 0.078817734 | 0.001889304 |
| **Leaning Juniper [9]** | 22 | 0.47332869 | 1 | 0.050642479 | 0.078817734 | 0.001889304 |
| **Leaning Juniper [9]** | 23 | 0.47332869 | 1 | 0.050642479 | 0.078817734 | 0.001889304 |
| **Leaning Juniper [9]** | 24 | 0.47332869 | 1 | 0.050642479 | 0.078817734 | 0.001889304 |
| **Leaning Juniper [9]** | 25 | 0.47332869 | 1 | 0.050642479 | 0.078817734 | 0.001889304 |
| **Leaning Juniper [9]** | 26 | 0.47332869 | 1 | 0.050642479 | 0.078817734 | 0.001889304 |
| **Leaning Juniper [9]** | 27 | 0.47332869 | 1 | 0.050642479 | 0.078817734 | 0.001889304 |
| **Leaning Juniper [9]** | 28 | 0.47332869 | 1 | 0.050642479 | 0.078817734 | 0.001889304 |
| **Leaning Juniper [9]** | 29 | 0.47332869 | 1 | 0.050642479 | 0.078817734 | 0.001889304 |
| **Leaning Juniper [9]** | 30 | 0.47332869 | 1 | 0.050642479 | 0.078817734 | 0.001889304 |
| **Leaning Juniper [9]** | 31 | 0.47332869 | 1 | 0.050642479 | 0.078817734 | 0.001889304 |
| **Leaning Juniper [9]** | 32 | 0.47332869 | 1 | 0.050642479 | 0.078817734 | 0.001889304 |
| **Leaning Juniper [9]** | 33 | 0.47332869 | 1 | 0.050642479 | 0.078817734 | 0.001889304 |
| **Leaning Juniper [9]** | 34 | 0.47332869 | 1 | 0.050642479 | 0.078817734 | 0.001889304 |
| **Leaning Juniper [9]** | 35 | 0.47332869 | 1 | 0.050642479 | 0.078817734 | 0.001889304 |
| **Leaning Juniper [9]** | 36 | 0.47332869 | 1 | 0.050642479 | 0.078817734 | 0.001889304 |
| **Leaning Juniper [9]** | 37 | 0.47332869 | 1 | 0.050642479 | 0.078817734 | 0.001889304 |
| **Leaning Juniper [9]** | 38 | 0.47332869 | 1 | 0.050642479 | 0.078817734 | 0.001889304 |
| **Leaning Juniper [9]** | 39 | 0.47332869 | 1 | 0.050642479 | 0.078817734 | 0.001889304 |
| **Leaning Juniper [9]** | 40 | 0.47332869 | 1 | 0.050642479 | 0.078817734 | 0.001889304 |
| **Leaning Juniper [9]** | 41 | 0.47332869 | 1 | 0.050642479 | 0.078817734 | 0.001889304 |
| **Leaning Juniper [9]** | 42 | 0.47332869 | 1 | 0.050642479 | 0.078817734 | 0.001889304 |
| **Leaning Juniper [9]** | 43 | 0.47332869 | 1 | 0.050642479 | 0.078817734 | 0.001889304 |
| **Leaning Juniper [9]** | 44 | 0.47332869 | 1 | 0.050642479 | 0.078817734 | 0.001889304 |
| **Leaning Juniper [9]** | 45 | 0.47332869 | 1 | 0.050642479 | 0.078817734 | 0.001889304 |
| **Leaning Juniper [9]** | 46 | 0.47332869 | 1 | 0.050642479 | 0.078817734 | 0.001889304 |
| **Leaning Juniper [9]** | 47 | 0.47332869 | 1 | 0.050642479 | 0.078817734 | 0.001889304 |
| **Leaning Juniper [9]** | 48 | 0.47332869 | 1 | 0.050642479 | 0.078817734 | 0.001889304 |
| **Leaning Juniper [9]** | 49 | 0.47332869 | 1 | 0.050642479 | 0.078817734 | 0.001889304 |
| **Leaning Juniper [9]** | 50 | 0.47332869 | 1 | 0.050642479 | 0.078817734 | 0.001889304 |
| **Leaning Juniper [9]** | 51 | 0.47332869 | 1 | 0.050642479 | 0.078817734 | 0.001889304 |
| **Leaning Juniper [9]** | 52 | 0.47332869 | 1 | 0.050642479 | 0.078817734 | 0.001889304 |
| **Leaning Juniper [9]** | 53 | 0.47332869 | 1 | 0.050642479 | 0.078817734 | 0.001889304 |
| **Leaning Juniper [9]** | 54 | 0.47332869 | 1 | 0.050642479 | 0.078817734 | 0.001889304 |
| **Leaning Juniper [9]** | 55 | 0.47332869 | 1 | 0.050642479 | 0.078817734 | 0.001889304 |
| **Leaning Juniper [9]** | 56 | 0.47332869 | 1 | 0.050642479 | 0.078817734 | 0.001889304 |
| **Leaning Juniper [9]** | 57 | 0.47332869 | 1 | 0.050642479 | 0.078817734 | 0.001889304 |
| **Leaning Juniper [9]** | 58 | 0.47332869 | 1 | 0.050642479 | 0.078817734 | 0.001889304 |
| **Leaning Juniper [9]** | 59 | 0.47332869 | 1 | 0.050642479 | 0.078817734 | 0.001889304 |
| **Leaning Juniper [9]** | 60 | 0.47332869 | 1 | 0.050642479 | 0.078817734 | 0.001889304 |
| **Leaning Juniper [9]** | 61 | 0.47332869 | 1 | 0.050642479 | 0.078817734 | 0.001889304 |
| **Leaning Juniper [9]** | 62 | 0.47332869 | 1 | 0.050642479 | 0.078817734 | 0.001889304 |
| **Leaning Juniper [9]** | 63 | 0.47332869 | 1 | 0.050642479 | 0.078817734 | 0.001889304 |
| **Leaning Juniper [9]** | 64 | 0.47332869 | 1 | 0.050642479 | 0.078817734 | 0.001889304 |
| **Leaning Juniper [9]** | 65 | 0.47332869 | 1 | 0.050642479 | 0.078817734 | 0.001889304 |
| **Leaning Juniper [9]** | 66 | 0.47332869 | 1 | 0.050642479 | 0.078817734 | 0.001889304 |
| **Leaning Juniper [9]** | 67 | 0.47332869 | 1 | 0.050642479 | 0.078817734 | 0.001889304 |
| **Leaning Juniper [9]** | 68 | 0.47332869 | 1 | 0.050642479 | 0.078817734 | 0.001889304 |
| **Leaning Juniper [9]** | 69 | 0.47332869 | 1 | 0.050642479 | 0.078817734 | 0.001889304 |
| **Leaning Juniper [9]** | 70 | 0.47332869 | 1 | 0.050642479 | 0.078817734 | 0.001889304 |
| **Leaning Juniper [9]** | 71 | 0.47332869 | 1 | 0.050642479 | 0.078817734 | 0.001889304 |
| **Leaning Juniper [9]** | 72 | 0.47332869 | 1 | 0.050642479 | 0.078817734 | 0.001889304 |
| **Leaning Juniper [9]** | 73 | 0.47332869 | 1 | 0.050642479 | 0.078817734 | 0.001889304 |
| **Leaning Juniper [9]** | 74 | 0.47332869 | 1 | 0.050642479 | 0.078817734 | 0.001889304 |
| **Leaning Juniper [9]** | 75 | 0.47332869 | 1 | 0.050642479 | 0.078817734 | 0.001889304 |
| **Leaning Juniper [9]** | 76 | 0.47332869 | 1 | 0.050642479 | 0.078817734 | 0.001889304 |
| **Leaning Juniper [9]** | 77 | 0.47332869 | 1 | 0.050642479 | 0.078817734 | 0.001889304 |
| **Leaning Juniper [9]** | 78 | 0.47332869 | 1 | 0.050642479 | 0.078817734 | 0.001889304 |
| **Leaning Juniper [9]** | 79 | 0.47332869 | 1 | 0.050642479 | 0.078817734 | 0.001889304 |
| **Leaning Juniper [9]** | 80 | 0.47332869 | 1 | 0.050642479 | 0.078817734 | 0.001889304 |
| **Leaning Juniper [9]** | 81 | 0.47332869 | 1 | 0.050642479 | 0.078817734 | 0.001889304 |
| **Leaning Juniper [9]** | 82 | 0.47332869 | 1 | 0.050642479 | 0.078817734 | 0.001889304 |
| **Leaning Juniper [9]** | 83 | 0.47332869 | 1 | 0.050642479 | 0.078817734 | 0.001889304 |
| **Leaning Juniper [9]** | 84 | 0.47332869 | 1 | 0.050642479 | 0.078817734 | 0.001889304 |
| **Leaning Juniper [9]** | 85 | 0.47332869 | 1 | 0.050642479 | 0.078817734 | 0.001889304 |
| **Leaning Juniper [9]** | 86 | 0.47332869 | 1 | 0.050642479 | 0.078817734 | 0.001889304 |
| **Leaning Juniper [9]** | 87 | 0.47332869 | 1 | 0.050642479 | 0.078817734 | 0.001889304 |
| **Leaning Juniper [9]** | 88 | 0.47332869 | 1 | 0.050642479 | 0.078817734 | 0.001889304 |
| **Leaning Juniper [9]** | 89 | 0.47332869 | 1 | 0.050642479 | 0.078817734 | 0.001889304 |
| **Leaning Juniper [9]** | 90 | 0.47332869 | 1 | 0.050642479 | 0.078817734 | 0.001889304 |
| **Leaning Juniper [9]** | 91 | 0.47332869 | 1 | 0.050642479 | 0.078817734 | 0.001889304 |
| **Leaning Juniper [9]** | 92 | 0.47332869 | 1 | 0.050642479 | 0.078817734 | 0.001889304 |
| **Leaning Juniper [9]** | 93 | 0.47332869 | 1 | 0.050642479 | 0.078817734 | 0.001889304 |
| **Leaning Juniper [9]** | 94 | 0.47332869 | 1 | 0.050642479 | 0.078817734 | 0.001889304 |
| **Leaning Juniper [9]** | 95 | 0.47332869 | 1 | 0.050642479 | 0.078817734 | 0.001889304 |
| **Leaning Juniper [9]** | 96 | 0.47332869 | 1 | 0.050642479 | 0.078817734 | 0.001889304 |
| **Leaning Juniper [9]** | 97 | 0.47332869 | 1 | 0.050642479 | 0.078817734 | 0.001889304 |
| **Leaning Juniper [9]** | 98 | 0.47332869 | 1 | 0.050642479 | 0.078817734 | 0.001889304 |
| **Leaning Juniper [9]** | 99 | 0.47332869 | 1 | 0.050642479 | 0.078817734 | 0.001889304 |
| **Leaning Juniper [9]** | 100 | 0.47332869 | 1 | 0.050642479 | 0.078817734 | 0.001889304 |
| **Leaning Juniper [9]** | 101 | 0.47332869 | 1 | 0.050642479 | 0.078817734 | 0.001889304 |
| **Leaning Juniper [9]** | 102 | 0.47332869 | 1 | 0.050642479 | 0.078817734 | 0.001889304 |
| **Leaning Juniper [9]** | 103 | 0.47332869 | 1 | 0.050642479 | 0.078817734 | 0.001889304 |
| **Leaning Juniper [9]** | 104 | 0.47332869 | 1 | 0.050642479 | 0.078817734 | 0.001889304 |
| **Leaning Juniper [9]** | 105 | 0.47332869 | 1 | 0.050642479 | 0.078817734 | 0.001889304 |
| **Leaning Juniper [9]** | 106 | 0.47332869 | 1 | 0.050642479 | 0.078817734 | 0.001889304 |
| **Leaning Juniper [9]** | 107 | 0.47332869 | 1 | 0.050642479 | 0.078817734 | 0.001889304 |
| **Leaning Juniper [9]** | 108 | 0.47332869 | 1 | 0.050642479 | 0.078817734 | 0.001889304 |
| **Leaning Juniper [9]** | 109 | 0.47332869 | 1 | 0.050642479 | 0.078817734 | 0.001889304 |
| **Leaning Juniper [9]** | 110 | 0.47332869 | 1 | 0.050642479 | 0.078817734 | 0.001889304 |
| **Leaning Juniper [9]** | 111 | 0.47332869 | 1 | 0.050642479 | 0.078817734 | 0.001889304 |
| **Leaning Juniper [9]** | 112 | 0.47332869 | 1 | 0.050642479 | 0.078817734 | 0.001889304 |
| **Leaning Juniper [9]** | 113 | 0.47332869 | 1 | 0.050642479 | 0.078817734 | 0.001889304 |
| **Leaning Juniper [9]** | 114 | 0.47332869 | 1 | 0.050642479 | 0.078817734 | 0.001889304 |
| **Leaning Juniper [9]** | 115 | 0.47332869 | 1 | 0.050642479 | 0.078817734 | 0.001889304 |
| **Leaning Juniper [9]** | 116 | 0.47332869 | 1 | 0.050642479 | 0.078817734 | 0.001889304 |
| **Leaning Juniper [9]** | 117 | 0.47332869 | 1 | 0.050642479 | 0.078817734 | 0.001889304 |
| **Leaning Juniper [9]** | 118 | 0.47332869 | 1 | 0.050642479 | 0.078817734 | 0.001889304 |
| **Leaning Juniper [9]** | 119 | 0.47332869 | 1 | 0.050642479 | 0.078817734 | 0.001889304 |
| **Leaning Juniper [9]** | 120 | 0.47332869 | 1 | 0.050642479 | 0.078817734 | 0.001889304 |
| **Leaning Juniper [9]** | 121 | 0.47332869 | 1 | 0.050642479 | 0.078817734 | 0.001889304 |
| **Leaning Juniper [9]** | 122 | 0.47332869 | 1 | 0.050642479 | 0.078817734 | 0.001889304 |
| **Leaning Juniper [9]** | 123 | 0.47332869 | 0 | 0.050642479 | 0.078817734 | 6.64E-10 |
| **Leaning Juniper [9]** | 124 | 0.47332869 | 0 | 0.050642479 | 0.078817734 | 6.64E-10 |
| **Leaning Juniper [9]** | 125 | 0.47332869 | 0 | 0.050642479 | 0.078817734 | 6.64E-10 |
| **Leaning Juniper [9]** | 126 | 0.47332869 | 0 | 0.050642479 | 0.078817734 | 6.64E-10 |
| **Leaning Juniper [9]** | 127 | 0.47332869 | 0 | 0.050642479 | 0.078817734 | 6.64E-10 |
| **Leaning Juniper [9]** | 128 | 0.47332869 | 0 | 0.050642479 | 0.078817734 | 6.64E-10 |
| **Leaning Juniper [9]** | 129 | 0.47332869 | 0 | 0.050642479 | 0.078817734 | 6.64E-10 |
| **Leaning Juniper [9]** | 130 | 0.47332869 | 0 | 0.050642479 | 0.078817734 | 6.64E-10 |
| **Leaning Juniper [9]** | 131 | 0.47332869 | 0 | 0.050642479 | 0.078817734 | 6.64E-10 |
| **Leaning Juniper [9]** | 132 | 0.47332869 | 0 | 0.050642479 | 0.078817734 | 6.64E-10 |
| **Leaning Juniper [9]** | 133 | 0.47332869 | 0 | 0.050642479 | 0.078817734 | 6.64E-10 |
| **Leaning Juniper [9]** | 134 | 0.47332869 | 0 | 0.050642479 | 0.078817734 | 6.64E-10 |
| **Leaning Juniper [9]** | 135 | 0.47332869 | 0 | 0.050642479 | 0.078817734 | 6.64E-10 |
| **Leaning Juniper [9]** | 136 | 0.47332869 | 0 | 0.050642479 | 0.078817734 | 6.64E-10 |
| **Leaning Juniper [9]** | 137 | 0.47332869 | 0 | 0.050642479 | 0.078817734 | 6.64E-10 |
| **Leaning Juniper [9]** | 138 | 0.47332869 | 0 | 0.050642479 | 0.078817734 | 6.64E-10 |
| **Leaning Juniper [9]** | 139 | 0.47332869 | 0 | 0.050642479 | 0.078817734 | 6.64E-10 |
| **Leaning Juniper [9]** | 140 | 0.47332869 | 0 | 0.050642479 | 0.078817734 | 6.64E-10 |
| **Leaning Juniper [9]** | 141 | 0.47332869 | 0 | 0.050642479 | 0.078817734 | 6.64E-10 |
| **Leaning Juniper [9]** | 142 | 0.47332869 | 0 | 0.050642479 | 0.078817734 | 6.64E-10 |
| **Leaning Juniper [9]** | 143 | 0.47332869 | 0 | 0.050642479 | 0.078817734 | 6.64E-10 |
| **Leaning Juniper [9]** | 144 | 0.47332869 | 0 | 0.050642479 | 0.078817734 | 6.64E-10 |
| **Prairie Winds SD1 (2013-2014) [10]** | 3 | 0.37452127 | 1 | 0.081632653 | 0.083743842 | 0.002560314 |
| **Prairie Winds SD1 (2013-2014) [10]** | 4 | 0.37452127 | 1 | 0.081632653 | 0.083743842 | 0.002560314 |
| **Prairie Winds SD1 (2013-2014) [10]** | 5 | 0.37452127 | 1 | 0.081632653 | 0.083743842 | 0.002560314 |
| **Prairie Winds SD1 (2013-2014) [10]** | 6 | 0.37452127 | 1 | 0.081632653 | 0.083743842 | 0.002560314 |
| **Prairie Winds SD1 (2013-2014) [10]** | 7 | 0.37452127 | 1 | 0.081632653 | 0.083743842 | 0.002560314 |
| **Prairie Winds SD1 (2013-2014) [10]** | 8 | 0.37452127 | 1 | 0.081632653 | 0.083743842 | 0.002560314 |
| **Prairie Winds SD1 (2013-2014) [10]** | 9 | 0.37452127 | 1 | 0.081632653 | 0.083743842 | 0.002560314 |
| **Prairie Winds SD1 (2013-2014) [10]** | 10 | 0.37452127 | 1 | 0.081632653 | 0.083743842 | 0.002560314 |
| **Prairie Winds SD1 (2013-2014) [10]** | 11 | 0.37452127 | 1 | 0.081632653 | 0.083743842 | 0.002560314 |
| **Prairie Winds SD1 (2013-2014) [10]** | 12 | 0.37452127 | 1 | 0.081632653 | 0.083743842 | 0.002560314 |
| **Prairie Winds SD1 (2013-2014) [10]** | 13 | 0.37452127 | 1 | 0.081632653 | 0.083743842 | 0.002560314 |
| **Prairie Winds SD1 (2013-2014) [10]** | 14 | 0.37452127 | 1 | 0.081632653 | 0.083743842 | 0.002560314 |
| **Prairie Winds SD1 (2013-2014) [10]** | 15 | 0.37452127 | 1 | 0.081632653 | 0.083743842 | 0.002560314 |
| **Prairie Winds SD1 (2013-2014) [10]** | 16 | 0.37452127 | 1 | 0.081632653 | 0.083743842 | 0.002560314 |
| **Prairie Winds SD1 (2013-2014) [10]** | 17 | 0.37452127 | 1 | 0.081632653 | 0.083743842 | 0.002560314 |
| **Prairie Winds SD1 (2013-2014) [10]** | 18 | 0.37452127 | 1 | 0.081632653 | 0.083743842 | 0.002560314 |
| **Prairie Winds SD1 (2013-2014) [10]** | 19 | 0.37452127 | 1 | 0.081632653 | 0.083743842 | 0.002560314 |
| **Prairie Winds SD1 (2013-2014) [10]** | 20 | 0.37452127 | 1 | 0.081632653 | 0.083743842 | 0.002560314 |
| **Prairie Winds SD1 (2013-2014) [10]** | 21 | 0.37452127 | 1 | 0.081632653 | 0.083743842 | 0.002560314 |
| **Prairie Winds SD1 (2013-2014) [10]** | 22 | 0.37452127 | 1 | 0.081632653 | 0.083743842 | 0.002560314 |
| **Prairie Winds SD1 (2013-2014) [10]** | 23 | 0.37452127 | 1 | 0.081632653 | 0.083743842 | 0.002560314 |
| **Prairie Winds SD1 (2013-2014) [10]** | 24 | 0.37452127 | 1 | 0.081632653 | 0.083743842 | 0.002560314 |
| **Prairie Winds SD1 (2013-2014) [10]** | 25 | 0.37452127 | 1 | 0.081632653 | 0.083743842 | 0.002560314 |
| **Prairie Winds SD1 (2013-2014) [10]** | 26 | 0.37452127 | 1 | 0.081632653 | 0.083743842 | 0.002560314 |
| **Prairie Winds SD1 (2013-2014) [10]** | 27 | 0.37452127 | 1 | 0.081632653 | 0.083743842 | 0.002560314 |
| **Prairie Winds SD1 (2013-2014) [10]** | 28 | 0.37452127 | 1 | 0.081632653 | 0.083743842 | 0.002560314 |
| **Prairie Winds SD1 (2013-2014) [10]** | 29 | 0.37452127 | 1 | 0.081632653 | 0.083743842 | 0.002560314 |
| **Prairie Winds SD1 (2013-2014) [10]** | 30 | 0.37452127 | 1 | 0.081632653 | 0.083743842 | 0.002560314 |
| **Prairie Winds SD1 (2013-2014) [10]** | 31 | 0.37452127 | 1 | 0.081632653 | 0.083743842 | 0.002560314 |
| **Prairie Winds SD1 (2013-2014) [10]** | 32 | 0.37452127 | 1 | 0.081632653 | 0.083743842 | 0.002560314 |
| **Prairie Winds SD1 (2013-2014) [10]** | 33 | 0.37452127 | 1 | 0.081632653 | 0.083743842 | 0.002560314 |
| **Prairie Winds SD1 (2013-2014) [10]** | 34 | 0.37452127 | 1 | 0.081632653 | 0.083743842 | 0.002560314 |
| **Prairie Winds SD1 (2013-2014) [10]** | 35 | 0.37452127 | 1 | 0.081632653 | 0.083743842 | 0.002560314 |
| **Prairie Winds SD1 (2013-2014) [10]** | 36 | 0.37452127 | 1 | 0.081632653 | 0.083743842 | 0.002560314 |
| **Prairie Winds SD1 (2013-2014) [10]** | 37 | 0.37452127 | 1 | 0.081632653 | 0.083743842 | 0.002560314 |
| **Prairie Winds SD1 (2013-2014) [10]** | 38 | 0.37452127 | 1 | 0.081632653 | 0.083743842 | 0.002560314 |
| **Prairie Winds SD1 (2013-2014) [10]** | 39 | 0.37452127 | 1 | 0.081632653 | 0.083743842 | 0.002560314 |
| **Prairie Winds SD1 (2013-2014) [10]** | 40 | 0.37452127 | 1 | 0.081632653 | 0.083743842 | 0.002560314 |
| **Prairie Winds SD1 (2013-2014) [10]** | 41 | 0.37452127 | 1 | 0.081632653 | 0.083743842 | 0.002560314 |
| **Prairie Winds SD1 (2013-2014) [10]** | 42 | 0.37452127 | 1 | 0.081632653 | 0.083743842 | 0.002560314 |
| **Prairie Winds SD1 (2013-2014) [10]** | 43 | 0.37452127 | 1 | 0.081632653 | 0.083743842 | 0.002560314 |
| **Prairie Winds SD1 (2013-2014) [10]** | 44 | 0.37452127 | 1 | 0.081632653 | 0.083743842 | 0.002560314 |
| **Prairie Winds SD1 (2013-2014) [10]** | 45 | 0.37452127 | 1 | 0.081632653 | 0.083743842 | 0.002560314 |
| **Prairie Winds SD1 (2013-2014) [10]** | 46 | 0.37452127 | 1 | 0.081632653 | 0.083743842 | 0.002560314 |
| **Prairie Winds SD1 (2013-2014) [10]** | 47 | 0.37452127 | 1 | 0.081632653 | 0.083743842 | 0.002560314 |
| **Prairie Winds SD1 (2013-2014) [10]** | 48 | 0.37452127 | 1 | 0.081632653 | 0.083743842 | 0.002560314 |
| **Prairie Winds SD1 (2013-2014) [10]** | 49 | 0.37452127 | 1 | 0.081632653 | 0.083743842 | 0.002560314 |
| **Prairie Winds SD1 (2013-2014) [10]** | 50 | 0.37452127 | 1 | 0.081632653 | 0.083743842 | 0.002560314 |
| **Prairie Winds SD1 (2013-2014) [10]** | 51 | 0.37452127 | 1 | 0.081632653 | 0.083743842 | 0.002560314 |
| **Prairie Winds SD1 (2013-2014) [10]** | 52 | 0.37452127 | 1 | 0.081632653 | 0.083743842 | 0.002560314 |
| **Prairie Winds SD1 (2013-2014) [10]** | 53 | 0.37452127 | 1 | 0.081632653 | 0.083743842 | 0.002560314 |
| **Prairie Winds SD1 (2013-2014) [10]** | 54 | 0.37452127 | 1 | 0.081632653 | 0.083743842 | 0.002560314 |
| **Prairie Winds SD1 (2013-2014) [10]** | 55 | 0.37452127 | 1 | 0.081632653 | 0.083743842 | 0.002560314 |
| **Prairie Winds SD1 (2013-2014) [10]** | 56 | 0.37452127 | 1 | 0.081632653 | 0.083743842 | 0.002560314 |
| **Prairie Winds SD1 (2013-2014) [10]** | 57 | 0.37452127 | 1 | 0.081632653 | 0.083743842 | 0.002560314 |
| **Prairie Winds SD1 (2013-2014) [10]** | 58 | 0.37452127 | 1 | 0.081632653 | 0.083743842 | 0.002560314 |
| **Prairie Winds SD1 (2013-2014) [10]** | 59 | 0.37452127 | 1 | 0.081632653 | 0.083743842 | 0.002560314 |
| **Prairie Winds SD1 (2013-2014) [10]** | 60 | 0.37452127 | 1 | 0.081632653 | 0.083743842 | 0.002560314 |
| **Prairie Winds SD1 (2013-2014) [10]** | 61 | 0.37452127 | 1 | 0.081632653 | 0.083743842 | 0.002560314 |
| **Prairie Winds SD1 (2013-2014) [10]** | 62 | 0.37452127 | 1 | 0.081632653 | 0.083743842 | 0.002560314 |
| **Prairie Winds SD1 (2013-2014) [10]** | 63 | 0.37452127 | 1 | 0.081632653 | 0.083743842 | 0.002560314 |
| **Prairie Winds SD1 (2013-2014) [10]** | 64 | 0.37452127 | 1 | 0.081632653 | 0.083743842 | 0.002560314 |
| **Prairie Winds SD1 (2013-2014) [10]** | 65 | 0.37452127 | 1 | 0.081632653 | 0.083743842 | 0.002560314 |
| **Prairie Winds SD1 (2013-2014) [10]** | 66 | 0.37452127 | 1 | 0.081632653 | 0.083743842 | 0.002560314 |
| **Prairie Winds SD1 (2013-2014) [10]** | 67 | 0.37452127 | 1 | 0.081632653 | 0.083743842 | 0.002560314 |
| **Prairie Winds SD1 (2013-2014) [10]** | 68 | 0.37452127 | 1 | 0.081632653 | 0.083743842 | 0.002560314 |
| **Prairie Winds SD1 (2013-2014) [10]** | 69 | 0.37452127 | 1 | 0.081632653 | 0.083743842 | 0.002560314 |
| **Prairie Winds SD1 (2013-2014) [10]** | 70 | 0.37452127 | 1 | 0.081632653 | 0.083743842 | 0.002560314 |
| **Prairie Winds SD1 (2013-2014) [10]** | 71 | 0.37452127 | 1 | 0.081632653 | 0.083743842 | 0.002560314 |
| **Prairie Winds SD1 (2013-2014) [10]** | 72 | 0.37452127 | 1 | 0.081632653 | 0.083743842 | 0.002560314 |
| **Prairie Winds SD1 (2013-2014) [10]** | 73 | 0.37452127 | 1 | 0.081632653 | 0.083743842 | 0.002560314 |
| **Prairie Winds SD1 (2013-2014) [10]** | 74 | 0.37452127 | 1 | 0.081632653 | 0.083743842 | 0.002560314 |
| **Prairie Winds SD1 (2013-2014) [10]** | 75 | 0.37452127 | 1 | 0.081632653 | 0.083743842 | 0.002560314 |
| **Prairie Winds SD1 (2013-2014) [10]** | 76 | 0.37452127 | 1 | 0.081632653 | 0.083743842 | 0.002560314 |
| **Prairie Winds SD1 (2013-2014) [10]** | 77 | 0.37452127 | 1 | 0.081632653 | 0.083743842 | 0.002560314 |
| **Prairie Winds SD1 (2013-2014) [10]** | 78 | 0.37452127 | 1 | 0.081632653 | 0.083743842 | 0.002560314 |
| **Prairie Winds SD1 (2013-2014) [10]** | 79 | 0.37452127 | 1 | 0.081632653 | 0.083743842 | 0.002560314 |
| **Prairie Winds SD1 (2013-2014) [10]** | 80 | 0.37452127 | 1 | 0.081632653 | 0.083743842 | 0.002560314 |
| **Prairie Winds SD1 (2013-2014) [10]** | 81 | 0.37452127 | 1 | 0.081632653 | 0.083743842 | 0.002560314 |
| **Prairie Winds SD1 (2013-2014) [10]** | 82 | 0.37452127 | 1 | 0.081632653 | 0.083743842 | 0.002560314 |
| **Prairie Winds SD1 (2013-2014) [10]** | 83 | 0.37452127 | 1 | 0.081632653 | 0.083743842 | 0.002560314 |
| **Prairie Winds SD1 (2013-2014) [10]** | 84 | 0.37452127 | 1 | 0.081632653 | 0.083743842 | 0.002560314 |
| **Prairie Winds SD1 (2013-2014) [10]** | 85 | 0.37452127 | 1 | 0.081632653 | 0.083743842 | 0.002560314 |
| **Prairie Winds SD1 (2013-2014) [10]** | 86 | 0.37452127 | 1 | 0.081632653 | 0.083743842 | 0.002560314 |
| **Prairie Winds SD1 (2013-2014) [10]** | 87 | 0.37452127 | 1 | 0.081632653 | 0.083743842 | 0.002560314 |
| **Prairie Winds SD1 (2013-2014) [10]** | 88 | 0.37452127 | 1 | 0.081632653 | 0.083743842 | 0.002560314 |
| **Prairie Winds SD1 (2013-2014) [10]** | 89 | 0.37452127 | 1 | 0.081632653 | 0.083743842 | 0.002560314 |
| **Prairie Winds SD1 (2013-2014) [10]** | 90 | 0.37452127 | 1 | 0.081632653 | 0.083743842 | 0.002560314 |
| **Prairie Winds SD1 (2013-2014) [10]** | 91 | 0.37452127 | 1 | 0.081632653 | 0.083743842 | 0.002560314 |
| **Prairie Winds SD1 (2013-2014) [10]** | 92 | 0.37452127 | 1 | 0.081632653 | 0.083743842 | 0.002560314 |
| **Prairie Winds SD1 (2013-2014) [10]** | 93 | 0.37452127 | 1 | 0.081632653 | 0.083743842 | 0.002560314 |
| **Prairie Winds SD1 (2013-2014) [10]** | 94 | 0.37452127 | 1 | 0.081632653 | 0.083743842 | 0.002560314 |
| **Prairie Winds SD1 (2013-2014) [10]** | 95 | 0.37452127 | 1 | 0.081632653 | 0.083743842 | 0.002560314 |
| **Prairie Winds SD1 (2013-2014) [10]** | 96 | 0.37452127 | 1 | 0.081632653 | 0.083743842 | 0.002560314 |
| **Prairie Winds SD1 (2013-2014) [10]** | 97 | 0.37452127 | 1 | 0.081632653 | 0.083743842 | 0.002560314 |
| **Prairie Winds SD1 (2013-2014) [10]** | 98 | 0.37452127 | 1 | 0.081632653 | 0.083743842 | 0.002560314 |
| **Prairie Winds SD1 (2013-2014) [10]** | 99 | 0.37452127 | 1 | 0.081632653 | 0.083743842 | 0.002560314 |
| **Prairie Winds SD1 (2013-2014) [10]** | 100 | 0.37452127 | 1 | 0.081632653 | 0.083743842 | 0.002560314 |
| **Prairie Winds SD1 (2013-2014) [10]** | 101 | 0.37452127 | 1 | 0.081632653 | 0.083743842 | 0.002560314 |
| **Prairie Winds SD1 (2013-2014) [10]** | 102 | 0.37452127 | 1 | 0.081632653 | 0.083743842 | 0.002560314 |
| **Prairie Winds SD1 (2013-2014) [10]** | 103 | 0.37452127 | 0 | 0.081632653 | 0.083743842 | 6.64E-10 |
| **Prairie Winds SD1 (2013-2014) [10]** | 104 | 0.37452127 | 0 | 0.081632653 | 0.083743842 | 6.64E-10 |
| **Prairie Winds SD1 (2013-2014) [10]** | 105 | 0.37452127 | 0 | 0.081632653 | 0.083743842 | 6.64E-10 |
| **Prairie Winds SD1 (2013-2014) [10]** | 106 | 0.37452127 | 0 | 0.081632653 | 0.083743842 | 6.64E-10 |
| **Prairie Winds SD1 (2013-2014) [10]** | 107 | 0.37452127 | 0 | 0.081632653 | 0.083743842 | 6.64E-10 |
| **Prairie Winds SD1 (2013-2014) [10]** | 108 | 0.37452127 | 0 | 0.081632653 | 0.083743842 | 6.64E-10 |
| **Prairie Winds SD1 (2013-2014) [10]** | 109 | 0.37452127 | 0 | 0.081632653 | 0.083743842 | 6.64E-10 |
| **Prairie Winds SD1 (2013-2014) [10]** | 110 | 0.37452127 | 0 | 0.081632653 | 0.083743842 | 6.64E-10 |
| **Prairie Winds SD1 (2013-2014) [10]** | 111 | 0.37452127 | 0 | 0.081632653 | 0.083743842 | 6.64E-10 |
| **Prairie Winds SD1 (2013-2014) [10]** | 112 | 0.37452127 | 0 | 0.081632653 | 0.083743842 | 6.64E-10 |
| **Prairie Winds SD1 (2013-2014) [10]** | 113 | 0.37452127 | 0 | 0.081632653 | 0.083743842 | 6.64E-10 |
| **Prairie Winds SD1 (2013-2014) [10]** | 114 | 0.37452127 | 0 | 0.081632653 | 0.083743842 | 6.64E-10 |
| **Prairie Winds SD1 (2013-2014) [10]** | 115 | 0.37452127 | 0 | 0.081632653 | 0.083743842 | 6.64E-10 |
| **Prairie Winds SD1 (2013-2014) [10]** | 116 | 0.37452127 | 0 | 0.081632653 | 0.083743842 | 6.64E-10 |
| **Prairie Winds SD1 (2013-2014) [10]** | 117 | 0.37452127 | 0 | 0.081632653 | 0.083743842 | 6.64E-10 |
| **Prairie Winds SD1 (2013-2014) [10]** | 118 | 0.37452127 | 0 | 0.081632653 | 0.083743842 | 6.64E-10 |
| **Prairie Winds SD1 (2013-2014) [10]** | 119 | 0.37452127 | 0 | 0.081632653 | 0.083743842 | 6.64E-10 |
| **Prairie Winds SD1 (2013-2014) [10]** | 120 | 0.37452127 | 0 | 0.081632653 | 0.083743842 | 6.64E-10 |
| **Prairie Winds SD1 (2013-2014) [10]** | 121 | 0.37452127 | 0 | 0.081632653 | 0.083743842 | 6.64E-10 |
| **Prairie Winds SD1 (2013-2014) [10]** | 122 | 0.37452127 | 0 | 0.081632653 | 0.083743842 | 6.64E-10 |
| **Prairie Winds SD1 (2013-2014) [10]** | 123 | 0.37452127 | 0 | 0.081632653 | 0.083743842 | 6.64E-10 |
| **Prairie Winds SD1 (2013-2014) [10]** | 124 | 0.37452127 | 0 | 0.081632653 | 0.083743842 | 6.64E-10 |
| **Prairie Winds SD1 (2013-2014) [10]** | 125 | 0.37452127 | 0 | 0.081632653 | 0.083743842 | 6.64E-10 |
| **Prairie Winds SD1 (2013-2014) [10]** | 126 | 0.37452127 | 0 | 0.081632653 | 0.083743842 | 6.64E-10 |
| **Prairie Winds SD1 (2013-2014) [10]** | 127 | 0.37452127 | 0 | 0.081632653 | 0.083743842 | 6.64E-10 |
| **Prairie Winds SD1 (2013-2014) [10]** | 128 | 0.37452127 | 0 | 0.081632653 | 0.083743842 | 6.64E-10 |
| **Prairie Winds SD1 (2013-2014) [10]** | 129 | 0.37452127 | 0 | 0.081632653 | 0.083743842 | 6.64E-10 |
| **Prairie Winds SD1 (2013-2014) [10]** | 130 | 0.37452127 | 0 | 0.081632653 | 0.083743842 | 6.64E-10 |
| **Prairie Winds SD1 (2013-2014) [10]** | 131 | 0.37452127 | 0 | 0.081632653 | 0.083743842 | 6.64E-10 |
| **Prairie Winds SD1 (2013-2014) [10]** | 132 | 0.37452127 | 0 | 0.081632653 | 0.083743842 | 6.64E-10 |
| **Prairie Winds SD1 (2013-2014) [10]** | 133 | 0.37452127 | 0 | 0.081632653 | 0.083743842 | 6.64E-10 |
| **Prairie Winds SD1 (2013-2014) [10]** | 134 | 0.37452127 | 0 | 0.081632653 | 0.083743842 | 6.64E-10 |
| **Prairie Winds SD1 (2013-2014) [10]** | 135 | 0.37452127 | 0 | 0.081632653 | 0.083743842 | 6.64E-10 |
| **Prairie Winds SD1 (2013-2014) [10]** | 136 | 0.37452127 | 0 | 0.081632653 | 0.083743842 | 6.64E-10 |
| **Prairie Winds SD1 (2013-2014) [10]** | 137 | 0.37452127 | 0 | 0.081632653 | 0.083743842 | 6.64E-10 |
| **Prairie Winds SD1 (2013-2014) [10]** | 138 | 0.37452127 | 0 | 0.081632653 | 0.083743842 | 6.64E-10 |
| **Prairie Winds SD1 (2013-2014) [10]** | 139 | 0.37452127 | 0 | 0.081632653 | 0.083743842 | 6.64E-10 |
| **Prairie Winds SD1 (2013-2014) [10]** | 140 | 0.37452127 | 0 | 0.081632653 | 0.083743842 | 6.64E-10 |
| **Prairie Winds SD1 (2013-2014) [10]** | 141 | 0.37452127 | 0 | 0.081632653 | 0.083743842 | 6.64E-10 |
| **Prairie Winds SD1 (2013-2014) [10]** | 142 | 0.37452127 | 0 | 0.081632653 | 0.083743842 | 6.64E-10 |
| **Prairie Winds SD1 (2013-2014) [10]** | 143 | 0.37452127 | 0 | 0.081632653 | 0.083743842 | 6.64E-10 |
| **Prairie Winds SD1 (2013-2014) [10]** | 144 | 0.37452127 | 0 | 0.081632653 | 0.083743842 | 6.64E-10 |
| **Red Hills [11]** | 3 | 0.67628181 | 1 | 0.061980348 | 0.019704433 | 0.000825935 |
| **Red Hills [11]** | 4 | 0.67628181 | 1 | 0.061980348 | 0.019704433 | 0.000825935 |
| **Red Hills [11]** | 5 | 0.67628181 | 1 | 0.061980348 | 0.019704433 | 0.000825935 |
| **Red Hills [11]** | 6 | 0.67628181 | 1 | 0.061980348 | 0.019704433 | 0.000825935 |
| **Red Hills [11]** | 7 | 0.67628181 | 1 | 0.061980348 | 0.019704433 | 0.000825935 |
| **Red Hills [11]** | 8 | 0.67628181 | 1 | 0.061980348 | 0.019704433 | 0.000825935 |
| **Red Hills [11]** | 9 | 0.67628181 | 1 | 0.061980348 | 0.019704433 | 0.000825935 |
| **Red Hills [11]** | 10 | 0.67628181 | 1 | 0.061980348 | 0.019704433 | 0.000825935 |
| **Red Hills [11]** | 11 | 0.67628181 | 1 | 0.061980348 | 0.019704433 | 0.000825935 |
| **Red Hills [11]** | 12 | 0.67628181 | 1 | 0.061980348 | 0.019704433 | 0.000825935 |
| **Red Hills [11]** | 13 | 0.67628181 | 1 | 0.061980348 | 0.019704433 | 0.000825935 |
| **Red Hills [11]** | 14 | 0.67628181 | 1 | 0.061980348 | 0.019704433 | 0.000825935 |
| **Red Hills [11]** | 15 | 0.67628181 | 1 | 0.061980348 | 0.019704433 | 0.000825935 |
| **Red Hills [11]** | 16 | 0.67628181 | 1 | 0.061980348 | 0.019704433 | 0.000825935 |
| **Red Hills [11]** | 17 | 0.67628181 | 1 | 0.061980348 | 0.019704433 | 0.000825935 |
| **Red Hills [11]** | 18 | 0.67628181 | 1 | 0.061980348 | 0.019704433 | 0.000825935 |
| **Red Hills [11]** | 19 | 0.67628181 | 1 | 0.061980348 | 0.019704433 | 0.000825935 |
| **Red Hills [11]** | 20 | 0.67628181 | 1 | 0.061980348 | 0.019704433 | 0.000825935 |
| **Red Hills [11]** | 21 | 0.67628181 | 1 | 0.061980348 | 0.019704433 | 0.000825935 |
| **Red Hills [11]** | 22 | 0.67628181 | 1 | 0.061980348 | 0.019704433 | 0.000825935 |
| **Red Hills [11]** | 23 | 0.67628181 | 1 | 0.061980348 | 0.019704433 | 0.000825935 |
| **Red Hills [11]** | 24 | 0.67628181 | 1 | 0.061980348 | 0.019704433 | 0.000825935 |
| **Red Hills [11]** | 25 | 0.67628181 | 1 | 0.061980348 | 0.019704433 | 0.000825935 |
| **Red Hills [11]** | 26 | 0.67628181 | 1 | 0.061980348 | 0.019704433 | 0.000825935 |
| **Red Hills [11]** | 27 | 0.67628181 | 1 | 0.061980348 | 0.019704433 | 0.000825935 |
| **Red Hills [11]** | 28 | 0.67628181 | 1 | 0.061980348 | 0.019704433 | 0.000825935 |
| **Red Hills [11]** | 29 | 0.67628181 | 1 | 0.061980348 | 0.019704433 | 0.000825935 |
| **Red Hills [11]** | 30 | 0.67628181 | 1 | 0.061980348 | 0.019704433 | 0.000825935 |
| **Red Hills [11]** | 31 | 0.67628181 | 1 | 0.061980348 | 0.019704433 | 0.000825935 |
| **Red Hills [11]** | 32 | 0.67628181 | 1 | 0.061980348 | 0.019704433 | 0.000825935 |
| **Red Hills [11]** | 33 | 0.67628181 | 1 | 0.061980348 | 0.019704433 | 0.000825935 |
| **Red Hills [11]** | 34 | 0.67628181 | 1 | 0.061980348 | 0.019704433 | 0.000825935 |
| **Red Hills [11]** | 35 | 0.67628181 | 1 | 0.061980348 | 0.019704433 | 0.000825935 |
| **Red Hills [11]** | 36 | 0.67628181 | 1 | 0.061980348 | 0.019704433 | 0.000825935 |
| **Red Hills [11]** | 37 | 0.67628181 | 1 | 0.061980348 | 0.019704433 | 0.000825935 |
| **Red Hills [11]** | 38 | 0.67628181 | 1 | 0.061980348 | 0.019704433 | 0.000825935 |
| **Red Hills [11]** | 39 | 0.67628181 | 1 | 0.061980348 | 0.019704433 | 0.000825935 |
| **Red Hills [11]** | 40 | 0.67628181 | 1 | 0.061980348 | 0.019704433 | 0.000825935 |
| **Red Hills [11]** | 41 | 0.67628181 | 1 | 0.061980348 | 0.019704433 | 0.000825935 |
| **Red Hills [11]** | 42 | 0.67628181 | 1 | 0.061980348 | 0.019704433 | 0.000825935 |
| **Red Hills [11]** | 43 | 0.67628181 | 1 | 0.061980348 | 0.019704433 | 0.000825935 |
| **Red Hills [11]** | 44 | 0.67628181 | 1 | 0.061980348 | 0.019704433 | 0.000825935 |
| **Red Hills [11]** | 45 | 0.67628181 | 1 | 0.061980348 | 0.019704433 | 0.000825935 |
| **Red Hills [11]** | 46 | 0.67628181 | 1 | 0.061980348 | 0.019704433 | 0.000825935 |
| **Red Hills [11]** | 47 | 0.67628181 | 1 | 0.061980348 | 0.019704433 | 0.000825935 |
| **Red Hills [11]** | 48 | 0.67628181 | 1 | 0.061980348 | 0.019704433 | 0.000825935 |
| **Red Hills [11]** | 49 | 0.67628181 | 1 | 0.061980348 | 0.019704433 | 0.000825935 |
| **Red Hills [11]** | 50 | 0.67628181 | 1 | 0.061980348 | 0.019704433 | 0.000825935 |
| **Red Hills [11]** | 51 | 0.67628181 | 1 | 0.061980348 | 0.019704433 | 0.000825935 |
| **Red Hills [11]** | 52 | 0.67628181 | 1 | 0.061980348 | 0.019704433 | 0.000825935 |
| **Red Hills [11]** | 53 | 0.67628181 | 0 | 0.061980348 | 0.019704433 | 6.64E-10 |
| **Red Hills [11]** | 54 | 0.67628181 | 0 | 0.061980348 | 0.019704433 | 6.64E-10 |
| **Red Hills [11]** | 55 | 0.67628181 | 0 | 0.061980348 | 0.019704433 | 6.64E-10 |
| **Red Hills [11]** | 56 | 0.67628181 | 0 | 0.061980348 | 0.019704433 | 6.64E-10 |
| **Red Hills [11]** | 57 | 0.67628181 | 0 | 0.061980348 | 0.019704433 | 6.64E-10 |
| **Red Hills [11]** | 58 | 0.67628181 | 0 | 0.061980348 | 0.019704433 | 6.64E-10 |
| **Red Hills [11]** | 59 | 0.67628181 | 0 | 0.061980348 | 0.019704433 | 6.64E-10 |
| **Red Hills [11]** | 60 | 0.67628181 | 0 | 0.061980348 | 0.019704433 | 6.64E-10 |
| **Red Hills [11]** | 61 | 0.67628181 | 0 | 0.061980348 | 0.019704433 | 6.64E-10 |
| **Red Hills [11]** | 62 | 0.67628181 | 0 | 0.061980348 | 0.019704433 | 6.64E-10 |
| **Red Hills [11]** | 63 | 0.67628181 | 0 | 0.061980348 | 0.019704433 | 6.64E-10 |
| **Red Hills [11]** | 64 | 0.67628181 | 0 | 0.061980348 | 0.019704433 | 6.64E-10 |
| **Red Hills [11]** | 65 | 0.67628181 | 0 | 0.061980348 | 0.019704433 | 6.64E-10 |
| **Red Hills [11]** | 66 | 0.67628181 | 0 | 0.061980348 | 0.019704433 | 6.64E-10 |
| **Red Hills [11]** | 67 | 0.67628181 | 0 | 0.061980348 | 0.019704433 | 6.64E-10 |
| **Red Hills [11]** | 68 | 0.67628181 | 0 | 0.061980348 | 0.019704433 | 6.64E-10 |
| **Red Hills [11]** | 69 | 0.67628181 | 0 | 0.061980348 | 0.019704433 | 6.64E-10 |
| **Red Hills [11]** | 70 | 0.67628181 | 0 | 0.061980348 | 0.019704433 | 6.64E-10 |
| **Red Hills [11]** | 71 | 0.67628181 | 0 | 0.061980348 | 0.019704433 | 6.64E-10 |
| **Red Hills [11]** | 72 | 0.67628181 | 0 | 0.061980348 | 0.019704433 | 6.64E-10 |
| **Red Hills [11]** | 73 | 0.67628181 | 0 | 0.061980348 | 0.019704433 | 6.64E-10 |
| **Red Hills [11]** | 74 | 0.67628181 | 0 | 0.061980348 | 0.019704433 | 6.64E-10 |
| **Red Hills [11]** | 75 | 0.67628181 | 0 | 0.061980348 | 0.019704433 | 6.64E-10 |
| **Red Hills [11]** | 76 | 0.67628181 | 0 | 0.061980348 | 0.019704433 | 6.64E-10 |
| **Red Hills [11]** | 77 | 0.67628181 | 0 | 0.061980348 | 0.019704433 | 6.64E-10 |
| **Red Hills [11]** | 78 | 0.67628181 | 0 | 0.061980348 | 0.019704433 | 6.64E-10 |
| **Red Hills [11]** | 79 | 0.67628181 | 0 | 0.061980348 | 0.019704433 | 6.64E-10 |
| **Red Hills [11]** | 80 | 0.67628181 | 0 | 0.061980348 | 0.019704433 | 6.64E-10 |
| **Red Hills [11]** | 81 | 0.67628181 | 0 | 0.061980348 | 0.019704433 | 6.64E-10 |
| **Red Hills [11]** | 82 | 0.67628181 | 0 | 0.061980348 | 0.019704433 | 6.64E-10 |
| **Red Hills [11]** | 83 | 0.67628181 | 0 | 0.061980348 | 0.019704433 | 6.64E-10 |
| **Red Hills [11]** | 84 | 0.67628181 | 0 | 0.061980348 | 0.019704433 | 6.64E-10 |
| **Red Hills [11]** | 85 | 0.67628181 | 0 | 0.061980348 | 0.019704433 | 6.64E-10 |
| **Red Hills [11]** | 86 | 0.67628181 | 0 | 0.061980348 | 0.019704433 | 6.64E-10 |
| **Red Hills [11]** | 87 | 0.67628181 | 0 | 0.061980348 | 0.019704433 | 6.64E-10 |
| **Red Hills [11]** | 88 | 0.67628181 | 0 | 0.061980348 | 0.019704433 | 6.64E-10 |
| **Red Hills [11]** | 89 | 0.67628181 | 0 | 0.061980348 | 0.019704433 | 6.64E-10 |
| **Red Hills [11]** | 90 | 0.67628181 | 0 | 0.061980348 | 0.019704433 | 6.64E-10 |
| **Red Hills [11]** | 91 | 0.67628181 | 0 | 0.061980348 | 0.019704433 | 6.64E-10 |
| **Red Hills [11]** | 92 | 0.67628181 | 0 | 0.061980348 | 0.019704433 | 6.64E-10 |
| **Red Hills [11]** | 93 | 0.67628181 | 0 | 0.061980348 | 0.019704433 | 6.64E-10 |
| **Red Hills [11]** | 94 | 0.67628181 | 0 | 0.061980348 | 0.019704433 | 6.64E-10 |
| **Red Hills [11]** | 95 | 0.67628181 | 0 | 0.061980348 | 0.019704433 | 6.64E-10 |
| **Red Hills [11]** | 96 | 0.67628181 | 0 | 0.061980348 | 0.019704433 | 6.64E-10 |
| **Red Hills [11]** | 97 | 0.67628181 | 0 | 0.061980348 | 0.019704433 | 6.64E-10 |
| **Red Hills [11]** | 98 | 0.67628181 | 0 | 0.061980348 | 0.019704433 | 6.64E-10 |
| **Red Hills [11]** | 99 | 0.67628181 | 0 | 0.061980348 | 0.019704433 | 6.64E-10 |
| **Red Hills [11]** | 100 | 0.67628181 | 0 | 0.061980348 | 0.019704433 | 6.64E-10 |
| **Red Hills [11]** | 101 | 0.67628181 | 0 | 0.061980348 | 0.019704433 | 6.64E-10 |
| **Red Hills [11]** | 102 | 0.67628181 | 0 | 0.061980348 | 0.019704433 | 6.64E-10 |
| **Red Hills [11]** | 103 | 0.67628181 | 0 | 0.061980348 | 0.019704433 | 6.64E-10 |
| **Red Hills [11]** | 104 | 0.67628181 | 0 | 0.061980348 | 0.019704433 | 6.64E-10 |
| **Red Hills [11]** | 105 | 0.67628181 | 0 | 0.061980348 | 0.019704433 | 6.64E-10 |
| **Red Hills [11]** | 106 | 0.67628181 | 0 | 0.061980348 | 0.019704433 | 6.64E-10 |
| **Red Hills [11]** | 107 | 0.67628181 | 0 | 0.061980348 | 0.019704433 | 6.64E-10 |
| **Red Hills [11]** | 108 | 0.67628181 | 0 | 0.061980348 | 0.019704433 | 6.64E-10 |
| **Red Hills [11]** | 109 | 0.67628181 | 0 | 0.061980348 | 0.019704433 | 6.64E-10 |
| **Red Hills [11]** | 110 | 0.67628181 | 0 | 0.061980348 | 0.019704433 | 6.64E-10 |
| **Red Hills [11]** | 111 | 0.67628181 | 0 | 0.061980348 | 0.019704433 | 6.64E-10 |
| **Red Hills [11]** | 112 | 0.67628181 | 0 | 0.061980348 | 0.019704433 | 6.64E-10 |
| **Red Hills [11]** | 113 | 0.67628181 | 0 | 0.061980348 | 0.019704433 | 6.64E-10 |
| **Red Hills [11]** | 114 | 0.67628181 | 0 | 0.061980348 | 0.019704433 | 6.64E-10 |
| **Red Hills [11]** | 115 | 0.67628181 | 0 | 0.061980348 | 0.019704433 | 6.64E-10 |
| **Red Hills [11]** | 116 | 0.67628181 | 0 | 0.061980348 | 0.019704433 | 6.64E-10 |
| **Red Hills [11]** | 117 | 0.67628181 | 0 | 0.061980348 | 0.019704433 | 6.64E-10 |
| **Red Hills [11]** | 118 | 0.67628181 | 0 | 0.061980348 | 0.019704433 | 6.64E-10 |
| **Red Hills [11]** | 119 | 0.67628181 | 0 | 0.061980348 | 0.019704433 | 6.64E-10 |
| **Red Hills [11]** | 120 | 0.67628181 | 0 | 0.061980348 | 0.019704433 | 6.64E-10 |
| **Red Hills [11]** | 121 | 0.67628181 | 0 | 0.061980348 | 0.019704433 | 6.64E-10 |
| **Red Hills [11]** | 122 | 0.67628181 | 0 | 0.061980348 | 0.019704433 | 6.64E-10 |
| **Red Hills [11]** | 123 | 0.67628181 | 0 | 0.061980348 | 0.019704433 | 6.64E-10 |
| **Red Hills [11]** | 124 | 0.67628181 | 0 | 0.061980348 | 0.019704433 | 6.64E-10 |
| **Red Hills [11]** | 125 | 0.67628181 | 0 | 0.061980348 | 0.019704433 | 6.64E-10 |
| **Red Hills [11]** | 126 | 0.67628181 | 0 | 0.061980348 | 0.019704433 | 6.64E-10 |
| **Red Hills [11]** | 127 | 0.67628181 | 0 | 0.061980348 | 0.019704433 | 6.64E-10 |
| **Red Hills [11]** | 128 | 0.67628181 | 0 | 0.061980348 | 0.019704433 | 6.64E-10 |
| **Red Hills [11]** | 129 | 0.67628181 | 0 | 0.061980348 | 0.019704433 | 6.64E-10 |
| **Red Hills [11]** | 130 | 0.67628181 | 0 | 0.061980348 | 0.019704433 | 6.64E-10 |
| **Red Hills [11]** | 131 | 0.67628181 | 0 | 0.061980348 | 0.019704433 | 6.64E-10 |
| **Red Hills [11]** | 132 | 0.67628181 | 0 | 0.061980348 | 0.019704433 | 6.64E-10 |
| **Red Hills [11]** | 133 | 0.67628181 | 0 | 0.061980348 | 0.019704433 | 6.64E-10 |
| **Red Hills [11]** | 134 | 0.67628181 | 0 | 0.061980348 | 0.019704433 | 6.64E-10 |
| **Red Hills [11]** | 135 | 0.67628181 | 0 | 0.061980348 | 0.019704433 | 6.64E-10 |
| **Red Hills [11]** | 136 | 0.67628181 | 0 | 0.061980348 | 0.019704433 | 6.64E-10 |
| **Red Hills [11]** | 137 | 0.67628181 | 0 | 0.061980348 | 0.019704433 | 6.64E-10 |
| **Red Hills [11]** | 138 | 0.67628181 | 0 | 0.061980348 | 0.019704433 | 6.64E-10 |
| **Red Hills [11]** | 139 | 0.67628181 | 0 | 0.061980348 | 0.019704433 | 6.64E-10 |
| **Red Hills [11]** | 140 | 0.67628181 | 0 | 0.061980348 | 0.019704433 | 6.64E-10 |
| **Red Hills [11]** | 141 | 0.67628181 | 0 | 0.061980348 | 0.019704433 | 6.64E-10 |
| **Red Hills [11]** | 142 | 0.67628181 | 0 | 0.061980348 | 0.019704433 | 6.64E-10 |
| **Red Hills [11]** | 143 | 0.67628181 | 0 | 0.061980348 | 0.019704433 | 6.64E-10 |
| **Red Hills [11]** | 144 | 0.67628181 | 0 | 0.061980348 | 0.019704433 | 6.64E-10 |
| **Rugby [12]** | 3 | 0.27470672 | 0.999835515 | 0.053665911 | 0.02955665 | 0.000435664 |
| **Rugby [12]** | 4 | 0.27470672 | 0.999835515 | 0.053665911 | 0.02955665 | 0.000435664 |
| **Rugby [12]** | 5 | 0.27470672 | 0.999835515 | 0.053665911 | 0.02955665 | 0.000435664 |
| **Rugby [12]** | 6 | 0.27470672 | 0.999835515 | 0.053665911 | 0.02955665 | 0.000435664 |
| **Rugby [12]** | 7 | 0.27470672 | 0.999835515 | 0.053665911 | 0.02955665 | 0.000435664 |
| **Rugby [12]** | 8 | 0.27470672 | 0.998448706 | 0.053665911 | 0.02955665 | 0.00043506 |
| **Rugby [12]** | 9 | 0.27470672 | 0.991704805 | 0.053665911 | 0.02955665 | 0.000432121 |
| **Rugby [12]** | 10 | 0.27470672 | 0.972691531 | 0.053665911 | 0.02955665 | 0.000423836 |
| **Rugby [12]** | 11 | 0.27470672 | 0.93559419 | 0.053665911 | 0.02955665 | 0.000407672 |
| **Rugby [12]** | 12 | 0.27470672 | 0.897812152 | 0.053665911 | 0.02955665 | 0.000391209 |
| **Rugby [12]** | 13 | 0.27470672 | 0.857220237 | 0.053665911 | 0.02955665 | 0.000373521 |
| **Rugby [12]** | 14 | 0.27470672 | 0.819356354 | 0.053665911 | 0.02955665 | 0.000357023 |
| **Rugby [12]** | 15 | 0.27470672 | 0.793180181 | 0.053665911 | 0.02955665 | 0.000345617 |
| **Rugby [12]** | 16 | 0.27470672 | 0.784079869 | 0.053665911 | 0.02955665 | 0.000341651 |
| **Rugby [12]** | 17 | 0.27470672 | 0.778788158 | 0.053665911 | 0.02955665 | 0.000339346 |
| **Rugby [12]** | 18 | 0.27470672 | 0.77492069 | 0.053665911 | 0.02955665 | 0.000337661 |
| **Rugby [12]** | 19 | 0.27470672 | 0.77190793 | 0.053665911 | 0.02955665 | 0.000336348 |
| **Rugby [12]** | 20 | 0.27470672 | 0.768475052 | 0.053665911 | 0.02955665 | 0.000334852 |
| **Rugby [12]** | 21 | 0.27470672 | 0.757740984 | 0.053665911 | 0.02955665 | 0.000330175 |
| **Rugby [12]** | 22 | 0.27470672 | 0.749708797 | 0.053665911 | 0.02955665 | 0.000326675 |
| **Rugby [12]** | 23 | 0.27470672 | 0.744550506 | 0.053665911 | 0.02955665 | 0.000324427 |
| **Rugby [12]** | 24 | 0.27470672 | 0.74187162 | 0.053665911 | 0.02955665 | 0.00032326 |
| **Rugby [12]** | 25 | 0.27470672 | 0.742577274 | 0.053665911 | 0.02955665 | 0.000323567 |
| **Rugby [12]** | 26 | 0.27470672 | 0.749254493 | 0.053665911 | 0.02955665 | 0.000326477 |
| **Rugby [12]** | 27 | 0.27470672 | 0.755031783 | 0.053665911 | 0.02955665 | 0.000328994 |
| **Rugby [12]** | 28 | 0.27470672 | 0.757806926 | 0.053665911 | 0.02955665 | 0.000330203 |
| **Rugby [12]** | 29 | 0.27470672 | 0.755616943 | 0.053665911 | 0.02955665 | 0.000329249 |
| **Rugby [12]** | 30 | 0.27470672 | 0.752716985 | 0.053665911 | 0.02955665 | 0.000327986 |
| **Rugby [12]** | 31 | 0.27470672 | 0.742880603 | 0.053665911 | 0.02955665 | 0.000323699 |
| **Rugby [12]** | 32 | 0.27470672 | 0.736300034 | 0.053665911 | 0.02955665 | 0.000320832 |
| **Rugby [12]** | 33 | 0.27470672 | 0.732263145 | 0.053665911 | 0.02955665 | 0.000319073 |
| **Rugby [12]** | 34 | 0.27470672 | 0.729736296 | 0.053665911 | 0.02955665 | 0.000317972 |
| **Rugby [12]** | 35 | 0.27470672 | 0.727638688 | 0.053665911 | 0.02955665 | 0.000317058 |
| **Rugby [12]** | 36 | 0.27470672 | 0.725859459 | 0.053665911 | 0.02955665 | 0.000316283 |
| **Rugby [12]** | 37 | 0.27470672 | 0.724178874 | 0.053665911 | 0.02955665 | 0.00031555 |
| **Rugby [12]** | 38 | 0.27470672 | 0.721747059 | 0.053665911 | 0.02955665 | 0.000314491 |
| **Rugby [12]** | 39 | 0.27470672 | 0.71904904 | 0.053665911 | 0.02955665 | 0.000313315 |
| **Rugby [12]** | 40 | 0.27470672 | 0.718125343 | 0.053665911 | 0.02955665 | 0.000312913 |
| **Rugby [12]** | 41 | 0.27470672 | 0.722984847 | 0.053665911 | 0.02955665 | 0.00031503 |
| **Rugby [12]** | 42 | 0.27470672 | 0.725762316 | 0.053665911 | 0.02955665 | 0.00031624 |
| **Rugby [12]** | 43 | 0.27470672 | 0.727353163 | 0.053665911 | 0.02955665 | 0.000316934 |
| **Rugby [12]** | 44 | 0.27470672 | 0.728175015 | 0.053665911 | 0.02955665 | 0.000317292 |
| **Rugby [12]** | 45 | 0.27470672 | 0.727340563 | 0.053665911 | 0.02955665 | 0.000316928 |
| **Rugby [12]** | 46 | 0.27470672 | 0.721181732 | 0.053665911 | 0.02955665 | 0.000314245 |
| **Rugby [12]** | 47 | 0.27470672 | 0.716121072 | 0.053665911 | 0.02955665 | 0.000312039 |
| **Rugby [12]** | 48 | 0.27470672 | 0.712283416 | 0.053665911 | 0.02955665 | 0.000310367 |
| **Rugby [12]** | 49 | 0.27470672 | 0.709088835 | 0.053665911 | 0.02955665 | 0.000308975 |
| **Rugby [12]** | 50 | 0.27470672 | 0.707746486 | 0.053665911 | 0.02955665 | 0.00030839 |
| **Rugby [12]** | 51 | 0.27470672 | 0.710729211 | 0.053665911 | 0.02955665 | 0.00030969 |
| **Rugby [12]** | 52 | 0.27470672 | 0.713329919 | 0.053665911 | 0.02955665 | 0.000310823 |
| **Rugby [12]** | 53 | 0.27470672 | 0.708779716 | 0.053665911 | 0.02955665 | 0.000308841 |
| **Rugby [12]** | 54 | 0.27470672 | 0.703687655 | 0.053665911 | 0.02955665 | 0.000306622 |
| **Rugby [12]** | 55 | 0.27470672 | 0.699762471 | 0.053665911 | 0.02955665 | 0.000304911 |
| **Rugby [12]** | 56 | 0.27470672 | 0.693544664 | 0.053665911 | 0.02955665 | 0.000302202 |
| **Rugby [12]** | 57 | 0.27470672 | 0.687794519 | 0.053665911 | 0.02955665 | 0.000299697 |
| **Rugby [12]** | 58 | 0.27470672 | 0.683263212 | 0.053665911 | 0.02955665 | 0.000297722 |
| **Rugby [12]** | 59 | 0.27470672 | 0.67929807 | 0.053665911 | 0.02955665 | 0.000295994 |
| **Rugby [12]** | 60 | 0.27470672 | 0.675905073 | 0.053665911 | 0.02955665 | 0.000294516 |
| **Rugby [12]** | 61 | 0.27470672 | 0.673007238 | 0.053665911 | 0.02955665 | 0.000293253 |
| **Rugby [12]** | 62 | 0.27470672 | 0.670481813 | 0.053665911 | 0.02955665 | 0.000292153 |
| **Rugby [12]** | 63 | 0.27470672 | 0.668270911 | 0.053665911 | 0.02955665 | 0.000291189 |
| **Rugby [12]** | 64 | 0.27470672 | 0.666189556 | 0.053665911 | 0.02955665 | 0.000290282 |
| **Rugby [12]** | 65 | 0.27470672 | 0.665148061 | 0.053665911 | 0.02955665 | 0.000289829 |
| **Rugby [12]** | 66 | 0.27470672 | 0.668612026 | 0.053665911 | 0.02955665 | 0.000291338 |
| **Rugby [12]** | 67 | 0.27470672 | 0.670795208 | 0.053665911 | 0.02955665 | 0.000292289 |
| **Rugby [12]** | 68 | 0.27470672 | 0.673023028 | 0.053665911 | 0.02955665 | 0.00029326 |
| **Rugby [12]** | 69 | 0.27470672 | 0.67490322 | 0.053665911 | 0.02955665 | 0.000294079 |
| **Rugby [12]** | 70 | 0.27470672 | 0.675292619 | 0.053665911 | 0.02955665 | 0.000294249 |
| **Rugby [12]** | 71 | 0.27470672 | 0.670609324 | 0.053665911 | 0.02955665 | 0.000292208 |
| **Rugby [12]** | 72 | 0.27470672 | 0.667039077 | 0.053665911 | 0.02955665 | 0.000290653 |
| **Rugby [12]** | 73 | 0.27470672 | 0.664029636 | 0.053665911 | 0.02955665 | 0.000289341 |
| **Rugby [12]** | 74 | 0.27470672 | 0.68382547 | 0.053665911 | 0.02955665 | 0.000297967 |
| **Rugby [12]** | 75 | 0.27470672 | 0.684290529 | 0.053665911 | 0.02955665 | 0.00029817 |
| **Rugby [12]** | 76 | 0.27470672 | 0.688273459 | 0.053665911 | 0.02955665 | 0.000299905 |
| **Rugby [12]** | 77 | 0.27470672 | 0.690686569 | 0.053665911 | 0.02955665 | 0.000300957 |
| **Rugby [12]** | 78 | 0.27470672 | 0.691253969 | 0.053665911 | 0.02955665 | 0.000301204 |
| **Rugby [12]** | 79 | 0.27470672 | 0.691317361 | 0.053665911 | 0.02955665 | 0.000301232 |
| **Rugby [12]** | 80 | 0.27470672 | 0.689713154 | 0.053665911 | 0.02955665 | 0.000300533 |
| **Rugby [12]** | 81 | 0.27470672 | 0.684810459 | 0.053665911 | 0.02955665 | 0.000298396 |
| **Rugby [12]** | 82 | 0.27470672 | 0.680120412 | 0.053665911 | 0.02955665 | 0.000296353 |
| **Rugby [12]** | 83 | 0.27470672 | 0.676774606 | 0.053665911 | 0.02955665 | 0.000294895 |
| **Rugby [12]** | 84 | 0.27470672 | 0.674536996 | 0.053665911 | 0.02955665 | 0.00029392 |
| **Rugby [12]** | 85 | 0.27470672 | 0.672801753 | 0.053665911 | 0.02955665 | 0.000293164 |
| **Rugby [12]** | 86 | 0.27470672 | 0.671178387 | 0.053665911 | 0.02955665 | 0.000292456 |
| **Rugby [12]** | 87 | 0.27470672 | 0.669596054 | 0.053665911 | 0.02955665 | 0.000291767 |
| **Rugby [12]** | 88 | 0.27470672 | 0.667023323 | 0.053665911 | 0.02955665 | 0.000290646 |
| **Rugby [12]** | 89 | 0.27470672 | 0.665032964 | 0.053665911 | 0.02955665 | 0.000289779 |
| **Rugby [12]** | 90 | 0.27470672 | 0.66430831 | 0.053665911 | 0.02955665 | 0.000289463 |
| **Rugby [12]** | 91 | 0.27470672 | 0.66780877 | 0.053665911 | 0.02955665 | 0.000290988 |
| **Rugby [12]** | 92 | 0.27470672 | 0.670546166 | 0.053665911 | 0.02955665 | 0.000292181 |
| **Rugby [12]** | 93 | 0.27470672 | 0.672090449 | 0.053665911 | 0.02955665 | 0.000292854 |
| **Rugby [12]** | 94 | 0.27470672 | 0.67224182 | 0.053665911 | 0.02955665 | 0.00029292 |
| **Rugby [12]** | 95 | 0.27470672 | 0.671775468 | 0.053665911 | 0.02955665 | 0.000292716 |
| **Rugby [12]** | 96 | 0.27470672 | 0.667276376 | 0.053665911 | 0.02955665 | 0.000290756 |
| **Rugby [12]** | 97 | 0.27470672 | 0.663679248 | 0.053665911 | 0.02955665 | 0.000289189 |
| **Rugby [12]** | 98 | 0.27470672 | 0.660999272 | 0.053665911 | 0.02955665 | 0.000288021 |
| **Rugby [12]** | 99 | 0.27470672 | 0.659052571 | 0.053665911 | 0.02955665 | 0.000287173 |
| **Rugby [12]** | 100 | 0.27470672 | 0.658957894 | 0.053665911 | 0.02955665 | 0.000287131 |
| **Rugby [12]** | 101 | 0.27470672 | 0.661753554 | 0.053665911 | 0.02955665 | 0.00028835 |
| **Rugby [12]** | 102 | 0.27470672 | 0.661903678 | 0.053665911 | 0.02955665 | 0.000288415 |
| **Rugby [12]** | 103 | 0.27470672 | 0.582245118 | 0.053665911 | 0.02955665 | 0.000253705 |
| **Rugby [12]** | 104 | 0.27470672 | 0.5200324 | 0.053665911 | 0.02955665 | 0.000226597 |
| **Rugby [12]** | 105 | 0.27470672 | 0.47928092 | 0.053665911 | 0.02955665 | 0.00020884 |
| **Rugby [12]** | 106 | 0.27470672 | 0.442521939 | 0.053665911 | 0.02955665 | 0.000192823 |
| **Rugby [12]** | 107 | 0.27470672 | 0.413516406 | 0.053665911 | 0.02955665 | 0.000180184 |
| **Rugby [12]** | 108 | 0.27470672 | 0.388918642 | 0.053665911 | 0.02955665 | 0.000169466 |
| **Rugby [12]** | 109 | 0.27470672 | 0.36659483 | 0.053665911 | 0.02955665 | 0.000159738 |
| **Rugby [12]** | 110 | 0.27470672 | 0.345720446 | 0.053665911 | 0.02955665 | 0.000150643 |
| **Rugby [12]** | 111 | 0.27470672 | 0.326139935 | 0.053665911 | 0.02955665 | 0.000142111 |
| **Rugby [12]** | 112 | 0.27470672 | 0.30854434 | 0.053665911 | 0.02955665 | 0.000134444 |
| **Rugby [12]** | 113 | 0.27470672 | 0.29133916 | 0.053665911 | 0.02955665 | 0.000126947 |
| **Rugby [12]** | 114 | 0.27470672 | 0.274732803 | 0.053665911 | 0.02955665 | 0.000119711 |
| **Rugby [12]** | 115 | 0.27470672 | 0.259503189 | 0.053665911 | 0.02955665 | 0.000113075 |
| **Rugby [12]** | 116 | 0.27470672 | 0.245849196 | 0.053665911 | 0.02955665 | 0.000107125 |
| **Rugby [12]** | 117 | 0.27470672 | 0.23280284 | 0.053665911 | 0.02955665 | 0.00010144 |
| **Rugby [12]** | 118 | 0.27470672 | 0.219530525 | 0.053665911 | 0.02955665 | 9.57E-05 |
| **Rugby [12]** | 119 | 0.27470672 | 0.206678912 | 0.053665911 | 0.02955665 | 9.01E-05 |
| **Rugby [12]** | 120 | 0.27470672 | 0.194510862 | 0.053665911 | 0.02955665 | 8.48E-05 |
| **Rugby [12]** | 121 | 0.27470672 | 0.182649859 | 0.053665911 | 0.02955665 | 7.96E-05 |
| **Rugby [12]** | 122 | 0.27470672 | 0.170996601 | 0.053665911 | 0.02955665 | 7.45E-05 |
| **Rugby [12]** | 123 | 0.27470672 | 0.16004641 | 0.053665911 | 0.02955665 | 6.97E-05 |
| **Rugby [12]** | 124 | 0.27470672 | 0.150052026 | 0.053665911 | 0.02955665 | 6.54E-05 |
| **Rugby [12]** | 125 | 0.27470672 | 0.140593791 | 0.053665911 | 0.02955665 | 6.13E-05 |
| **Rugby [12]** | 126 | 0.27470672 | 0.131384628 | 0.053665911 | 0.02955665 | 5.72E-05 |
| **Rugby [12]** | 127 | 0.27470672 | 0.121370423 | 0.053665911 | 0.02955665 | 5.29E-05 |
| **Rugby [12]** | 128 | 0.27470672 | 0.112098664 | 0.053665911 | 0.02955665 | 4.88E-05 |
| **Rugby [12]** | 129 | 0.27470672 | 0.103365698 | 0.053665911 | 0.02955665 | 4.50E-05 |
| **Rugby [12]** | 130 | 0.27470672 | 0.09542775 | 0.053665911 | 0.02955665 | 4.16E-05 |
| **Rugby [12]** | 131 | 0.27470672 | 0 | 0.053665911 | 0.02955665 | 6.64E-10 |
| **Rugby [12]** | 132 | 0.27470672 | 0 | 0.053665911 | 0.02955665 | 6.64E-10 |
| **Rugby [12]** | 133 | 0.27470672 | 0 | 0.053665911 | 0.02955665 | 6.64E-10 |
| **Rugby [12]** | 134 | 0.27470672 | 0 | 0.053665911 | 0.02955665 | 6.64E-10 |
| **Rugby [12]** | 135 | 0.27470672 | 0 | 0.053665911 | 0.02955665 | 6.64E-10 |
| **Rugby [12]** | 136 | 0.27470672 | 0 | 0.053665911 | 0.02955665 | 6.64E-10 |
| **Rugby [12]** | 137 | 0.27470672 | 0 | 0.053665911 | 0.02955665 | 6.64E-10 |
| **Rugby [12]** | 138 | 0.27470672 | 0 | 0.053665911 | 0.02955665 | 6.64E-10 |
| **Rugby [12]** | 139 | 0.27470672 | 0 | 0.053665911 | 0.02955665 | 6.64E-10 |
| **Rugby [12]** | 140 | 0.27470672 | 0 | 0.053665911 | 0.02955665 | 6.64E-10 |
| **Rugby [12]** | 141 | 0.27470672 | 0 | 0.053665911 | 0.02955665 | 6.64E-10 |
| **Rugby [12]** | 142 | 0.27470672 | 0 | 0.053665911 | 0.02955665 | 6.64E-10 |
| **Rugby [12]** | 143 | 0.27470672 | 0 | 0.053665911 | 0.02955665 | 6.64E-10 |
| **Rugby [12]** | 144 | 0.27470672 | 0 | 0.053665911 | 0.02955665 | 6.64E-10 |
| **Anonymous Wind Facility, CO** | 3 | 0.59207658 | 0.999835515 | 0.037792895 | 0.118226601 | 0.002645037 |
| **Anonymous Wind Facility, CO** | 4 | 0.59207658 | 0.999835515 | 0.037792895 | 0.118226601 | 0.002645037 |
| **Anonymous Wind Facility, CO** | 5 | 0.59207658 | 0.999835515 | 0.037792895 | 0.118226601 | 0.002645037 |
| **Anonymous Wind Facility, CO** | 6 | 0.59207658 | 0.999835515 | 0.037792895 | 0.118226601 | 0.002645037 |
| **Anonymous Wind Facility, CO** | 7 | 0.59207658 | 0.999835515 | 0.037792895 | 0.118226601 | 0.002645037 |
| **Anonymous Wind Facility, CO** | 8 | 0.59207658 | 0.999835515 | 0.037792895 | 0.118226601 | 0.002645037 |
| **Anonymous Wind Facility, CO** | 9 | 0.59207658 | 0.999835515 | 0.037792895 | 0.118226601 | 0.002645037 |
| **Anonymous Wind Facility, CO** | 10 | 0.59207658 | 0.999835515 | 0.037792895 | 0.118226601 | 0.002645037 |
| **Anonymous Wind Facility, CO** | 11 | 0.59207658 | 0.999835515 | 0.037792895 | 0.118226601 | 0.002645037 |
| **Anonymous Wind Facility, CO** | 12 | 0.59207658 | 0.999835515 | 0.037792895 | 0.118226601 | 0.002645037 |
| **Anonymous Wind Facility, CO** | 13 | 0.59207658 | 0.999835515 | 0.037792895 | 0.118226601 | 0.002645037 |
| **Anonymous Wind Facility, CO** | 14 | 0.59207658 | 0.999835515 | 0.037792895 | 0.118226601 | 0.002645037 |
| **Anonymous Wind Facility, CO** | 15 | 0.59207658 | 0.999835515 | 0.037792895 | 0.118226601 | 0.002645037 |
| **Anonymous Wind Facility, CO** | 16 | 0.59207658 | 0.999835515 | 0.037792895 | 0.118226601 | 0.002645037 |
| **Anonymous Wind Facility, CO** | 17 | 0.59207658 | 0.999835515 | 0.037792895 | 0.118226601 | 0.002645037 |
| **Anonymous Wind Facility, CO** | 18 | 0.59207658 | 0.999835515 | 0.037792895 | 0.118226601 | 0.002645037 |
| **Anonymous Wind Facility, CO** | 19 | 0.59207658 | 0.999835515 | 0.037792895 | 0.118226601 | 0.002645037 |
| **Anonymous Wind Facility, CO** | 20 | 0.59207658 | 0.999835515 | 0.037792895 | 0.118226601 | 0.002645037 |
| **Anonymous Wind Facility, CO** | 21 | 0.59207658 | 0.999835515 | 0.037792895 | 0.118226601 | 0.002645037 |
| **Anonymous Wind Facility, CO** | 22 | 0.59207658 | 0.999835515 | 0.037792895 | 0.118226601 | 0.002645037 |
| **Anonymous Wind Facility, CO** | 23 | 0.59207658 | 0.999835515 | 0.037792895 | 0.118226601 | 0.002645037 |
| **Anonymous Wind Facility, CO** | 24 | 0.59207658 | 0.999835515 | 0.037792895 | 0.118226601 | 0.002645037 |
| **Anonymous Wind Facility, CO** | 25 | 0.59207658 | 0.999835515 | 0.037792895 | 0.118226601 | 0.002645037 |
| **Anonymous Wind Facility, CO** | 26 | 0.59207658 | 0.999835515 | 0.037792895 | 0.118226601 | 0.002645037 |
| **Anonymous Wind Facility, CO** | 27 | 0.59207658 | 0.999835515 | 0.037792895 | 0.118226601 | 0.002645037 |
| **Anonymous Wind Facility, CO** | 28 | 0.59207658 | 0.999835515 | 0.037792895 | 0.118226601 | 0.002645037 |
| **Anonymous Wind Facility, CO** | 29 | 0.59207658 | 0.999835515 | 0.037792895 | 0.118226601 | 0.002645037 |
| **Anonymous Wind Facility, CO** | 30 | 0.59207658 | 0.999835515 | 0.037792895 | 0.118226601 | 0.002645037 |
| **Anonymous Wind Facility, CO** | 31 | 0.59207658 | 0.999835515 | 0.037792895 | 0.118226601 | 0.002645037 |
| **Anonymous Wind Facility, CO** | 32 | 0.59207658 | 0.999835515 | 0.037792895 | 0.118226601 | 0.002645037 |
| **Anonymous Wind Facility, CO** | 33 | 0.59207658 | 0.999835515 | 0.037792895 | 0.118226601 | 0.002645037 |
| **Anonymous Wind Facility, CO** | 34 | 0.59207658 | 0.999835515 | 0.037792895 | 0.118226601 | 0.002645037 |
| **Anonymous Wind Facility, CO** | 35 | 0.59207658 | 0.999835515 | 0.037792895 | 0.118226601 | 0.002645037 |
| **Anonymous Wind Facility, CO** | 36 | 0.59207658 | 0.999835515 | 0.037792895 | 0.118226601 | 0.002645037 |
| **Anonymous Wind Facility, CO** | 37 | 0.59207658 | 0.999835515 | 0.037792895 | 0.118226601 | 0.002645037 |
| **Anonymous Wind Facility, CO** | 38 | 0.59207658 | 0.999835515 | 0.037792895 | 0.118226601 | 0.002645037 |
| **Anonymous Wind Facility, CO** | 39 | 0.59207658 | 0.999835515 | 0.037792895 | 0.118226601 | 0.002645037 |
| **Anonymous Wind Facility, CO** | 40 | 0.59207658 | 0.999835515 | 0.037792895 | 0.118226601 | 0.002645037 |
| **Anonymous Wind Facility, CO** | 41 | 0.59207658 | 0.999835515 | 0.037792895 | 0.118226601 | 0.002645037 |
| **Anonymous Wind Facility, CO** | 42 | 0.59207658 | 0.999835515 | 0.037792895 | 0.118226601 | 0.002645037 |
| **Anonymous Wind Facility, CO** | 43 | 0.59207658 | 0.999835515 | 0.037792895 | 0.118226601 | 0.002645037 |
| **Anonymous Wind Facility, CO** | 44 | 0.59207658 | 0.999835515 | 0.037792895 | 0.118226601 | 0.002645037 |
| **Anonymous Wind Facility, CO** | 45 | 0.59207658 | 0.999835515 | 0.037792895 | 0.118226601 | 0.002645037 |
| **Anonymous Wind Facility, CO** | 46 | 0.59207658 | 0.999835515 | 0.037792895 | 0.118226601 | 0.002645037 |
| **Anonymous Wind Facility, CO** | 47 | 0.59207658 | 0.999835515 | 0.037792895 | 0.118226601 | 0.002645037 |
| **Anonymous Wind Facility, CO** | 48 | 0.59207658 | 0.999835515 | 0.037792895 | 0.118226601 | 0.002645037 |
| **Anonymous Wind Facility, CO** | 49 | 0.59207658 | 0.999835515 | 0.037792895 | 0.118226601 | 0.002645037 |
| **Anonymous Wind Facility, CO** | 50 | 0.59207658 | 0.999835515 | 0.037792895 | 0.118226601 | 0.002645037 |
| **Anonymous Wind Facility, CO** | 51 | 0.59207658 | 0.999835515 | 0.037792895 | 0.118226601 | 0.002645037 |
| **Anonymous Wind Facility, CO** | 52 | 0.59207658 | 0.999835515 | 0.037792895 | 0.118226601 | 0.002645037 |
| **Anonymous Wind Facility, CO** | 53 | 0.59207658 | 0.999835515 | 0.037792895 | 0.118226601 | 0.002645037 |
| **Anonymous Wind Facility, CO** | 54 | 0.59207658 | 0.999835515 | 0.037792895 | 0.118226601 | 0.002645037 |
| **Anonymous Wind Facility, CO** | 55 | 0.59207658 | 0.999835515 | 0.037792895 | 0.118226601 | 0.002645037 |
| **Anonymous Wind Facility, CO** | 56 | 0.59207658 | 0.999835515 | 0.037792895 | 0.118226601 | 0.002645037 |
| **Anonymous Wind Facility, CO** | 57 | 0.59207658 | 0.999835515 | 0.037792895 | 0.118226601 | 0.002645037 |
| **Anonymous Wind Facility, CO** | 58 | 0.59207658 | 0.999835515 | 0.037792895 | 0.118226601 | 0.002645037 |
| **Anonymous Wind Facility, CO** | 59 | 0.59207658 | 0.999835515 | 0.037792895 | 0.118226601 | 0.002645037 |
| **Anonymous Wind Facility, CO** | 60 | 0.59207658 | 0.999835515 | 0.037792895 | 0.118226601 | 0.002645037 |
| **Anonymous Wind Facility, CO** | 61 | 0.59207658 | 0.999835515 | 0.037792895 | 0.118226601 | 0.002645037 |
| **Anonymous Wind Facility, CO** | 62 | 0.59207658 | 0.999835515 | 0.037792895 | 0.118226601 | 0.002645037 |
| **Anonymous Wind Facility, CO** | 63 | 0.59207658 | 0.999835515 | 0.037792895 | 0.118226601 | 0.002645037 |
| **Anonymous Wind Facility, CO** | 64 | 0.59207658 | 0.999835515 | 0.037792895 | 0.118226601 | 0.002645037 |
| **Anonymous Wind Facility, CO** | 65 | 0.59207658 | 0.999835515 | 0.037792895 | 0.118226601 | 0.002645037 |
| **Anonymous Wind Facility, CO** | 66 | 0.59207658 | 0.999835515 | 0.037792895 | 0.118226601 | 0.002645037 |
| **Anonymous Wind Facility, CO** | 67 | 0.59207658 | 0.999835515 | 0.037792895 | 0.118226601 | 0.002645037 |
| **Anonymous Wind Facility, CO** | 68 | 0.59207658 | 0.999835515 | 0.037792895 | 0.118226601 | 0.002645037 |
| **Anonymous Wind Facility, CO** | 69 | 0.59207658 | 0.999835515 | 0.037792895 | 0.118226601 | 0.002645037 |
| **Anonymous Wind Facility, CO** | 70 | 0.59207658 | 0.999835515 | 0.037792895 | 0.118226601 | 0.002645037 |
| **Anonymous Wind Facility, CO** | 71 | 0.59207658 | 0.999835515 | 0.037792895 | 0.118226601 | 0.002645037 |
| **Anonymous Wind Facility, CO** | 72 | 0.59207658 | 0.999835515 | 0.037792895 | 0.118226601 | 0.002645037 |
| **Anonymous Wind Facility, CO** | 73 | 0.59207658 | 0.999835515 | 0.037792895 | 0.118226601 | 0.002645037 |
| **Anonymous Wind Facility, CO** | 74 | 0.59207658 | 0.999835515 | 0.037792895 | 0.118226601 | 0.002645037 |
| **Anonymous Wind Facility, CO** | 75 | 0.59207658 | 0.999835515 | 0.037792895 | 0.118226601 | 0.002645037 |
| **Anonymous Wind Facility, CO** | 76 | 0.59207658 | 0.999835515 | 0.037792895 | 0.118226601 | 0.002645037 |
| **Anonymous Wind Facility, CO** | 77 | 0.59207658 | 0.999835515 | 0.037792895 | 0.118226601 | 0.002645037 |
| **Anonymous Wind Facility, CO** | 78 | 0.59207658 | 0.999835515 | 0.037792895 | 0.118226601 | 0.002645037 |
| **Anonymous Wind Facility, CO** | 79 | 0.59207658 | 0.999835515 | 0.037792895 | 0.118226601 | 0.002645037 |
| **Anonymous Wind Facility, CO** | 80 | 0.59207658 | 0.999835515 | 0.037792895 | 0.118226601 | 0.002645037 |
| **Anonymous Wind Facility, CO** | 81 | 0.59207658 | 0.999835515 | 0.037792895 | 0.118226601 | 0.002645037 |
| **Anonymous Wind Facility, CO** | 82 | 0.59207658 | 0.999835515 | 0.037792895 | 0.118226601 | 0.002645037 |
| **Anonymous Wind Facility, CO** | 83 | 0.59207658 | 0.999835515 | 0.037792895 | 0.118226601 | 0.002645037 |
| **Anonymous Wind Facility, CO** | 84 | 0.59207658 | 0.999835515 | 0.037792895 | 0.118226601 | 0.002645037 |
| **Anonymous Wind Facility, CO** | 85 | 0.59207658 | 0.999835515 | 0.037792895 | 0.118226601 | 0.002645037 |
| **Anonymous Wind Facility, CO** | 86 | 0.59207658 | 0.999835515 | 0.037792895 | 0.118226601 | 0.002645037 |
| **Anonymous Wind Facility, CO** | 87 | 0.59207658 | 0.999835515 | 0.037792895 | 0.118226601 | 0.002645037 |
| **Anonymous Wind Facility, CO** | 88 | 0.59207658 | 0.999835515 | 0.037792895 | 0.118226601 | 0.002645037 |
| **Anonymous Wind Facility, CO** | 89 | 0.59207658 | 0.999835515 | 0.037792895 | 0.118226601 | 0.002645037 |
| **Anonymous Wind Facility, CO** | 90 | 0.59207658 | 0.999835515 | 0.037792895 | 0.118226601 | 0.002645037 |
| **Anonymous Wind Facility, CO** | 91 | 0.59207658 | 0.999835515 | 0.037792895 | 0.118226601 | 0.002645037 |
| **Anonymous Wind Facility, CO** | 92 | 0.59207658 | 0.999835515 | 0.037792895 | 0.118226601 | 0.002645037 |
| **Anonymous Wind Facility, CO** | 93 | 0.59207658 | 0.874955358 | 0.037792895 | 0.118226601 | 0.00231467 |
| **Anonymous Wind Facility, CO** | 94 | 0.59207658 | 0.770634852 | 0.037792895 | 0.118226601 | 0.002038693 |
| **Anonymous Wind Facility, CO** | 95 | 0.59207658 | 0.70406163 | 0.037792895 | 0.118226601 | 0.001862576 |
| **Anonymous Wind Facility, CO** | 96 | 0.59207658 | 0.651059214 | 0.037792895 | 0.118226601 | 0.001722359 |
| **Anonymous Wind Facility, CO** | 97 | 0.59207658 | 0.605908345 | 0.037792895 | 0.118226601 | 0.001602914 |
| **Anonymous Wind Facility, CO** | 98 | 0.59207658 | 0.566104433 | 0.037792895 | 0.118226601 | 0.001497614 |
| **Anonymous Wind Facility, CO** | 99 | 0.59207658 | 0.530291046 | 0.037792895 | 0.118226601 | 0.00140287 |
| **Anonymous Wind Facility, CO** | 100 | 0.59207658 | 0.497626866 | 0.037792895 | 0.118226601 | 0.001316458 |
| **Anonymous Wind Facility, CO** | 101 | 0.59207658 | 0.467399366 | 0.037792895 | 0.118226601 | 0.001236492 |
| **Anonymous Wind Facility, CO** | 102 | 0.59207658 | 0.439244452 | 0.037792895 | 0.118226601 | 0.001162009 |
| **Anonymous Wind Facility, CO** | 103 | 0.59207658 | 0.413002074 | 0.037792895 | 0.118226601 | 0.001092586 |
| **Anonymous Wind Facility, CO** | 104 | 0.59207658 | 0.388216302 | 0.037792895 | 0.118226601 | 0.001027016 |
| **Anonymous Wind Facility, CO** | 105 | 0.59207658 | 0.36473717 | 0.037792895 | 0.118226601 | 0.000964902 |
| **Anonymous Wind Facility, CO** | 106 | 0.59207658 | 0.342618578 | 0.037792895 | 0.118226601 | 0.000906388 |
| **Anonymous Wind Facility, CO** | 107 | 0.59207658 | 0.321342803 | 0.037792895 | 0.118226601 | 0.000850104 |
| **Anonymous Wind Facility, CO** | 108 | 0.59207658 | 0.301280006 | 0.037792895 | 0.118226601 | 0.000797028 |
| **Anonymous Wind Facility, CO** | 109 | 0.59207658 | 0.281840104 | 0.037792895 | 0.118226601 | 0.0007456 |
| **Anonymous Wind Facility, CO** | 110 | 0.59207658 | 0.263447172 | 0.037792895 | 0.118226601 | 0.000696942 |
| **Anonymous Wind Facility, CO** | 111 | 0.59207658 | 0.245568146 | 0.037792895 | 0.118226601 | 0.000649644 |
| **Anonymous Wind Facility, CO** | 112 | 0.59207658 | 0.228529972 | 0.037792895 | 0.118226601 | 0.00060457 |
| **Anonymous Wind Facility, CO** | 113 | 0.59207658 | 0.212061594 | 0.037792895 | 0.118226601 | 0.000561003 |
| **Anonymous Wind Facility, CO** | 114 | 0.59207658 | 0.196081844 | 0.037792895 | 0.118226601 | 0.000518729 |
| **Anonymous Wind Facility, CO** | 115 | 0.59207658 | 0.180848198 | 0.037792895 | 0.118226601 | 0.000478429 |
| **Anonymous Wind Facility, CO** | 116 | 0.59207658 | 0.165906049 | 0.037792895 | 0.118226601 | 0.0004389 |
| **Anonymous Wind Facility, CO** | 117 | 0.59207658 | 0 | 0.037792895 | 0.118226601 | 6.64E-10 |
| **Anonymous Wind Facility, CO** | 118 | 0.59207658 | 0 | 0.037792895 | 0.118226601 | 6.64E-10 |
| **Anonymous Wind Facility, CO** | 119 | 0.59207658 | 0 | 0.037792895 | 0.118226601 | 6.64E-10 |
| **Anonymous Wind Facility, CO** | 120 | 0.59207658 | 0 | 0.037792895 | 0.118226601 | 6.64E-10 |
| **Anonymous Wind Facility, CO** | 121 | 0.59207658 | 0 | 0.037792895 | 0.118226601 | 6.64E-10 |
| **Anonymous Wind Facility, CO** | 122 | 0.59207658 | 0 | 0.037792895 | 0.118226601 | 6.64E-10 |
| **Anonymous Wind Facility, CO** | 123 | 0.59207658 | 0 | 0.037792895 | 0.118226601 | 6.64E-10 |
| **Anonymous Wind Facility, CO** | 124 | 0.59207658 | 0 | 0.037792895 | 0.118226601 | 6.64E-10 |
| **Anonymous Wind Facility, CO** | 125 | 0.59207658 | 0 | 0.037792895 | 0.118226601 | 6.64E-10 |
| **Anonymous Wind Facility, CO** | 126 | 0.59207658 | 0 | 0.037792895 | 0.118226601 | 6.64E-10 |
| **Anonymous Wind Facility, CO** | 127 | 0.59207658 | 0 | 0.037792895 | 0.118226601 | 6.64E-10 |
| **Anonymous Wind Facility, CO** | 128 | 0.59207658 | 0 | 0.037792895 | 0.118226601 | 6.64E-10 |
| **Anonymous Wind Facility, CO** | 129 | 0.59207658 | 0 | 0.037792895 | 0.118226601 | 6.64E-10 |
| **Anonymous Wind Facility, CO** | 130 | 0.59207658 | 0 | 0.037792895 | 0.118226601 | 6.64E-10 |
| **Anonymous Wind Facility, CO** | 131 | 0.59207658 | 0 | 0.037792895 | 0.118226601 | 6.64E-10 |
| **Anonymous Wind Facility, CO** | 132 | 0.59207658 | 0 | 0.037792895 | 0.118226601 | 6.64E-10 |
| **Anonymous Wind Facility, CO** | 133 | 0.59207658 | 0 | 0.037792895 | 0.118226601 | 6.64E-10 |
| **Anonymous Wind Facility, CO** | 134 | 0.59207658 | 0 | 0.037792895 | 0.118226601 | 6.64E-10 |
| **Anonymous Wind Facility, CO** | 135 | 0.59207658 | 0 | 0.037792895 | 0.118226601 | 6.64E-10 |
| **Anonymous Wind Facility, CO** | 136 | 0.59207658 | 0 | 0.037792895 | 0.118226601 | 6.64E-10 |
| **Anonymous Wind Facility, CO** | 137 | 0.59207658 | 0 | 0.037792895 | 0.118226601 | 6.64E-10 |
| **Anonymous Wind Facility, CO** | 138 | 0.59207658 | 0 | 0.037792895 | 0.118226601 | 6.64E-10 |
| **Anonymous Wind Facility, CO** | 139 | 0.59207658 | 0 | 0.037792895 | 0.118226601 | 6.64E-10 |
| **Anonymous Wind Facility, CO** | 140 | 0.59207658 | 0 | 0.037792895 | 0.118226601 | 6.64E-10 |
| **Anonymous Wind Facility, CO** | 141 | 0.59207658 | 0 | 0.037792895 | 0.118226601 | 6.64E-10 |
| **Anonymous Wind Facility, CO** | 142 | 0.59207658 | 0 | 0.037792895 | 0.118226601 | 6.64E-10 |
| **Anonymous Wind Facility, CO** | 143 | 0.59207658 | 0 | 0.037792895 | 0.118226601 | 6.64E-10 |
| **Anonymous Wind Facility, CO** | 144 | 0.59207658 | 0 | 0.037792895 | 0.118226601 | 6.64E-10 |

^a^ All weights for one to two m from turbines are 0.0 to account for the radius of the turbine base. All weights from 145 to 175 m from turbines are 6.64E -10, or 1/1000^th^ of the smallest observed weight. Weights associated with numbers of turbines and fatality rates (large birds/turbine) are scaled so that their sums across studies are 1.0.

### References

1. Chatfield A, Bay K. Post-construction studies for the Mustang Hills and Alta VIII wind energy facilities, Kern County, California. Final report for the first year of operation: July 2012 – October 2013; 2014.

2. Chatfield A, Sonnenberg M, Bay K. Avian and bat mortality monitoring at the Alta-Oak Creek Mojave project, Kern County, California. Final report for the first year of operation March 22, 2011 – June 15, 2012; 2012.

3. Western EcoSystems Technology, Inc. Post-construction fatality surveys for the Barton Chapel wind project: Iberdrola Renewables; 2011.

4. Kronner K, Gritski B, Downes S. Big Horn wind power project wildlife fatality monitoring study: 2006 – 2007; 2008.

5. Jeffrey JD, Bay K, Erickson WP, Sonneberg M, Baker J, Kesterke M, et al. Portland General Electric Biglow Canyon wind farm phase I post-construction avian and bat monitoring first annual report, Sherman County, Oregon: January 2008 – December 2008; 2009.

6. Enk T, Bay K, Sonnenberg M, Boehrs JR. Year 2 avian and bat monitoring report: Biglow Canyon wind farm phase II, Sherman County, Oregon. September 13, 2010 – September 15, 2011; 2012.

7. Derby C, Chodachek K, Bay K, Merrill A. Post-construction fatality survey for the Buffalo Ridge I wind project: May 2009 – May 2010; 2010.

8. Enz T, Bay K. Post-construction fatality surveys for the Juniper Canyon wind project, May 2011 – May 2012; 2012.

9. Gritski R, Kronner K, Downes S. Leaning Juniper wind power project, 2006 – 2008: wildlife monitoring final report; 2008.

10. Derby C, Dahl A, DiDonato G. Post-construction fatality monitoring studies for the PrairieWinds SD1 wind energy facility, South Dakota. Final report: March 2013 – February 2014; 2014.

11. Derby C, Iskali G, Kauffman M, Thorn T, Lyon T, Dahl A. Post-construction monitoring results, Red Hills wind farm, Roger Mills and Custer counties, Oklahoma. Final report: March 2012 – March 2013; 2013.

12. Derby C, Chodachek K, Bay K, Nomani S. Post-construction fatality surveys for the Rugby wind project: Iberdrola Renewables, Inc.: March 2010 – March 2011; 2011.
